# Near-infrared-active Nd–BiO_2-x_/MIL-101(Fe) with vacancy-induced interfaces for solar water purification

**DOI:** 10.1038/s41467-026-77239-z

**Published:** 2026-08-26

**Authors:** Shan Liu, Zhonglin Chen, Jimin Shen, Jingkai Lin, Jing Kang, Congshi Zhu, Simin Long, Yi Zhao, Lanbo Bi, Huayang Zhang, Sihui Zhan

**Affiliations:** 1 State Key Laboratory of Urban-rural Water Resources and Environment, School of Environment, Harbin Institute of Technology, Harbin, China; 2 School of Chemical Engineering, Adelaide University, Adelaide, SA Australia; 3 MOE Key Laboratory of Pollution Processes and Environmental Criteria, College of Environmental Science and Engineering, Nankai University, Tianjin, China; 4 School of Environmental Science and Engineering, Tianjin University, Tianjin, China

**Keywords:** Photocatalysis, Pollution remediation

## Abstract

Engineering vacancy interfaces in semiconductor–metal-organic framework heterostructures provides a promising route to regulate interfacial charge transfer under solar irradiation. Here, we construct a near-infrared-active Nd–BiO_2-x_/MIL-101(Fe) heterostructure, where ultrathin oxygen-vacancy-rich Nd-doped BiO_2-x_ nanosheets (2-5 nm) are anchored onto porous MIL-101(Fe) through Bi–O = C bonds. The vacancy-induced interface establishes an intrinsic built-in electric field and stabilizes a direct Z-scheme charge-transfer pathway. Nd doping generates asymmetric oxygen vacancies that narrow the bandgap, enhance near-infrared absorption, and strengthen O_2_ imprint adsorption. The catalyst exhibits a 1.9-fold increase in bisphenol AF degradation under full-spectrum irradiation compared with near-infrared-filtered light, with singlet oxygen and photogenerated holes acting synergistically. In a flat-plate continuous-flow reactor under natural sunlight, the system achieves stable removal of eleven emerging contaminants with negligible Bi^3+^ leaching. This work presents a vacancy-interface strategy for built-in electric field-driven solar micropollutant removal.

## Abstract (web summary)

Vacancy-engineered Nd-BiO_2-x_/MIL-101(Fe) heterostructures enhance solar-driven micropollutant degradation through utilizing oxygen vacancies and optimized charge transfer mechanisms.

## Introduction

Solar full-spectrum-driven catalytic degradation of aquatic micropollutants has attracted increasing attention owing to its sustainable nature^1–3^. Compared with conventional ultraviolet (UV) photocatalysts, such as TiO_2_^4,5^, bismuth-based semiconductors possess narrower bandgaps, enabling UV-visible (Vis) light absorption, low toxicity, and favorable Bi 6 s electronic features^6,7^. Current strategies for extending the light absorption edge mainly rely on ion doping and heterojunction construction, but effective methods for regulating near-infrared (NIR) light response remain limited. Introducing oxygen vacancies (O_v_) through low-valence metal doping or alkaline hydrothermal treatment further extends light absorption into the near-infrared region (NIR), allowing broader solar utilization. However, the narrowed bandgap accelerates rapid recombination of photoexcited electrons (e^-^) and holes (h^+^)^8^, while lamellar stacking reduces active site exposure and exacerbates photocorrosion, thereby limiting photocatalytic activity^9^. Existing heterojunction designs mainly focus on the role of O_v_ in O_2_ activation, whereas their ability to induce interfacial chemical bonding is rarely considered. In practical systems, additional challenges include reliance on artificial light sources, limited generation and use of reactive oxygen species, and poor selectivity toward target pollutants in complex water matrices^10^. Hence, integrated strategies such as O_v_ engineering, direct Z-scheme heterojunction construction, and adsorption-catalysis synergy are vital for overcoming these limitations^11–13^.

Hydrothermal coordination between hydroxyl groups and bismuth ions can suppress vertical crystal growth, favoring the formation of O_v_ on high-surface-energy facets and generating electron-rich defect sites that extend light absorption into NIR^13,14^. BiO_2-x_ is a typical O_v_-rich n-type semiconductor that can efficiently activate O_2_ through an imprinting effect, lowering the O_2_ adsorption energy and promoting the formation of reactive oxygen species such as superoxide free radicals (•O_2_⁻) and singlet oxygen (^1^O_2_)^15^. Doping metal ions, including Ni^3+^ and Nd^3+^, adjacent to O_v_ introduces an asymmetric local structure and redistributes the electron density around the vacancies, further strengthening O_2_ adsorption and activation^13,14,16^. Despite these benefits, photocorrosion remains a major limitation for bismuth-based photocatalysts, even when O_v_ and metal dopants are introduced.

The role of O_v_ in forming stable chemical bonds at heterojunction interfaces, and in generating an intrinsic built-in electric field (IEF) to direct charge separation, has been largely overlooked^17,18^. Conventional heterojunctions are mainly held together by weak physical interactions, such as van der Waals forces or electrostatic attraction, which can limit interfacial electron coupling because of lattice mismatch and poor orbital overlap^19,20^. Current studies on direct Z-scheme heterojunctions mainly rely on XPS evidence of chemical bonds and analysis of photogenerated carriers’ migration pathway^21,22^. However, the atomic-level connection between vacancy-induced interfacial bonding, charge redistribution, and IEF formation remains insufficiently understood. In contrast, O_v_ can induce the coordination of oxygen-containing functional groups, leading to the formation of robust interfacial chemical bonds with stronger electron interactions. Electron redistribution across these bonds creates depletion or accumulation regions and induces band bending at the interface. This atomic-scale interfacial channel establishes an IEF that drives directional charge migration, suppresses charge recombination, and enhances photocatalytic performance^23,24^. Therefore, O_v_-induced interfacial bonding can improve both structural stability and charge separation, providing a rational strategy for constructing efficient photocatalytic systems.

In conventional photocatalytic oxidation, h^+^ often show limited ability to directly oxidize pollutants, while nonselective radical reactions reduce target selectivity. In complex water matrices, competing species further weaken the coupling between adsorption and catalysis. Improving surface mass transfer, therefore, requires porous materials with large specific areas, such as metal-organic framework (MOF), that can effectively confine and adsorb micropollutants^25,26^. In MIL-101(Fe), carboxyl groups coordinate with Fe³⁺ to form a three-dimensional framework, and their p-π conjugation with benzene rings supports efficient UV/visible light absorption. Dispersing lamellar BiO_2-x_ on MIL-101(Fe) suppresses stacking, stabilizes active sites, and reduces Bi³⁺ leaching in water. In addition, the lower Fermi level of MIL-101(Fe) favors h^+^ migration toward the valence band, improving the use of oxygen-derived reactive species and increasing the probability of direct h^+^ oxidation, while reducing interference from coexisting ions. Integrating O_2_ activation, direct h^+^ oxidation, and adsorption-based pollutant confinement provides an effective strategy for photocatalytic water treatment.

In this study, Nd-BiO_2-x_ with asymmetric O_v_ is prepared by hydrothermal synthesis combined with Nd^3+^ doping and then coupled with MIL-101(Fe) through ball-milling. The O_v_ dually facilitates the formation of a direct Z-scheme heterojunction and promotes O_2_ adsorption-activation via an imprinting effect under full-spectrum irradiation. The heterojunction catalyst Nd-BiO_2-x_/MIL-101Fe (B_x_M_y_), designed with dual-functional O_v_, effectively separates photogenerated carriers. This heterojunction is designed to couple direct h^+^ oxidation with synergistic O_2_ activation, thereby enhancing photocatalytic performance in real water matrices. The synergistic construction of the IEF by O_v_ and Bi−O = C bonds will be demonstrated to facilitate the migration of photogenerated carriers via Kelvin probe force microscopy (KPFM) and density functional theory (DFT) calculations. In-situ electron paramagnetic resonance (EPR) measurements, competitive kinetics, and chemical quenching experiments jointly will demonstrate the heterogeneous catalytic oxidation mechanism. Furthermore, this system, combined with aeration under sunlight, will remove micropollutants from reservoir water matrices, demonstrating its potential for large-scale application.

## Results

### Synthesis of B_x_M_y_ and identification of the Bi − O = C

**Fig. 1:**
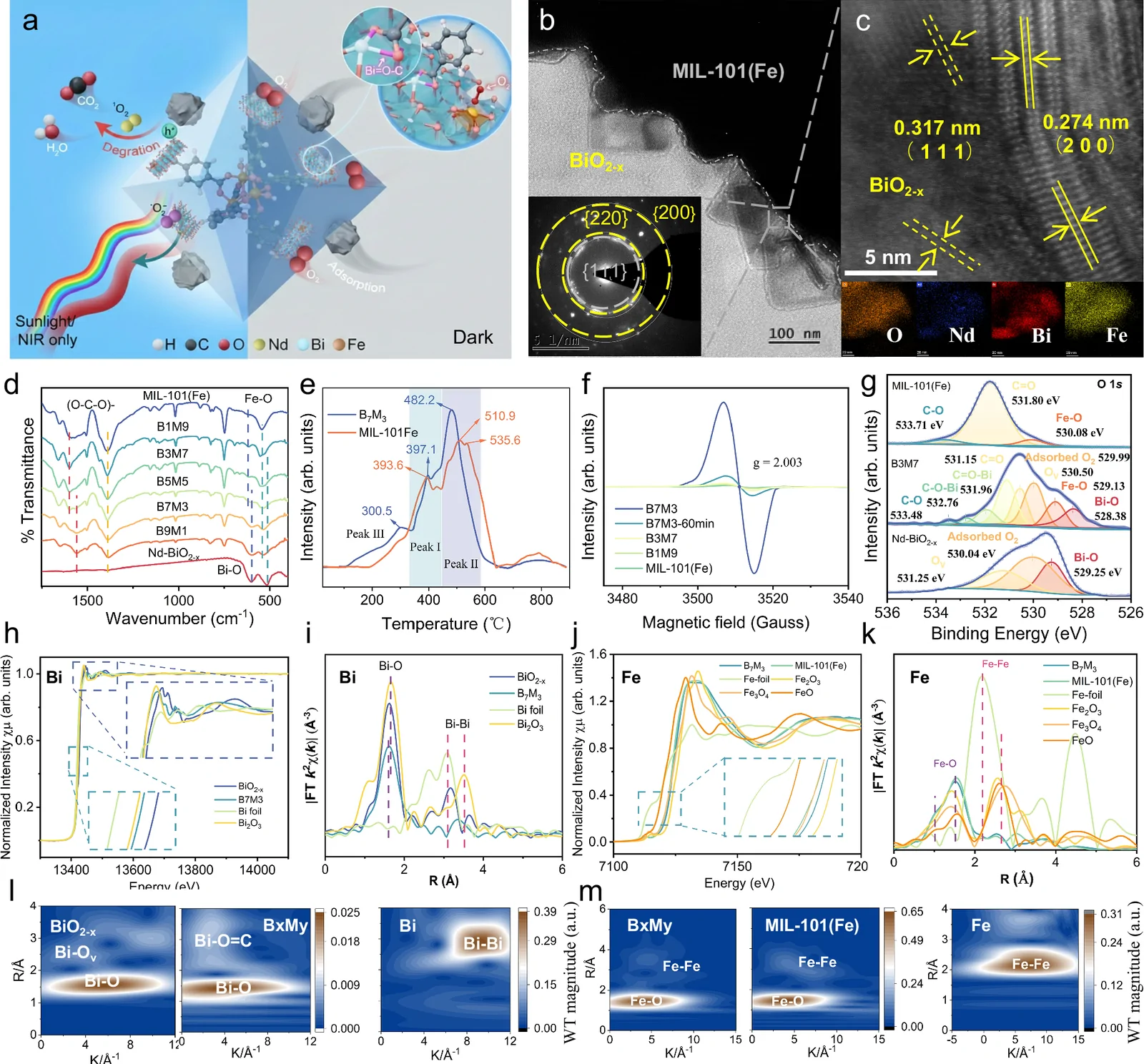
Synthesis and characterization of the B_x_M_y_. **a** Schematic illustration of the adsorption-catalysis synergy of B_x_M_y_ heterojunctions. Adsorption under dark conditions, and after exposure to light, the oxygen adsorbed at the interface is activated and becomes oxygen free radicals, which mineralize organic pollutants (wrinkled rocky-like sphere). **b** HRTEM image (insert: selected-area electron diffraction), Data are representative of two independent experiments, both yielding similar results. **c** Aberration-Corrected-TEM and EDS elemental mapping of the B_7_M_3_. **d** FTIR spectra of Nd-BiO_2-x_, MIL-101(Fe), and B_x_M_y_ heterojunctions, where two dashed lines indicate peak shifts. Data are representative of two independent experiments, both yielding similar results. **e** H_2_-TPR, **f** EPR spectra, **g** XPS spectra of O 1 *s*, **h** Bi L_3_-edge and **j** Fe K-edge normalized intensity in XANES spectra, **i** and **k** k^2^-weighted FT-EXAFS spectra, **l** and **m** wavelet transform analysis of the as-prepared samples. The corresponding unedited TEM images for (**b**) and (**c**) are provided in Supplementary Fig. 4. Source data are provided as a Source Data file.

A combined hydrothermal treatment and ball-milling strategy was employed to fabricate a series of vacancy-rich Nd-BiO_2-x_/MIL-101(Fe) (B_x_M_y_) (Fig. 1a and Fig. S1). The sheet material of the BiO_2-x_ attached to the octahedron of the MIL-101(Fe) was observed in the scanning electron microscope (SEM) image (Fig. S2). Likewise, a heterostructure composed of MIL-101(Fe) and BiO_2-x_ layer can be observed in the high-angle annular dark-field scanning transmission electron microscopy (HAADF-STEM) and its corresponding electron diffraction image of B_x_M_y_ (Fig. 1b and Fig. S3a–c). The strained heterojunction shows local lattice distortion and close interfacial contact, which may create defect-rich regions on the Nd-BiO_2-x_ surface and facilitate interfacial electronic coupling. Although clear lattice fringes of MIL-101(Fe) are difficult to resolve in the high-resolution TEM image because MOF frameworks are sensitive to high-energy electron-beam irradiation, the retained octahedral morphology and the close interfacial contact between BiO_2-x_ and MIL-101(Fe) support the successful integration of the two components. In the selected area electron diffraction (SAED) pattern, diffraction spots corresponding to the {101} lattice planes of MIL-101(Fe) are also observed. Aberration-Corrected-TEM images (Fig. 1c) show real lattice spacings of 0.317 nm and 0.274 nm, corresponding to the (111) and (200) planes, respectively, indicating that BiO_2-x_ preferentially exposes the (01$\bar{1}$) facet^12^. Element mapping (Fig. 1c and Fig. S3d) confirms that Nd-BiO_2-x_ is uniformly anchored on the surface of MIL-101(Fe), with homogeneous distributions of Nd, Bi, Fe, and O. These morphological, diffraction, and elemental mapping results collectively confirm the nanoscale contact between Nd-BiO_2-x_ and MIL-101(Fe), which provides structural evidence for heterojunction construction. To further elucidate the chemical state of Nd, XAFS analysis was performed (Fig. S5), confirming that Nd exists mainly in an ionic form. Neodymium (Nd^3^⁺) is preferentially located near O_v_ in BiO_2-x_, forming an asymmetric Nd–O_v_–Bi local coordination environment^13,16^. The weak carbon signal indicates that carbon is mainly embedded within the framework. The faint edge contrast of the nanosheets suggests an ultrathin structure, consistent with the 2 − 5 nm thickness of BiO_2-x_ measured by atomic force microscopy (AFM) image (Fig. S6). Powder X-ray diffraction (XRD) patterns show that BiO_2-x_ retains its original crystal structure after ball milling (Fig. S7). With increasing BiO_2-x_ content, the diffraction peaks indexed to the (110), (200), (220), and (311) planes (JCPDS No. 47-1057) become more pronounced.

Considering the imprinting effect of O_v_, it is proposed that O_v_ formed during heterojunction construction can induce bonding between oxygen-containing functional groups with metals. A comparison of samples before and after ball milling shows clear vibrational features of Bi−O, Fe−O, and (O-C-O)- in FTIR spectra (Fig. 1d and Fig. S8). After ball-milling, B_7_M_3_ exhibits red shifts in the (O-C-O)- and Fe−O stretching vibrations relative to MIL-101(Fe), from 1600 cm^-1^ to 1561 cm^-1^ and from 543 cm^-1^ to 536 cm^-1^, respectively. In contrast, the Bi–O stretching modes of BiO_2-x_ at 514 cm⁻^1^ (associated with the (01$\bar{1}$) facet) and 601 cm⁻^1^ (perpendicular to this facet) shift to higher wavenumbers at 536 and 618 cm⁻^1^, respectively^27^. With increasing MIL-101(Fe) content, the intensity of the Bi–O vibration on the exposed (01$\bar{1}$) facet decreases markedly, indicating interfacial bonding between C = O or Fe–O groups and Bi–O or Bi–O_v_ sites on BiO_2-x_. These peak shifts indicate that the two components are not simply physically mixed, but are chemically coupled through vacancy-induced interfacial bonding. Raman spectra further confirm the presence of O_v_, and the bonding features are consistent with the FTIR results (Fig. S9).

H_2_ temperature-programmed reduction (H_2_-TPR) and O_2_ Temperature-programmed desorption (O_2_-TPD) were used to evaluate the properties of O_v_. As shown in Fig. 1e, B_7_M_3_ exhibits two main reduction peaks at 397.1 and 482.2 °C, which are assigned to the reduction of Fe (III) to Fe (II) or Bi (III) to Bi, and further reduction of Fe (II) to Fe. Compared with MIL-101(Fe), the overall peak intensity increases and the second peak shifts to a lower temperature, indicating enhanced reducibility^28^. In addition, peak III at 300.5 °C becomes broader in B_7_M_3_, which is attributed to the reduction of the adsorbed O⁻/O_2_ species. O_2_-TPD results show that B_7_M_3_ releases a larger amount of O⁻/O_2_ (0.153 mmol g^-1^) than MIL-101(Fe) (0.139 mmol g^-1^) (Fig. S10 and Table S1). EPR spectra (Fig. 1f) display an asymmetric signal at g = 2.003 for B_x_M_y_, arising from unpaired electrons trapped at O_v_, whereas MIL-101(Fe) shows no such signal^29^. The strongest vacancy signal is observed for B_7_M_3_ after 30 min of ball milling, whereas prolonging the milling to 60 min weakens the signal. These results indicate that O_v_ in BiO_2-x_ act as active sites that promote interfacial bonding, enhance catalyst reducibility, facilitate oxygen species adsorption, and improve oxygen-related catalytic activity.

The interfacial properties and bonding features were further examined to clarify the heterointerface interaction. XPS O 1 *s* and Bi 4 *f* spectra of the prepared materials are shown in Fig. 1g and Fig. S11, respectively. Compared with the pristine materials, B_7_M_3_ exhibits two additional O 1 *s* components with binding energies located between those of C–O (533.71 eV) and C = O (531.80 eV), which is attributed to the formation of Bi–O = C bonds between Bi–O_v_ sites and the carboxyl groups of MIL-101(Fe)^10^. The resolved O 1 *s* peaks corresponding to O_v_ and chemisorbed O_2_ in BiO_2-x_ and B_7_M_3_ further confirm the presence of O_v_. The Bi 4 *f* spectra show clear binding energy shifts after heterojunction formation, indicating interfacial electron redistribution between Nd-BiO_2-x_ and MIL−101(Fe). Combined with the KPFM results, this charge redistribution is attributed to electron transfer from Nd-BiO_2-x_ to MIL−101(Fe) under dark equilibrium because MIL−101(Fe) has a higher work function. As a result, upon contact, Nd-BiO_2-x_ becomes relatively electron-deficient, while MIL−101(Fe) becomes electron-rich, establishing an intrinsic electric field directed from Nd-BiO_2-x_ to MIL-101(Fe). Under illumination, this built-in electric field facilitates directional charge migration and spatial separation of photogenerated carriers^30^. In addition, changes in contact angle, Brunauer–Emmett–Teller (BET) surface area, and zeta potential provide indirect but consistent evidence for the formation of Bi–O = C interfacial bonds (Figs. S12–S14, Table S2, and Note S1). X-ray Absorption Fine Structure (XAFS) further clarifies the electronic structure and coordination environment of Bi atoms. X-ray Absorption Near-Edge Structure (XANES) at the L_3_-edge (Fig. 1h) shows that the white line of Bi in BiO_2-x_ lies between those of α-Bi_2_O_3_ and NaBiO₃, indicating the coexistence of Bi(Ⅲ) and Bi(Ⅴ) valence states^31^. The k^2^-weighted Fourier-transformed Extended X-ray Absorption Fine Structure (EXAFS) spectra reveal that the coordination signals of surface Bi are primarily concentrated at 1.7 Å and 3.2 Å (Fig. 1i), which are attributed to Bi–O and Bi–Bi coordinations. According to the EXAFS shell fitting results (Table S3), the Bi-O bond length in BiO_2-x_ is 2.06 Å with a coordination number of 4.1, while the Bi-Bi bond length is 3.66 Å with a coordination number of 2.1^32^. O_v_ cause partial Bi–O bond elongation to 2.55 Å and 3.01 Å, with coordination numbers reduced to 1.2 and 1.7, respectively, indicating the presence of abundant oxygen vacancies. The overall elongation of the Bi-Bi bond lengths is attributed to carboxyl coordination. The fitted Fe-O bond length in MIL-101(Fe) are 1.90 and 2.04 Å, with a coordination number of 3. The Fe-Fe bond lengths are 2.92 and 3.35 Å with fitted coordination number of 1.0 and 1.1, respectively. Compared with B_7_M_3_ (Fe-Fe, 3.01 Å), the presence of a second Fe-Fe shell in MIL-101(Fe) indicates the retention of a more intact intrinsic crystalline structure and higher long-range order (Fig. 1j and Fig. 1k). In B_7_M_3_, O = C coordinate with Bi atoms from bismuth oxides through O_v_-induced interfacial bonding, introducing defects and local disorder, which disrupts the long-range Fe-Fe coordination structure, so that only one Fe-Fe shell is resolved during fitting. Wavelet transform (Fig. 1l**and** Fig. 1m) shows that BiO_2-x_ is mainly coordinated by Bi–O and Bi–O_v_, whereas B_7_M_3_ forms a new Bi–O = C signal due to O_v_-induced carbonyl and carboxyl bonding.

### Exploration of the driving force of the interfacial electric field

**Fig. 2:**
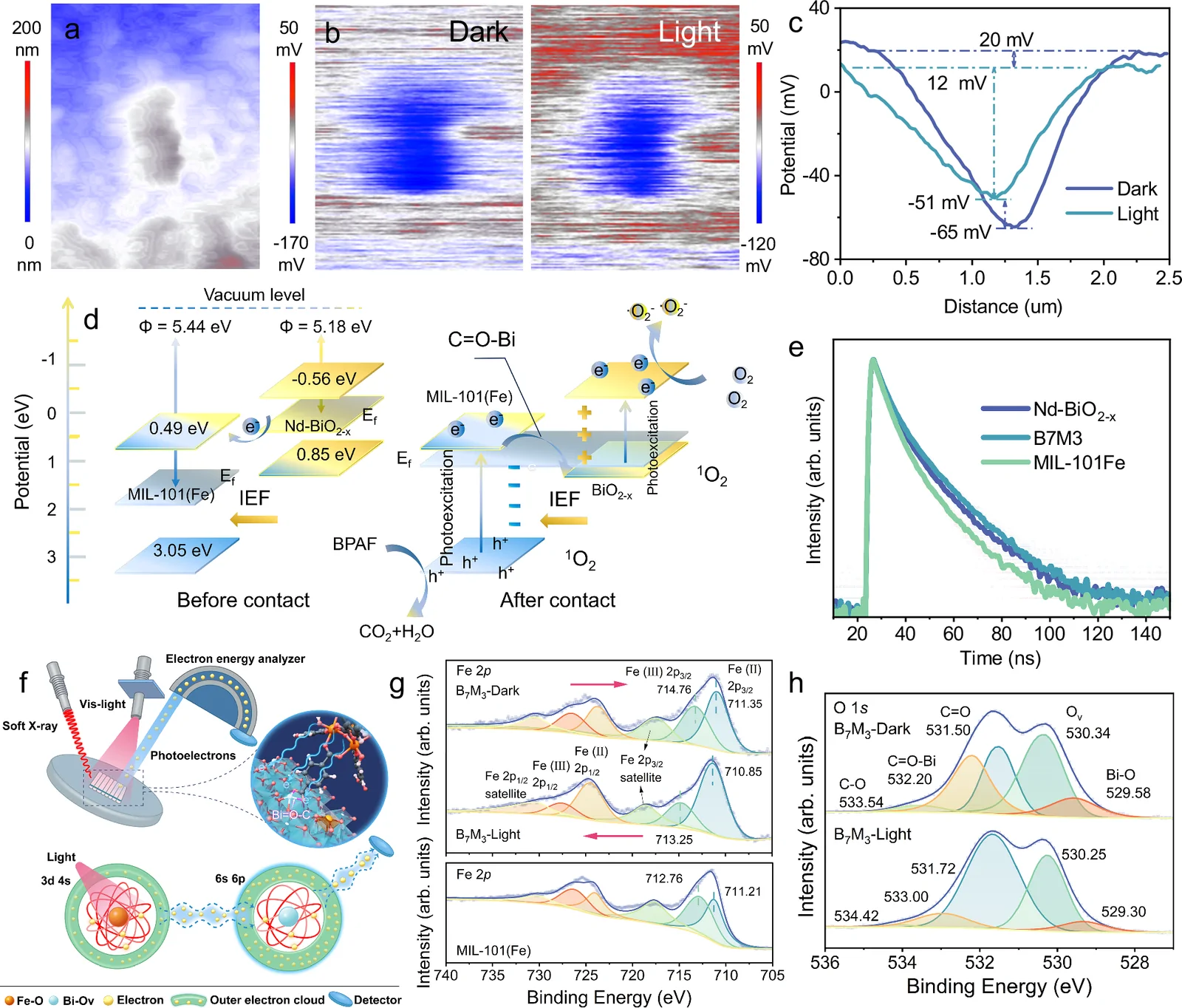
Electron transfer pathway and photophysical and electrochemical measurements. **a**–**c** KPFM images and corresponding surface potential distribution of B_x_M_y_ heterojunctions in darkness and under light. **d** Schematic illustration of the Nd-BiO_2−x_/MIL-101(Fe) direct Z-scheme heterojunction with an interfacial electric field (IEF). Φ is the work function, and BPAF is the pollutant bisphenol AF. **e** Time-resolved photoluminescence (TRPL) decay curves of the as-prepared samples. **f** Schematic illustration of the in situ XPS setup. **g** high−resolution Fe 2*p* and (**h**) O 1 *s* spectra before and after light. Source data are provided as a Source Data file.

To clarify the electron transfer pathway and optical behavior, photophysical and electrochemical characterizations were performed (Fig. 2). Kelvin probe force microscopy (KPFM) was used to probe surface potential distributions under dark and illuminated conditions, providing insight into the built-in electric field (IEF) between Nd–BiO_2-x_ and MIL−101(Fe) (Fig. 2a, b)^33,34^. In the dark, the surface potential difference (SPD) between the center and edge of B_7_M_3_ reaches 85 mV (Fig. 2c), slightly higher than that of Nd–BiO_2-x_ (82 mV, Fig. S15). The central region associated with MIL-101(Fe) shows a lower potential ( − 65 mV) than the edge region dominated by BiO_2-x_ (≈20 mV). In SKPM mode, the measured potential is inversely related to the sample work function according to Eq. (1), where Φ_probe_ and Φ_sample_ represent the work function of probe and sample, respectively, and *e* is the elementary charge^35^.

1 $${V}_{{probe}}=\frac{{\varPhi }_{{probe}}-{\varPhi }_{{sample}}}{e}$$

Therefore, the lower surface potential of MIL−101(Fe) indicates its higher work function compared with Nd-BiO_2-x_. After contact, electrons transfer from Nd-BiO_2-x_ to MIL-101(Fe) until Fermi-level equilibrium is reached, with Nd-BiO_2-x_ serving as the electron donor and MIL-101(Fe) as the electron acceptor in the dark state^36^. Under irradiation, photoinduced charge redistribution occurs across the heterojunction, leading to an increase in the central potential from −65 to −51 mV and a decrease at the edges from 20 to 12 mV. These changes indicate that the light-induced charge transfer process differs from the initial dark-state equilibration. Photogenerated electrons preferentially accumulate on BiO_2-x_, while holes remain on MIL-101(Fe), consistent with the direct Z-scheme charge transfer pathway^37^. Under visible and near-infrared irradiation, the surface potential difference of Nd–BiO_2-x_ decreases by 22 and 14 mV, respectively, both smaller than that of B_7_M_3_ (Fig. S15), indicating more effective charge separation in the heterojunction. UV–Vis diffuse reflectance spectra show that Nd–BiO_2-x_ enhances light absorption of B_x_M_y_ compared with MIL-101(Fe) (Fig. S16). The flat-band potentials and band gaps derived from Mott–Schottky analysis and Kubelka–Munk plots (Figure S17, 18) reveal that the composite exhibits an effective band structure distinct from its components, further supporting the formation of a Z-scheme heterojunction^38^.

In n-type semiconductor photocatalysts, electrons are the dominant charge carriers and play a central role in catalytic reactions. The energy level alignment between Nd-BiO_2-x_ and MIL-101(Fe) is schematically shown in Fig. 2d. Density functional theory calculations with dipole corrections were used to determine the work functions of the two components (Fig. S19). The calculated work function of MIL-101(Fe) is 5.44 eV, which is higher than that of Nd-BiO_2-x_ (5.18 eV), indicating that electrons migrate from Nd-BiO_2-x_ to MIL-101(Fe) upon contact to equilibrate their Fermi levels^39,40^. As a result, Nd-BiO_2-x_ becomes positively charged, while MIL-101(Fe) becomes negatively charged, establishing a built-in electric field at the interface. Under illumination, this built-in electric field facilitates directional charge migration and spatial separation of photogenerated carriers, driving the direct Z-scheme pathway in which electrons accumulate on Nd-BiO_2-x_ and holes remain on MIL-101(Fe)^41^. In addition, O_v_-associated undercoordinated Bi/Nd sites introduce localized defect states and act as electron-accumulation centers on the Nd-BiO_2-x_ surface, facilitating subsequent redox reactions^11–13^.

Electrochemical impedance spectroscopy (EIS), transient photocurrent measurements, and time-resolved photoluminescence (TRPL) were further employed to verify heterojunction formation and the IEF. As shown in Fig. S20a and Table S4, B_7_M_3_ exhibits a much lower charge transfer resistance (2.74 kΩ) than Nd–BiO_2-x_ (3.71 kΩ) and MIL-101(Fe) (10.87 kΩ), indicating more efficient interfacial charge transport. Transient photocurrent responses measured following the Kanata–Kito model show that the photocurrent density follows the order B_7_M_3_ > B_5_M_5_ > B_3_M_7_ > Nd–BiO_2-x_ (Fig. S20b)^42^. Upon light irradiation, the photocurrent rapidly increases and reaches a steady state, reflecting continuous carrier transport across the heterojunction under reaction conditions^43,44^. TRPL analysis further shows that the average carrier lifetime (Τ_ave_) increases from 4.28 ns for MIL-101(Fe) to 5.41 ns for B_7_M_3_ (Fig. 2e and Table S5). To gain deeper insights into the interfacial charge transfer mechanism within the B_x_M_y_ heterojunction, comparative in situ XPS analyses were conducted under dark and illuminated conditions. Figure 2f illustrates the mechanism of synchronous illumination X-ray photoelectron spectroscopy device simply^45^. The Fe 2*p* spectra (Fig. 2g) show clear electronic changes around Fe sites after coupling with Nd-BiO_2-x_, supporting interfacial electron redistribution between the two components. Under light irradiation, the binding energies of the Fe 2*p* peaks and the O 1 *s* peaks assigned to C = O and C–O bonds shift positively, whereas the O 1 *s* peak assigned to Bi–O shift negatively compared with those in the dark state (Fig. 2h). These opposite binding energy shifts reveal photoinduced charge redistribution across the heterojunction. Specifically, photogenerated electrons accumulate on Nd-BiO_2-x_, while holes remain on MIL-101(Fe), consistent with the direct Z-scheme charge transfer pathway driven by the interfacial electric field. These results confirm that the built-in electric field, together with the Bi–O = C interfacial bond, promotes charge separation and carrier migration to reactive sites.

### Evaluation of full-spectrum photocatalytic performance

**Fig. 3:**
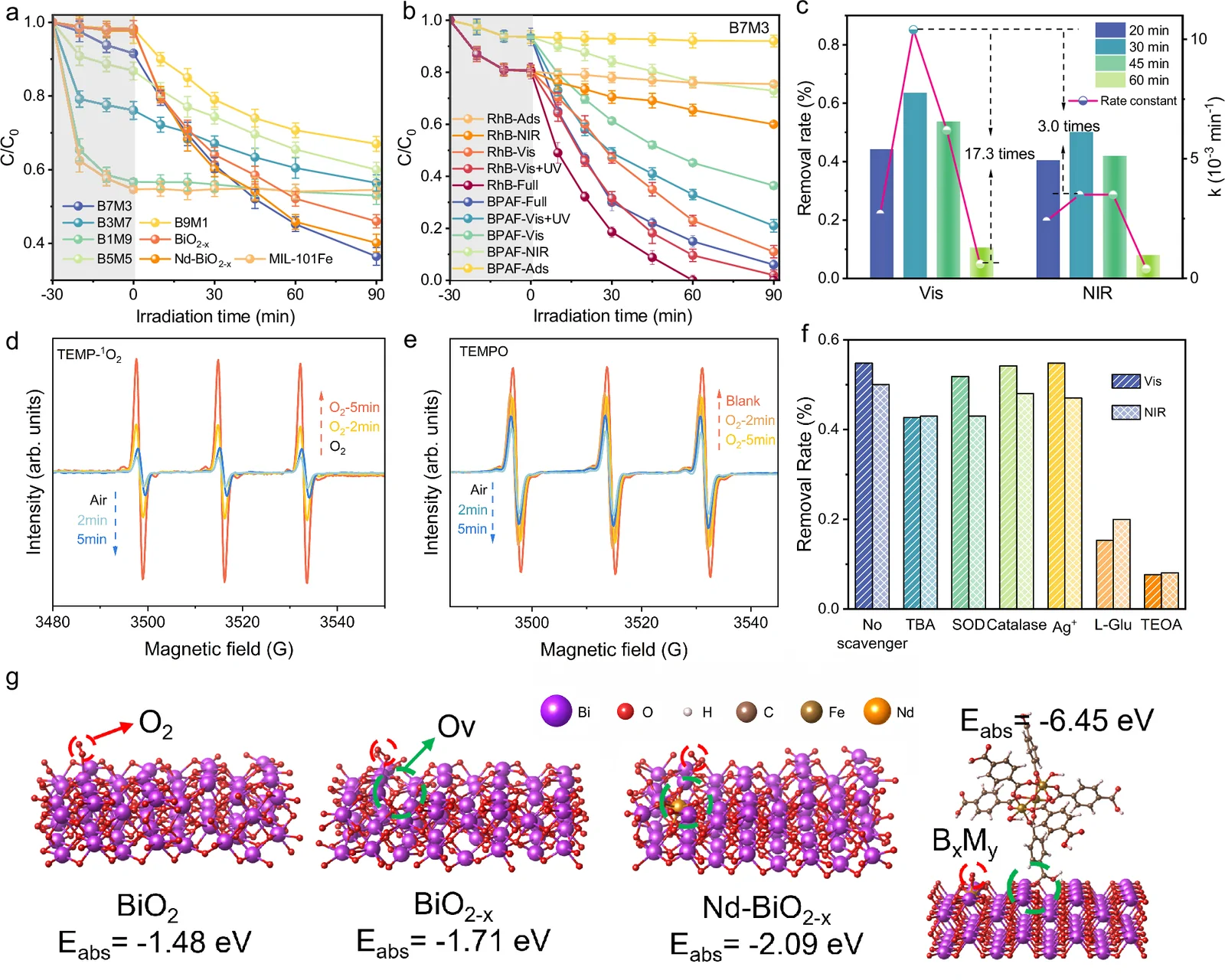
Photocatalytic performance of B_x_M_y_. **a** Degradation curves of BPAF with a series B_x_M_y_ under Vis light. **b** Photocatalytic efficiency of bisphenol AF (BPAF) and rhodamine B (RhB) with B_7_M_3_ under different light conditions. The shaded area represents the dark reaction, while the white background indicates the illumination. **c** Removal rate and apparent reaction rate constant of B_7_M_3_ with various ball-milling times. **d**,**e** EPR signals for TEMP-^1^O_2_ and TEMPO. **f** Effects of quenching agents on BPAF removal under Vis and NIR light. **g** The O_2_ adsorption energies of as-prepared samples. Reaction conditions: [BPAF] = [RhB] = 0.06 mM, [Cat.] = 0.2 g L^-1^, [TEMP]_0_ = 100 mM, [TEMPO]_0_ = 100 mM, 20×AM 1.5 G illumination, [Scavenger] = 10 mM, [SOD]_0_ = 100 mg L^-1^, pH_0_ = 7.8, and *T* = 25 °C ± 3 °C. Data for (**a**) and (**b**) are presented as mean values  ±  SD (*n*  =  2). Source data are provided as a Source Data file.

MIL-101(Fe) contains abundant surface carboxyl groups and an accessible porous structure, which favors micropollutant adsorption^46,47^. Bisphenol AF (BPAF) and Rhodamine B (RhB) were selected as representative target pollutants to evaluate the adsorption and photocatalytic performance, with BPAF serving as a toxic bisphenol substitute and RhB as a common photocatalytic model compound. As shown in Fig. 3a, BPAF adsorption increases with MIL-101(Fe) content, reaching 46% for MIL-101(Fe), while RhB shows a similar adsorption removal of 51%. However, after adsorption equilibrium, MIL-101(Fe) shows negligible additional removal under light irradiation, indicating its role mainly as an adsorbent rather than an active photocatalyst. Nd-BiO_2-x_ and B_9_M_1_ show limited adsorption, whereas B_1_M_9_ exhibits strong adsorption but poor photocatalytic activity. Under visible light, B_7_M_3_ achieves the highest BPAF removal of 64% after 90 min through coupled adsorption and photocatalysis, while B_5_M_5_ shows lower efficiency, likely due to partial coverage of exposed Bi–O and Bi–O_v_ sites on the (01$\bar{1}$) surface of Nd-BiO_2-x_ by excess MIL-101(Fe). Under NIR irradiation, Nd-BiO_2-x_ shows limited activity, whereas B_7_M_3_ reaches 51% BPAF removal, confirming its NIR-driven catalytic performance (Fig. S21). The RhB removal results show a similar trend under visible and NIR irradiation (Fig. S22), further supporting the adsorption–photocatalysis synergy of the Nd-BiO_2-x_/MIL-101(Fe) heterojunction. Because RhB has better light absorption, its dye polarization effect leads to a faster degradation rate (Fig. S23). Simulated full-spectrum irradiation (Fig. 3b) further enhances the performance of B_7_M_3_, with added UV light ( < 420 nm) markedly increasing degradation efficiency. After 90 min under UV–Vis irradiation, BPAF and RhB removal reach 80% and 99.9%, respectively. Kinetic analysis based on a pseudo-first-order model shows that full-spectrum irradiation increases the apparent rate constants for BPAF and RhB degradation by factors of 1.9 and 1.2, respectively, enabling complete RhB removal and 94% BPAF elimination. As shown in Fig. 3c, B_7_M_3_ prepared with 30 min ball milling exhibits the highest rate constant under visible light (1.04 × 10⁻^2^ min⁻^1^), nearly three times that under NIR light. Prolonging the ball milling to 60 min sharply reduces activity, with BPAF removal limited to 11% under visible light and 8% under NIR irradiation, highlighting the need to control the milling time to preserve the pore structure. As shown in Fig. S24 and Table S6, B_7_M_3_ exhibits the highest catalyst-dose-normalized rate constant, *k* (L min⁻^1^ g⁻^1^), under xenon-lamp irradiation, demonstrating the high efficiency of the present system.

The reusability and structural stability of the photocatalyst are important for its potential application in environmental remediation. To evaluate the cycling stability of B_7_M_3_, five consecutive degradation cycles were conducted for BPAF and RhB under identical experimental conditions. As shown in Fig. S25, B_7_M_3_ maintains good catalytic durability during repeated use. In the first cycle, the removal efficiencies of BPAF and RhB reach 93.2% and 98.5%, respectively. After five successive cycles, the removal efficiencies remain at 78.4% for BPAF and 85.1% for RhB. The slight decrease in performance is mainly attributed to minor catalyst loss during recovery and partial occupation of surface active sites by adsorbed degradation intermediates. To further examine the structural stability of B_7_M_3_, XRD and SEM characterizations were performed before and after the five-cycle tests. As shown in Fig. S26, the spent catalyst largely retains its original morphology after cycling, and its XRD pattern remains consistent with that of the pristine catalyst, confirming the good structural stability of B_7_M_3_.

### Reactive oxygen species identification

Due to the imprinting effect of the Ov, oxygen (O_2_) readily adsorbs at O_v_ sites, where photogenerated electrons can directly transfer to O_2_ to form •O_2_⁻ and ^1^O_2_^28,48,49^. In situ EPR spectroscopy (Fig. 3d, e and Fig. S27) combined with chemical quenching experiments (Fig. 3f) was used to identify the reactive oxygen species involved^15^. Upon continuous introduction of air and O_2_, the EPR signals of TEMP-^1^O_2_ and DMPO-•O_2_⁻ sustained conversion of O_2_ into reactive species during photocatalysis. The signal intensity under O_2_ is much higher than under air, confirming that micropollutant removal is strongly dependent on O_2_ availability. TEMPO, a paramagnetic probe with a characteristic triplet EPR signal, is reduced by photogenerated electrons to TEMP, leading to signal attenuation. Under an air atmosphere, the gradual decrease of the TEMPO signal reflects continuous electron generation. In contrast, when O_2_ is continuously supplied, the TEMPO signal remains nearly unchanged because O_2_ competes for electrons to produce ^1^O_2_, which subsequently oxidizes TEMP back to TEMPO.

The effects of different scavengers on BPAF degradation are summarized in Table S7. Under both visible and NIR irradiation, tert-butanol (TBA, *k*_·OH_** =** 5.2 × 10^10 ^M^-1^ S^-1^) has little influence on BPAF removal (Fig. 3f and S28a, b), indicating that •OH is not the dominant reactive species, consistent with the absence of DMPO–•OH signals in the EPR spectra^28^. In contrast, the addition of L-tyrosine (L-Tyr, *k*^1^O_2_** =** ~10^8 ^M^-1^ S^-1^)^50^ significantly suppresses BPAF degradation, leading to a 42% decrease in removal efficiency, whereas superoxide dismutase shows no measurable effect. Triethanolamine (TEOA), a typical h^+^ scavenger, almost completely inhibits BPAF removal^14,51^. This strong inhibition arises from the chelation of TEOA with both iron and bismuth species, which blocks hole-driven reactions and suppresses superoxide generation at O_v_ sites. These results indicate that ^1^O_2_ and photogenerated h^+^ are the main reactive species in this system. For RhB, scavenging •OH, •O_2_⁻, and H_2_O_2_ only slightly affected the degradation performance, while the quenching of ^1^O_2_ and h^+^ causes clear inhibition under both visible and NIR irradiation (Fig. S29a). Based on the TEMPO results, O_2_ concentration directly affects electron transfer and ROS formation. Therefore, BPAF degradation was evaluated under different gas atmospheres (Fig. S28c). Under visible light, removal efficiencies at 60 min reach 70% under O_2_ and 40% under N_2_, while under NIR irradiation, the corresponding values are 40% and 20%. Thus, O_2_ increases the photocatalytic removal rate by approximately 20–30%. Based on the results of the EPR and radical scavenging experiments, ^1^O_2_, and h^+^ are the primary reactive species in the system. To provide further quantitative evidence, we have added measurements of the main reactive species using ABDA for ^1^O_2_, NBT for •O_2_ˉ, and KI oxidation for photogenerated h⁺ (Table S8). Under visible and NIR light irradiation, the exposed h⁺ equivalents are 59.64 and 19.46 μM, respectively, while the ^1^O_2_ are 7.2 and 2.9 μM, with superoxide radicals being almost negligible. To further quantify the relative contributions of ^1^O_2_ and h^+^, competitive kinetic analysis combined with a standard ^1^O_2_-generating system (NaClO/H_2_O_2_) was applied (Note S2)^52,53^. For B_7_M_3_, the contribution ratios of ^1^O_2_ to BPAF degradation under visible and NIR irradiation are estimated to be 36% and 31%, respectively, while the corresponding h⁺ contributions are 64% and 69%. These results demonstrate that BPAF degradation is mainly driven by the synergistic action of ^1^O_2_ and photogenerated h⁺, with h⁺ playing a larger role under both light conditions. For RhB degradation, the contributions of ^1^O_2_ and h⁺ are estimated to be 56% and 44%, respectively, further confirming that these two active species dominate the pollutant removal process. The different contribution ratios are related to the molecular structures of the two pollutants. BPAF contains fluorinated aromatic rings, where the electron-withdrawing fluorine groups reduce the electron density of the aromatic structure and make electrophilic oxidation more difficult. In contrast, RhB has an electron-rich conjugated chromophore, which is more readily attacked by ^1^O_2_ and directly oxidized by photogenerated h⁺. Therefore, RhB removal mainly proceeds through oxidative destruction of the chromophoric conjugated structure, while BPAF degradation requires stronger hole-driven oxidation together with ^1^O_2_-mediated transformation.

To evaluate oxygen enrichment induced by the imprinting effect, O_2_ adsorption energies were calculated for catalysts with oxygen vacancies and Nd doping (Fig. 3g). For the atomic coordinates of the optimized computational model, please refer to Supplementary Data 1. Compared with stoichiometric BiO_2_, BiO_2-x_ shows a lower adsorption energy, decreasing from −1.48 to −1.71 eV, with O_2_ adopting a Pauling-type configuration. Introducing Nd near oxygen vacancies forms an asymmetric Nd–O_v_–Bi structure and further reduces the adsorption energy to −2.09 eV. After heterojunction formation, electron transfer from BiO_2-x_ to MIL-101(Fe) drives a transition of O_2_ adsorption from Pauling-type to Yeager-type, resulting in a much stronger adsorption energy ( − 6.45 eV). These results suggest that ionic Nd^3+^ modulates the electronic and coordination environment of O_v_ sites, making them electron-rich centers for O_2_ adsorption and activation under irradiation.

### Direct oxidation with Bi-O = C by h^+^

**Fig. 4:**
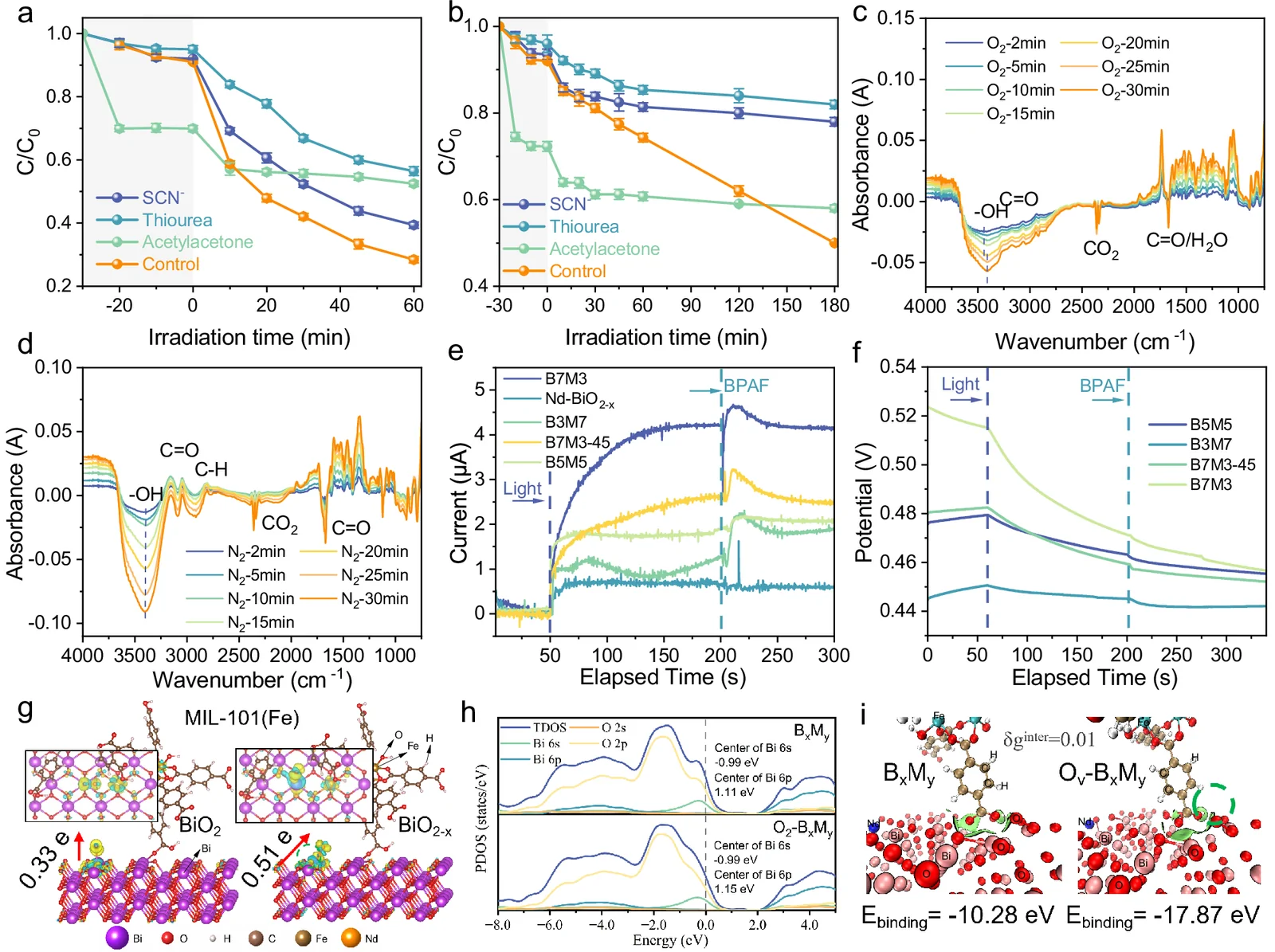
Identification of metal active sites and photogenerated holes. Evaluation of active sites through metal masking under **a** Vis and **b** NIR light. The shaded area represents the dark reaction, while the white background indicates the illumination. Under continuous introduction of **c** oxygen and **d** nitrogen, the in-situ FTIR spectra of the catalyst in water. **e** Photocurrent diagrams and **f** in-situ open-circuit voltages after the addition of bisphenol AF (BPAF). **g** B_x_M_y_/O_2_ interface, with blue and yellow indicating charge accumulation and depletion, respectively. **h** The partial density of states (PDOS) of the optimized configurations of B_x_M_y_ and B_x_M_y_/O_2_. **i** The independent gradient model based on Hirshfeld partitioning (IGMH) and the binding energy of MIL-101(Fe) clusters on the (01$\bar{1}$) surface of BiO_2**-**x_. Reaction conditions: [Cat.] = 0.2 g L^-1^, [BPAF] = 0.06 mM, [Ligand] = 10 mM, pH_0_ = 7.8, and *T* = 25 °C ± 3 °C. Data for (a) and (**b**) are presented as mean values  ±  SD (*n*  =  2). Source data are provided as a Source Data file.

Directional migration of photogenerated electrons enhances the oxidative ability of photogenerated holes in the valence band^54,55^. To assess the roles of h^+^ and metal active sites, a series of metal chelating agents was introduced (Fig. 4a, b), and the stability constants of the corresponding complexes are listed in Table S9. The addition of SCN⁻ decreases BPAF removal by 11 and 28 percentage points under visible and near-infrared light, respectively, indicating partial suppression of metal-centered reactions. In dark conditions, acetylacetone increases BPAF adsorption to about 30% and, under illumination, provides a continuous electron source to Fe^3^⁺ in B_7_M_3_, leading to a photocatalytic removal of approximately 12%. Acetylacetone coordinates with iron ions in a bidentate mode, replacing surface water and hydroxyl groups and increasing surface hydrophobicity. Electron transfer from acetylacetone through Bi–O = C bonds to oxygen vacancies promotes oxygen radical generation and accelerates early-stage BPAF degradation. In contrast, thiourea addition results in pronounced decreases in removal efficiency, by 28% under visible light and 32% under near-infrared light. Thiourea preferentially binds to bismuth species and can also quench superoxide radicals in solution. For RhB removal, thiourea also shows the strongest inhibitory effect among the tested masking agents (Fig. S29b), suggesting that Bi-related sites play a major role in dye oxidation. These results indicate that Bi-site-assisted activation, ^1^O_2_, and photogenerated h⁺ dominate RhB degradation, while Fe-related hole-mediated reactions provide a secondary contribution.

In situ FTIR measurements were performed under continuous N_2_ or O_2_ flow during illumination to monitor changes in surface functional groups (Fig. 4c, d)^56,57^. After subtracting the background signal of the catalyst in water, clear differences are observed under oxygenated photocatalytic conditions. The carbonyl and hydroxyl bands of the carboxyl groups in phthalic acid weaken and shift to lower wavenumbers, indicating electron transfer from the carboxyl groups to BiO_2-x_ through Bi–O = C interfacial bonds. In contrast, under N_2_, no obvious peak shift is detected, and only a slight decrease in the hydroxyl signal is observed. In situ FTIR spectra recorded without background subtraction show negligible spectral changes (Fig. S30), confirming the structural stability of the catalyst during operation. In addition, under irradiation, new features at 2940 and 3070 cm⁻^1^ appear, which are assigned to double-bond stretching and overtone vibrations of the benzene ring and Bi–O = C, arising from photon energy input and the driving force of the built-in electric field.

Photogenerated holes at iron sites can participate in interfacial reactions with water molecules, forming carbonyl or hydroxyl species that contribute to BPAF oxidation^58,59^. Under illumination, the formation of Bi–O = C interfacial bonds induces directed charge migration, giving B_7_M_3_ the highest photocurrent response (Fig. 4e). After BPAF addition, adsorbed BPAF donates electrons to iron centers, and these electrons are subsequently transferred through Bi–O = C bonds, further promoting charge transport. The photocurrent of B_7_M_3_ prepared with 30 min ball milling is about 1.5 times that of the 45 min sample and nearly nine times higher than that of pristine Nd–BiO_2-x_. To evaluate changes in oxidative potential, open-circuit voltage measurements were performed (Fig. 4f)^58^. Upon illumination, the built-in electric field drives electron transfer to reduce O_2_ to •O_2_⁻, while iron sites interact with water to form hydroxyl species, leading to a decrease in surface potential. After BPAF addition, a rapid potential drop is observed, with B_7_M_3_ showing the largest decrease of 55 mV (from 515 to 460 mV), indicating a strong capability for direct oxidation of organic pollutants in water.

The differential charge density distributions of BiO_2-x_/MIL-101(Fe) and B_x_M_y_/O_2_ are shown in Fig. 4g and Fig. S31, highlighting the effect of O_v_. The presence of O_v_ enhances electron transfer from BiO_2-x_ to MIL-101(Fe), increasing the transferred charge from 2.12 to 2.59 e, which agrees with the work function and KPFM analyses (Fig. 2). These calculations also support the O_v_-induced formation of Bi–O = C interfacial bonds and the associated electron redistribution driven by the IEF. For Yeager-type O_2_ adsorption, the differential charge analysis shows that O_v_ promote electron transfer from the Bi/Nd sites to adsorbed O_2_, with the transferred charge increasing from 0.33 to 0.51 e. To further elucidate the electronic interaction of Nd and Bi near O_v_, the density of states and projected density of states were analyzed (Fig. 4h). Unlike transition metals that typically donate *d* electrons to the π* antibonding orbitals of O_2_, Bi possesses filled *d* orbitals, while its 6 *s* and 6*p* states lie close to the Fermi level and dominate the valence band together with O 2*p* states. Upon excitation, electrons are promoted to Bi 6*p* and O 2*p* antibonding states, enabling electron transfer to adsorbed O_2_. After O_2_ adsorption, the PDOS center of the Bi 6*p* orbital shifts from 1.11 to 1.15 eV, and the Nd 4*f*-band center shifts from 0.47 to 1.39 eV (Fig. S32), indicating orbital hybridization between Bi/Nd states and O_2_ orbitals. The interfacial binding between MIL-101(Fe) clusters and the (01$\bar{1}$) surface of Nd-BiO_2-x_ was further examined by DFT-based molecular dynamics (Fig. S33). Compared with BiO_2_ without vacancies, the binding energy increases from 10.28 to 17.87 eV for BiO_2-x_, indicating stronger interfacial interactions. Independent gradient model based on Hirshfeld partitioning (IGMH) shows a δg^inter^ value of 0.01 and 0.04 for the BiO_2_/MIL-101(Fe) and BiO_2-x_/MIL-101(Fe) interface (Fig. 4i and S34), further confirming the enhanced interfacial interaction induced by oxygen vacancies. To further examine the bonding character, electron localization function (ELF) calculations were performed (Figure S35). The ELF map shows enhanced electron localization in the interfacial region of BiO_2-x_/MIL-101(Fe) compared with BiO_2_/MIL-101(Fe), indicating stronger interfacial electronic coupling induced by oxygen vacancies, supporting the formation of Bi–O = C interfacial bonds.

### Evaluate catalytic performance and degradation pathways by the flat-plate reactor

**Fig. 5:**
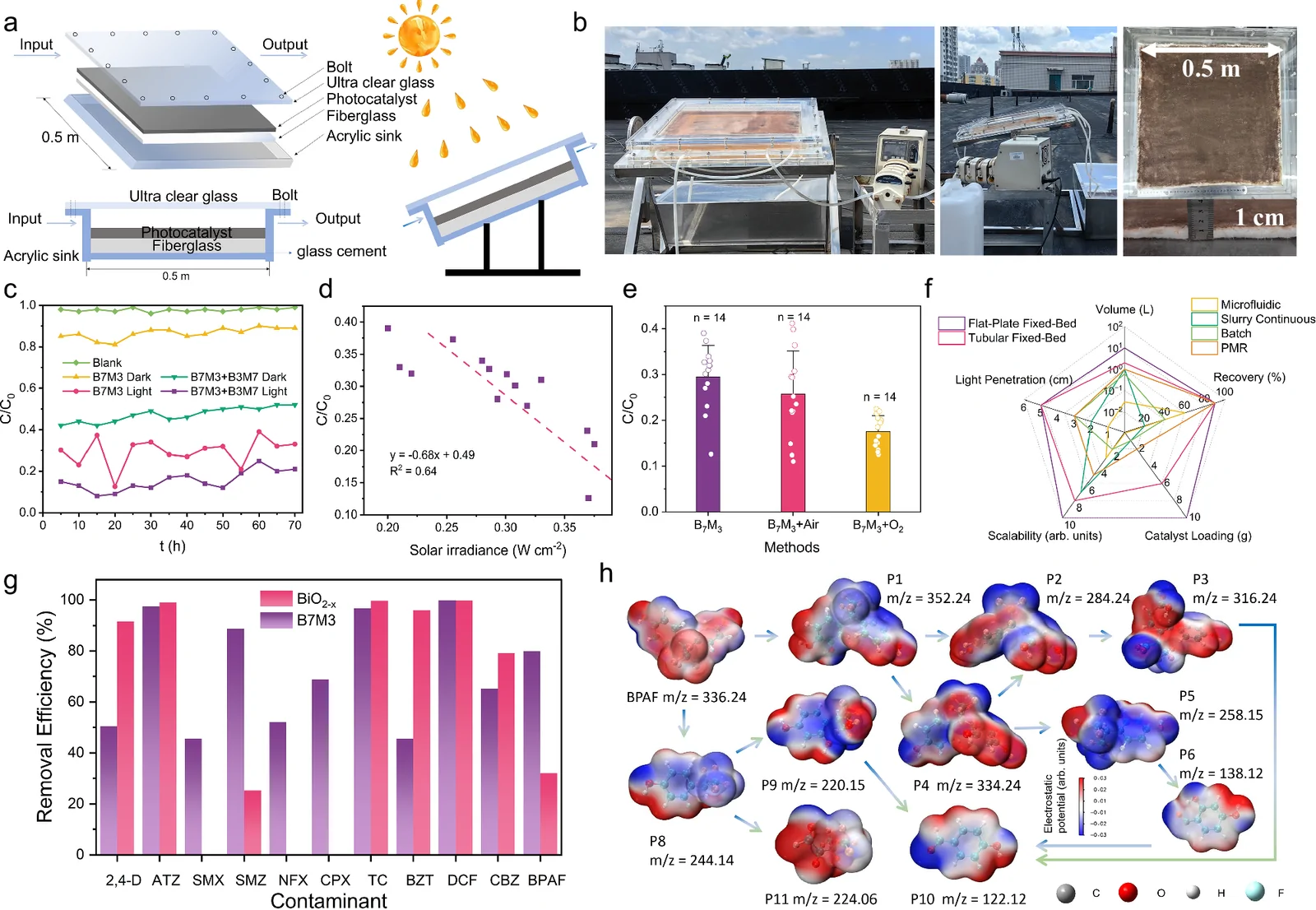
Evaluation of the practical photocatalytic performance of the flat-plate reactor. **a** Schematic diagram of the flat-plate device structure simulation. **b** Enlarged photo of the actual reaction device. **c** Actual catalytic performance of the photocatalyst in the scaled-up continuous flow experiment, reaction conditions: [BPAF] = 3 μM, pH_0_ = 7.8, and *T* = 25 °C ± 3 °C. **d** Relationship between the degradation effect and light intensity by linear fitting. **e** Impact of introducing O_2_ and air on degradation efficiency in the scaled-up continuous flow experiment. The overlaid points represent 14 measurements collected at different time points during the continuous-flow experiment. Data for (**e**) is presented as mean values  ±  SD (*n*  =  14). **f** Radar chart of parameters for different types of photocatalytic reactors for flat-plate fixed-bed, tubular fixed-bed, microfluidic, slurry continuous-flow, batch, and photocatalytic membrane reactors (PMR)^40,60–63,66–70^. The values represent the median values obtained from previously published studies. **g** Removal efficiency of eleven distinct micropollutants in reservoir water, reaction conditions: 2×AM 1.5 G illumination, [Contaminant] = 0.5 μM, pH_0_ = 7.8, and *T* = 25 °C ± 3 °C. The micropollutants names and abbreviations are respectively, 2,4-dichlorophenoxyacetic acid (2,4-D), atrazine (ATZ), sulfamethoxazole (SMX), benzathine (BZT), ciprofloxacin (CPX), tetracycline (TC), norfloxacin (NFX), sulfamethizole (SMZ), diclofenac sodium (DCF), bisphenol AF (BPAF), and carbamazepine (CBZ). **h** Degradation pathway diagram of BPAF under sunlight. Source data are provided as a Source Data file.

Practical deployment is a central consideration in photocatalysis research. A continuous-flow flat-plate reactor was constructed and installed outdoors to evaluate BPAF and mixed micropollutant removal under natural sunlight (Fig. 5a, b). The powdered catalyst was immobilized on glass fiber and packed into an expanded plate reactor with dimensions of 0.5 × 0.05 m. To maximize light transmission while maintaining low cost, the reactor was covered with ultra-clear glass. Two catalyst configurations were used: B_7_M_3_, which relies primarily on photocatalysis, and a composite bed combining photocatalysis and adsorption (B_7_M_3_ + B_3_M_7_), where B_3_M_7_ provides enhanced adsorption capacity (Fig. 5c). This combined design supports continuous reactor operation. Under dark conditions, bare glass fiber shows no adsorption, B_7_M_3_ achieves less than 20% removal, and B_7_M_3_ + B_3_M_7_ reaches about 50% removal due to adsorption. Under sunlight, B_7_M_3_ attains removal efficiencies between 60% and 90%, depending on solar irradiance and ultraviolet intensity, showing a clear positive correlation between light intensity and pollutant removal (Fig. 5d and Fig.e S36).

The bismuth concentration in the reactor effluent remains low, while the Fe^3^⁺ concentration gradually decreases and then stabilizes over time (Fig. S37), indicating acceptable metal stability during continuous operation. Previous experiments under O_2_ and air atmospheres show that oxygen interacts with oxygen vacancies to generate •O_2_⁻ and ^1^O_2_, which enhances electron transfer and hole formation. To further verify the role of oxygen under realistic sunlight conditions, comparative continuous-flow experiments were conducted with O_2_ and Air injection (Fig. 5e). Figure 5f systematically evaluates six distinct photocatalytic reactor systems (flat-plate fixed-bed^60^, Tubular fixed-bed^40^, Microfluidic^61^, Slurry continuous, Batch, and Photocatalytic Membrane Reactor (PMR)) across five critical performance metrics (effective reactor volume (L), catalyst recovery or immobilized catalyst retention (%), total catalyst loading (g), effective light penetration distance (cm), and scalability (arb. units)). Many reactor types are more suitable for precious catalysts in laboratory-scale research and cannot meet the requirements of waterworks processes (Note S3). The flat-plate fixed-bed reactor demonstrates the most balanced and superior performance across almost all metrics^62,63^. It achieves the highest Scalability and the maximum volume ( > 10 L), while maintaining excellent light penetration ( > 5 cm). This suggests that immobilization of the catalyst on a flat surface effectively mitigates the shading effects common in high-loading systems. Similarly, the tubular fixed-bed shows robust performance, though it operates at a slightly lower volume and scalability threshold compared to the flat-plate design.

The applicability of the continuous-flow system was further evaluated using a mixture of eleven micropollutants (Fig. 5g), including pesticides (2,4-dichlorophenoxyacetic acid and atrazine), antibiotics (sulfamethoxazole, benzathine, ciprofloxacin, tetracycline, norfloxacin, and sulfamethizole), and pharmaceuticals (diclofenac sodium and carbamazepine). All compounds show efficient removal under sunlight. The lack of a clear correlation between removal efficiency and half-wave potential (Fig. S38) suggests that degradation is not dominated by free radical reactions in bulk solution. Instead, adsorption is favored by electron-donating groups on aromatic rings, whereas electron-withdrawing substituents weaken π–π electron donor–acceptor interactions and reduce adsorption. Degradation intermediates and possible reaction pathways were identified using HPLC–MS combined with DFT calculations (Fig. 5h, Table S10-11, and Figs. S39, 40). Due to their strong oxidizing power, h^+^ abstract electrons from the benzene ring via single-electron transfer, leading to hydroxylation reactions at the *ortho*- and *para*-positions. Additionally, ^1^O_2_ undergoes addition to the phenol ring to form benzoquinone derivatives, which subsequently undergo ring cleavage. Meanwhile, e^-^ can contribute to the reductive cleavage of a small fraction of C–F bonds, promoting partial defluorination. Toxicity assessments of BPAF and its intermediates (Figs. S41, 42) show that toxicity toward algae, daphnia, and fish decreases as molecular polarity is reduced during degradation.

## Discussion

In summary, we constructed an Nd-BiO_2-x_/MIL-101(Fe) photocatalyst and propose a synergistic O_2_ activation mechanism driven by a direct Z-scheme heterojunction formed through oxygen-vacancy-induced interfacial bonding. O_v_ play a dual role by inducing Bi−O = C chemical bonds that establish a direct Z-scheme heterojunction and by promoting imprint adsorption of O_2_, enabling full-spectrum solar activation. Quenching and competitive kinetic experiments confirm that ^1^O_2_ and photogenerated h^+^ are the dominant reactive species. Incorporation of NIR light increases the apparent degradation rates of BPAF and RhB by factors of 1.9 and 1.2, respectively, with complete RhB removal and 94% BPAF elimination under full-spectrum irradiation. Additive experiments verify the presence of dual metal active sites, with bismuth species remaining dominant. Spectroscopic analyses (FTIR, XPS, and Raman) confirm the formation of Bi–O = C bonds, while KPFM measurements, together with enhanced photocurrent responses and reduced impedance, demonstrate the generation of a built-in electric field and improved charge transport. Finally, scaled-up continuous-flow tests under natural sunlight show that the combined effects of adsorption and photocatalysis enable efficient removal of eleven micropollutants from reservoir water, highlighting the practical potential of this system.

## Methods

### Materials

All chemicals were commercially available for the experiments without further purification and described in Supplementary Methods 1.1.

### Establishing Z-scheme heterojunction BxMy

MIL-101(Fe) was synthesized by a simple hydrothermal process following the published report with slight modifications^47,64^. Normally, H_2_BDC (2.5 mmol) and FeCl_3_·6H_2_O (5 mmol) were mixed in 40 ml of N,N’-dimethylformamide (DMF) and stirred for an hour. Following that, the mixture was heated for 20 h at 110 °C after being placed into a 100 mL Teflon-lined autoclave. After that, the brown-red product was centrifuged to recover it before being washed once with 100% ethanol and three times with ultra-pure water. The obtained materials were then allowed to dry for 12 h in an oven set at 105 °C.

Nd-BiO_2-x_ micro-nano sheet was synthesized via a hydrothermal process following the protocol mentioned in previous work^12,13^. In particular, 60 mL of deionized water was used to dissolve 2.80 g of NaBiO_3_, 0.175 g Nd(NO_3_)_3_·6H_2_O (4%), and 2.4 g of NaOH. The produced suspensions were then further stirred for 30 min. The final step involved transferring the produced aqueous suspensions to a 100 mL Teflon-lined stainless-steel autoclave and heating it for 18 h at 180 °C. The products were then centrifuged, cleaned multiple times with ultra-pure water, and heated at 80 °C for 4 h.

The B_x_M_y_ heterojunctions were fabricated via the ball-milling method following the procedure in Figure S1. Typically, different mass ratios of BiO_2-x_ and MIL-101Fe (BiO_2-x_: MIL-101(Fe)** =** *x*: *y*** =** 1:9, 3:7, 1:1, 7:3, 9:1) in a total amount of 1 g were added into a QM-3SP04 ball miller (Nanjing Keyscience Electronic Technology Company). The ball-milling operation was carried out at a rotational frequency of 50 Hz with *z*** =** 10** −** 60 min, namely B_x_M_y_-z. In particular, the material by ball milling for 30 min is abbreviated as B_x_M_y_. The acquired samples with varying mass ratios were cleaned multiple times with ultra-pure water.

### Characterizations

The crystal structure and morphology of as-prepared samples were measured using X-ray diffraction (XRD, Bruker D8 Advance diffractometer, the Netherlands) with Cu *K*α radiation (γ = 1.5418 Å) in a 2θ range of 5 to 90°. The valence state and chemical 34 composition of elements were analyzed by X-ray photoelectron spectroscopy (XPS, ESCALAB 250Xi, Thermo Fisher Scientific Inc., USA) with an Al *K*α X-ray source, and all the binding energies were calibrated using the C 1 *s* peak at 284.8 eV. The analysis of the electron spin resonance (ESR, A200S-95/12) was conducted with a Bruker EMXplus. The UV/Vis diffuse reflectance spectra were recorded by a spectrophotometer (Shimadzu, UV-2550), in which BaSO_4_ was employed as the background. The Fourier transform infrared spectroscopy (FTIR) was recorded by a spectrometer (PerkinElmer, Frontier).

The Kelvin force microscopy scans (KPFM) were performed on Bruker multimode 8, the Netherlands. Scanning electron microscopy (SEM) and atomic force microscopy (AFM) were performed on Sigma500 (ZEISS Co., Germany) and ICON (Bruker, the Netherlands) instruments. The high-resolution transmission electron microscopy (HR-TEM), high-angle annular dark field scanning transmission electron microscopy (HAADF-STEM) images, and corresponding energy dispersive spectroscopic (EDS) mapping analyses were performed on a Tecnai G2 F30 (Thermo Scientific Inc., USA) instrument. The time–resolved photoluminescence (TRPL) measurements were recorded on a HORIBA FluoroMax-4 detector with the NANOLED at an excitation wavelength of 391 nm.

The characterization methods, including BET, TRPL, electrochemical tests, and in situ FTIR, are described in detail in Supplementary Methods 1.2** −** 1.4.

### Pollutant degradation experiments

The different spectrum photocatalytic activities of B_x_M_y_ were investigated for the removal of Bisphenol AF (BPAF) and Rhodamine B (RhB) in an aqueous solution under xenon lamp (20×AM 1.5 G illumination), visible light (420 nm cutoff filter), and NIR (800 nm cutoff filter). The light intensity for the continuous flow experiments under sunlight is 2-3×AM 1.5 G. The details of degradation experiments, qualitative and quantitative analytical methods, including UPLC, UPLC-MS, TOC, quenching, and coordination experiments, are available in Supplementary Methods 1.5 − 1.6.

In the continuous-flow sunlight setup, the reservoir was made of acrylic, and the angle-adjustable frame was constructed from stainless steel. The top surface was covered with a 3 mm-thick ultra-clear glass with a light transmittance exceeding 90%. 10 g of the catalyst was loaded onto a 1 cm-thick glass fibre mat via the dry impregnation method. The catalyst-loaded mat was then fixed onto the acrylic backplate with silicone sealant.

### Density functional theory

The DFT calculations were performed on CP2K 2025.1^65^. Core electrons were represented by GTH-PBE pseudopotentials, and valence states were expanded in the DZVP-MOLOPT-SR-GTH Gaussian basis. A plane-wave cut-off of 400 Ry and a relative cut-off of 55 Ry were employed. The details as seen in Supplementary Methods 1.7.

### Reporting summary

Further information on research design is available in the Nature Portfolio Reporting Summary linked to this article.

## Supplementary information

Supplementary Information

Description of Additional Supplementary Files

Supplementary Data 1

Reporting Summary

Transparent Peer Review file

## Source data

Source Data

## Data Availability

The data that support the findings of the study are included in the main text and supplementary information files. Source data are provided with this paper.
