# Supplementary material for: Near-infrared-active Nd–BiO2-x/MIL-101(Fe) with vacancy-induced interfaces for solar water purification
Source: Nat Commun. 2026 Aug 26;17:10237. doi: 10.1038/s41467-026-77239-z (PMC13616951; doi:10.1038/s41467-026-77239-z)
Supplement: Supplementary file 1 [file 41467_2026_77239_MOESM1_ESM.pdf]

Supporting Information

Near-Infrared-Active Nd–BiO_{2-x}/MIL-101(Fe) with Vacancy-Induced Interfaces for Solar Water Purification

Shan Liu[1], Zhonglin Chen[1], Jimin Shen[1], Jingkai Lin[2], Jing Kang[1]*, Congshi Zhu[1], Simin Long[1], Yi Zhao[1], Lanbo Bi[1], Huayang Zhang [2*], Sihui Zhan [3,4]*

[1] State Key Laboratory of Urban-rural Water Resources and Environment, School of Environment, Harbin Institute of Technology, Harbin, China

[2] School of Chemical Engineering, Adelaide University, Adelaide, South Australia 5005, Australia.

[3] MOE Key Laboratory of Pollution Processes and Environmental Criteria, College of Environmental Science and Engineering, Nankai University, Tianjin, China

4 School of Environmental Science and Engineering, Tianjin University, Tianjin, China.

*Corresponding authors: jingkanghit@163.com; huayang.zhang@adelaide.edu.au; shzhan@tju.edu.cn

Table of Contents

1. Supplementary Materials and Methods 3

1.1 Chemicals and Reagents 3

1.2. Characterization 3

1.3 Electrochemical Tests 4

1.4 In-situ FTIR studies 5

1.5 Quantification of pollutants 5

1.6 Photocatalytic experimental procedures 6

1.7 Density Functional Theory calculations 8

2 Supplementary Figures 9

3 Supplementary Tables 34

4 Supplementary Notes 40

Supplementary Note 1 | Identification of the Ov and the formation of the Bi−O=C bond.. 40

Supplementary Note 2 | Quantify the contribution ratios of $^1O_2$ and h^+ to the degradation of BPAF.. 41

Supplementary Note 3 | Definitions, calculation procedures, and data sources for the parameters presented in Figure 5f.. 42

Supplementary References.. 44

1. Supplementary Materials and Methods

1.1 Chemicals and Reagents

All chemicals were commercially available for the experiments without further purification. Deionized water (R=18.25 MΩ) was used in all the experiments. Neodymium(III) nitrate hexahydrate ($Nd(NO_3)_3·6H_2O$, 99.0%), N,N-Dimethylformamide (DMF, 99.5%), Rhodamine B ($C_{28}H_{31}ClN_2O_3$, AR), Hexafluorobisphenol A ($C_{15}H_{10}F_6O_2$, 98%), Natural β-carotene ($C_{40}H_{56}$, 96%), tert-Butanol ($C_4H_{10}O$, 99.0%), 2,2,6,6-Tetramethylpiperidine (TEMP, 97%), TEMPO ($C_9H_{18}NO$, 98%), DMPO (97%), Acetylacetone ($C_5H_8O_2$, 99%), Thiourea (CH_4N_2S, 99%) and Potassium thiocyanate (KSCN, 99%) were obtained from Macklin Co., China. Sodium hydroxide (NaOH, 97%) and Nafion117 solution (~5 wt%) were purchased from Kermel Co., China, and Aladdin Chemistry Co., China, respectively. Sodium bismuth dihydrate ($NaBiO_3$(anhy), 90.0%), Iron(III) chloride hexahydrate ($FeCl_3·6H_2O$, 99.0%), and Terephthalic acid (H_2BDC, 99%) were acquired from Energy Chemical Co., China. Superoxide dismutase (SOD, 6000 u mg^{-1}) and Catalase (5000 u mg^{-1}) were bought from Shanghai Yuanye Bio-Technology Co., China.

1.2. Characterization

The time-resolved photoluminescence decay curves were fitted using a double-exponential decay model. Components 1 and 2 represent the short-lived and long-lived decay processes, respectively. The equations (S1-2) for the exponential fitting and the calculation of the average lifetime are given below:

$$I(t) = A + B_1 e^{-\frac{t}{T_1}} + B_2 e^{-\frac{t}{T_2}} \qquad (S1)$$

$$T = \frac{B_1 T_1}{B_1 + B_2} + \frac{B_2 T_2}{B_1 + B_2} \qquad (S2)$$

where $I(t)$ is the photoluminescence intensity at time t, B is the weighting coefficient, and T is the lifetime.

The pore size distributions, surface areas, and total pore volumes of samples were measured by nitrogen physisorption data at -196℃ after outgassing at 150℃ for 12 h and under a residual vacuum of less than 10^{-4} Pa with a Quadrasorb SI analyzer. The specific surface area was calculated using the Brunauer-Emmett-Teller (BET) equation.[1] The obtained XAFS data was processed in Athena (version 0.9.25) for background, pre-edge line and post-edge line calibrations. Then Fourier transformed fitting was carried out in Artemis (version 0.9.25). The k^2 weighting, k-range of 3 - 11$Å^{-1}$ and R range of 1 ~3 Å were used for the fitting. The four parameters, coordination number, bond length, Debye-Waller factor and E_0 shift (CN, R, σ^2, ΔE_0) were fitted without anyone was fixed, constrained, or correlated. For Wavelet Transform analysis, the χ(k) exported from Athena was imported into the Hama Fortran code. The parameters were listed as follow: R range, 0 − 6 Å, k range, 0 − 15 $Å^{-1}$; k weight, 2; and Morlet function with κ=10, σ=1 was used as the mother wavelet to provide the overall distribution.

1.3 Electrochemical Tests

Preparation of a catalyst-coated glassy carbon electrode

Catalyst ink was prepared by adding 5 mg of catalyst into a mixed solution containing 0.02 mL of Nafion perfluorinated resin solution and 0.1 mL of ethanol. The mixture was sonicated for 30 min to evenly disperse the catalyst, and then 10 μL of the mixture was dripped onto the surface of a glassy carbon electrode (GCE, Φ3 mm) or a F-doped Tin Oxide substrate (FTO, 1.0 × 1.0 cm^2) and dried at 60 ºC.

Chronoamperometry tests

Photoelectrochemical measurements were recorded using an electrochemical workstation (CHI660E, Chenhua). Tests were conducted upon the irradiation of a 300 W Xe lamp.

Amperometric i-t curves were conducted in 0.2 M Na_2SO_4 solution at pH = 6.7 with a three-electrode cell configuration including a working electrode (FTO), a counter electrode (platinum electrode, 10 × 10 × 0.1 mm), and a reference electrode (Ag/AgCl

electrode). The working electrode was biased to the applied open circuit voltage of the corresponding system (vs Ag/AgCl). BPAF was added to the solution to monitor the changed current.

Open circuit potential measurements

Using an Ag/AgCl electrode as a reference electrode, the open-circuit potential of the catalyst-FTO was detected by chronopotentiometry. All potential values were normalized to a reversible hydrogen electrode (RHE). The sampling time was set to 340 s, the light was turned on at 40 s, and BPAF was added at 200 s.

Electrochemical impedance spectroscopy (EIS) measurements

The EIS measurements were carried out using a three-electrode system at a potential bias applied to 10 mV (V_{RHE}) and a scanning frequency in the range of 0.1~100 kHz. The working electrode used was the GCE.

1.4 In-situ FTIR studies

In-situ MIR-IR spectra of the BxMy samples were obtained by using the FTIR spectrometer (Bruker INVENIO S) with the attenuated total reflection (ATR) method, immersed at the bottom of a 30 mL liquid phase cell by a mercury cadmium telluride detector. All of the FTIR spectra were recorded at a resolution of 4 cm^{-1} in the range of 600 − 4000 cm^{-1}. In a typical test, the 25 mL DI water and 5 mg as-prepared samples were placed in the liquid sample holder and ATR, and nitrogen was then continuously injected into the cell. After about 10 minutes in the dark, the Infrared subtracted the blank background. Then, the Xenon lamp was turned on, and the photocatalytic reaction began with oxygen or nitrogen continuously injected. In addition, the measurement of the photocatalytic activity of the samples was performed under visible light illumination ($\lambda > 400$ nm), which was radiated by the Xenon lamp (CEL-HXF300) with a 400 nm UV-cutoff filter.

1.5 Quantification of pollutants

BPAF was quantified using ultra-high performance liquid chromatography

(UHPLC, LC 300, PerkinElmer, Inc. USA) with a C18 column (PE, Quasar C18 100×2.1 mm 3μm, UK). A methyl alcohol/water (70: 30, v: v) mixture was used as the mobile phase, and the detection wavelength was 225 nm. The elution process was conducted at a flow rate of 0.1 mL min^{-1}.

The concentration of Rhodamine B was determined using a UV-Vis spectrophotometer. The absorbance measurements were recorded at the maximum absorption wavelength of 554 nm using a standard 1 cm quartz cuvette.

Eleven trace emerging contaminants were analyzed using liquid chromatography coupled with triple quadrupole tandem mass spectrometry (LC-MS/MS). The mobile phase consisted of methanol and 0.2% formic acid aqueous solution (90:10, v/v), and the flow rate was 0.2 mL/min. The detailed detection parameters, including fragment ions and m/z ratios, are provided in **Supplementary Table 11**.

The photodegradation intermediates of BPAF were determined by a UHPLC system equipped with a time-of-flight (TOF) mass spectrometer (Agilent 6545). The electrospray interface (ESI) ion source was operated in negative ion mode, and the scan acquisition of MS measurements was m/z 50 − 450. The mobile phase comprised methanol (A) and 0.2% formic acid aqueous solution (B) at a flow rate of 0.2 mL/min. The gradient elution program was set as follows: 0 – 1 min, A/B at 5/95; 1 – 15 min, the A/B ratio transitioned linearly from 5/95 to 95/5 and was maintained at this ratio until 24 min; 24 – 26 min, the A/B ratio was rapidly switched back to 5/95 and held at this proportion until 30 min to equilibrate the column for the next injection.

1.6 Photocatalytic experimental procedures

The photodegradation experiments were carried out under the irradiation of a 300 W xenon lamp with the cutoff filters (> 420 nm and > 800 nm). Generally, the photodegradation process was carried out as follows: Firstly, the 20 mg photocatalysts were dispersed in 100 mL BPAF solution with a concentration of 20 mg/L, and after pH adjustment, the mixed solution was stirred for 30 min to achieve adsorption-desorption equilibrium in the dark. Subsequently, the samples were collected from the

reaction solution every 10 minutes under xenon lamp irradiation. Finally, the concentration of BPAF in the samples was determined by high-performance liquid chromatography (HPLC). Quantitative analysis of RhB was conducted via spectrophotometry using a 1 cm path length cuvette with an absorption wavelength set at 554 nm. The desired pH of solutions was achieved by adding 10 mM NaOH or 10 mM HCL and measured using a pH meter. In addition, error bars indicate the reproducibility of data from repeated two independent experiments. The interference of O_2, N_2, coordination, and chemical quenchers can be controlled by continuous gas purging within the original reaction system or initial incorporation of quenchers or coordination.

To study the radicals generated in the photocatalytic reaction process, ESR spin-trapping experiments were carried out. In general, 5 mg photocatalysts were dispersed into 0.3 mL ultrapure water, and then 50 μL DMPO, TEMPO, or TEMP was added to the mixed solution. Subsequently, the reaction solution was placed under xenon lamp irradiation or dark for 1 min, and then the samples were measured for DMPO-·OH and TEMP-$^1O_2$ signals by electron paramagnetic resonance spectrometer. The DMPO-·O_2^- signals were also tested in the same way as above, except for replacing 0.3 mL ultrapure water with 0.1 mL methanol.

The exposure of photogenerated holes was determined using the potassium iodide (KI) oxidation method. A standard curve was first established by measuring the absorbance of triiodide solutions with concentrations ranging from 5 to 100 μM. For the photocatalytic measurement, 30 mg of the catalyst was dispersed in 100 mL of a 10 mM KI aqueous solution. The suspension was irradiated under visible or NIR light. During the 30 min reaction, 3 mL aliquots were collected at selected time intervals and filtered to remove the catalyst

Singlet oxygen was quantified using 9,10-anthracenediyl-bis(methylene)dimalonic acid (ABDA) as the scavenger. A standard curve was prepared using varying concentrations of ABDA diluted in a 0.05 M phosphate-buffered saline (PBS, pH = 7.5). In the typical experimental procedure, 30 mg of the catalyst was added to a mixed

solution comprising 10 mL of 0.5 mM ABDA and 90 mL of deionized water. The suspension was stirred in the dark for 10 min to adsorption equilibrium and then irradiated under visible or NIR light for 30 minutes.

Superoxide radicals were quantified utilizing the nitroblue tetrazolium (NBT) reduction assay. To prepare the standard curve, varying volumes of xanthine oxidase were added to a solution containing NBT, xanthine, and PBS. After reacting at 25 °C for 10 minutes, the reaction was quenched with superoxide dismutase (SOD), followed by extraction with dimethyl sulfoxide (DMSO). For the photocatalytic measurement, 30 mg of the catalyst was added to a solution containing 20 mL of NBT and 80 mL of deionized water. The mixture was stirred in the dark for 5 min and then irradiated under visible or NIR light. After irradiation, the light source was turned off, and 0.5 mL of SOD was immediately added to terminate the reaction. DMSO was then added for extraction, and the absorbance of the final solution was recorded using a UV–Vis spectrophotometer in the wavelength range of 500 – 700 nm.

1.7 Density Functional Theory calculations

All periodic DFT calculations were carried out with **CP2K 2025.1**[2]. Exchange–correlation effects were treated using the **PBE functional** supplemented with **Grimme's D3(BJ) dispersion correction**[3,4]. Core electrons were represented by **GTH-PBE** pseudopotentials, and valence states were expanded in the **DZVP-MOLOPT-SR-GTH** Gaussian basis. A plane-wave cut-off of 400 Ry and a relative cut-off of 55 Ry were employed.

Geometry optimisation

Atomic coordinates were relaxed with the BFGS algorithm until the following thresholds were met: max $|F| \leq 4.5 \times 10^{-4}$ Ha Bohr^{-1}, max $|\Delta R| \leq 3.0 \times 10^{-3}$ Bohr, $\Delta E \leq 1.0 \times 10^{-6}$ Ha.

Adsorption energy

$$E_{ads}=E_{slab+mol}-E_{slab}-E_{mol}$$

Where each term is the fully relaxed total energy obtained under identical settings.

Harmonic vibrational analyses were performed for all adsorbed intermediates, and zero-point energies were included in the final adsorption values.

Electronic analysis

Charge-density differences, density of states, and related wave-function analyses were carried out with **Multiwfn 3.8(dev)**[5–7] and visualised using **VESTA 3.9.0.5a**.[8]

Work function

Calculating with dipole correction.

2 Supplementary Figures

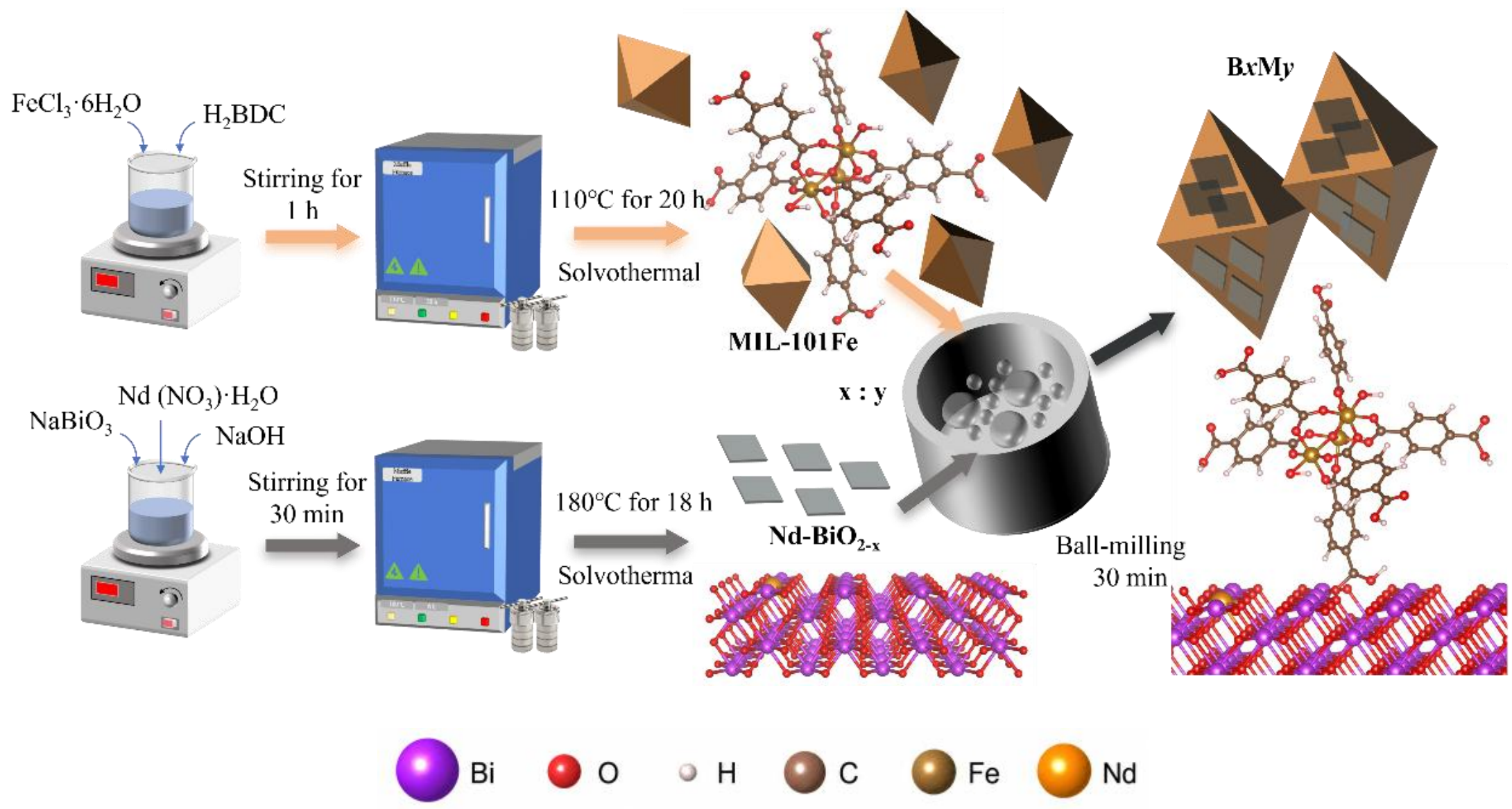

Supplementary Fig.1 | Schematic illustration of the preparation procedures of the BxMy heterojunctions.

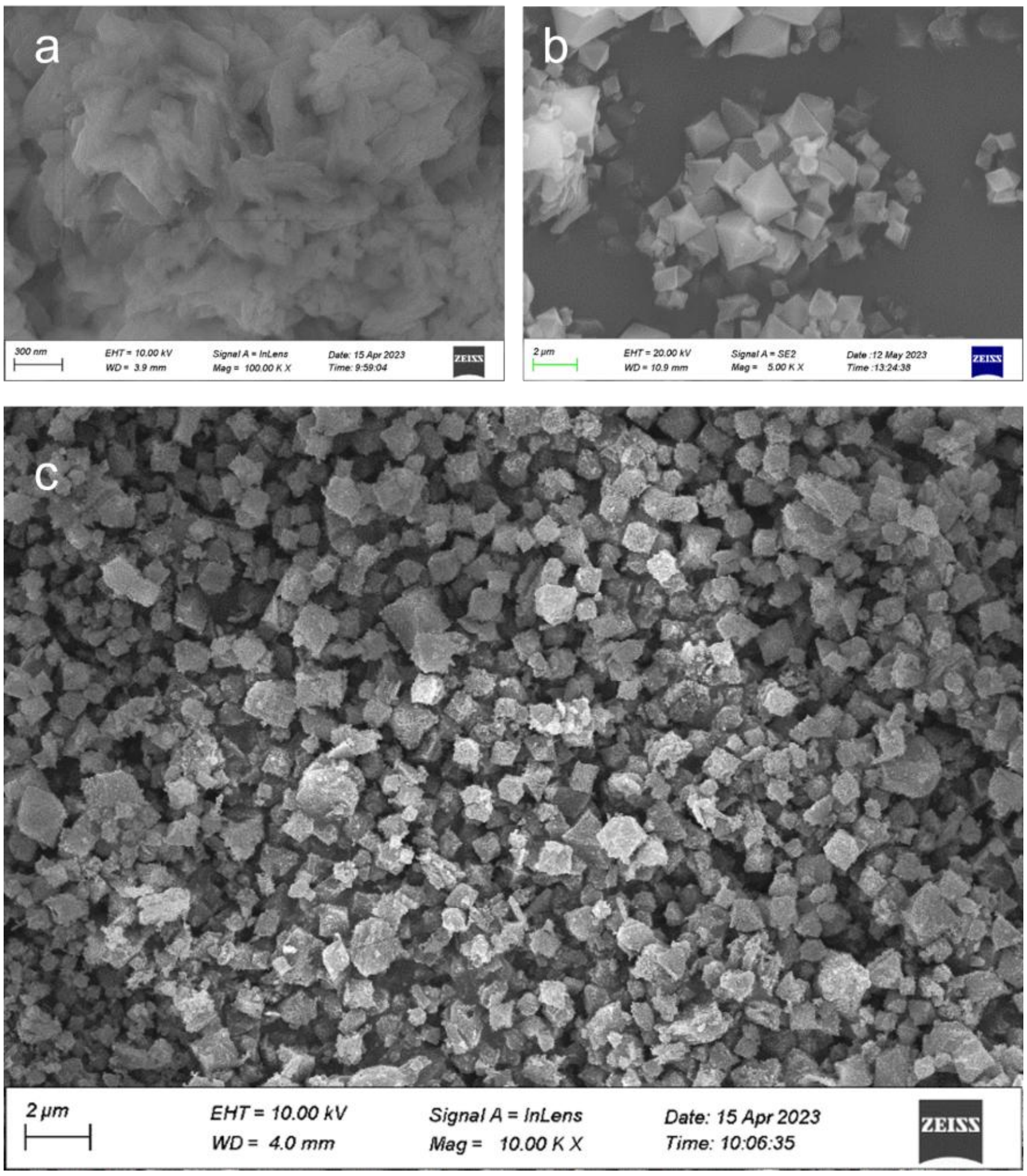

Supplementary Fig.2 | SEM image of the BiO_{2-x} and MIL-101(Fe). (a) The stacking of sheet-like BiO_{2-x} results in an appearance resembling a cluster of flowers. **(b)** The synthesized MIL-101(Fe) particles are all regular hexahedra, with sizes ranging from tens of nanometers to several micrometers. (c) SEM image of the B_xM_y. Ultrathin nanosheets uniformly dispersed on MIL-101(Fe) suppress stacking and improve oxidation efficiency.

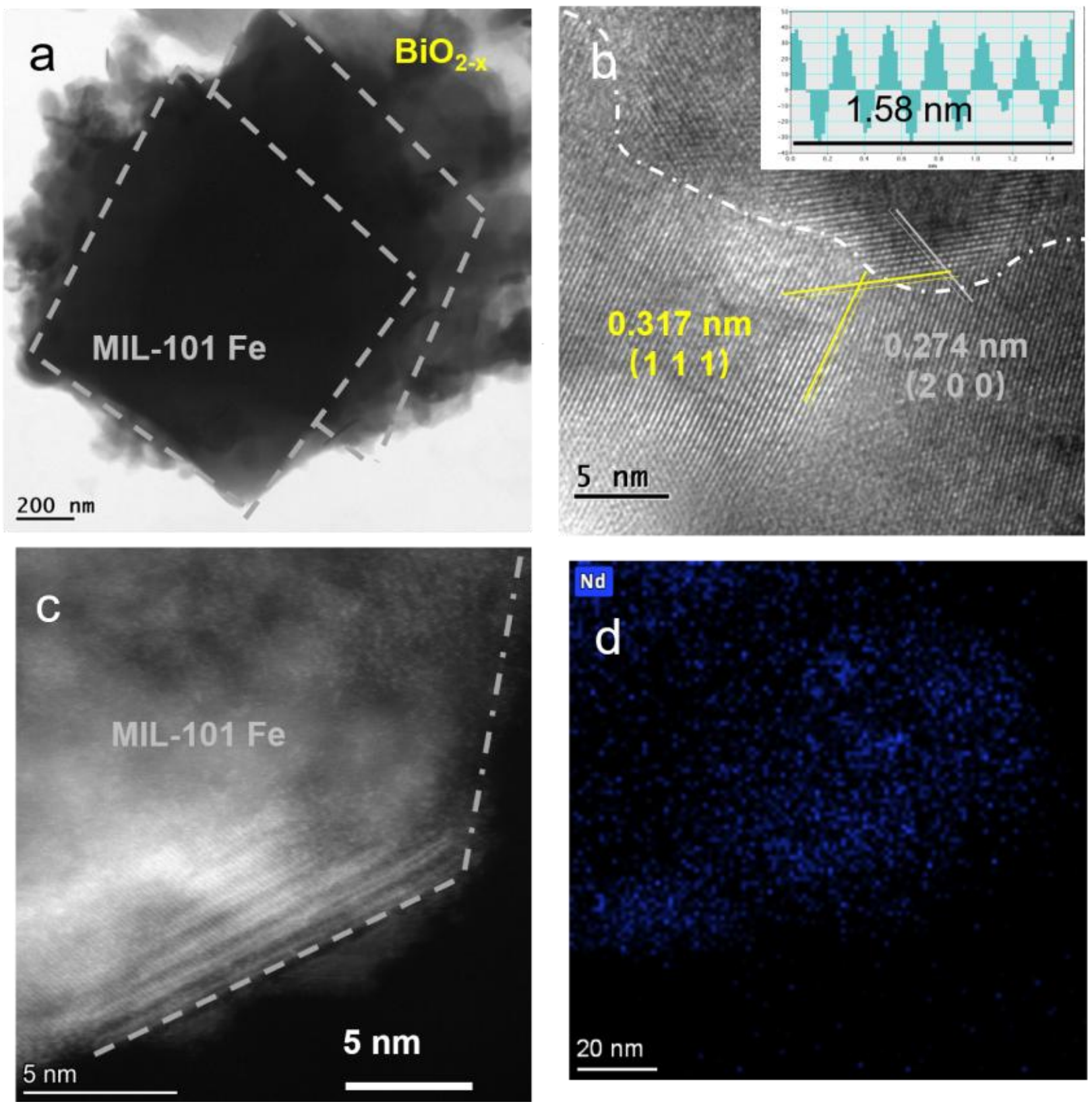

Supplementary Fig.3 | HRTEM and STEM images of the B_xM_y. (a) BF-TEM, (b) HRTEM, (c) aberration-corrected-TEM images, and (d) STEM mapping of Nd.

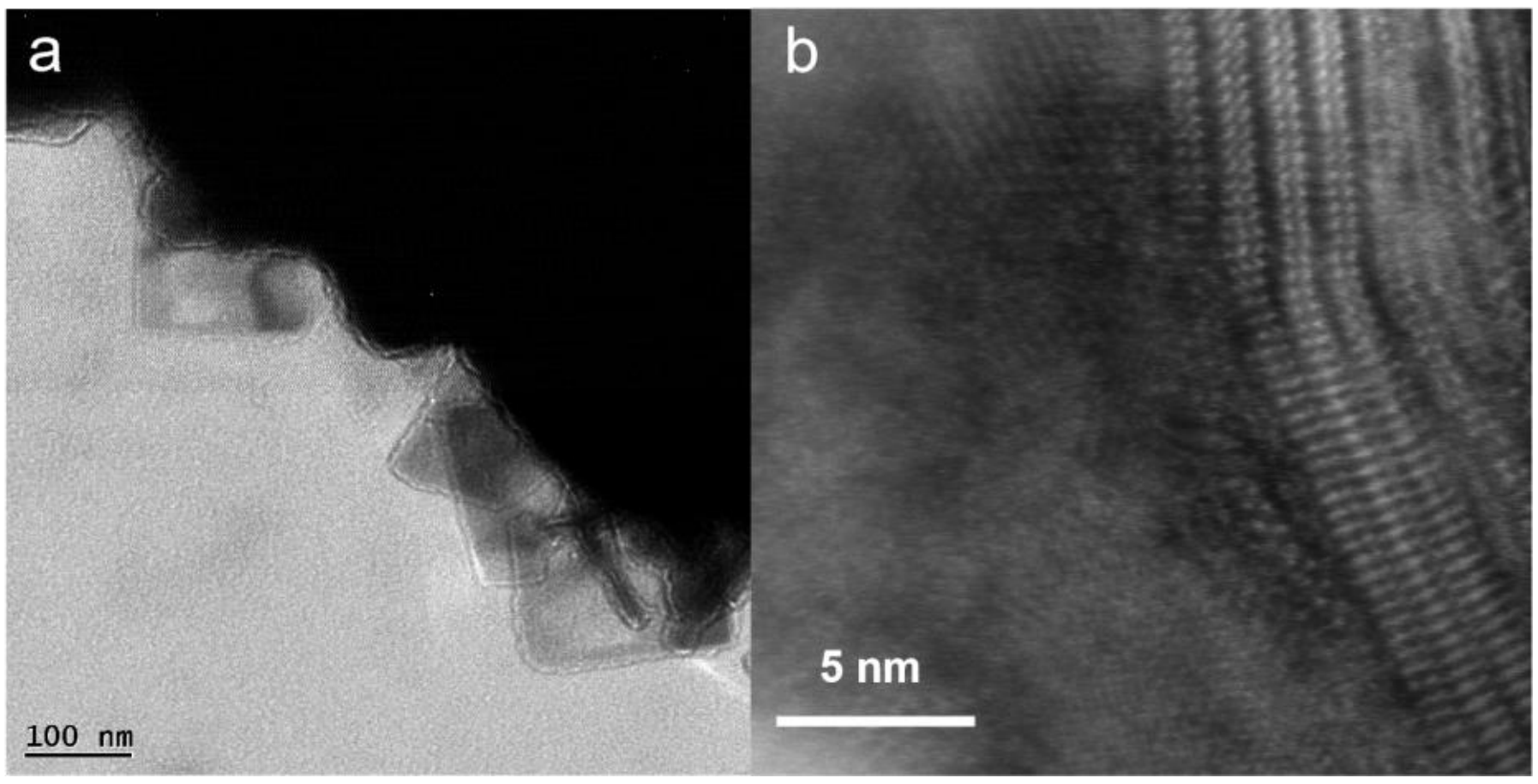

Supplementary Fig.4 | Unedited HRTEM and Aberration-Corrected-TEM images corresponding to the annotated images in Figure 1b and c.

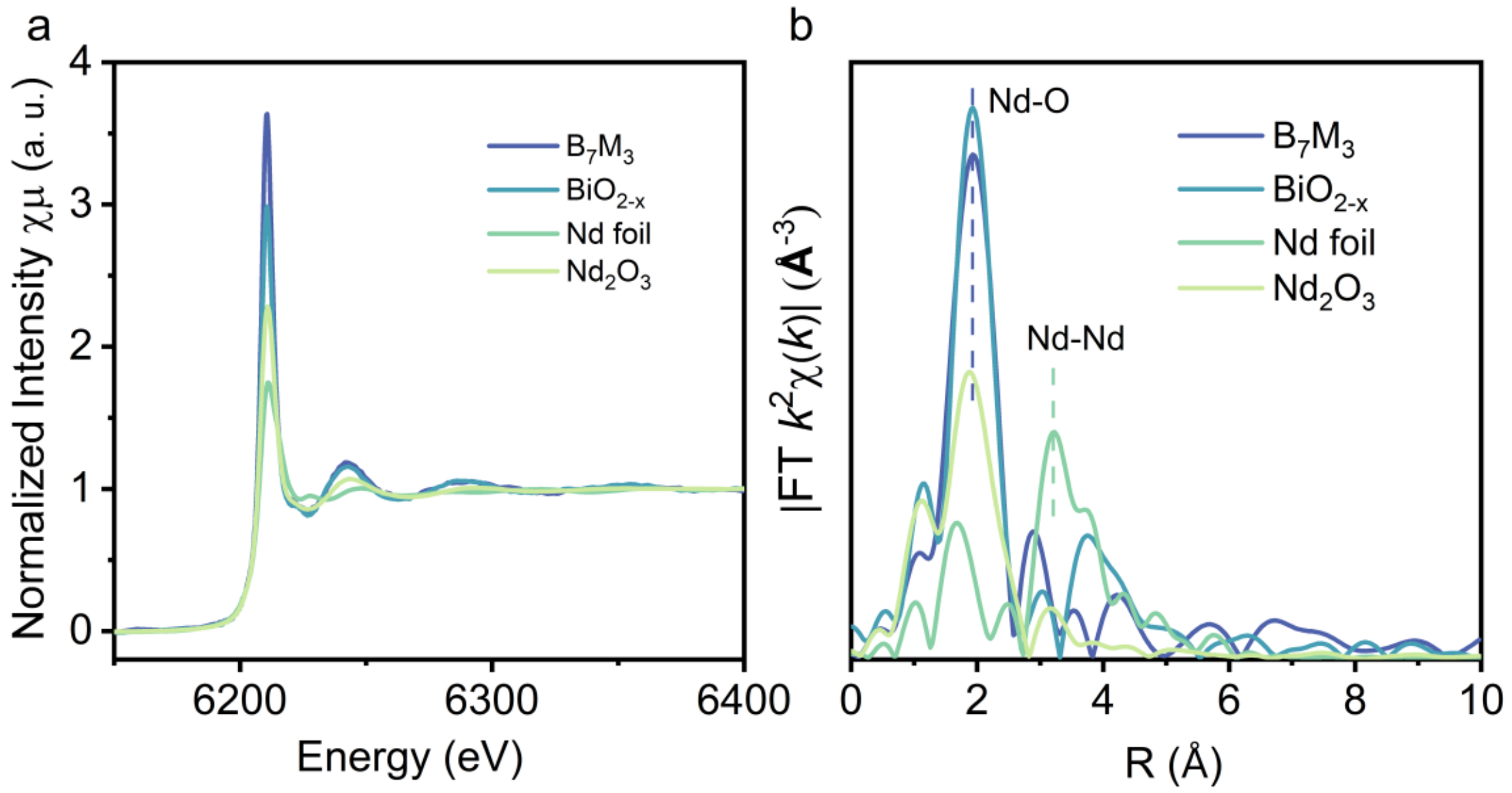

Supplementary Fig.5 | XAS characterization of the Nd element. **(a)** Nd L_3-edge normalized intensity in XANES spectra, and **(b)** k^2-weighted FT-EXAFS spectra of the as-prepared samples.

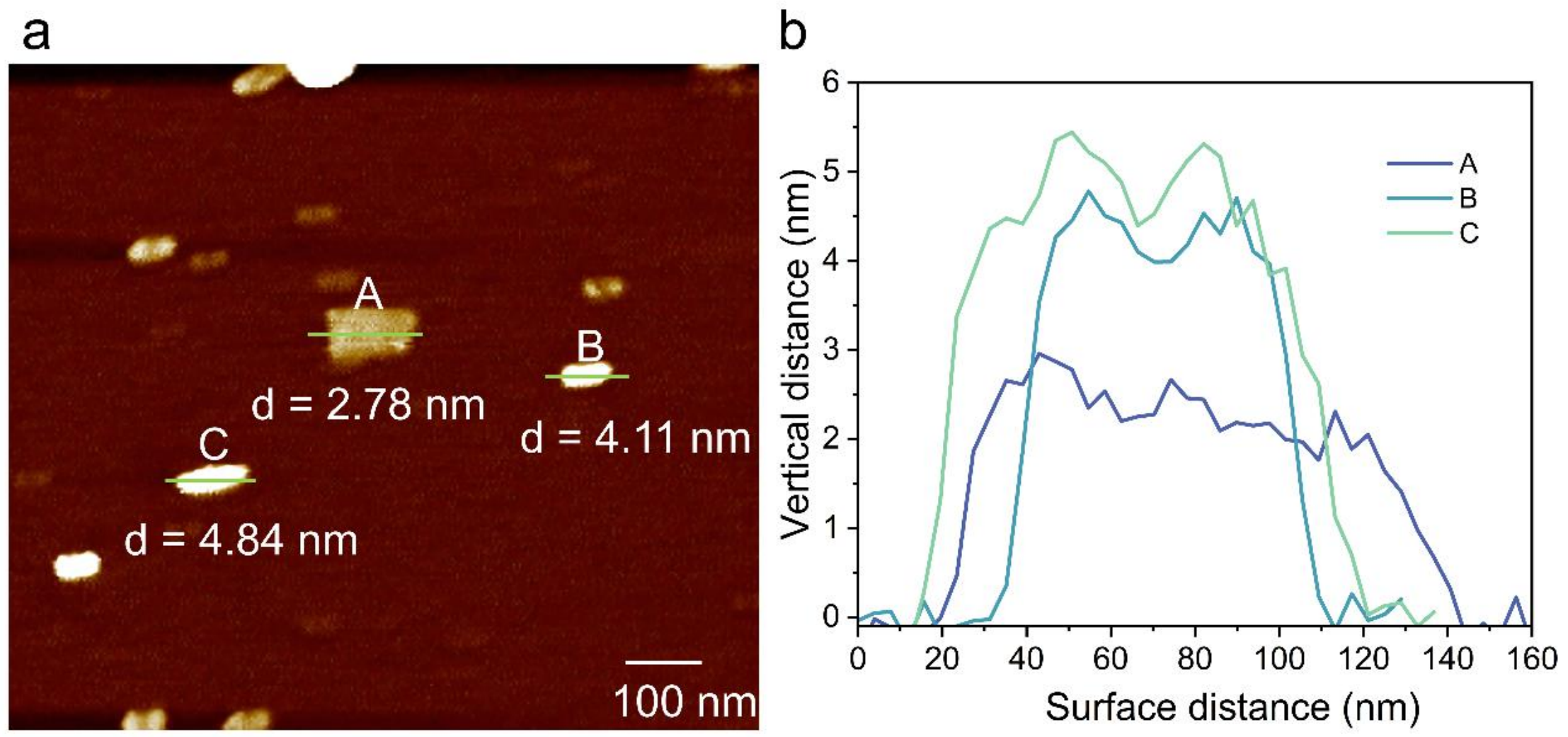

Supplementary Fig.6 | AFM image and the thickness of the BiO_{2-x} samples. (a-b) The three curves of the (**a**) vertical and (**b**) surface distance correspond to the three nanosheets.

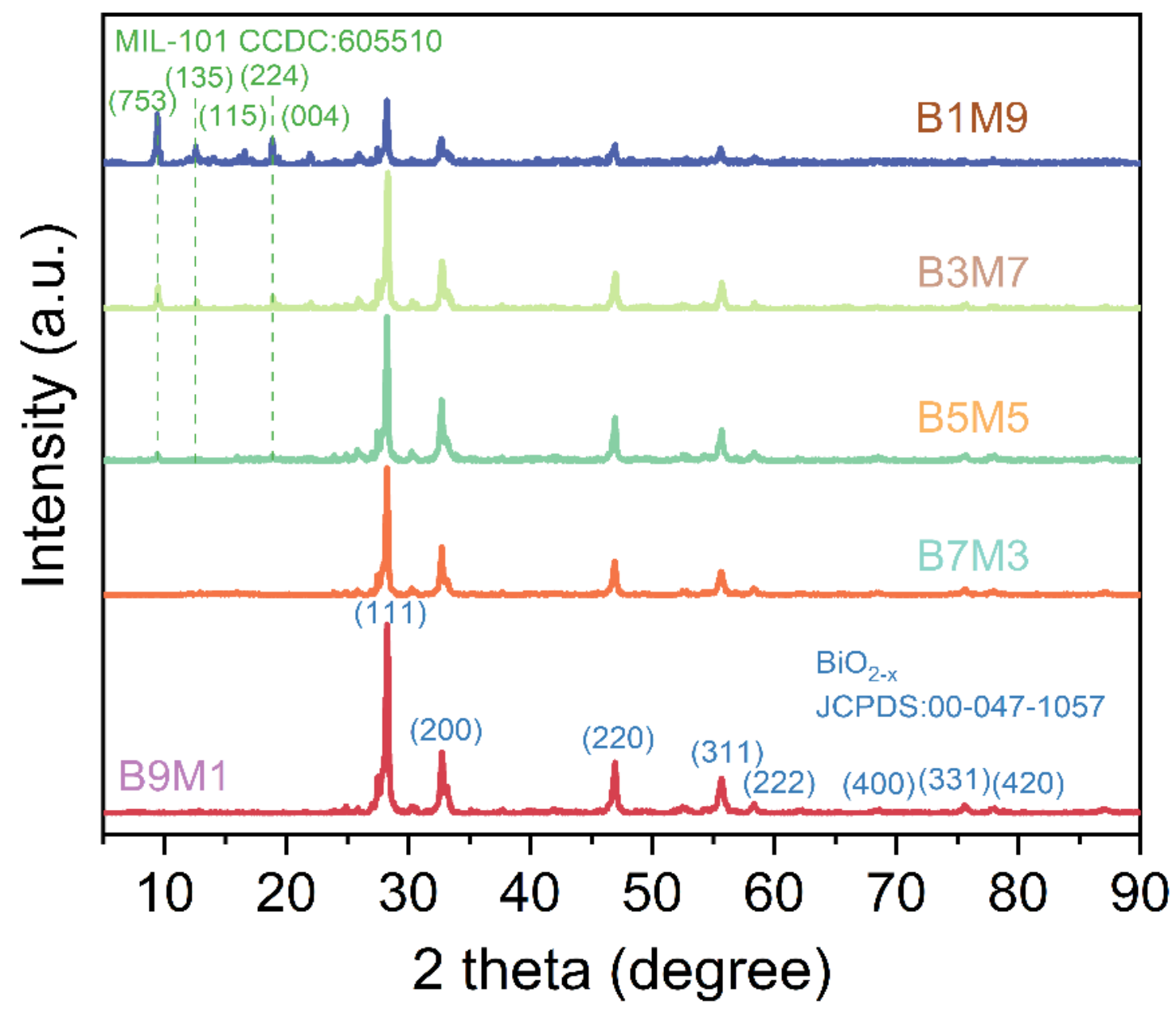

Supplementary Fig.7 | XRD patterns of the B_xM_y samples.

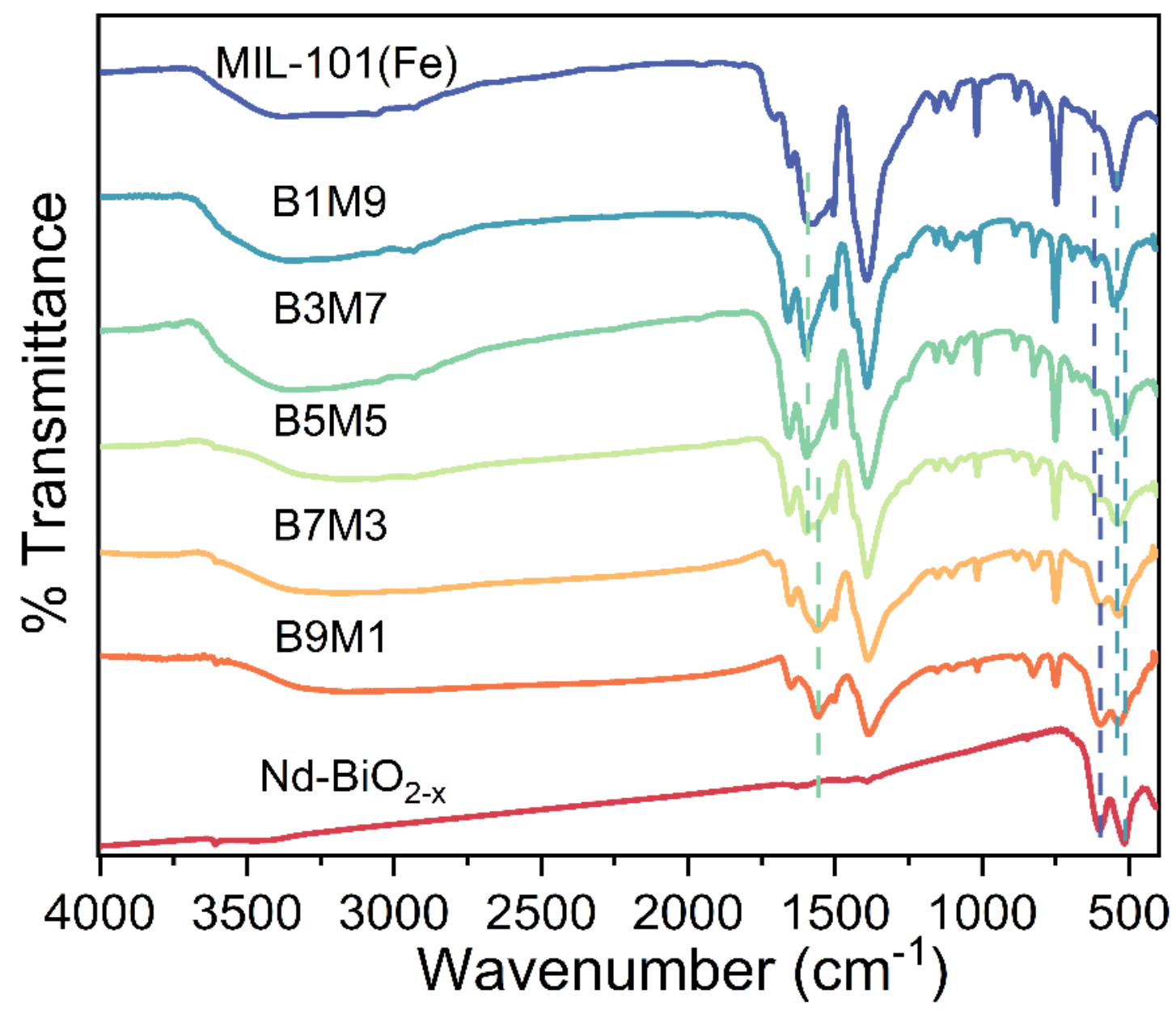

Supplementary Fig.8 | FTIR patterns of the BxMy samples (500 – 4000 cm^{-1}).

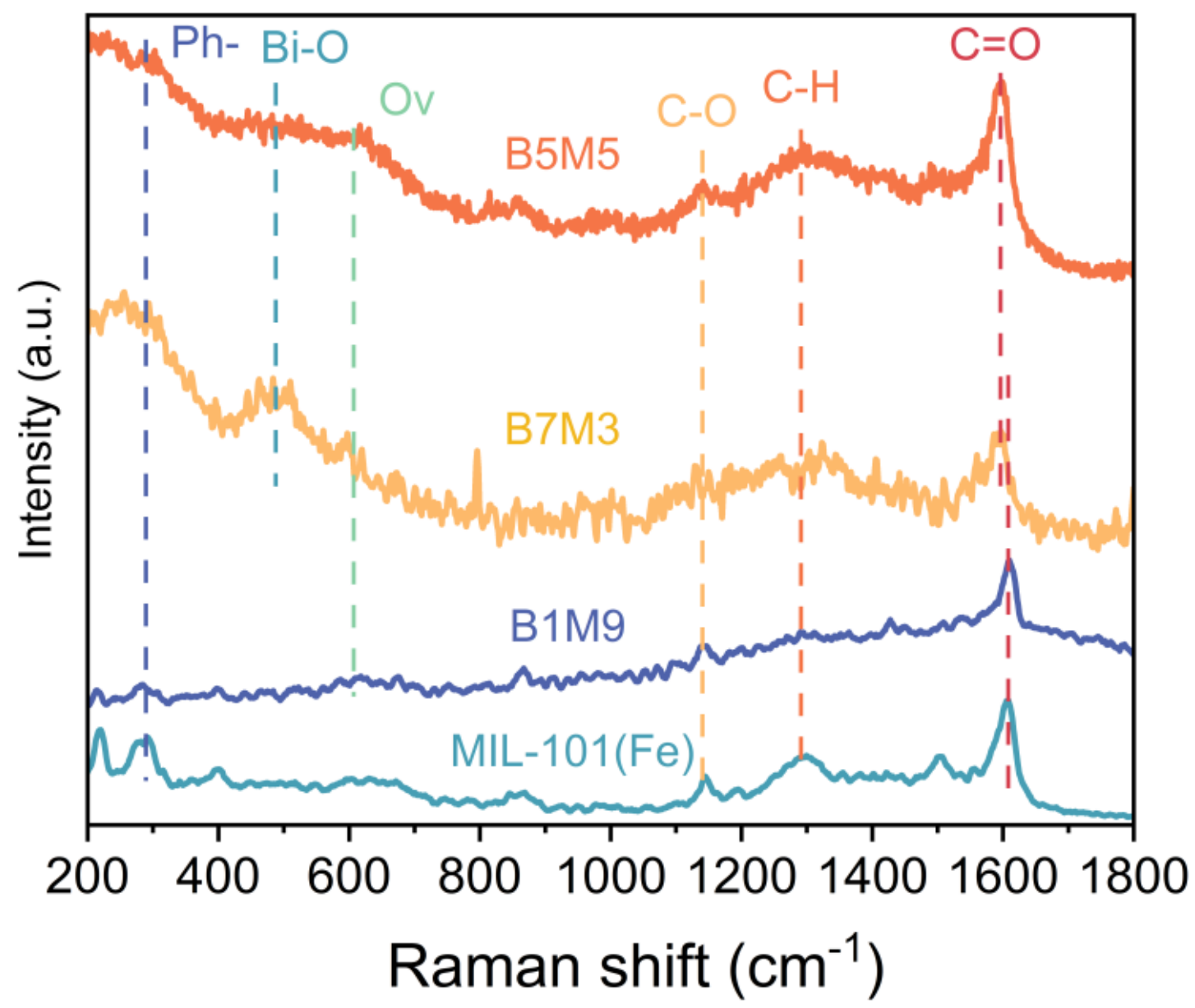

Supplementary Fig.9 | Raman spectra of Nd-BiO_{2-x}, MIL-101(Fe), and BxMy heterojunctions.

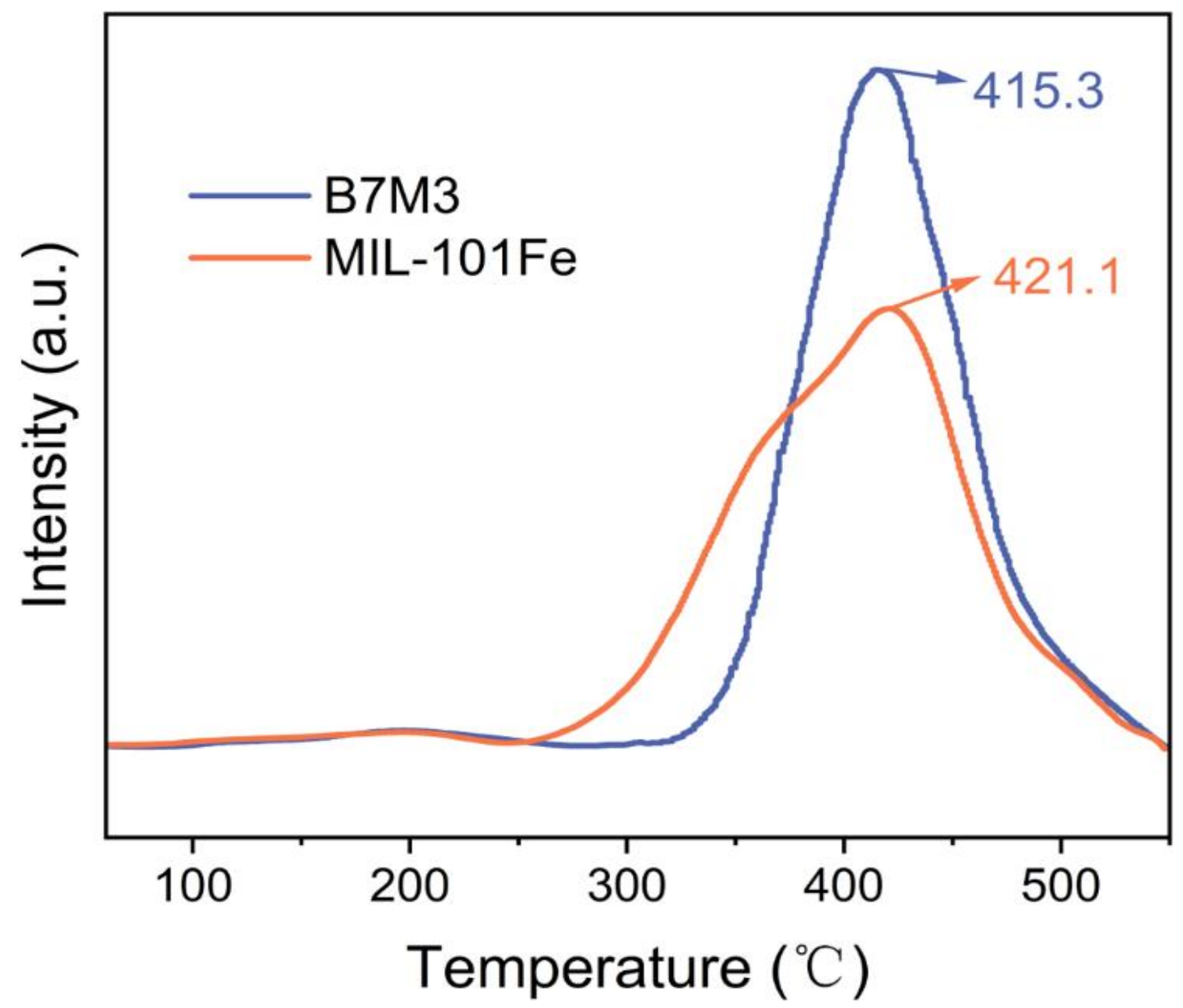

Supplementary Fig.10 | O_2-TPD of MIL-101(Fe) and B_7M_3 heterojunctions.

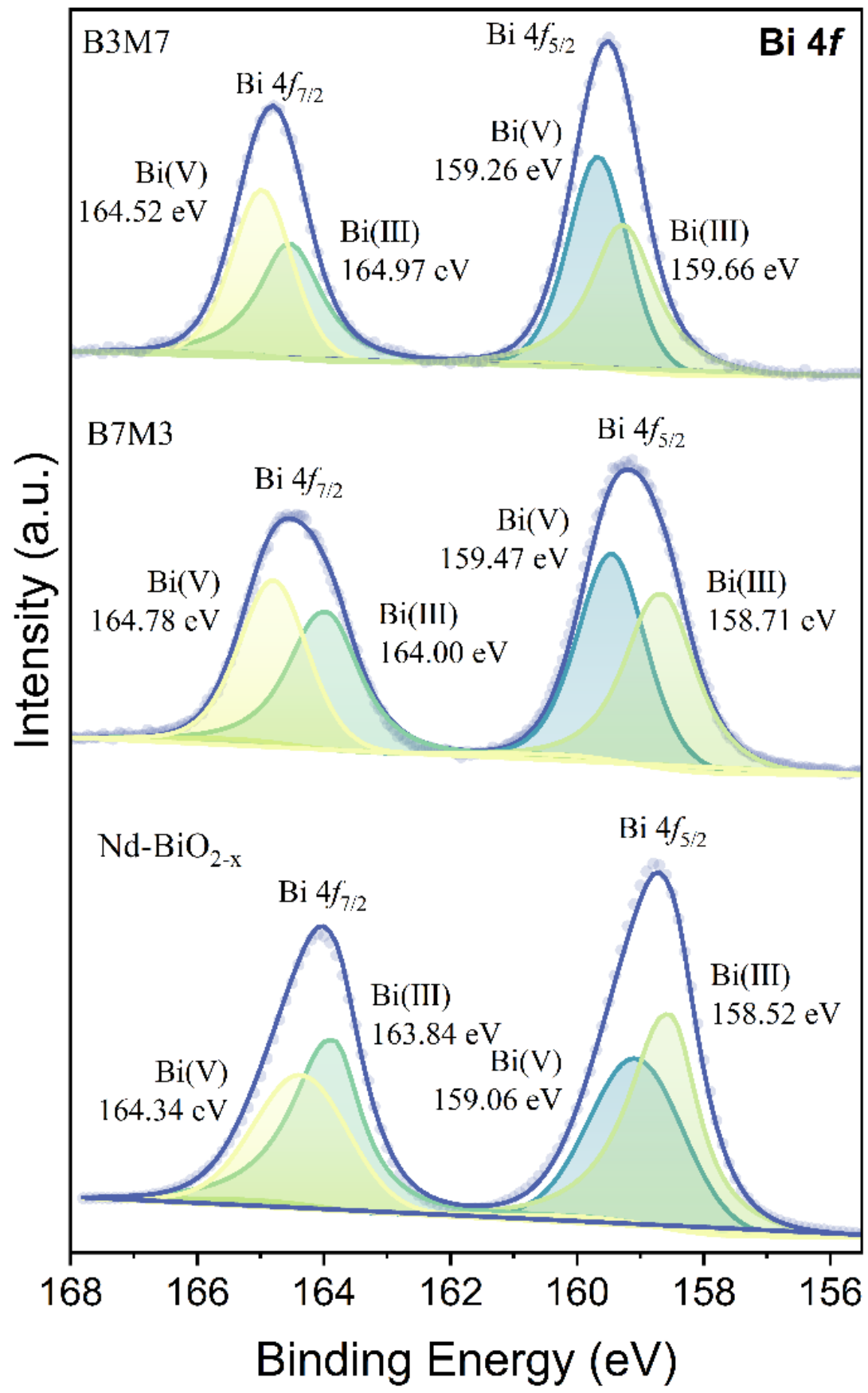

Supplementary Fig.11 | XPS spectra of Bi 4*f* of the as-prepared samples.

B_7M_3 presented the two peaks of the Bi 4*f* spectrum (**Supplementary Fig. 11**) similar to Nd-BiO_{2-x}, while the peaks showed positive shifts of ΔE = 0.43 and 0.37 eV, respectively. B_1M_9 showed stronger positive shifts of ΔE = 0.70 and 0.73 eV. It is demonstrated that the binding energy of Bi 4*f* electrons increased with the MIL-101(Fe) content, indicating a continuous transfer of electrons from Bi to MIL-101(Fe) by ball milling.

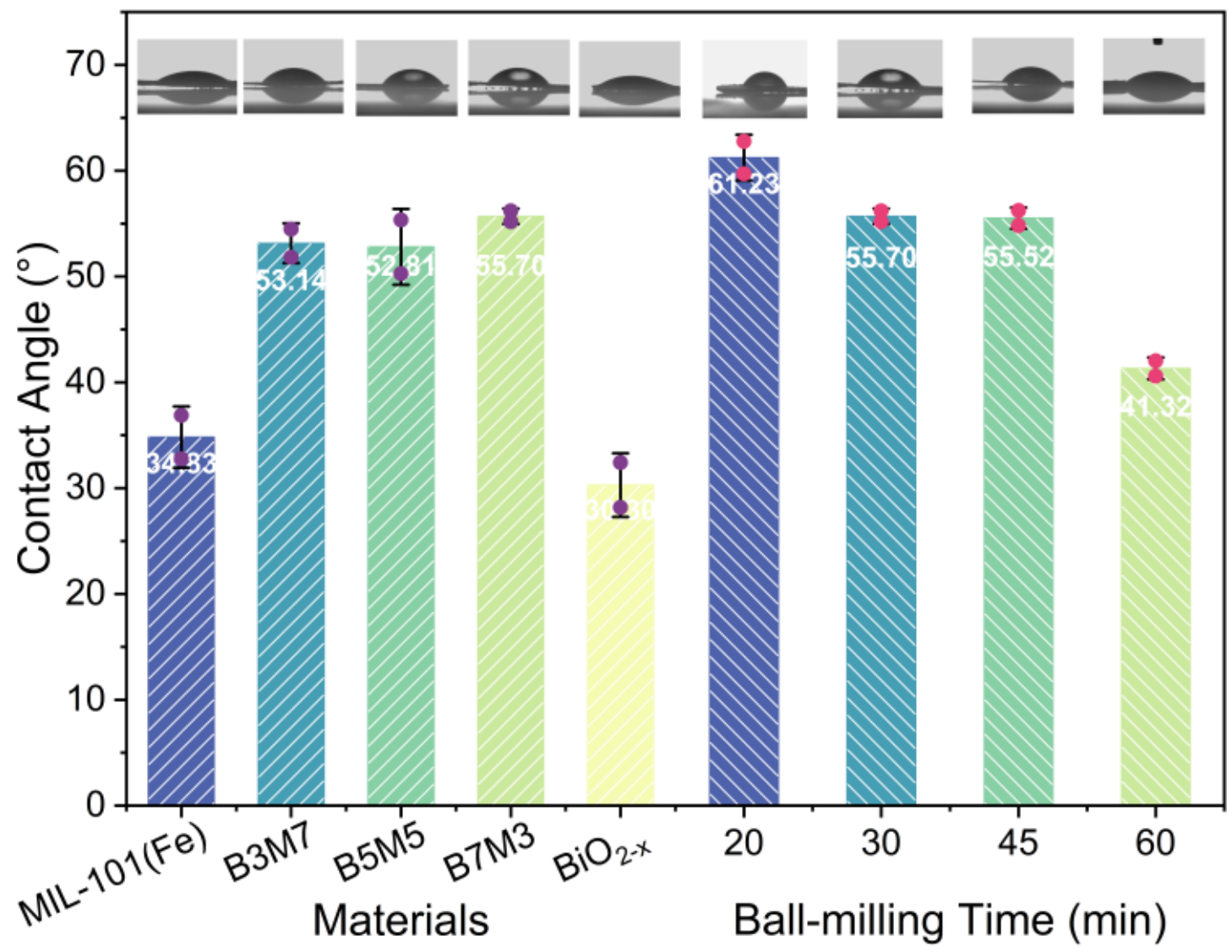

Supplementary Fig.12 | Contact Angle measurement by sessile drop of Nd-BiO_{2-x}, MIL-101(Fe), and B_xM_y heterojunctions. The error bars represent the standard deviations, obtained by repeating the experiment twice.

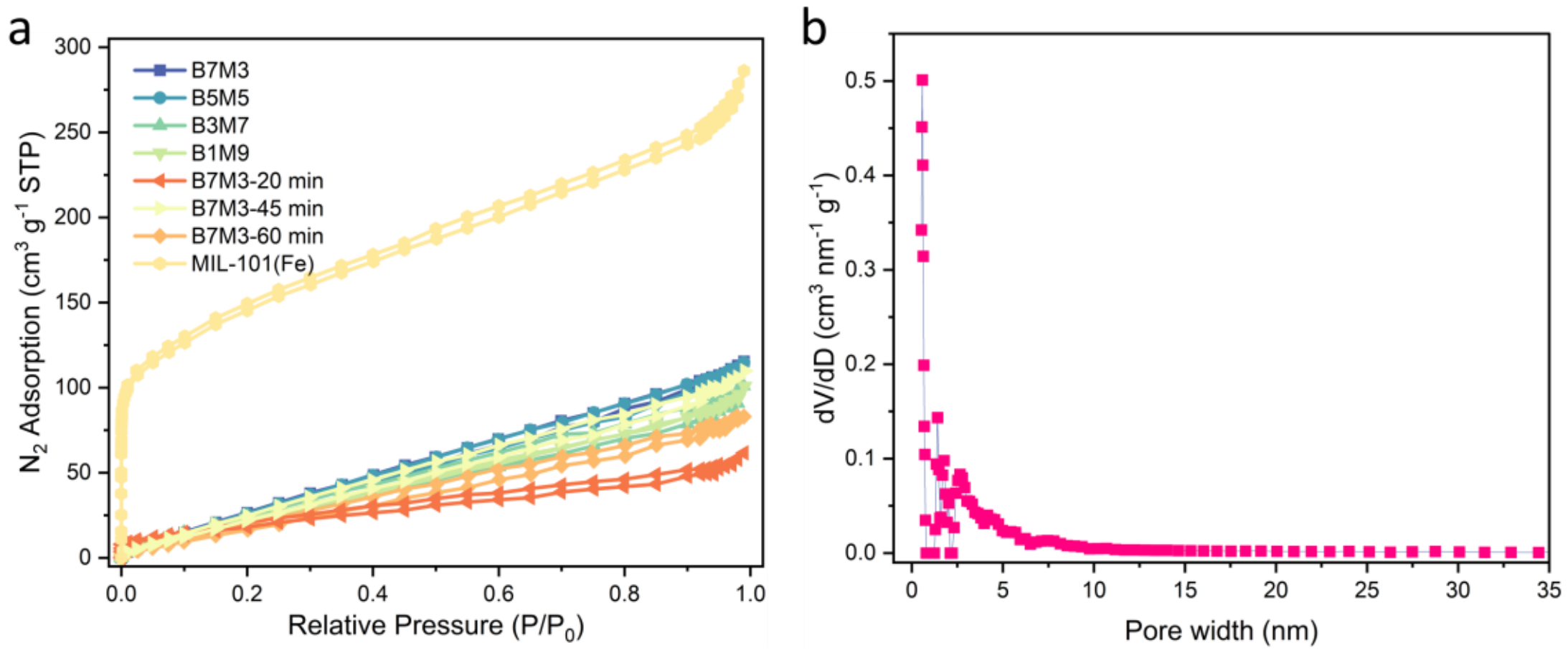

Supplementary Fig.13 | (a) N_2 adsorption-desorption isotherms of Nd-BiO_{2-x}, MIL-101(Fe), and B_xM_y heterojunctions and (b) the pore-size distribution of MIL-101(Fe) using a density functional theory-based pore mode.

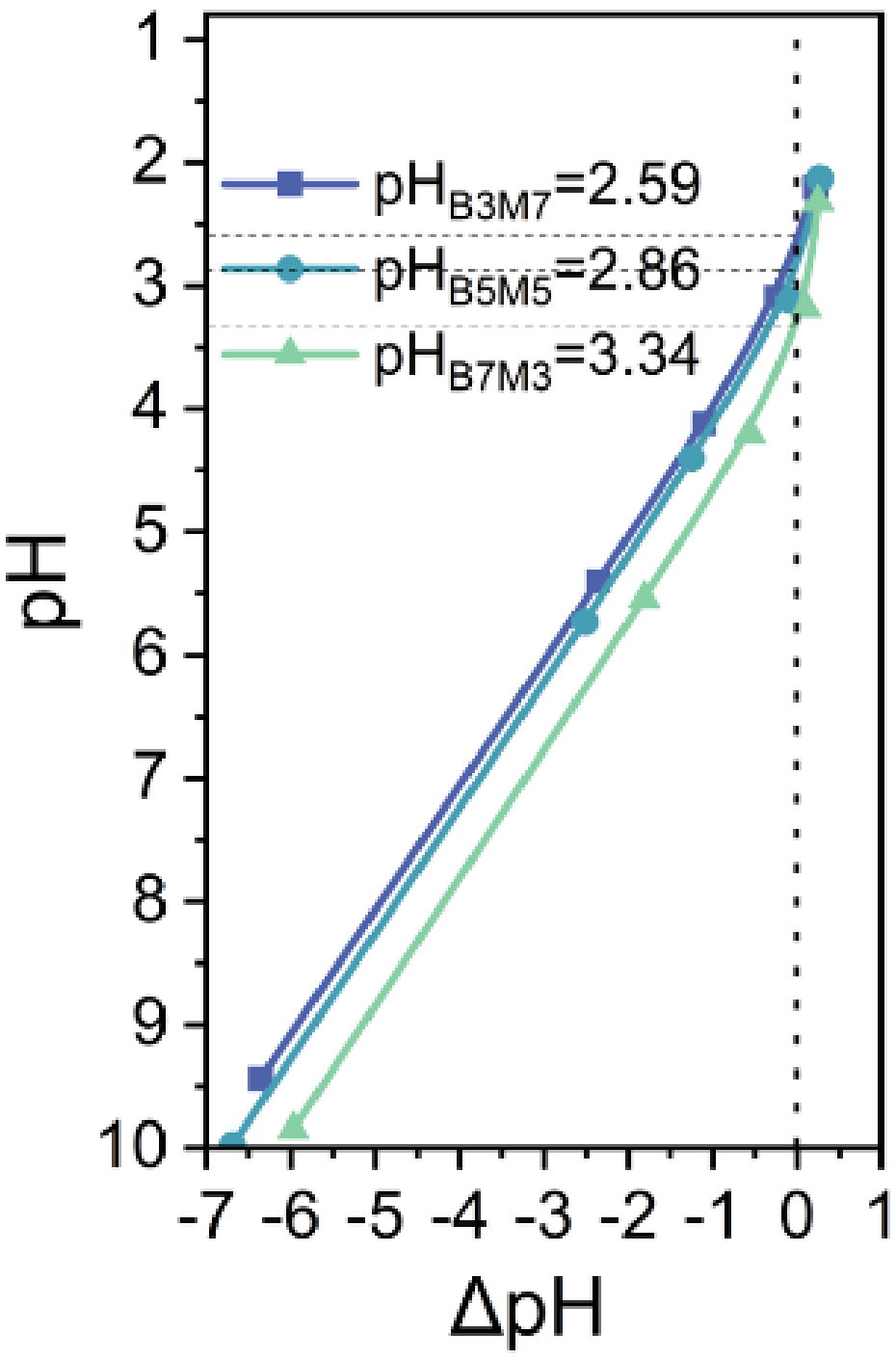

Supplementary Fig.14 | Zeta potential of B_xM_y heterojunctions.

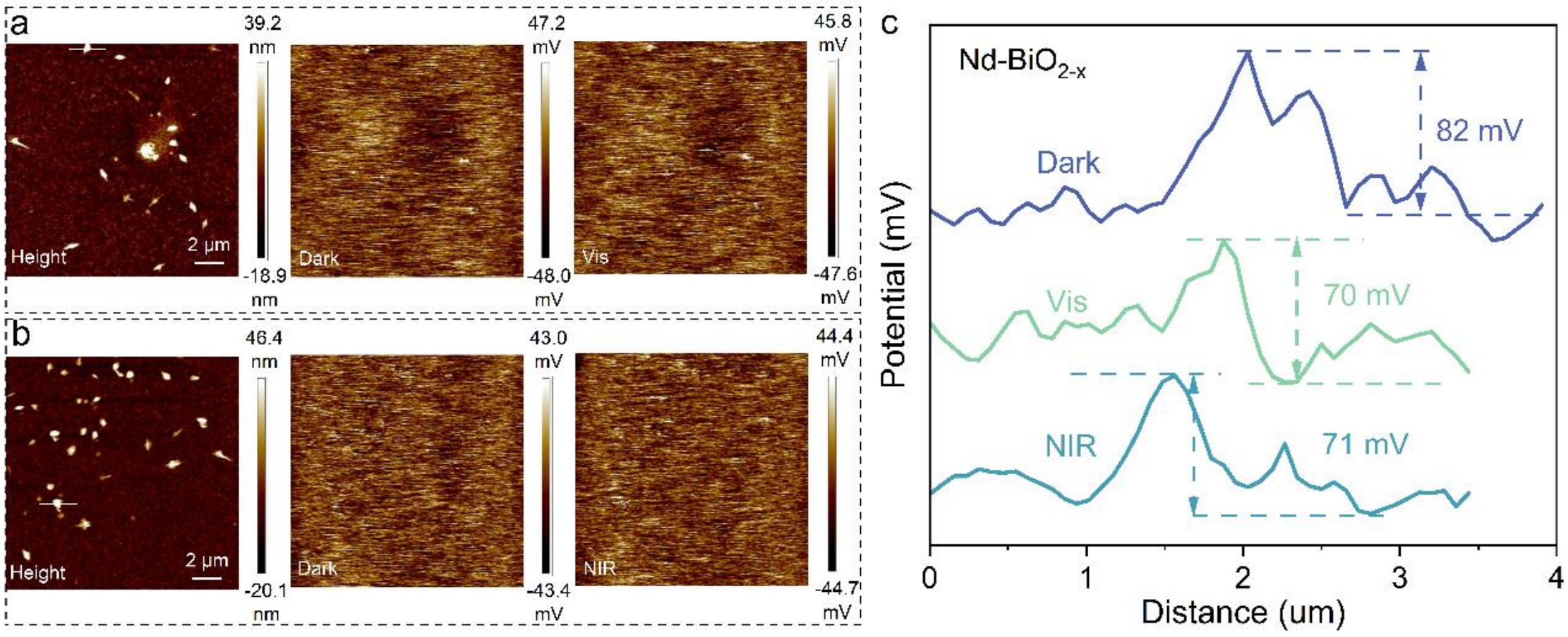

Supplementary Fig.15 | KPFM image of the BiO_{2-x}. (a-b) The KPFM image and **c** corresponding surface potential distribution in darkness, under **(a)** Vis light, and **(b)** NIR light.

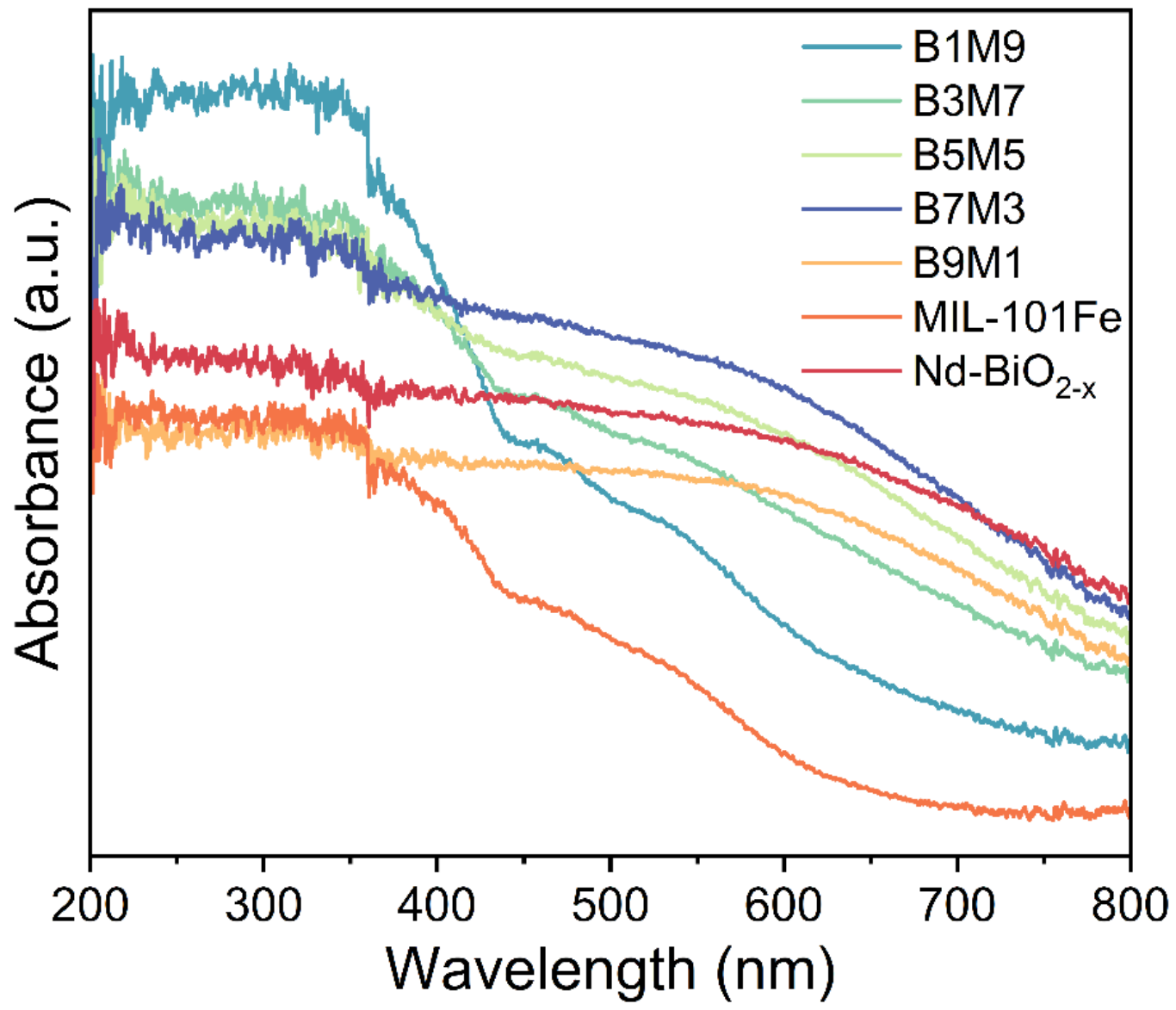

Supplementary Fig.16 | UV-Vis diffuse reflectance spectrum of Nd-BiO_{2-x}, MIL-101(Fe), and B_xM_y heterojunctions.

B_7M_3 exhibited intense light absorption at wavelengths ranging from 400 to 800 nm, even higher than Nd-BiO_{2-x}, suggesting that the enhanced optical absorption and degradation efficiency of B_7M_3 are not attributed to the characteristics of the original materials.

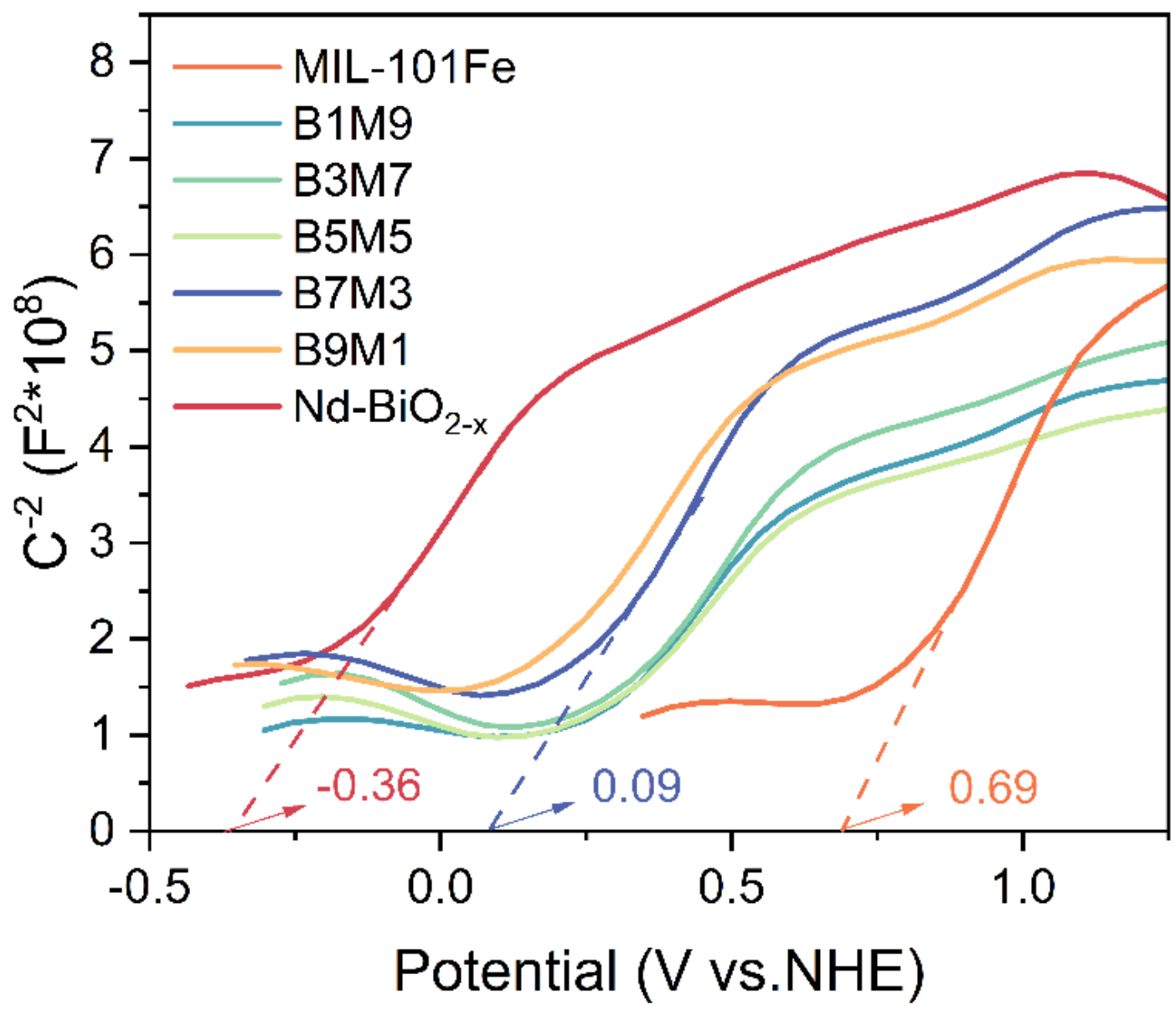

Supplementary Fig.17 | Mott-Schottky (M-S) plot of Nd-BiO_{2-x}, MIL-101(Fe), and B_xM_y heterojunctions.

The E_{fb} of B_7M_3, MIL-101(Fe), and Nd-BiO_{2-x} were determined to be 0.09, 0.69, and -0.36 eV, respectively. All the M-S plots of the tested samples exhibited a positive slope, indicative of n-type semiconductor traits. As is known, the conduction band potential (E_{CB}) of the n-type semiconductor is approximately 0.2 eV more negative than the E_{fb}.[9] The incorporation of Nd-BiO_{2-x} (-0.56 eV) led to a substantial elevation in the conduction band (CB) edge of the B_7M_3 (-0.11 eV) compared with MIL-101(Fe) (0.49 eV).

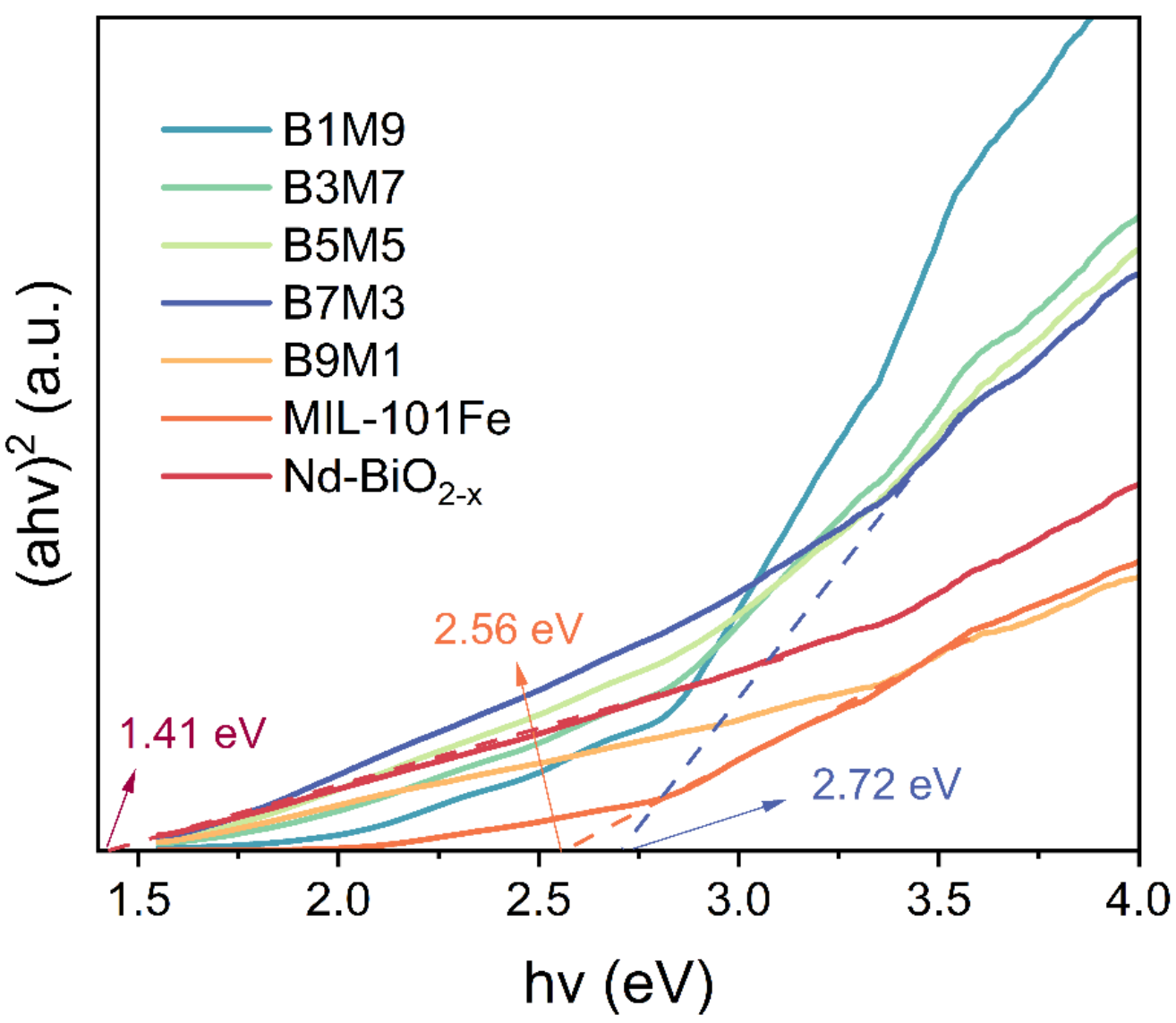

Supplementary Fig.18 | The bandgap value estimated by a related curve of $(\alpha h\nu)^{1/2}$ vs. photon energy plotted of Nd-BiO_{2-x}, MIL-101(Fe), and B_xM_y heterojunctions.

The band gap values (Eg) of the as-prepared samples were estimated from Tauc plots derived from the UV–Vis diffuse reflectance spectra shown in Supplementary Fig. 16 using the Kubelka–Munk (K–M) function (Supplementary Fig. 18).[10] The Eg of B_7M_3, MIL-101(Fe), and Nd-BiO_{2-x} were determined to be 2.72, 2.56, and 1.41 eV, respectively. The narrow Eg of BiO_{2-x} would facilitate light absorption and carrier excitation, thereby promoting carrier transport in the heterojunctions.

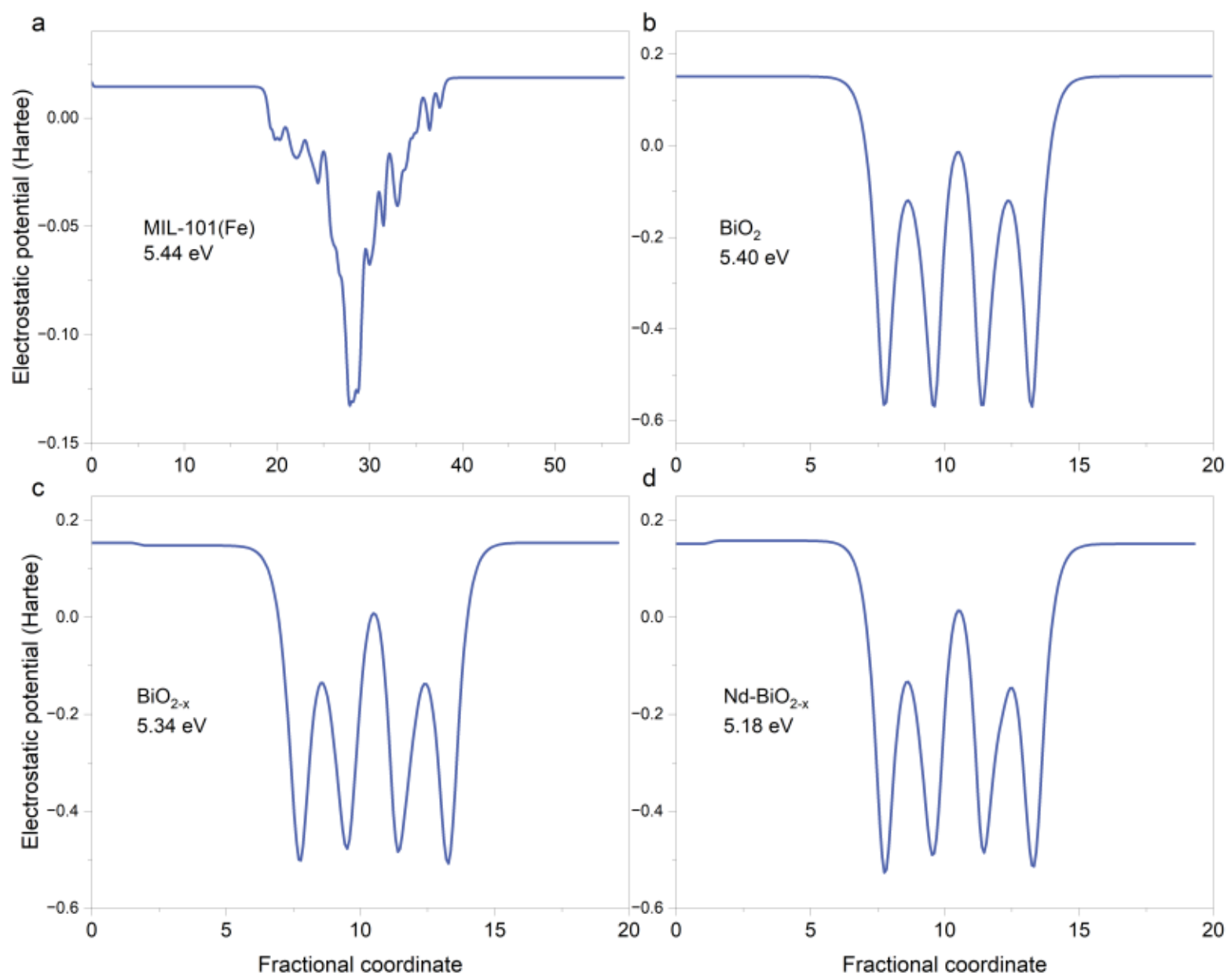

Supplementary Fig.19 | Electrostatic potentials of BiO_2, BiO_{2-x}, Nd-BiO_{2-x}, and MIL-101(Fe).

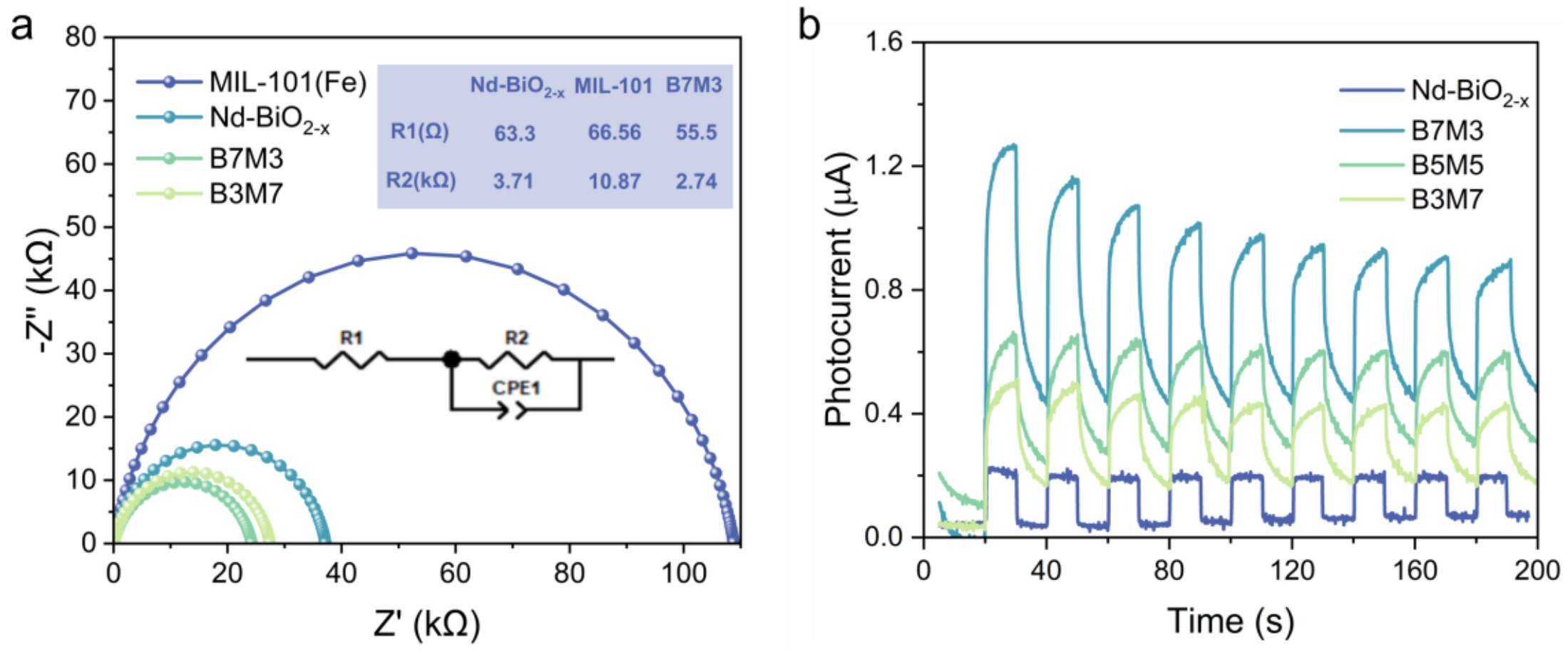

Supplementary Fig.20 | (a) Electrochemical impedance spectroscopy (EIS) Nyquist plots and (b) transient photocurrent responses of as-prepared samples.

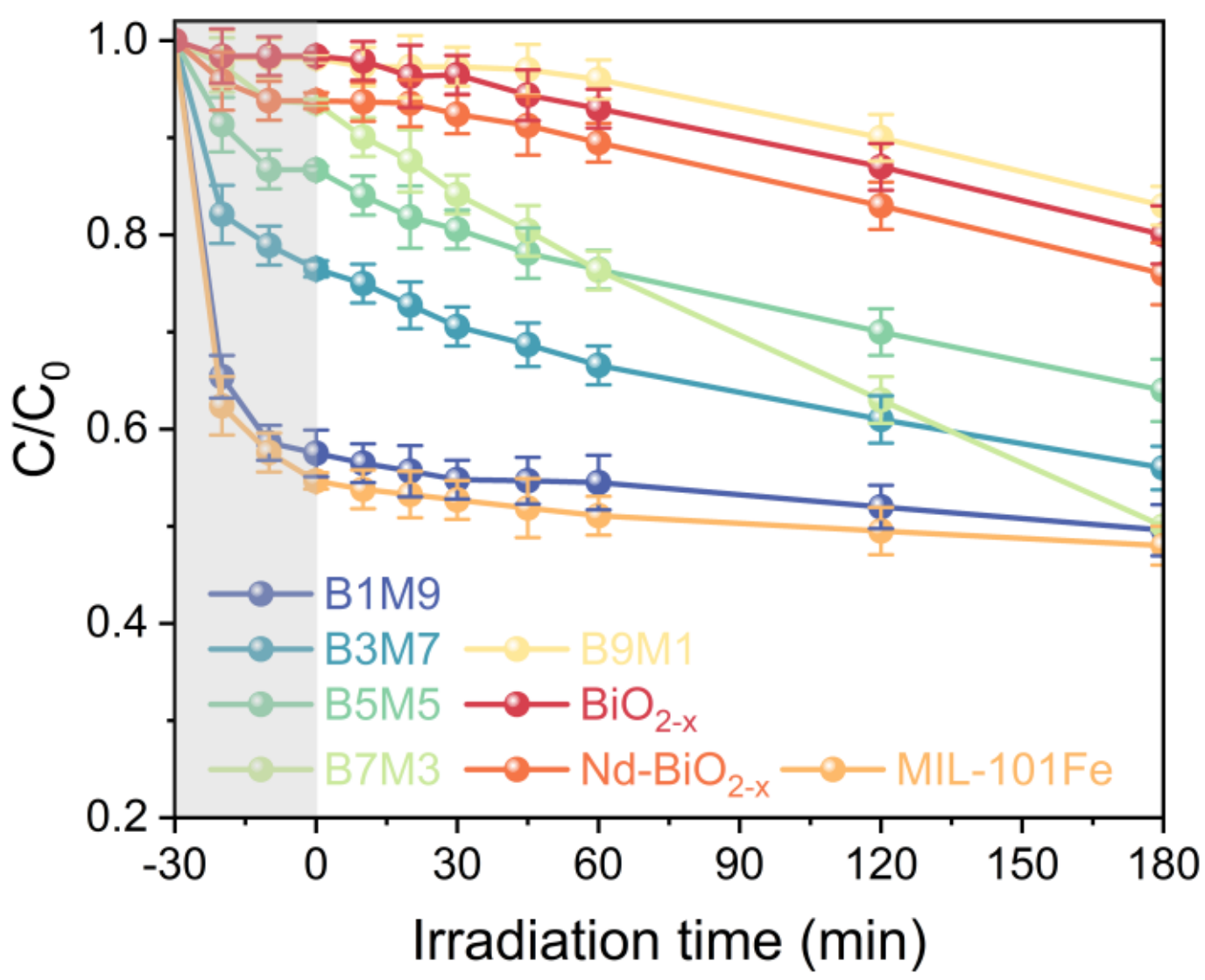

Supplementary Fig.21 | Degradation curves of BPAF with a series B_xM_y under NIR light. Reaction conditions: [BPAF] = 0.06 mM, [Cat.] = 0.2 g L^{-1}, pH_0 = 7.8, and T = 25 ± 3 °C. Data are presented as mean values ± SD (n = 2).

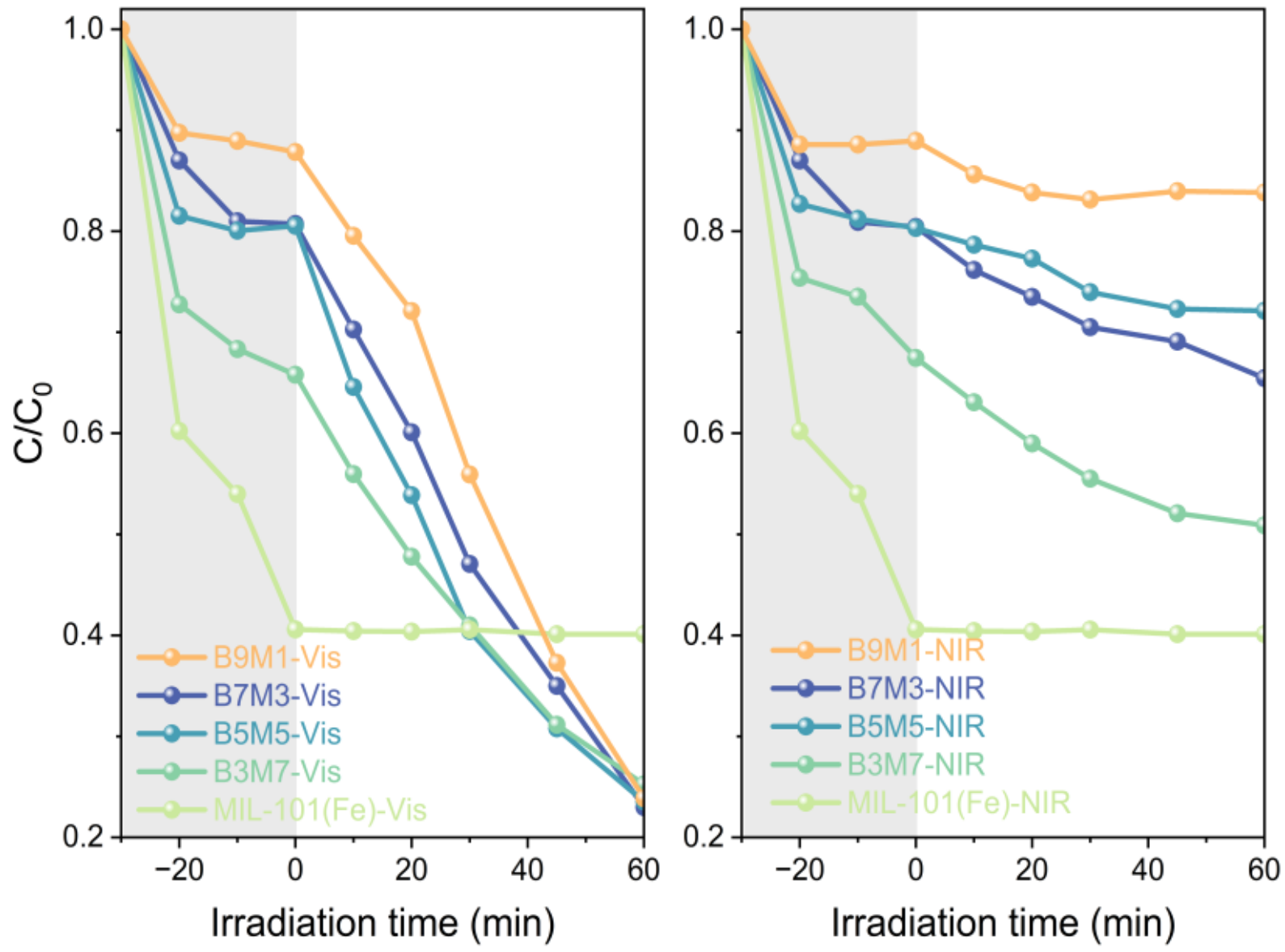

Supplementary Fig.22 | Degradation curves of RhB with a series B_xM_y under Vis and NIR light. Reaction conditions: [RhB] = 0.06 mM, [Cat.] = 0.2 g L^{-1}, pH_0 = 7.8, and $T = 25 \pm 3$ °C.

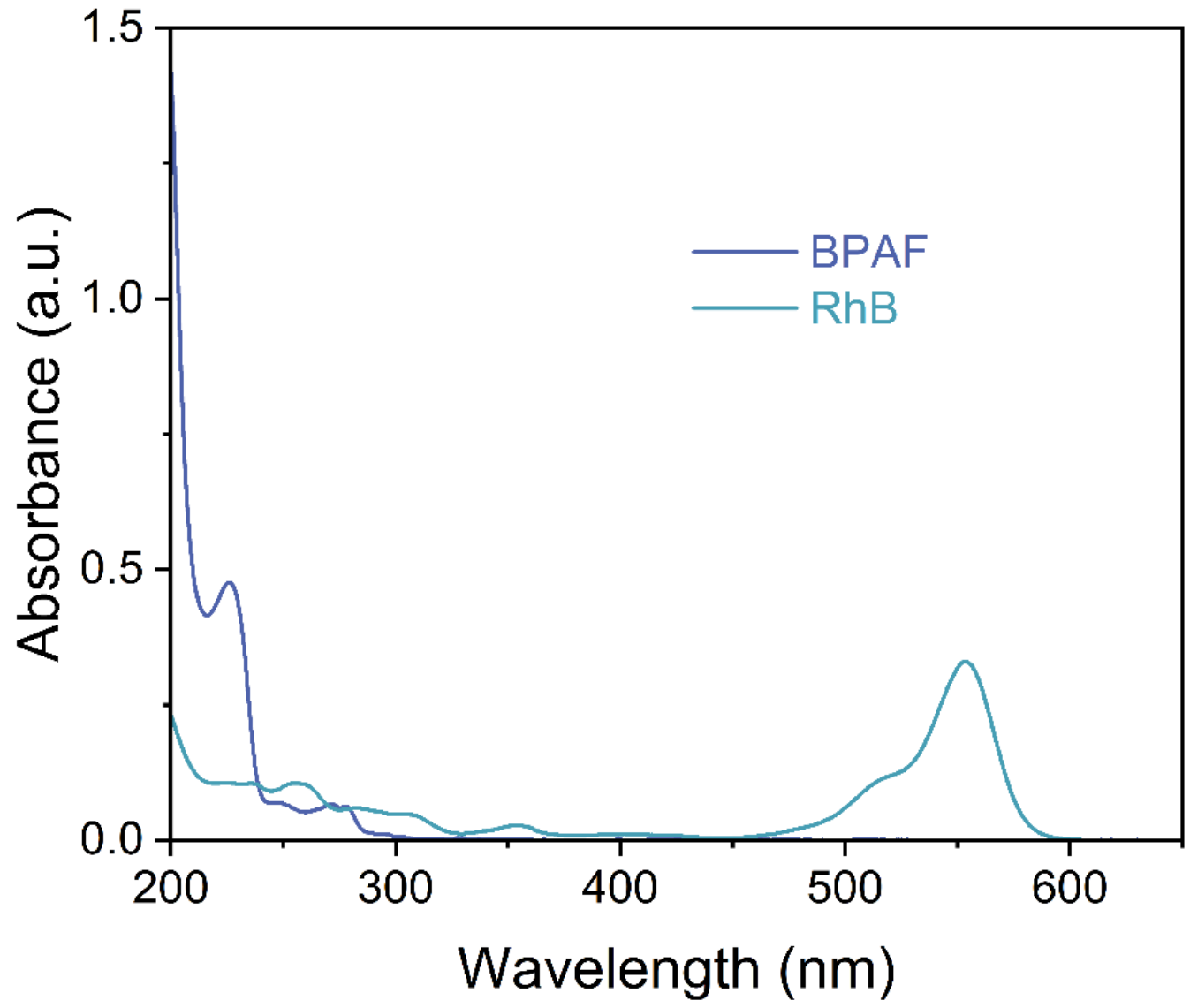

Supplementary Fig.23 | Ultraviolet-visible spectrum of BPAF and RhB (20 mg L^{-1}**).**

RhB had a wide peak (from 450 to 600 nm) in the UV-Vis light absorption spectrum.

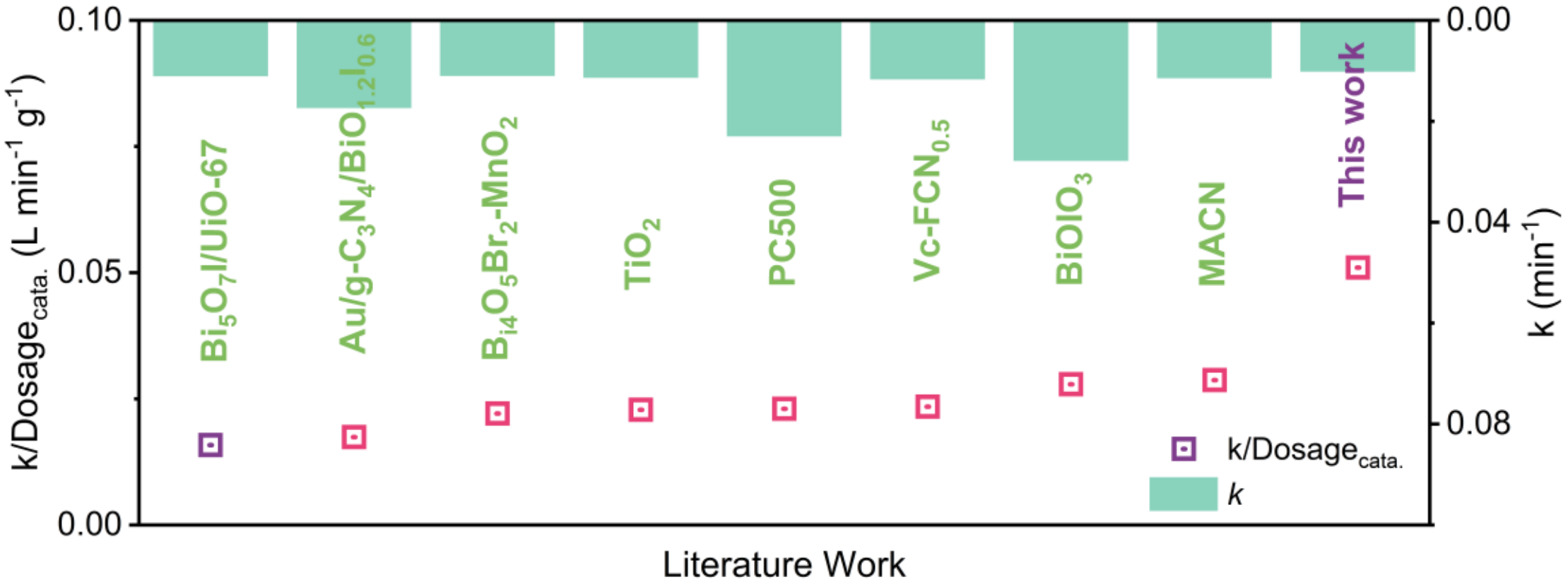

Supplementary Fig.24 | Comparisons of catalytic efficiency with previously reported catalysts[11–17]. The detailed literature comparison is provided in Table S6.

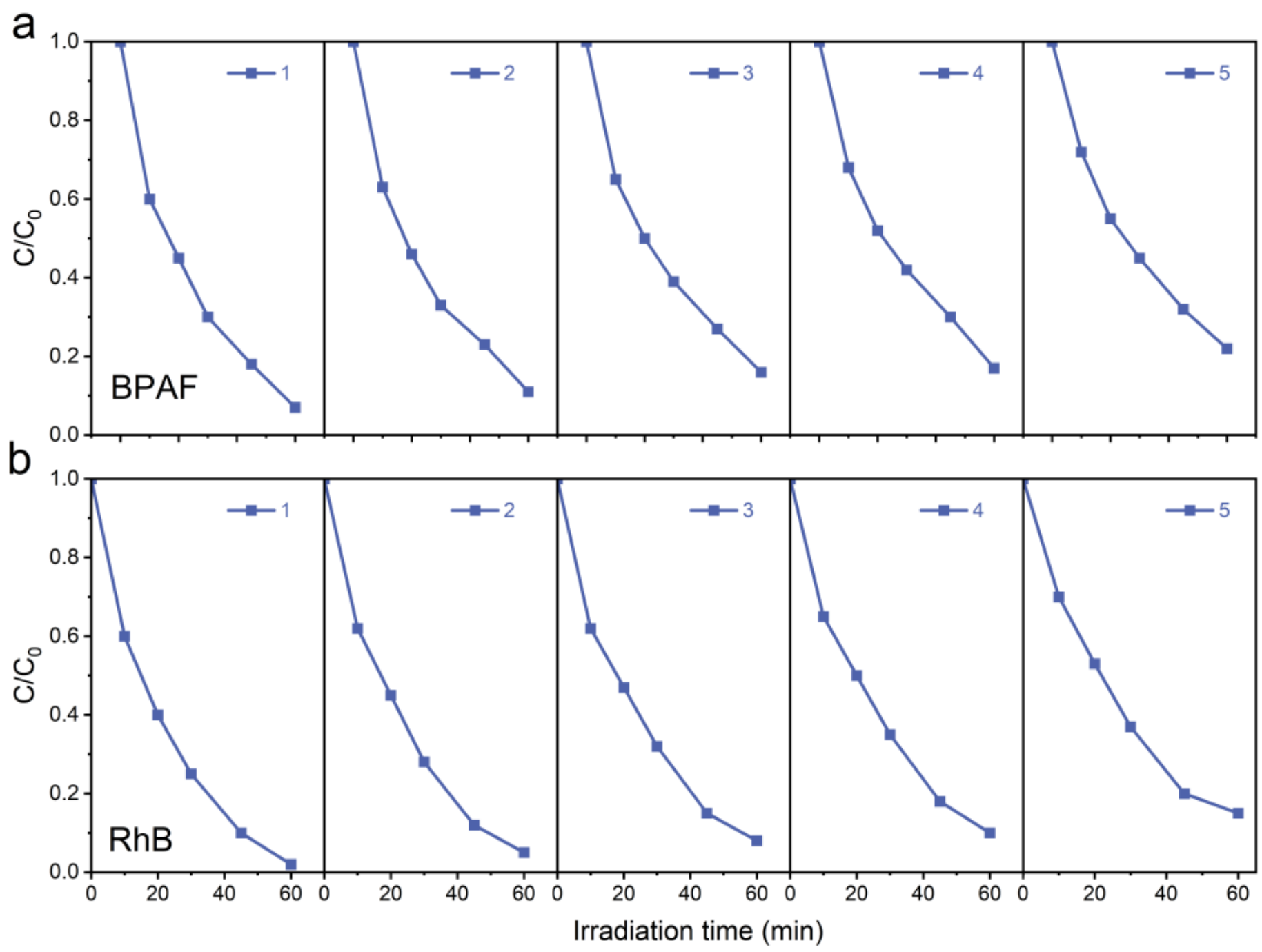

Supplementary Fig.25 | Five consecutive cycling experiments for the degradation of (a) BPAF and (b) RhB under identical experimental conditions. Xenon lamp, [BPAF] = [RhB] = 0.06 mM, [Cat.] = 0.2 g L^{-1}, pH_0 = 7.8, and T = 25 ± 3 °C.

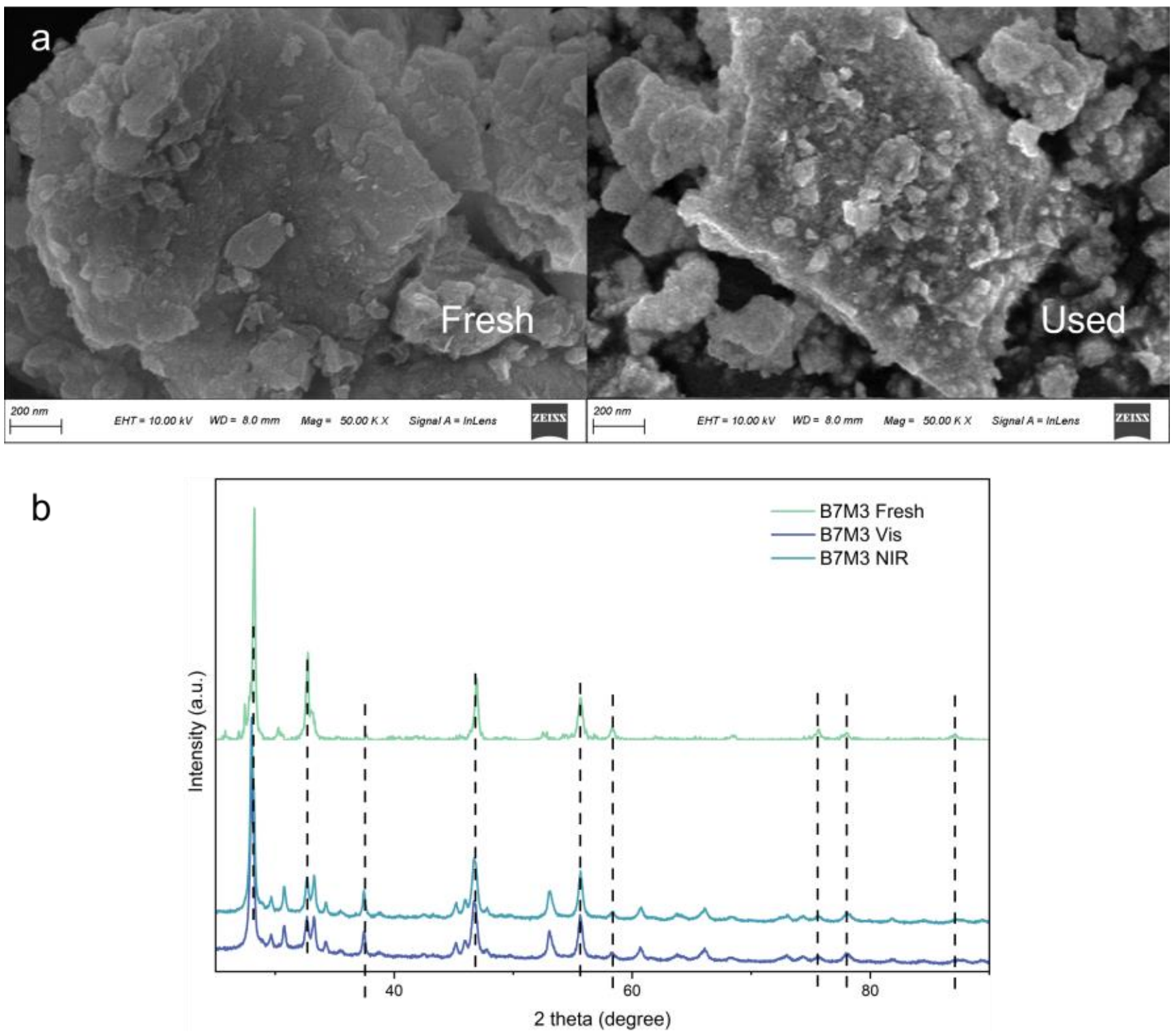

Supplementary Fig.26 | (a) SEM images and (b) XRD spectra of B7M3 before and after five consecutive degradation cycles.

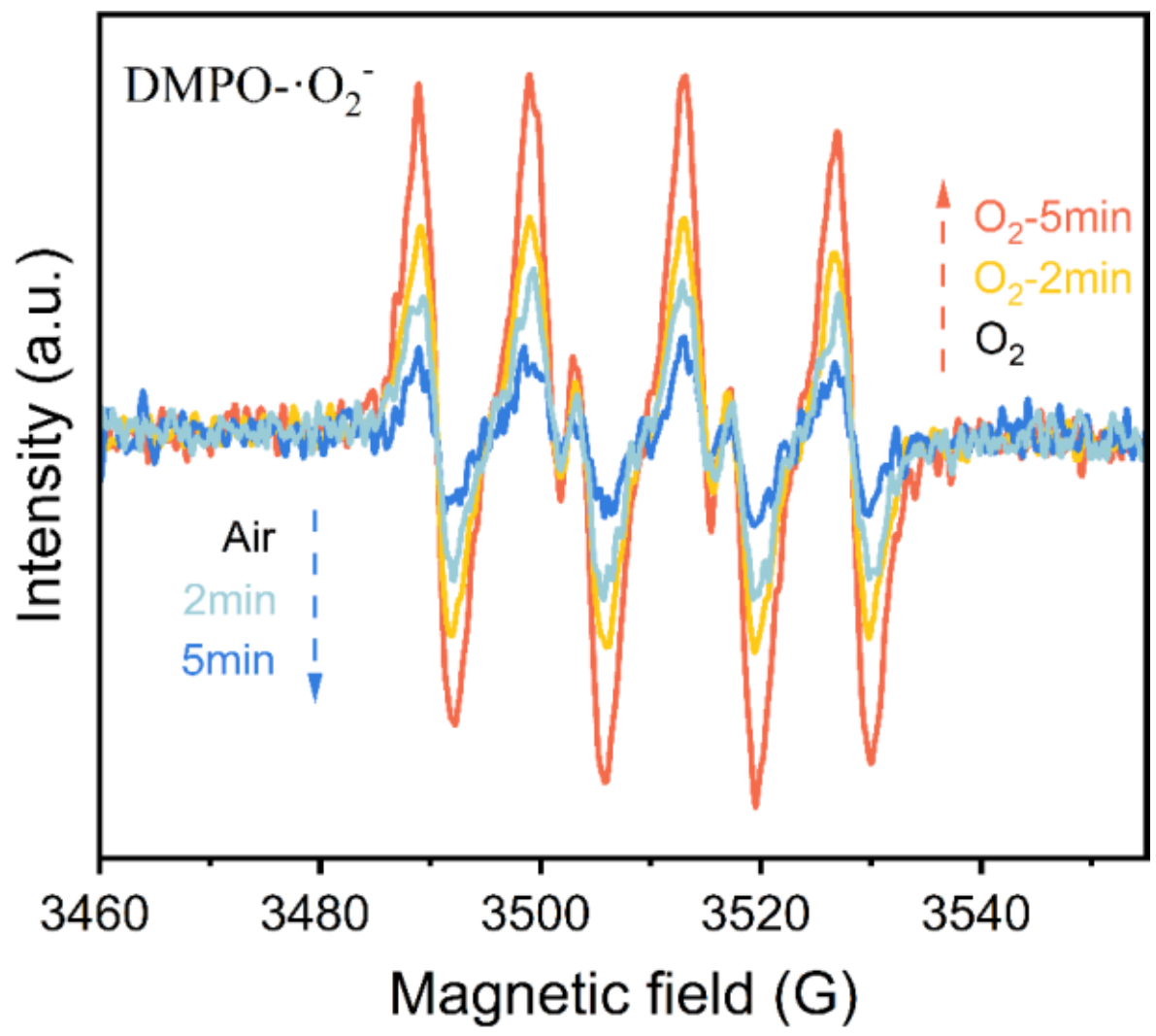

Supplementary Fig.27 | EPR signals for DMPO-•O_2^-.

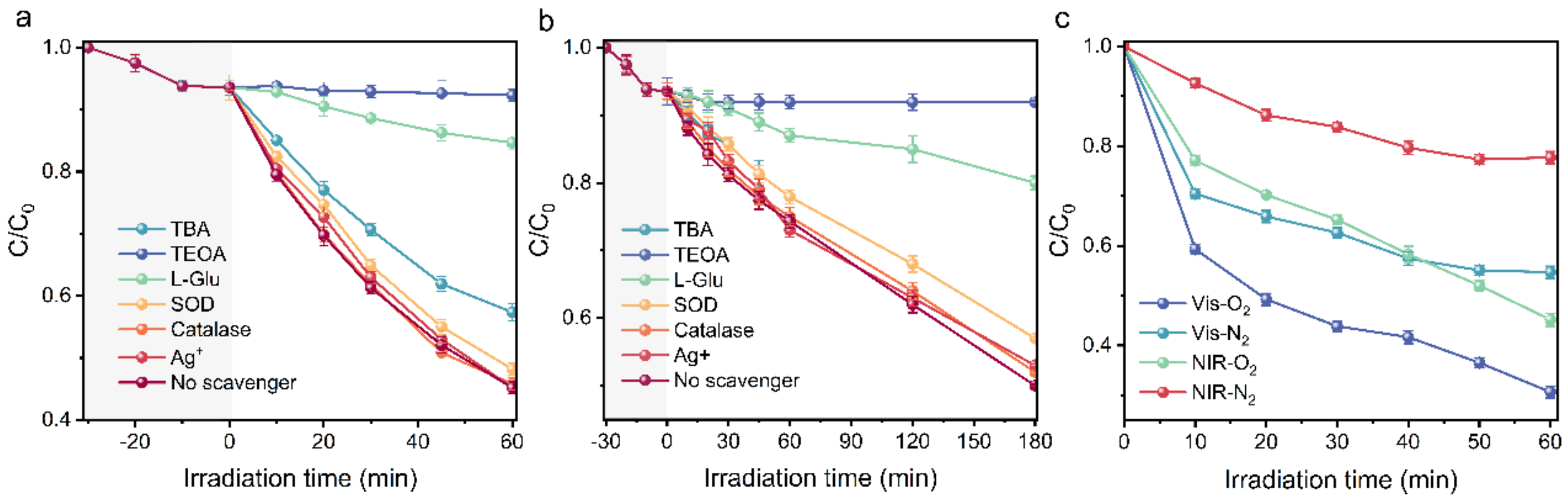

Supplementary Fig.28 | (a)-(b) Effects of quenching agents on BPAF removal under Vis and NIR light, respectively. **(c)** BPAF removal under different gases. Reaction conditions: [BPAF] = 0.06 mM, [B_7M_3] = 0.2 g L^{-1}, $[DMPO]_0$ = 100 mM, $[TEMP]_0$ = 100 mM, $[TEMPO]_0$ = 100 mM, [Scavenger] = 100 mM, $[SOD]_0$ = 100 mg L^{-1}, pH_0 = 7.8, and T = 25 ± 3 °C. Data for (a-c) are presented as mean values ± SD (n = 2).

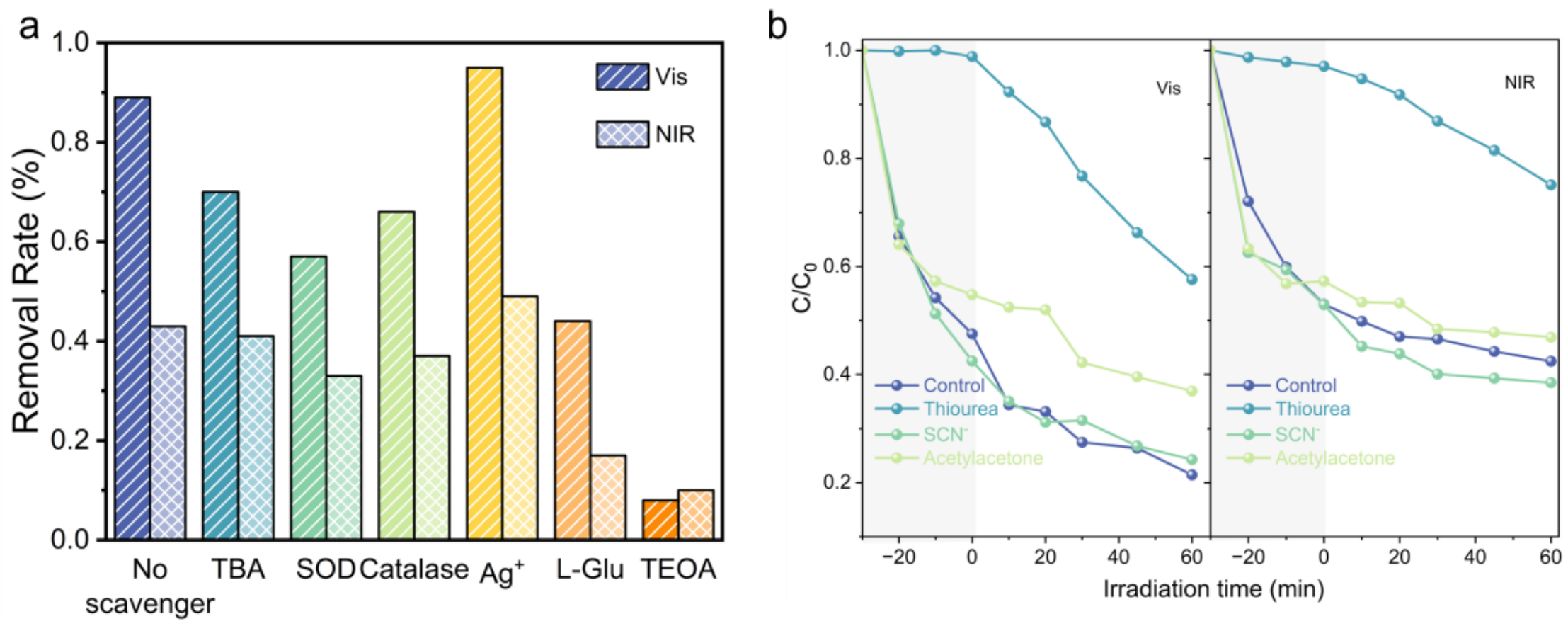

Supplementary Fig.29 | (a) Effects of quenching agents on RhB removal and **(b)** evaluation of active sites through metal masking under Vis and NIR light

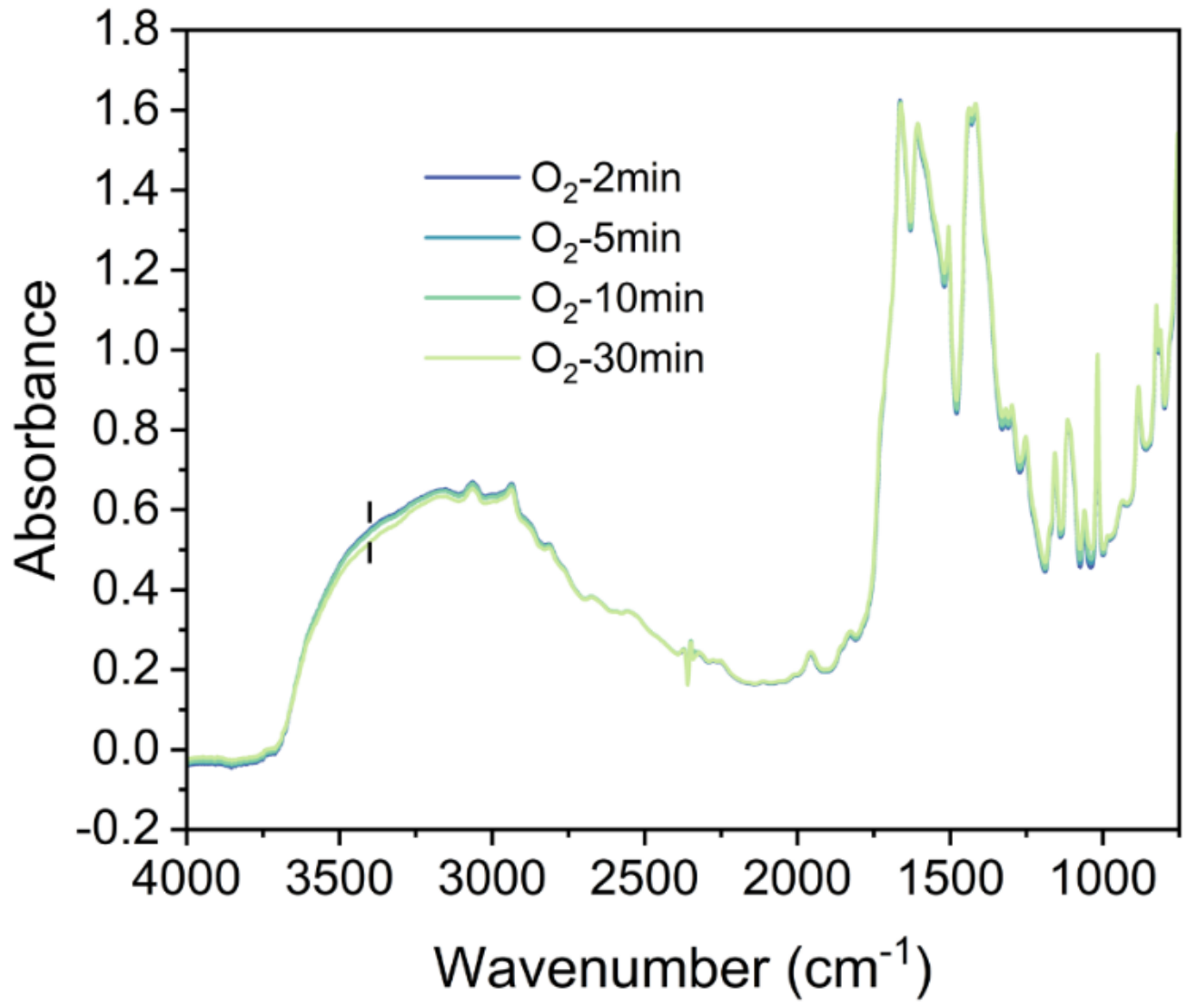

Supplementary Fig.30 | In situ FTIR spectra. In situ FTIR spectra over B7M3 collected at different times under the visible-light irradiation (λ > 420 nm).

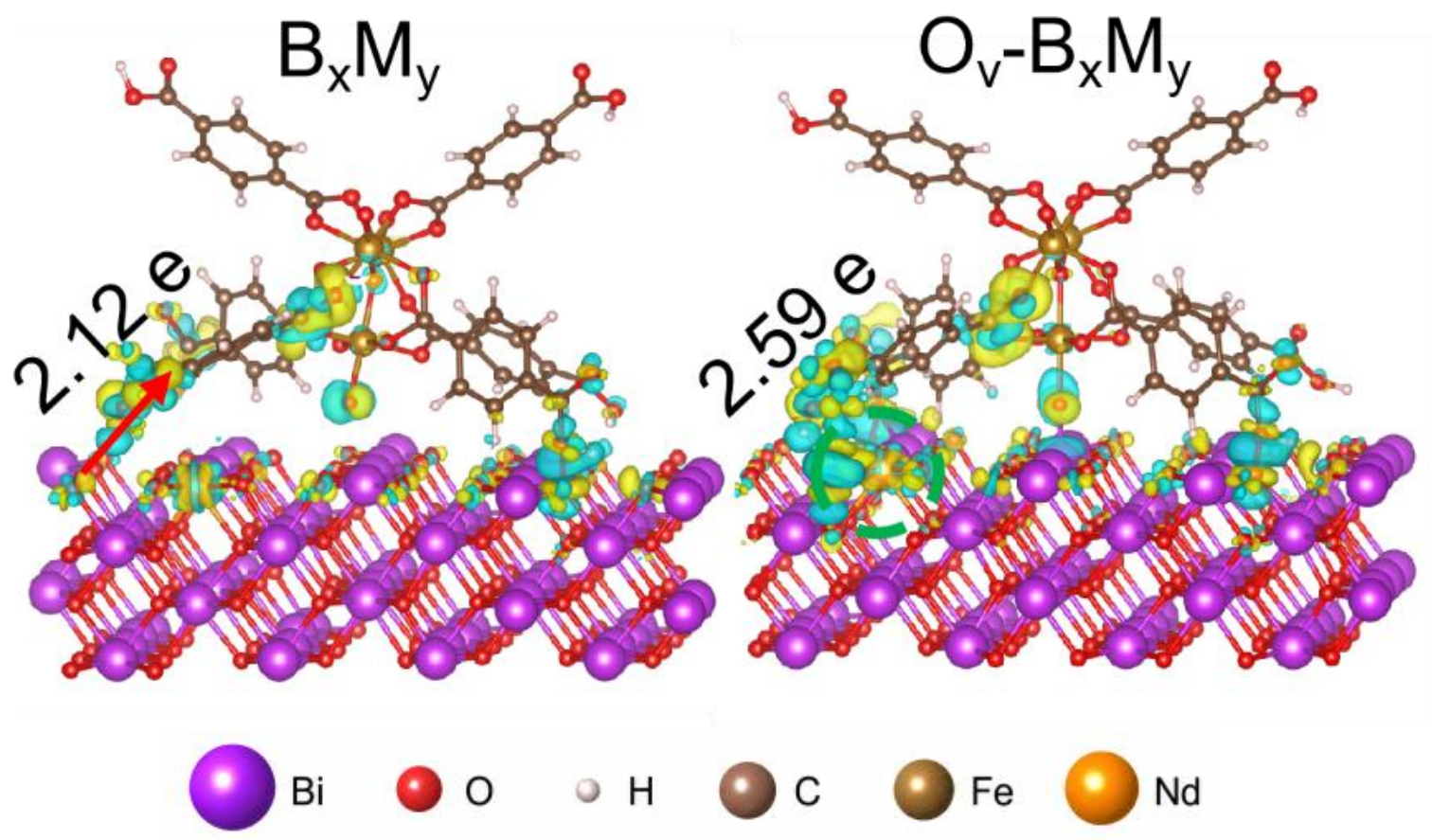

Supplementary Fig.31 | The charge difference distribution of BiO_{2-x}/MIL-101(Fe) or BiO_2/MIL-101(Fe)

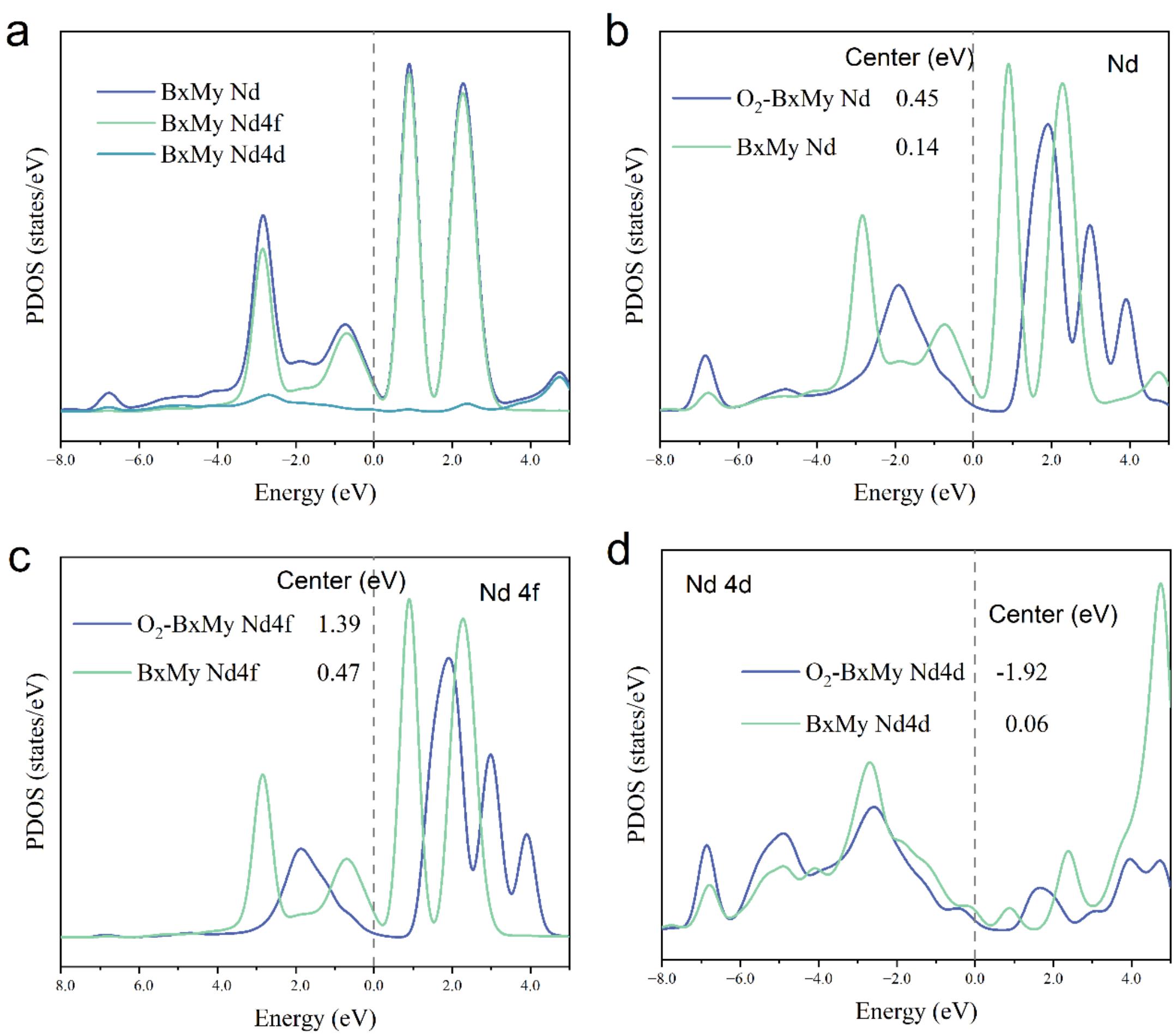

Supplementary Fig.32 | The Nd PDOS of the optimized configurations of BxMy. **(a)** The different Nd PDOS of the optimized configurations of BxMy. **(b-d)** The PDOS of Nd, Nd 4f, and Nd 4d before and after O_2 adsorption.

To further clarify the atomic-level interaction between Nd and adsorbed O_2, the PDOS before and after O_2 adsorption was calculated by DFT. As shown in **Figure S32a**, the electronic states of Nd are mainly dominated by Nd 4*f* orbitals. After O_2 adsorption, the most obvious change occurs in the Nd 4*f* orbitals (**Figure S32c**), where the 4*f*-band center shifts positively from 0.47 to 1.39 eV. This upward shift indicates orbital hybridization between Nd 4*f* and O_2-derived O 2*p* states, reflecting strong electronic interaction between Nd-associated sites and adsorbed O_2. Such electronic modulation supports the chemisorption and activation of O_2 near the Nd–O_V–Bi local structure, thereby facilitating the subsequent generation of reactive species. These results indicate that Nd enhances the catalytic process by regulating the electronic structure of O_V-rich Bi sites rather than acting as a separate nanoparticle active site.

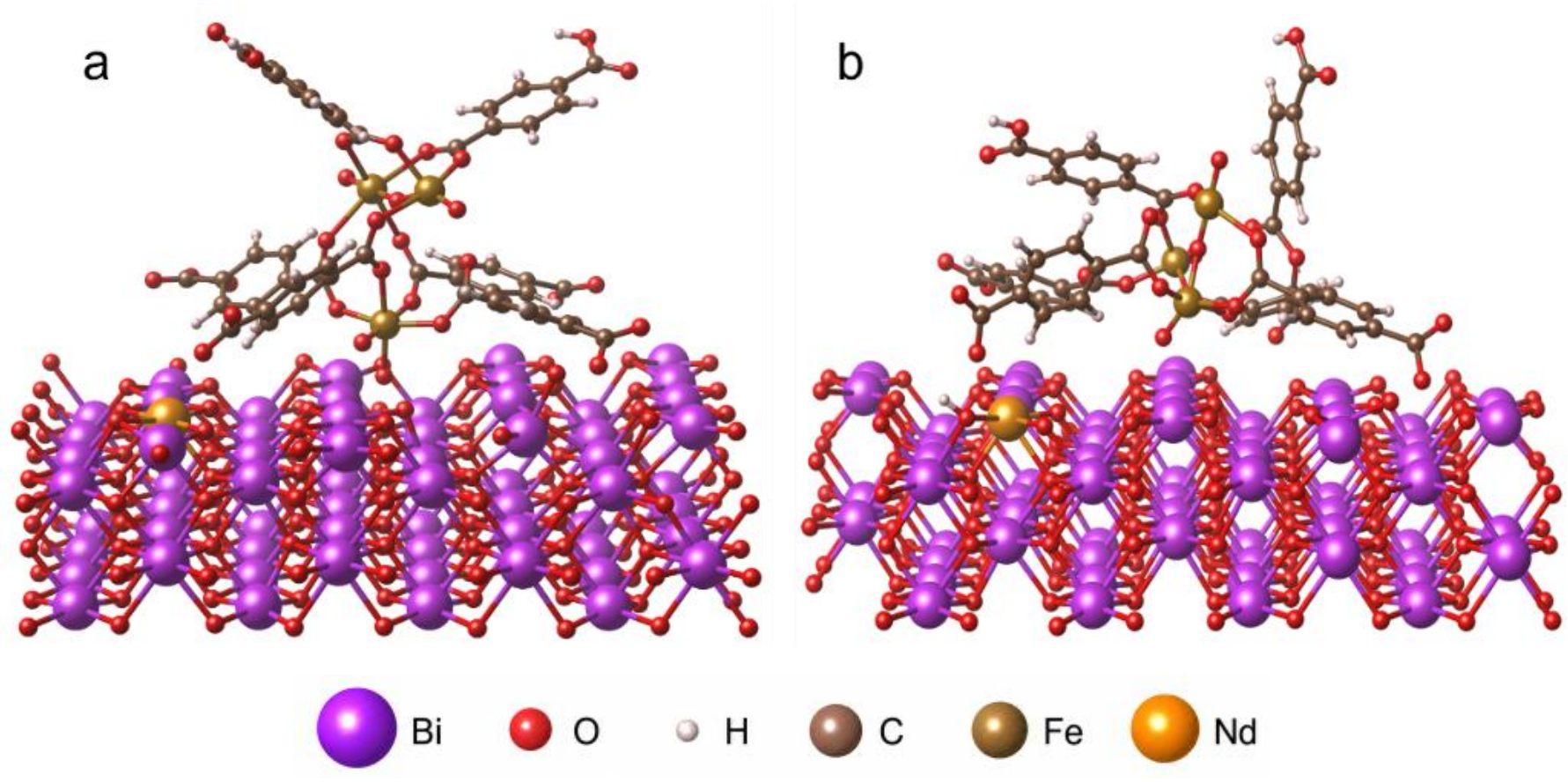

Supplementary Fig.33 | The binding energy of MIL-101(Fe) clusters on the $(01\bar{1})$ surface of **(a)** BiO_{2-x} and **(b)** BiO_2.

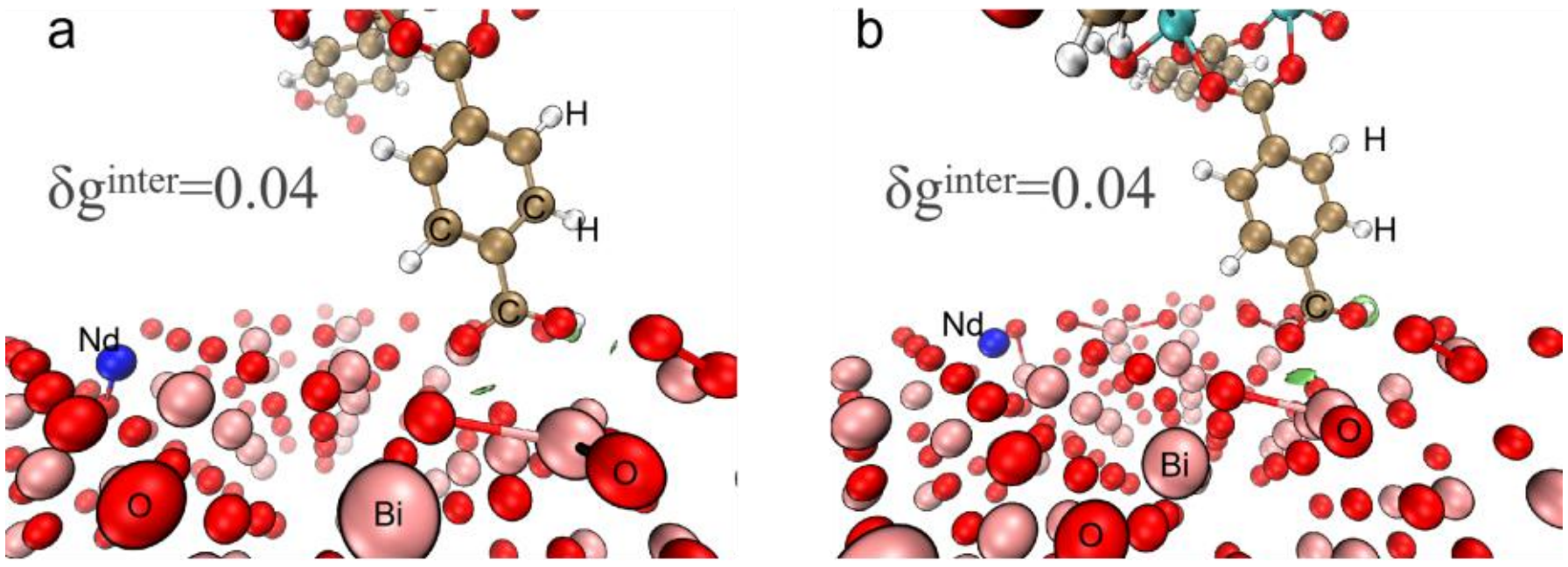

Supplementary Fig.34 | The IGMH of MIL-101(Fe) clusters on the $(01\bar{1})$ surface of **(a)** BiO_2 and **(b)** BiO_{2-x}.

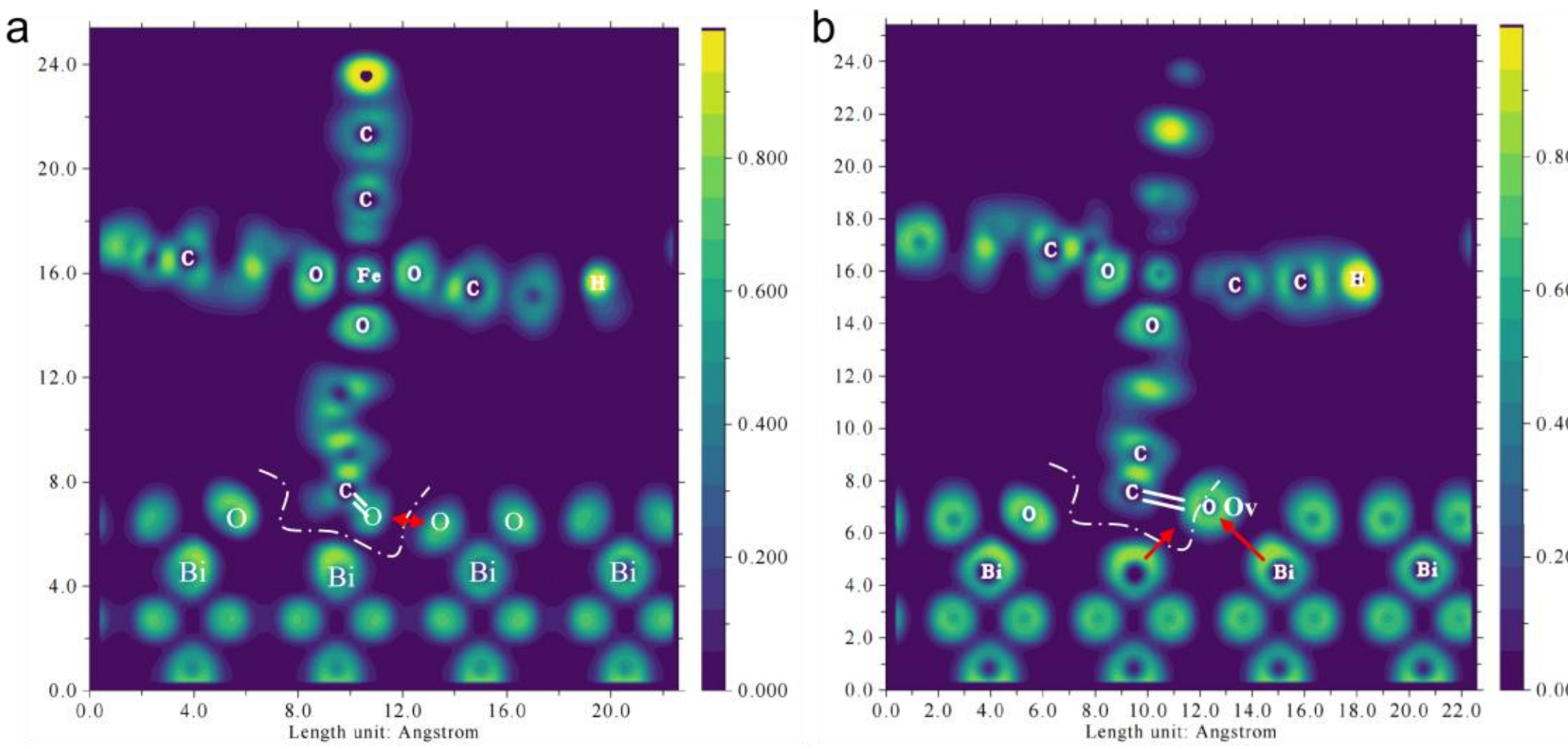

Supplementary Fig.35 | The ELF of MIL-101(Fe) clusters on the $(01\bar{1})$ surface of **(a)** BiO_2 and **(b)** BiO_{2-x}.

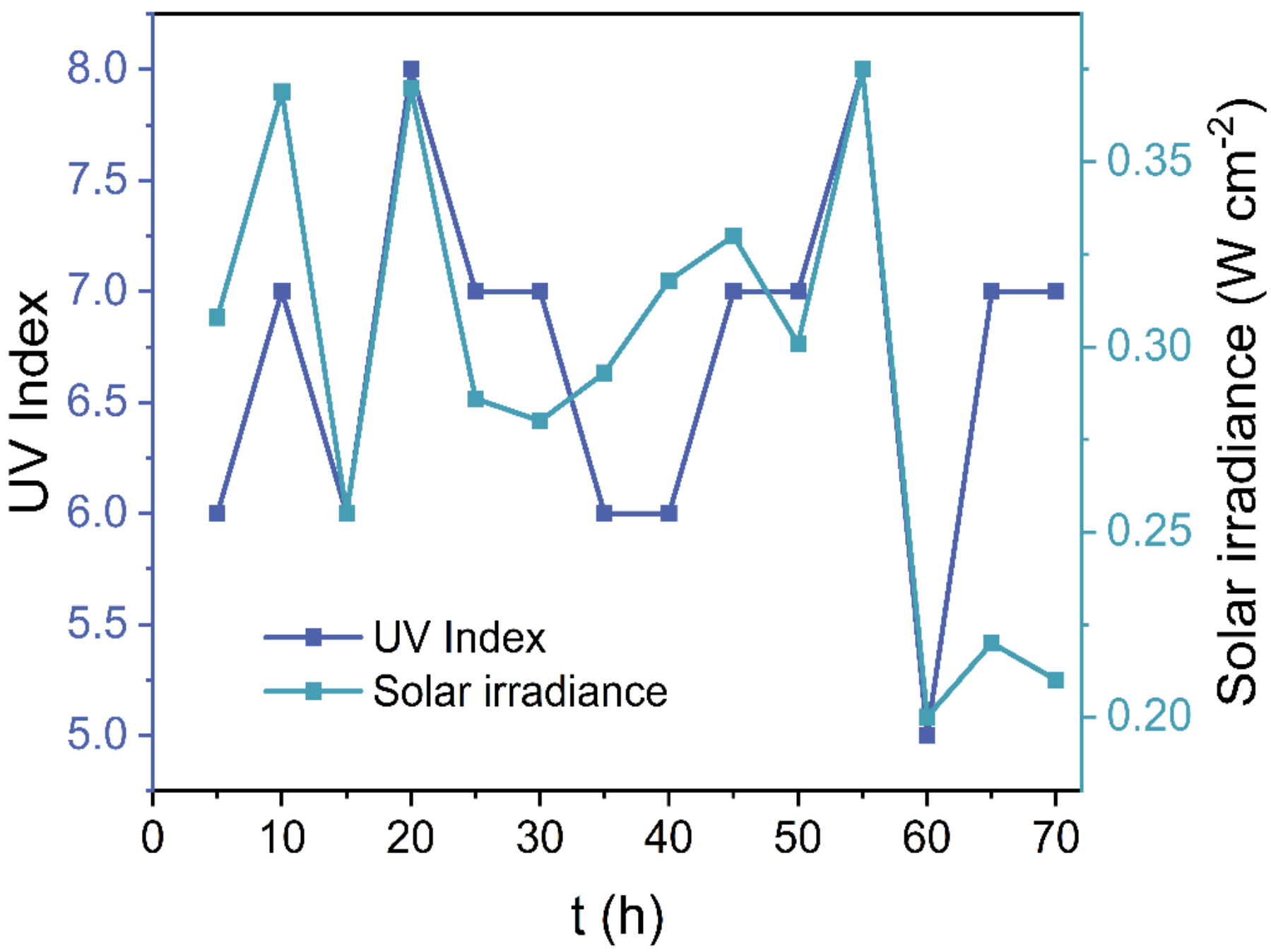

Supplementary Fig.36 | Solar irradiance and ultraviolet intensity at different times.

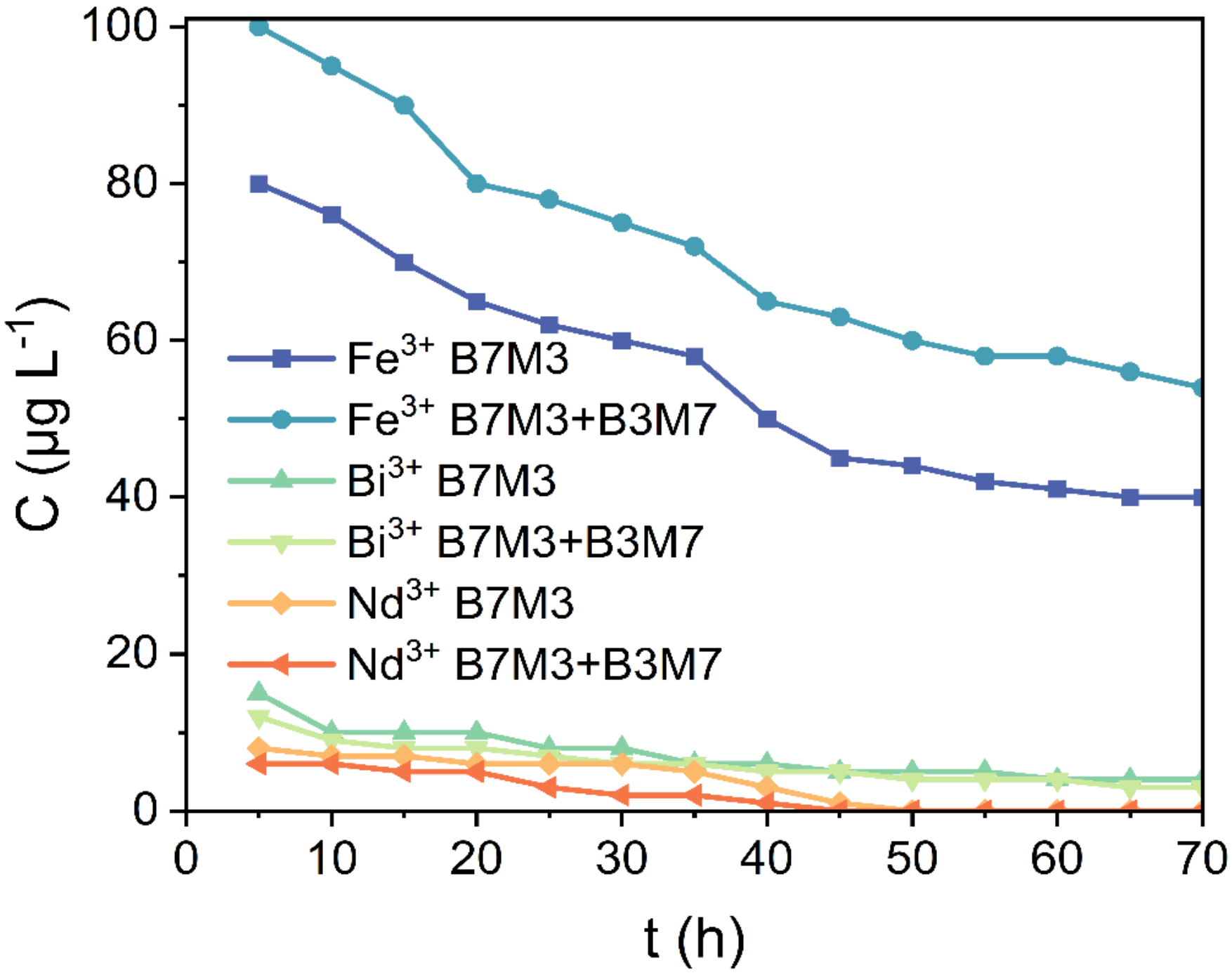

Supplementary Fig.37 | Concentrations of iron ions and bismuth ions in the effluent from the continuous flow experiment.

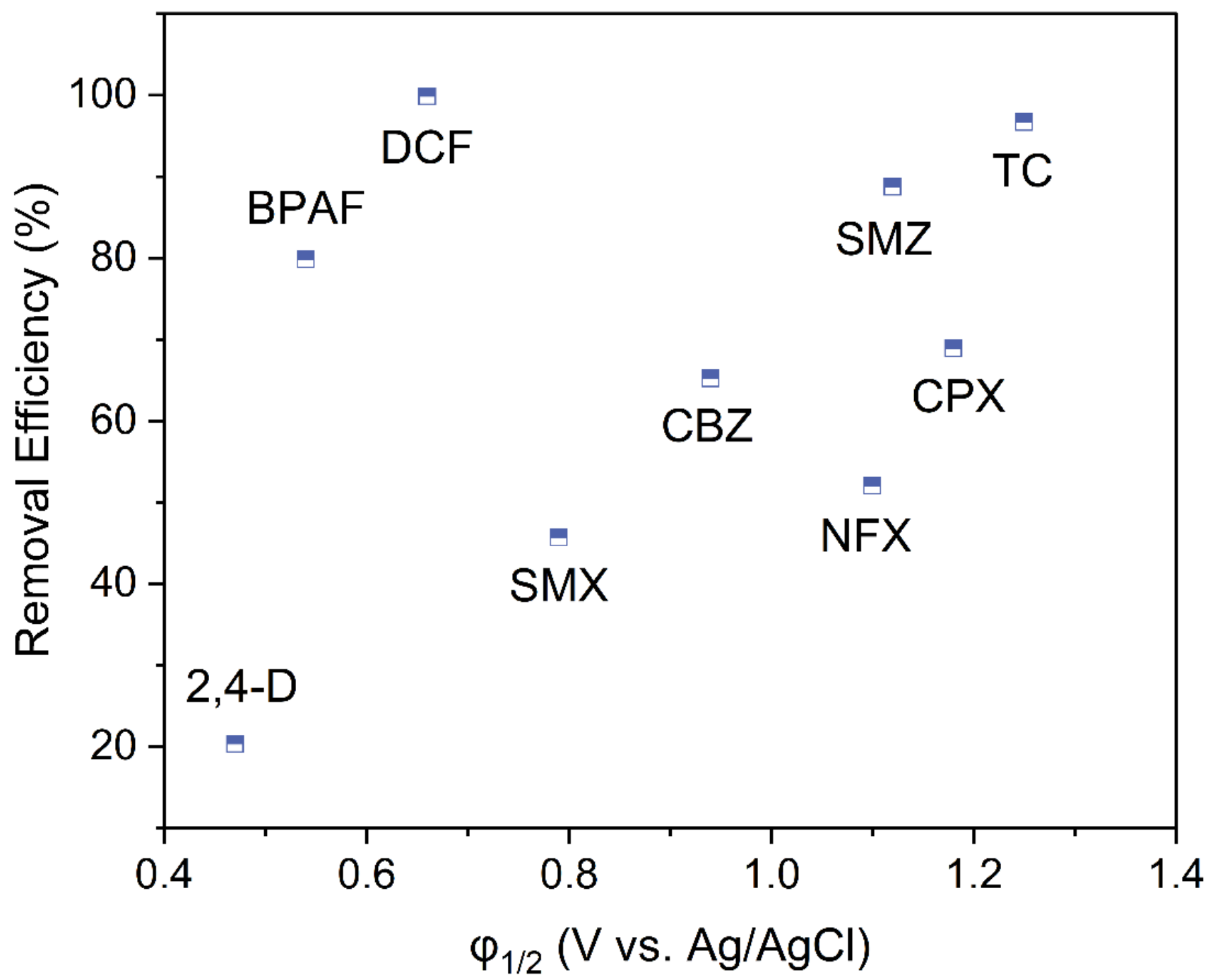

Supplementary Fig.38 | The half-wave potential of different contaminants.

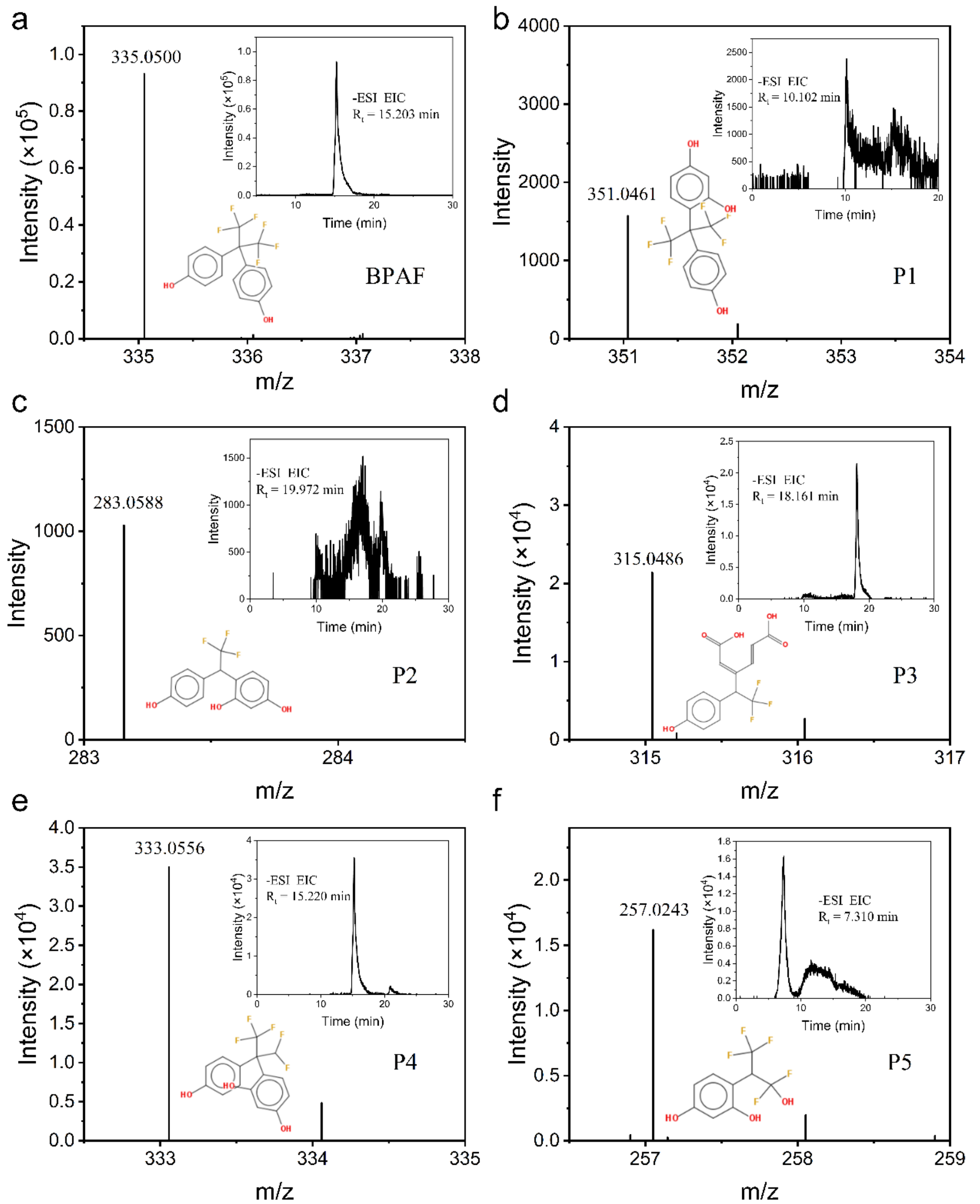

Supplementary Fig. 39 | Mass spectra of BPAF and its intermediate products (P1-P5). Mass spectra and extracted ion chromatograms (insets) of (a) BPAF (m/z 335.0500), (b) P1 (m/z 351.0461), (c) P2 (m/z 283.0588), (d) P3 (m/z 315.0486), (e) P4 (m/z 333.0556), and (f) P5 (m/z 257.0243). The proposed molecular structures of BPAF and the intermediate products are shown in the corresponding panels.

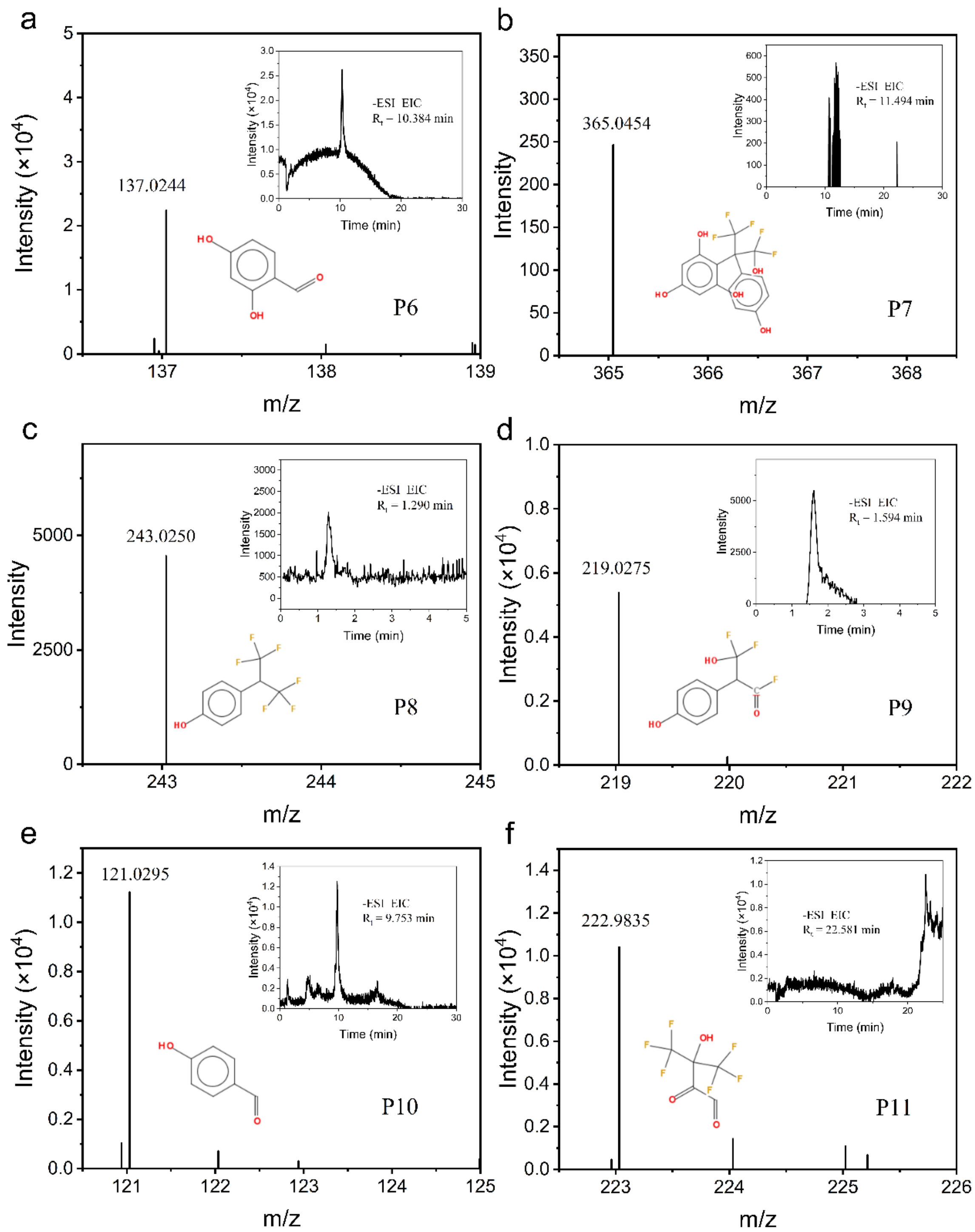

Supplementary Fig.40 | Mass spectra of intermediate products (P6-P11). Mass spectra and extracted ion chromatograms (insets) of (a) P6 (m/z 137.0244), (b) P7 (m/z 365.0454), (c) P8 (m/z 243.0250), (d) P9 (m/z 219.0275), (e) P10 (m/z 121.0295), and (f) P11 (m/z 222.9835). The proposed molecular structures of the intermediate products are shown in the corresponding panels.

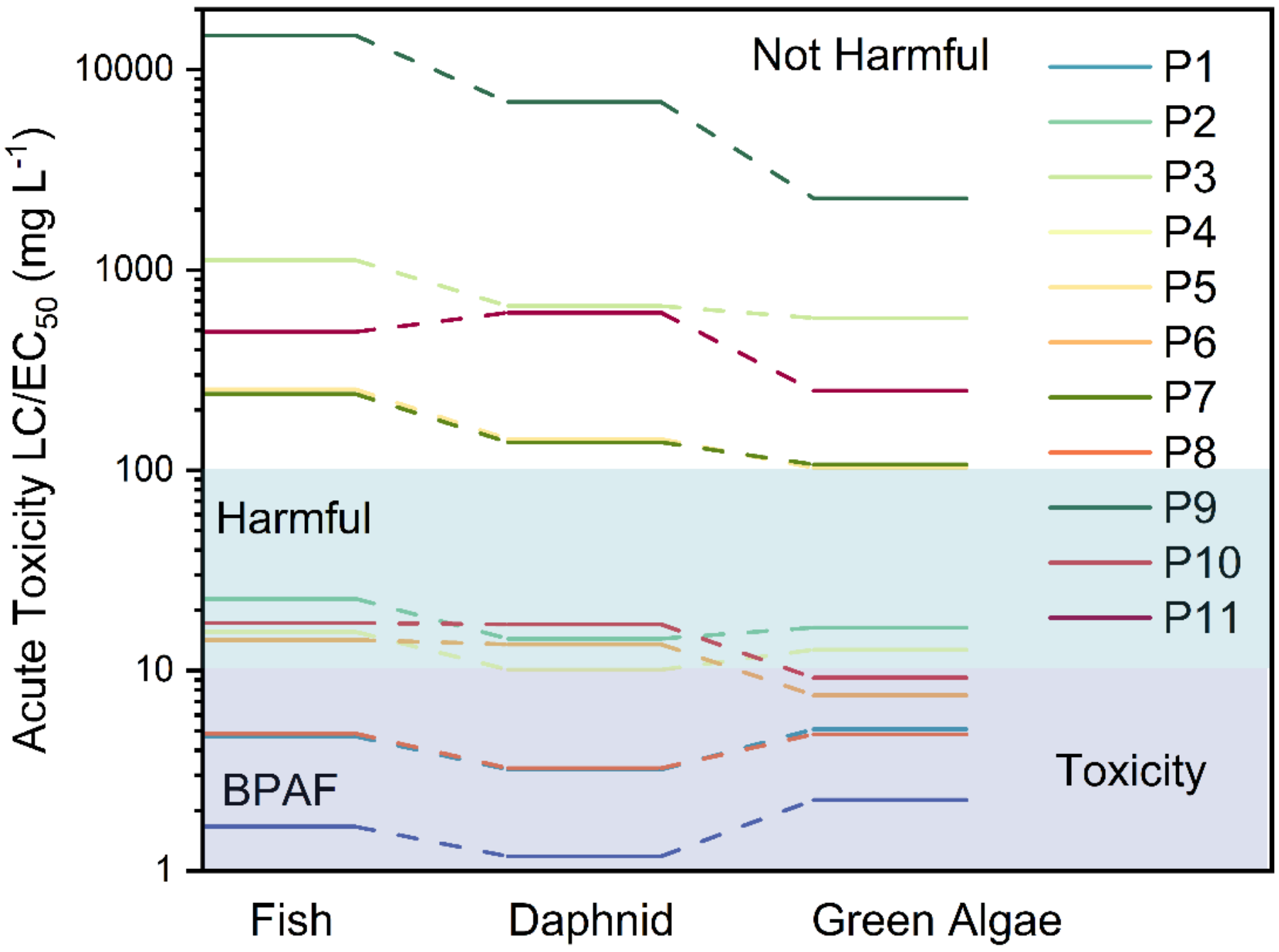

Supplementary Fig.41 | The acute toxicity profiles of BPAF and its intermediate metabolites.

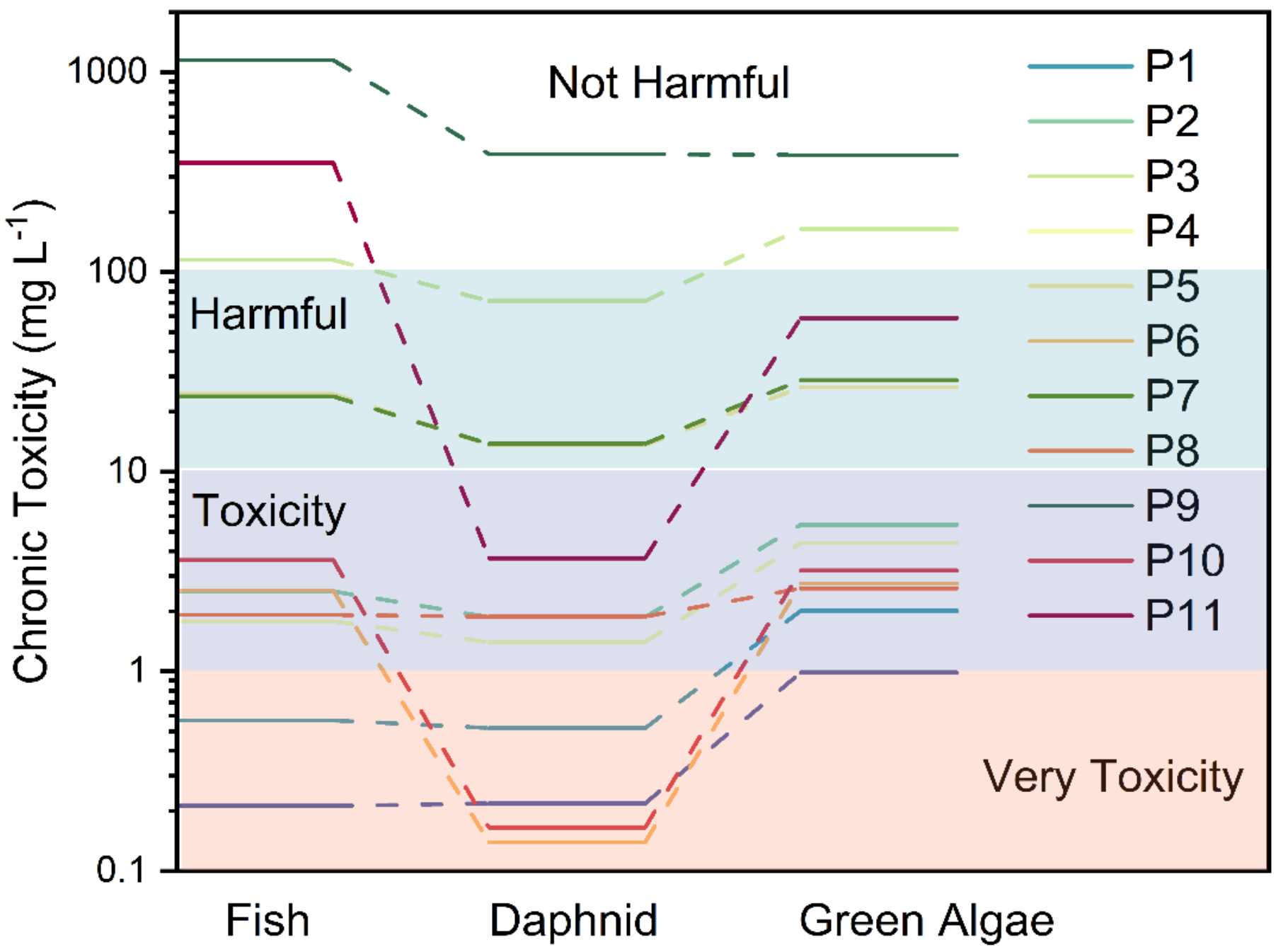

Supplementary Fig.42 | The chronic toxicity profiles of BPAF and its intermediate metabolites.

3 Supplementary Tables

Supplementary Table 1 | The capacity of materials in O_2-TPD and H_2-TPR tests.

Sample	Desorption of O_2 (mmol g^{-1})	Consumption of H_2 (mmol g^{-1})
MIL-101(Fe)	0.1393	6.936
B_7M_3	0.1533	7.539

Supplementary Table 2 | BET surface areas and pore parameters of the as-prepared samples.

Sample	BET surface area (m^2 g^{-1})	Total pore volume (mL g^{-1})	Average pore size (nm)
Nd-BiO_{2-x}	150	0.23	6.12
MIL-101(Fe)	511	0.44	3.46
B_1M_9	122	0.16	5.13
B_3M_7	118	0.16	5.28
B_5M_5	130	0.18	5.46
B_7M_3-20	76	0.10	5.00
B_7M_3-30	137	0.18	5.23
B_7M_3-45	136	0.17	5.01
B_7M_3-60	101	0.13	5.07

Supplementary Table 3 | EXAFS fitting parameters at the Bi L_3-edge and Fe K-edge for various samples.

**Sample**	**Shell**	***CN***	***R* (Å)**	**$\Delta\sigma^2$*10^3 (Å^2)**	***R*-factor**
$Nd\text{-}BiO_{2\text{-}x}$	Bi-O	4.1±1.1	2.06±0.02	6.7	0.012
	$Bi\text{-}O_v$ 1	1.2±0.4	2.55±0.03	4.8	
	$Bi\text{-}O_v$ 2	1.7±0.8	3.01±0.02	4.8	
	Bi-Bi 1	1.0±0.5	3.46±0.01	4.0	
	Bi-Bi 2	2.1±0.7	3.66±0.04	4.3	
MIL-101(Fe)	Fe-O 1	3.0±0.1	1.90±0.05	2.1	0.008
	Fe-O 2	3.0±0.1	2.04±0.05	5.3	
	Fe-Fe 1	1.0±0.1	2.92±0.09	8.4	
	Fe-Fe 2	1.1±0.1	3.35±0.06	6.7	
B_7M_3	Bi-O 1	3.4±0.5	2.14±0.01	5.4	0.015
	Bi-O=C	0.8±0.5	2.45±0.01	6.6	
	$Bi\text{-}O_v$	1.4±0.5	2.98±0.03	3.2	
	Bi-Bi 1	1.3±0.6	3.55±0.02	7.5	
	Bi-Bi 2	1.2±0.3	3.73±0.02	6.9	
	Fe-O 1	1.9±0.1	1.89±0.09	6.2	
	Fe-O 2	3.8±0.1	2.03±0.06	4.5	
	Fe-Fe	1.0±0.1	3.01±0.01	7.2	

CN: coordination numbers; *R*: bond distance; σ^2: Debye-Waller factors; R-factor: goodness of fit.

Supplementary Table 4 | Fitting results of R_1 and R_2 of the EIS Nyquist plots.

Sample	R_1 (Ω)	R_2 (kΩ)
MIL-101(Fe)	66.56	10.87
Nd-BiO_{2-x}	63.30	3.71
B_7M_3	55.50	2.74

Supplementary Table 5 | The fitting results of TRPL lifetimes.

Sample	T_{ave} (ns)	T_1 (ns)	T_2 (ns)
MIL-101(Fe)	4.27811	3.54	9.95
Nd-BiO_{2-x}	4.94997	3.76	10.69
B_7M_3	5.40497	3.79	10.95

Supplementary Table 6 | Comparison of catalytic efficiency for BPAF degradation over B_7M_3 and previously reported catalysts under xenon-lamp irradiation.

Photocatalysis	k (L min^{-1} g^{-1})	k_{obs} (min^{-1})	Dosage (g)	Reference
TiO_2NTAs	0.00274	0.00274	1	[13]
Gd-$BiVO_4$	0.00778	0.00778	1	[14]
BiOBr	0.00918	0.00459	0.5	[15]
Au/g-C_3N_4/$BiO_{1.2}I_{0.6}$	0.0174	0.0174	1	[16]
$BiOIO_3$	0.02787	0.02787	1	[17]
MXene/Bi_5O_7I/PMS	0.04973	0.02984	0.6	[11]
HPW/GR/TiO_2	0.066	0.0033	0.05	[12]

Supplementary Table 7 | Free radicals and their corresponding scavengers.

Scavenger	ROS
TBA	·OH
TEOA	h^+ and Ov
L-Tyr	$^1O_2$
SOD	$\bullet O_2^-$
Catalase	H_2O_2
Ag^+	e^-

Supplementary Table 8 | The concentrations of generated ROSs (μM).

B7M3	$\bullet O_2^-$	$^1O_2$	h^+
Vis	0.5	7.2	59.64
NIR	0.3	2.9	19.46

Supplementary Table 9 | Stability constants of metal ion coordination compounds.[18,19]

Ligand	Metal ion	Number of Ligand	lg βn
Thiourea **$H_2NC(=S)NH_2$**	Bi^{3+}	6	11.9
KSCN	Bi^{3+}	6	6.1
	Fe^{3+}	6	6.1
Acetylacetone **$CH_3C(O)CH_2C(O)CH_3$**	Fe^{3+}	3	26.7
Triethanolamine[20,21]	Bi^{3+}	6	-
**$N(CH_2CH_2OH)_3$**	Fe^{3+}	6	-

Supplementary Table 10 | The structural information of the possible intermediate products.

Name	Observed m/z	Formula	Retention Time
BPAF	335.0500	C15 H10 O2 F6	15.203
P1	351.0461	C13 H10 O3 F6	10.102
P2	283.0588	C14 H11 O3 F3	19.972
P3	315.0486	C14 H11 O5 F3	18.161
P4	333.0556	C15 H11 O3 F5	15.220
P5	257.0243	C9 H7 O3 F5	7.310
P6	137.0244	C7 H6 O3	10.384
P7	365.0454	C15 H11 O5 F5	11.494
P8	243.0250	C9 H6 O F6	1.269
P9	219.0275	C9 H7 O3 F3	1.594
P10	121.0295	C7 H6 O2	9.753
P11	222.9835	C5 H2 O3 F6	18.00

Supplementary Table 11 | The quantitative and qualitative analysis of mass-to-charge ratios (m/z) of ionic fragments.

Contaminants	m/z	ES +/-	Quantitative m/z	Qualitative m/z
2,4-D	221.04	-	160.9	125.0
ATZ	215.68	+	68.1	174.1
SMZ	264.30	+	92.0	156.0
CBZ	236.27	+	194.0	179.0
TC	444.43	+	154.0	410.0
BZT	361.82	+	274.0	153.9
NFX	319.34	+	233.1	276.1
CPX	331.34	+	247.0	318.0
BPAF	335.05	-	265.16	197.09
DCF	318.13	+	250.0	214.0
SMX	253.28	+	92.0	156.0/108.0

4 Supplementary Notes

Supplementary Note 1 | Identification of the Ov and the formation of the Bi−O=C bond.

We observed the signal of O_v in both the Raman spectra of the BiO_{2-x} and B_7M_3. The two distinct peaks of B_7M_3 at 440 cm^{-1} and 590 cm^{-1} correspond to the vibrations associated with Bi−O bonds and O_v, respectively **(Supplementary Fig. 9)**. The C=O−Fe band at 1500 cm^{-1} is observed in MIL-101(Fe), whereas the peak appears at the same position in B_7M_3, correspond to the vibration of Bi−O=C. These results are consistent with the FTIR spectra in **Figure 1d**.

Further characterization of the surface properties can elucidate the interfacial mechanisms of photocatalysis, serving as indirect evidence for the C=O−Fe bond. **Supplementary Fig. 12** shows that MIL-101(Fe) and BiO_{2-x} exhibited excellent hydrophilicity (contact angles < 35°). However, after ball milling, the decrease in interfacial hydrophilicity (contact angles > 50°) is attributed to the ionic bonding coordination between carboxyl groups and Bi. The increase in contact angle will lead to the easier accumulation of organic pollutants at the interface, thereby enhancing the reaction rate of photocatalysis. As the ball milling time increases, the continuous reduction of O_v signals and contact angles will be detrimental to the catalytic reactions.

The pore characteristics and specific surface area of the samples are shown in **Supplementary Fig. 13** and **Supplementary Table 2**. As the BiO_{2-x} content increases in the B_xM_y composites, the BET surface area increases from 122 to 137 m^2 g^{-1}, and the total pore volume increases from 0.16 to 0.18 cm^3 g^{-1}. Compared with MIL-101(Fe), the composites show lower surface areas and pore volumes, which can be attributed to interfacial bonding and partial coverage of the MIL-101(Fe) surface by sheet-like BiO_{2-x}.[1] The specific surface area is 76 m^2 g^{-1} with 20 minutes of ball milling, increases to 137 m^2 g^{-1} after 30 minutes. For B_7M_3 prepared with different ball-milling times, prolonging the milling time from 30 to 60 min decreases the BET surface area from

137 to 101 m^2 g^{-1} and the total pore volume from 0.18 to 0.13 cm^3 g^{-1}. This decrease indicates that excessive ball milling can damage the accessible pore structure and reduce the exposure of active sites, explaining the lower photocatalytic activity of B_7M_3-60 min. Therefore, controlling the ball-milling time is necessary to balance interfacial contact, pore accessibility, and catalytic performance. The zeta potential **(Supplementary Fig. 14)** of B_3M_7 and B_7M_3 were 2.59 and 3.34, respectively. The low zeta potential indicates that the material is negatively charged in water, due to electrostatic effects, which reduces interference from other anions while being more conducive to adsorbing positively charged pollutants.

Supplementary Note 2 | Quantify the contribution ratios of $^1O_2$ and h^+ to the degradation of BPAF

The relative contribution of $^1O_2$ to BPAF removal was evaluated quantitatively by a modified method.[22,23] $[^1O_2]_{ss}$ represents the steady-state concentration of $^1O_2$, which was calculated using eq 1.

$$[\,^1O_2]_{SS} = \frac{k'_{\text{phenol}}}{k_{\text{phenol}}} \qquad (1)$$

where k_{phenol} represents the second-order rate constants of $^1O_2$ reacting with phenol (as the probe compound of $^1O_2$, k = 2.6 × 10^6 M^{-1} s^{-1}) and k'_{phenol} represents the first-order rate constants of $^1O_2$ reacting with phenol.

$k_{^1O_2-\text{BPAF}}$ represents the second-order rate constants of $^1O_2$ reacting with BPAF, which was determined by a competitive kinetics experiment to estimate the contribution of singlet oxygen. BPA was selected as a competitor due to the fact that the second-order rate constant (k = 7.99 × 10^6 M^{-1} s^{-1}) with $^1O_2$ is comparable to that of BPAF[24]. Thus, was calculated by eq 2.

$$\ln\frac{[\text{BPAF}]}{[\text{BPAF}_0]} = \frac{k_{^1O_2-\text{BPAF}}}{k_{^1O_2-\text{BPA}}} \times \ln\frac{[\text{BPA}]}{[\text{BPA}_0]} \qquad (2)$$

When the values of $k_{^1O_2-\mathrm{BPAF}}$ and $[^1O_2]_{ss}$ were given, pseudo first-order rate constants of the reactions of BPAF with $^1O_2$ $k'_{^1O_2-\mathrm{BPAF}}$ can be calculated via eq 3.

$$k'_{^1O_2-\mathrm{BPAF}} = k_{^1O_2-\mathrm{BPAF}} \times [\,^1O_2]_{SS} \tag{3}$$

Finally, the contribution ratio of $^1O_2$ in B7M3 photocatalysis systems (η) was defined by eq 4, where k'_{BPAF} represents the pseudo first-order rate constants of BPAF in the competitive kinetics experiments.

$$\eta = \frac{k'_{^1O_2-\mathrm{BPAF}}}{k'_{\mathrm{BPAF}}} \times 100\% \tag{4}$$

Based on the aforementioned equations, the values of $[^1O_2]_{ss}$ were calculated to be 1.8×10^{-6} and 0.55×10^{-6} M for Vis and NIR light, respectively.

Supplementary Note 3 | Definitions, calculation procedures, and data sources for the parameters presented in Figure 5f

To compare the overall performance of flat-panel fixed-bed reactors, tubular fixed-bed reactors, microfluidic reactors, slurry continuous-flow reactors, batch reactors, and photocatalytic membrane reactors (PMR), data on effective working volume, treatment flow rate, residence time, catalyst dosage or immobilized catalyst mass, catalyst recovery, effective light penetration depth, and continuous operating conditions were collected from the published literature. Priority was given to studies that comprehensively reported reactor configurations and operating parameters. For each reactor type, the median values were used as representative data to minimize the influence of extreme conditions reported in individual studies.

For continuous-flow reactors, when the effective working volume was not explicitly reported, it was calculated from the treatment flow rate and hydraulic residence time.

Recovery (%) refers to the catalyst recovery rate or immobilized catalyst retention rate rather than the pollutant removal efficiency. For slurry reactors, it was calculated as the ratio of the catalyst mass recovered after the reaction to the initial catalyst mass.

For immobilized systems, including flat-panel fixed-bed, tubular fixed-bed, and photocatalytic membrane reactors, it was expressed as the ratio of the immobilized catalyst mass remaining after operation to the initial immobilized catalyst mass.

Catalyst Loading (g) denotes the total mass of catalyst actually used in the reactor. When the catalyst mass was directly provided in the literature, the reported value was adopted.

Light Penetration (cm) is defined as the characteristic distance over which light travels from the illuminated surface into the reaction medium to reach the farthest region where effective photochemical reactions can occur. For flat-panel fixed-bed reactors, it corresponds to the thickness of the liquid layer or catalyst bed; for tubular and annular reactors, the radial liquid-layer thickness along the direction of light propagation; for microfluidic reactors, the channel height; for batch reactors, the effective optical path length from the illuminated surface into the reaction solution; and for photocatalytic membrane reactors, the effective liquid-layer thickness from the membrane surface to the opposite boundary.

Scalability (arb. units) is a semi-quantitative index ranging from 0 to 10, calculated by assigning equal weights to five engineering-related criteria, each scored from 0 to 2. These criteria include continuous operation capability, modular parallelization or scale-up potential, ease of catalyst separation and recovery, complexity of reactor configuration and operational maintenance, and long-term stability and extent of pilot-scale validation.

Supplementary References

1. Liu, S. *et al.* Simultaneous regeneration of activated carbon and removal of adsorbed atrazine by ozonation process: From laboratory scale to pilot studies. *Water Res.* 121113 (2024) doi:10.1016/j.watres.2024.121113.
2. Kühne, T. D. *et al.* CP2K: an electronic structure and molecular dynamics software package - quickstep: efficient and accurate electronic structure calculations. *J. Chem. Phys.* **152**, 194103 (2020).
3. Perdew, J. P., Burke, K. & Ernzerhof, M. Generalized gradient approximation made simple. *Phys. Rev. Lett.* **77**, 3865–3868 (1996).
4. Grimme, S., Ehrlich, S. & Goerigk, L. Effect of the damping function in dispersion corrected density functional theory. *J. Comput. Chem.* **32**, 1456–1465 (2011).
5. Lu, T. & Chen, Q. Shermo: a general code for calculating molecular thermochemistry properties. *Comput. Theor. Chem.* **1200**, 113249 (2021).
6. Lu, T. A comprehensive electron wavefunction analysis toolbox for chemists, multiwfn. *J. Chem. Phys.* **161**, 82503 (2024).
7. Lu, T. & Chen, F. Multiwfn: a multifunctional wavefunction analyzer. *J. Comput. Chem.* **33**, 580–592 (2012).
8. Momma, K. & Izumi, F. VESTA 3 for three-dimensional visualization of crystal, volumetric and morphology data. *J. Appl. Crystallogr.* **44**, 1272–1276 (2011).
9. Wang, X. *et al.* Interfacial chemical bond and internal electric field modulated Z-scheme Sv-$ZnIn_2S_4/MoSe_2$ photocatalyst for efficient hydrogen evolution. *Nat. Commun.* **12**, 4112 (2021).

10. Zhou, B. *et al.* Modulating Adsorption–Redox Sites and Charge Separation of $Cs_3Bi_2Br_{9-x}$@AgBr Core–Shell Heterostructure for Selective Toluene Photooxidation. *ACS Energy Lett.* **9**, 1743–1752 (2024).

11. Mu, F. *et al.* Novel 2D/1D MXene/Bi5O7I Schottky junction for photocatalytic degradation of bisphenol AF by peroxymonosulfate-assisted photocatalysis. *Chinese Chemical Letters* **37**, 111866 (2026).

12. Ma, Y. *et al.* Photocatalytic degradation and rate constant prediction of chlorophenols and bisphenols by H3PW12O40/GR/TiO2 composite membrane. *Environ. Res.* **188**, 109786 (2020).

13. Jia, J. *et al.* Comparative study on bisphenols oxidation via TiO2 photocatalytic activation of peroxymonosulfate: Effectiveness, mechanism and pathways. *J. Hazard. Mater.* **424**, 127434 (2022).

14. Orona-Návar, C. *et al.* Gd3+ doped BiVO4 and visible light-emitting diodes (LED) for photocatalytic decomposition of bisphenol A, bisphenol S and bisphenol AF in water. *J. Environ. Chem. Eng.* **9**, 105842 (2021).

15. Jiang, R. *et al.* The bonding and intercalation efficacy of polyethyleneimine enhanced BiOBr semiconductor-organic framework degradation and detoxification of bisphenol AF. *Sep. Purif. Technol.* **320**, 124134 (2023).

16. Dai, B. *et al.* Designing S-scheme Au/g-C3N4/BiO1.2I0.6 plasmonic heterojunction for efficient visible-light photocatalysis. *Sep. Purif. Technol.* **287**, 120531 (2022).

17. Zhu, Z. *et al.* Dual-iodine-doped BiOIO3: Bulk and surface co–modification for

enhanced visible-light photocatalytic removal of bisphenol AF. *Chem. Eng. J.* **404**, 126543 (2021).

18. Perrin, D. D. Stability of Metal Complexes with Salicylic Acid and Related Substances. *Nature* **182**, 741–742 (1958).
19. Jiang, Q., Shen, J. & Zhong, G. Synthesis of bismuth(III) complexes and coordination chemistry of bismuth(III). *Prog. Chem.* **18**, 1634–1645 (2006).
20. Miller, W. T. Some Complex Sodium Bismuth Salts of Triethanolamine and Triisopropanolamine. *J. Am. Chem. Soc.* **62**, 2707–2709 (1940).
21. Whitmire, K. H. Bismuth: Inorganic Chemistry. in *Encyclopedia of Inorganic and Bioinorganic Chemistry* 1–32 (John Wiley & Sons, Ltd, 2014). doi:10.1002/9781119951438.eibc0018.pub2.
22. Shao, P. *et al.* Revisiting the Graphitized Nanodiamond-Mediated Activation of Peroxymonosulfate: Singlet Oxygenation versus Electron Transfer. *Environ. Sci. Technol.* **55**, 16078–16087 (2021).
23. Dou, J. *et al.* Neglected but Efficient Electron Utilization Driven by Biochar-Coactivated Phenols and Peroxydisulfate: Polyphenol Accumulation Rather than Mineralization. *Environ. Sci. Technol.* **57**, 5703–5713 (2023).
24. Xie, Z.-H. *et al.* Review of characteristics, generation pathways and detection methods of singlet oxygen generated in advanced oxidation processes (AOPs). *Chem. Eng. J.* **468**, 143778 (2023).
