# Supplementary material for: Near-infrared-active Nd–BiO2-x/MIL-101(Fe) with vacancy-induced interfaces for solar water purification
Source: Nat Commun. 2026 Aug 26;17:10237. doi: 10.1038/s41467-026-77239-z (PMC13616951; doi:10.1038/s41467-026-77239-z)
Supplement: Supplementary file 2 [file 41467_2026_77239_MOESM2_ESM.pdf]

Description of Additional Supplementary Files

Supplementary Data 1 : Atomic coordinates of the optimized structural models. This file contains the atomic coordinates of the geometry-optimized models. The "O_V-B_xM_y" sheet contains the BiO_{2-x}/MIL-101(Fe) interface model with oxygen vacancies; "B_xM_y" contains the corresponding interface model without oxygen vacancies; "BiO_2" contains the metal oxide model without oxygen vacancies; "BiO_{2-x}" contains the metal oxide model with oxygen vacancies; and "Nd-BiO_{2-x}" contains the Nd-doped metal oxide model with oxygen vacancies.
