# Supplementary material for: Near-infrared-active Nd–BiO2-x/MIL-101(Fe) with vacancy-induced interfaces for solar water purification
Source: Nat Commun. 2026 Aug 26;17:10237. doi: 10.1038/s41467-026-77239-z (PMC13616951; doi:10.1038/s41467-026-77239-z)
Supplement: Supplementary file 4 [file 41467_2026_77239_MOESM5_ESM.pdf]

nature portfolio

Peer Review File

Near-Infrared-Active Nd–BiO2-x/MIL-101(Fe) with Vacancy-Induced Interfaces for Solar Water Purification

Corresponding Author: Dr Huayang Zhang

This file contains all reviewer reports in order by version, followed by all author rebuttals in order by version.

Version 0:

Reviewer comments:

Reviewer #1

(Remarks to the Author)
This research innovatively observed the inducing effect of oxygen vacancies on interface bonding, constructs a direct Z-scheme Nd–BiO2-x/MIL-101(Fe) heterojunction with Bi–O=C interfacial bonds and an atomic-scale built-in electric field. By utilizing interface bonding, two semiconductors with differentiated Fermi levels can rapidly transfer photogenerated carriers during the illumination process. The outstanding contribution of this research lies in the expansion of the absorption edge into the near-infrared region, which enables the full utilization of sunlight. The characterization of interface bonding and heterojunctions is quite comprehensive. Finally, a continuous-flow device was set up to degrade organic pollutants in the water effectively. It is suggested to enrich the following statements with a minor revision.
1. It is suggested that in the introduction, additional references to other studies regarding the research status and relevant literature that did not consider interface bonding should be provided, in order to highlight the originality of this study.
2. In the XAS characterization, Section 2.1, it is recommended to appropriately supplement further descriptions of the Fe element in the main text and the appendix, and enrich the relevant conclusions regarding bonding.
3. The FTIR characterization diagrams in the main text have relatively few annotations, which makes it difficult for readers to understand and observe the shifts in peaks and the movements of positions. The movement of positions is an important piece of evidence for bond formation.
4. The experimental methods should further elaborate on some related characterization parameters and device fabrication procedures, such as the catalyst loading step in the continuous flow device.
5. Some texts require further refinement. On page 4, line 75, "asymmetric local structure" is missing the "an" before it. On page 11, line 276, "prolonged" should be changed to "prolonging". In line 278, "pore structure" is missing the word "the" before it.
6. It is suggested to add a comparison chart of reaction rate coefficients with other literature to highlight the effectiveness of this study.

Reviewer #2

(Remarks to the Author)
Shan et al. investigated the challenge of effectively utilizing NIR light in photocatalytic water purification. By integrating emerging MOF materials with inorganic oxides, they constructed a heterojunction system to improve photogenerated carrier migration. This enables enhanced NIR absorption and utilization, promotes the generation of reactive oxygen species, and facilitates hole-driven oxidation processes. The manuscript is generally well organized, supported by sufficient experimental data, and presents an interesting attempt toward scalable applications. In my opinion, the work may offer useful insights for photocatalysis and NIR-related photothermal photocatalytic systems. Several issues should be addressed before the manuscript can be considered for publication. If the following concerns are properly resolved, the work could make a meaningful contribution to photocatalysis and practical water purification.

1. The core innovation of this article lies in the utilization of near-infrared and the construction of the Z-scheme. To further highlight the originality of the article, the relevant research background of NIR and direct Z-scheme photocatalyst needs to be supplemented in the INTRODUCTION.

2. To further verify the electron transport path of the Z heterojunction and demonstrate the excellent migration of

photogenerated carriers, the authors were recommended to provide additional experimental evidence, such as in situ XPS measurements under dark and illuminated conditions.

3. The role of highlighting Nd and Ov on electron enrichment needs to be analyzed in DFT calculations and XPS. In particular, the mechanism by which Ov contributes to electron accumulation is crucial for understanding O2 activation.

4. Details regarding the assembly of the continuous flow device and the preparation of the catalyst coating in Figure 5 should be added to the Methods description.

5. The detection methods for pollutants and the intermediate products should be described in detail with specific experimental procedures.

6. The analysis of the final intermediate products and related toxicological changes is relatively brief in the text. Such analysis should be conducted in greater detail to highlight the practical advantages of the application.

7. The comparison between simulated light intensity in laboratory conditions and actual solar irradiation should be further clarified to improve the relevance to realistic applications.

8. p.14, "the carbonyl and hydroxyl bands of the carboxyl groups in phthalic acid weaken and shift to lower wavenumbers, indicating outward electron transfer to BiO2-x through Bi–O=C interfacial bonds." It should be clearly stated that the electrons of the carboxyl group are being transferred.

9. Further miscellaneous comments:
* Provide the fitting model and corresponding equations used for the TRPL analysis. This would improve the clarity and reproducibility of the carrier lifetime interpretation.

* In Figure 1k, the FT-EXAFS should indicate the positions of the characteristic peaks of Fe-O and Fe-Fe.

* Some sentences contain grammatical mistakes, such as on page 3, in the INTRODUCTION, “limited” should be “limit”. On page 5, section 2.1, “confirm” should be “confirms”. On page 15, section 2.5, “strong capability” should be “a strong capability”, and “andmixed” should be “and mixed”.

* For the “?” mark in Figure 2, no explanation was provided in the original text. It is suggested to add relevant descriptions to facilitate the subsequent discussion.

Reviewer #3

(Remarks to the Author)
The author prepared an Nd-BiO2-x/MIL-101(Fe) photocatalyst and proposed an Ov-induced direct Z-scheme heterojunction. The heterojunction material exhibited excellent degradation performance for both BPAF and RhB. Combined with DFT calculations and a series of advanced characterizations, the possible reaction mechanism for photocatalytic pollutant degradation was inferred. Furthermore, tests under natural sunlight demonstrated efficient removal of 11 pollutants from reservoir water, highlighting the system’s potential. However, the following issues should be addressed:
1.In the high-resolution TEM image of Figure 1c, the lattice fringes of MIL-101(Fe) cannot be observed. How can the construction of the heterojunction be proven?
2.KPFM measurements before and after illumination should be further confirmed. Under dark conditions, the potential in regions associated with MIL-101(Fe) is lower than that of BiO2-x, indicating that MIL-101(Fe) has a higher work function. This suggests that under dark conditions, BiO2-x should serve as the electron donor, rather than MIL-101(Fe).
3.In the charge transfer diagram of Figure 2d, the work function of MIL-101(Fe) should be checked and corrected.
4.The authors used XPS spectra of Bi 4f of the as-prepared samples to show an increase in the binding energy of Bi, inferring that BiO2-x can provide electrons to MIL-101(Fe). It is recommended to supplement the XPS data of Fe for MIL-101(Fe) and the heterojunction material to further directly demonstrate the charge transfer direction.
5.How can the statement “MIL-101(Fe) is highly porous with a large void structure” be justified? It is recommended to supplement the pore size distribution of MIL-101(Fe) to illustrate its microporous and mesoporous distribution.
6.In the BET test (Figure S11 and Table S2), it is recommended to supplement the data for MIL-101(Fe).
7.The adsorption and photocatalytic effects of MIL-101(Fe) on BPAF and RhB should be supplemented in the activity tests.
8.The photocatalytic performance of the BxMy series materials on RhB under visible light and near-infrared light should be supplemented.
9.What are the cycling stability and reusability of the heterojunction material for the removal of BPAF and RhB?
10.In the activity tests, it was found that changing the ball-milling time during the preparation of B7M3 affects its photocatalytic activity. The authors stated that this “highlights the necessity of controlling the milling time to protect the pore structure”. It is recommended to supplement the specific surface area and pore structure test data of B7M3 under different milling times (20-60 min).
11.It is recommended to supplement the effects of quenching agents on the removal of RhB by the photocatalyst under visible and near-infrared light.
12.It is recommended to supplement the contribution ratios of different reactive oxygen species to the removal of BPAF and RhB by the photocatalyst, in order to visually indicate the main active species responsible for pollutant removal.
13.The introduction of Nd, the presence of oxygen vacancies, and the construction of the heterojunction all affect the

generation of ROS. It is recommended to monitor the concentrations of generated ROSs (·OH, 1O2, ·O2-, H2O2), which would help quantitatively understand the enhancement of photocatalytic activity of B7M3.
14.What is the role of Nd? Does Nd exist in the material as ions or as nanoparticles? If it exists as nanoparticles, is there evidence from XRD, HR-TEM, and XPS?
15.The authors introduced a series of metal chelators to evaluate the roles of metal active sites (Fe ions and Bi ions). In what form does Nd exist in the heterojunction material? Can Nd also serve as a metal active site?
16.When investigating changes in metal active sites, h+, and oxidation potential, only the photocatalytic reaction of the material with BPAF was considered. What about the reaction of the material with RhB?
17.Why did the authors choose BPAF and RhB as two typical water pollutants?
18.The authors used DFT calculations to explore the charge transfer process of the heterojunction, highlighting the role of Ov. What exactly is the role of Nd? How should it be explained?
19.The authors demonstrated the formation of Bi-O=C interfacial bonds through the charge density difference diagram. It is recommended to supplement the electron localization functon(ELF) to visually determine whether electrons are transferred from one atom to another.
20.Was dipole correction considered when calculating the work function?
21.Should the reaction mechanism for the photocatalytic removal of RhB by the heterojunction material also be explained?
22.Format issues in the manuscript should be further checked and corrected (e.g., the font and format of figures and tables should be unified; subscripts and superscripts should be carefully reviewed).

Version 1:

Reviewer comments:

Reviewer #1

(Remarks to the Author)
The authors have carefully revised the manuscript according to the comments and I think the manuscript could be accepted for publication.

Reviewer #2

(Remarks to the Author)
The authors have adequately addressed my concerns, and I have no further comments.

Reviewer #3

(Remarks to the Author)
The author has revised the content as required. Recommend acceptance.

Open Access This Peer Review File is licensed under a Creative Commons Attribution 4.0 International License, which permits use, sharing, adaptation, distribution and reproduction in any medium or format, as long as you give appropriate credit to the original author(s) and the source, provide a link to the Creative Commons license, and indicate if changes were

made.
In cases where reviewers are anonymous, credit should be given to 'Anonymous Referee' and the source.
The images or other third party material in this Peer Review File are included in the article’s Creative Commons license, unless indicated otherwise in a credit line to the material. If material is not included in the article’s Creative Commons license and your intended use is not permitted by statutory regulation or exceeds the permitted use, you will need to obtain permission directly from the copyright holder.
To view a copy of this license, visit https://creativecommons.org/licenses/by/4.0/

Manuscript ID: NCOMMS-26-023685

Title: Near-Infrared-Active Nd–BiO_{2-x}/MIL-101(Fe) with Vacancy-Induced Interfaces for Solar Water Purification

RESPONSE TO REVIEWERS

Response to Reviewer #1

Reviewer's general comments

This research innovatively observed the inducing effect of oxygen vacancies on interface bonding, constructs a direct Z-scheme Nd-BiO_{2-x}/MIL-101(Fe) heterojunction with Bi-O=C interfacial bonds and an atomic-scale built-in electric field. By utilizing interface bonding, two semiconductors with differentiated Fermi levels can rapidly transfer photogenerated carriers during the illumination process. The outstanding contribution of this research lies in the expansion of the absorption edge into the near-infrared region, which enables the full utilization of sunlight. The characterization of interface bonding and heterojunctions is quite comprehensive. Finally, a continuous-flow device was set up to degrade organic pollutants in the water effectively. It is suggested to enrich the following statements with a minor revision.

Response to comments:

We sincerely thank reviewer #1 for the constructive and encouraging comments. We have carefully considered all suggestions and revised the manuscript accordingly. Each comment has been addressed point by point, and the corresponding changes are highlighted in the revised manuscript.

Comment 1-1: It is suggested that in the introduction, additional references to other studies regarding the research status and relevant literature that did not consider interface bonding should be provided, in order to highlight the originality of this study.

Response: We thank the reviewer for pointing out this suggestion. In response, we have added a discussion of interface bonding in the third paragraph of the introduction and cited additional relevant studies to better clarify the research gap and originality of this work.

Changes: *On pages 3 and 4, the description of relevant literature of interface bonding is as follows:*

"The role of O_v in forming stable chemical bonds at heterojunction interfaces, and in generating an intrinsic built-in electric field (IEF) to direct charge separation, has been largely overlooked[17,18]. Conventional heterojunctions are mainly held together by weak physical interactions, such as van der Waals forces or electrostatic attraction, which can limit interfacial electron coupling because of lattice mismatch and poor orbital

overlap[19,20]. Current studies on Z-direct heterojunctions mainly rely on XPS evidence of chemical bonds and analysis of photogenerated carriers' migration pathway [21,22]. However, the atomic-level connection between vacancy-induced interfacial bonding, charge redistribution, and IEF formation remains insufficiently understood. In contrast, O_v can induce the coordination of oxygen-containing functional groups, leading to the formation of robust interfacial chemical bonds with stronger electron interactions. Electron redistribution across these bonds creates depletion or accumulation regions and induces band bending at the interface. This atomic-scale interfacial channel establishes an IEF that drives directional charge migration, suppresses charge recombination, and enhances photocatalytic performance [23,24]. Therefore, O_v-induced interfacial bonding can improve both structural stability and charge separation, providing a rational strategy for constructing efficient photocatalytic systems."

Reference:

19. Wu, X. *et al.* Modulating g-C_3N_4-based van der Waals heterostructures with spatially separated reductive centers for tandem photocatalytic CO_2 methanation. *Appl. Catal., B* **330**, 122666 (2023).

20. Hu, J. *et al.* Lattice match-enabled covalent heterointerfaces with built-in electric field for efficient hydrogen peroxide photosynthesis. *Adv. Mater.* **36**, 2412070 (2024).

21. Lin, L. *et al.* Efficient and stable visible-light-driven Z-scheme overall water splitting using an oxysulfide H_2 evolution photocatalyst. *Nat. Commun.* **15**, 397 (2024).

22. Zhao, D. *et al.* Boron-doped nitrogen-deficient carbon nitride-based Z-scheme heterostructures for photocatalytic overall water splitting. *Nat. Energy* **6**, 388–397 (2021).

23. Wang, X. *et al.* Conformal and conductive biofilm-bridged artificial Z-scheme system for visible light–driven overall water splitting. *Sci. Adv.* **10**, eadn6211 (2024).

24. Zhou, E. *et al.* Ultrathin covalent organic framework nanosheets for enhanced photocatalytic water oxidation. *Sci. Adv.* **10**, eadk8564 (2024).

Comment 1-2: In the XAS characterization, Section 2.1, it is recommended to appropriately supplement further descriptions of the Fe element in the main text and the appendix, and enrich the relevant conclusions regarding bonding.

Response: We deeply appreciate your professional comments. Due to length constraints, the discussion of the Fe-related XAS results was not sufficiently detailed in the previous version. We have now expanded this part in the revised manuscript to provide a clearer analysis of the Fe coordination environment and its relationship with interfacial bonding.

Changes: *On page 8, the description of the Fe element of XAS characterization is as follows:*

"The fitted Fe-O bond length in MIL-101(Fe) are 1.90 and 2.04 Å, with a coordination number of 3. The Fe-Fe bond lengths are 2.92 and 3.35 Å with fitted coordination number of 1.0 and 1.1, respectively. Compared with B_7M_3 (Fe-Fe, 3.01 Å), the presence of a second Fe-Fe shell in MIL-101(Fe) indicates the retention of a more intact intrinsic crystalline structure and higher long-range order **(Figure 1j and Figure 1k)**. In B_7M_3, O=C coordinate with Bi atoms from bismuth oxides through O_v-induced interfacial bonding, introducing defects and local disorder, which disrupts the long-range Fe-Fe coordination structure, so that only one Fe-Fe shell is resolved during fitting. Wavelet transform **(Figure 1l and Figure 1m)** shows that BiO_{2-x} is mainly coordinated by Bi–O and Bi–O_v, whereas B_7M_3 forms a new Bi–O=C signal due to O_v-induced carbonyl and carboxyl bonding."

Comment 1-3: The FTIR characterization diagrams in the main text have relatively few annotations, which makes it difficult for readers to understand and observe the shifts in peaks and the movements of positions. The movement of positions is an important piece of evidence for bond formation.

Response: We thank the reviewer for this valuable suggestion. We have revised the FTIR spectra and k^2-weighted FT-EXAFS spectra by adding clear annotations for the assigned chemical bonds. In the FTIR spectra, paired dashed lines with the same color are used to indicate the redshift or blueshift of the corresponding peaks, allowing the peak position changes related to interfacial bond formation to be more clearly observed.

Changes:

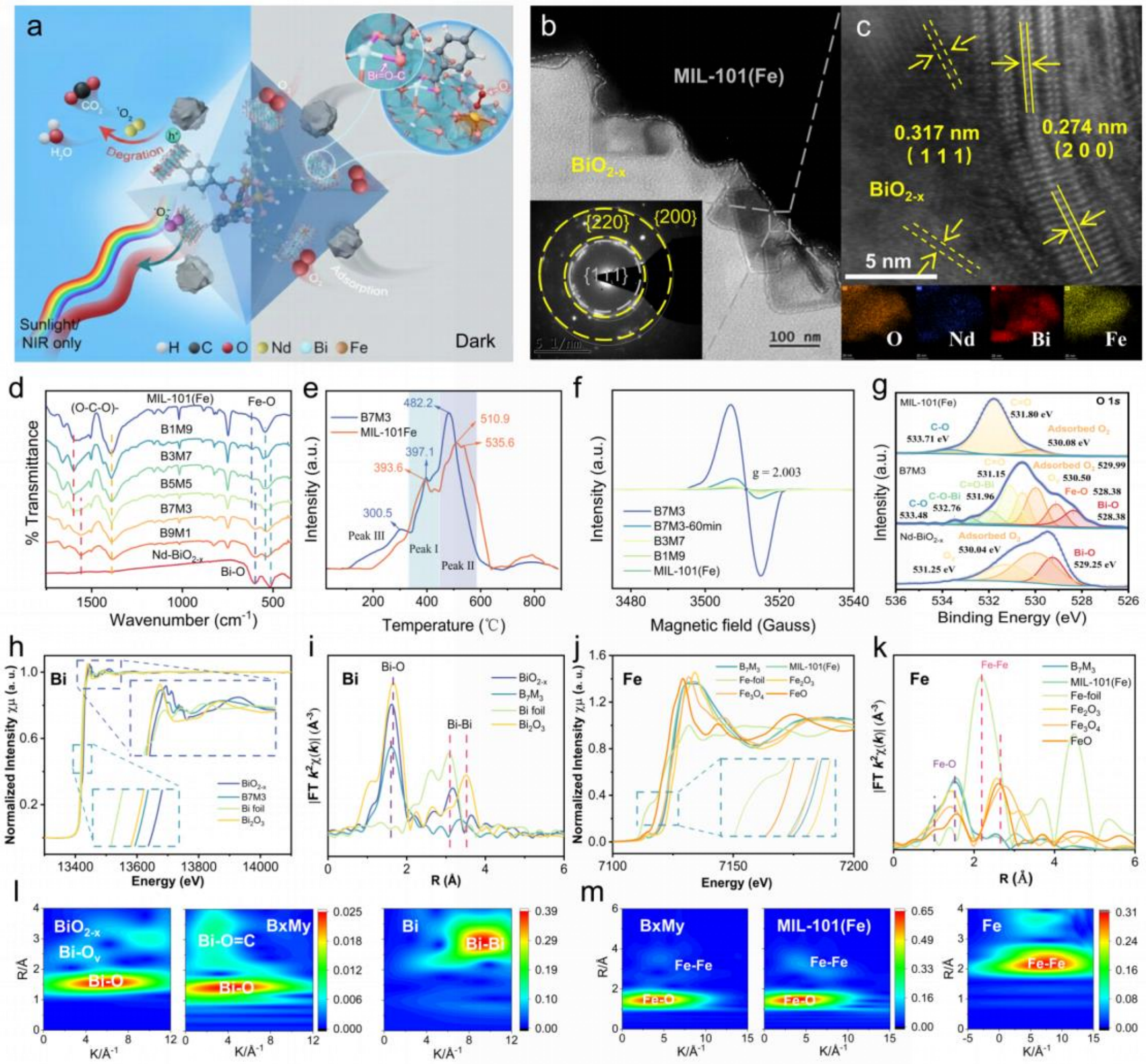

Figure 1 | Synthesis and characterization of the B_xM_y. (a) Schematic illustration of the adsorption-catalysis synergy of B_xM_y heterojunctions**. (b)** TEM image (insert: electron diffraction), (c) Aberration-Corrected-TEM and EDS elemental mapping of the B_7M_3. **(d)** FTIR spectra of Nd-BiO_{2-x}, MIL-101(Fe), and B_xM_y heterojunctions, where two dashed lines indicate peak shifts. **(e)** H_2-TPR, **(f)** EPR spectra, **(g)** XPS spectra of O 1*s*, **(h)** Bi L_3-edge and **(j)** Fe K-edge normalized intensity in XANES spectra, **(i)** and **(k)** k^2-weighted FT-EXAFS spectra, **(l)** and **(m)** wavelet transform analysis of the as-prepared samples.

Comment 1-4: The experimental methods should further elaborate on some related characterization parameters and device fabrication procedures, such as the catalyst loading step in the continuous flow device.

Response: We sincerely thank the reviewer for this helpful suggestion, and we accept it. Accordingly, we have revised the Experimental Methods and Supplementary Information to provide more detailed descriptions of the relevant characterization conditions, device assembly procedure, and catalyst loading process in the continuous-flow system.

Changes: *On pages 21, the description of the related characterization parameters and device fabrication procedures is as follows:*

Methods 4.4

In the continuous-flow sunlight setup, the reservoir was made of acrylic, and the angle-adjustable frame was constructed from stainless steel. The top surface was covered with a 3 mm-thick ultra-clear glass with a light transmittance exceeding 90%. 10 g of the catalyst was loaded onto a 1 cm-thick glass fibre mat via the dry impregnation method. The catalyst-loaded mat was then fixed onto the acrylic backplate with silicone sealant.

Comment 1-5: Some texts require further refinement. On page 4, line 75, "asymmetric local structure" is missing the "an" before it. On page 11, line 276, "prolonged" should be changed to "prolonging". In line 278, "pore structure" is missing the word "the" before it.

Response: We thank the reviewer for carefully checking the manuscript. We have corrected these errors and revised the details accordingly.

Changes:

"Doping metal ions, including Ni^{3+} and Nd^{3+}, adjacent to O_v introduces an asymmetric local structure and redistributes the electron density around the vacancies, further strengthening O_2 adsorption and activation[13,14,16]."

"The strongest vacancy signal is observed for B_7M_3 after 30 min of ball milling, whereas prolonging the milling to 60 min weakens the signal."

"Prolonging the ball milling to 60 min sharply reduces activity, with BPAF removal limited to 11% under visible light and 8% under NIR irradiation, highlighting the need to control the milling time to preserve the pore structure."

Comment 1-6: It is suggested to add a comparison chart of reaction rate coefficients with other literature to highlight the effectiveness of this study.

Response: We sincerely thank the reviewer for this valuable suggestion. We have added a comparison of the reaction rate constants for BPAF degradation using B_7M_3 and 8 previously reported catalysts. As shown in **Figure S23** and **Table S6**, B_7M_3 exhibits the highest catalyst-dose-normalized rate constant, k (L min^{-1} g^{-1}), under xenon-lamp irradiation, demonstrating the high efficiency of the present system.

Changes: *Supplementary Materials, on pages 24 and 37, the figure and table of compared k are as follows:*

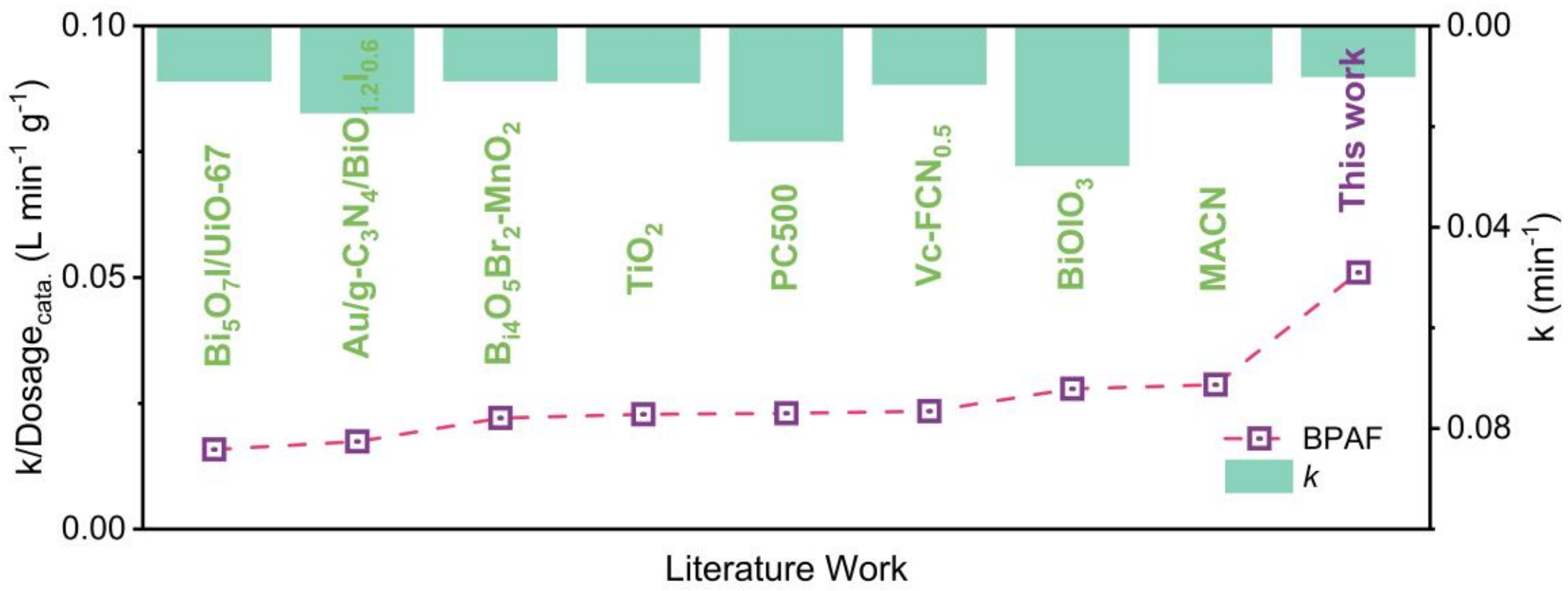

Supplementary Fig.23 | Comparisons of catalytic efficiency with previously reported catalysts. The detailed literature comparison is provided in Table S6.

Supplementary Table 6 | Comparison of catalytic efficiency for BPAF degradation over B_7M_3 and previously reported catalysts under xenon-lamp irradiation.

Photocatalysis	k (L min^{-1} g^{-1})	k_{obs} (min^{-1})	Dosage (g)
TiO_2NTAs	0.00274	0.00274	1
Gd-$BiVO_4$	0.00778	0.00778	1
BiOBr	0.00918	0.00459	0.5
Au/g-C_3N_4/$BiO_{1.2}I_{0.6}$	0.0174	0.0174	1
$BiOIO_3$	0.02787	0.02787	1
8-MXene/Bi_5O_7I/PMS	0.04973	0.02984	0.6
HPW/GR/TiO_2	0.066	0.0033	0.05

Response to Reviewer #2

Reviewer's general comments

Shan et al. investigated the challenge of effectively utilizing NIR light in photocatalytic water purification. By integrating emerging MOF materials with inorganic oxides, they constructed a heterojunction system to improve photogenerated carrier migration. This enables enhanced NIR absorption and utilization, promotes the generation of reactive oxygen species, and facilitates hole-driven oxidation processes. The manuscript is generally well organized, supported by sufficient experimental data, and presents an interesting attempt toward scalable applications. In my opinion, the work may offer useful insights for photocatalysis and NIR-related photothermal photocatalytic systems. Several issues should be addressed before the manuscript can be considered for publication. If the following concerns are properly resolved, the work could make a meaningful contribution to photocatalysis and practical water purification.

Response to comments:

We sincerely thank reviewer #2 for the positive and encouraging comments on our manuscripts. We also appreciate the reviewer's recognition of the practical relevance of solar-driven continuous-flow photocatalysis, and the use of near-infrared light for water purification. In response to the reviewer's suggestions regarding practical implementation, especially the long-term stability, we have added further experimental evidence and discussion in the revised manuscript.

Comment 2-1: The core innovation of this article lies in the utilization of near-infrared and the construction of the Z-scheme. To further highlight the originality of the article, the relevant research background of NIR and direct Z-scheme photocatalyst needs to be supplemented in the INTRODUCTION.

Response: We thank the reviewer for this suggestion. In response, we have expanded the discussion of near-infrared light use and direct Z-scheme heterojunction construction in the background.

Changes: *On pages 3 and 4, the description of relevant literature of interface bonding is as follows:*

"Solar full-spectrum-driven catalytic degradation of aquatic micropollutants has attracted increasing attention owing to its sustainable nature[1–3]. Compared with conventional ultraviolet (UV) photocatalysts, such as TiO_2[4,5], bismuth-based semiconductors possess narrower bandgaps, enabling UV-visible (Vis) light absorption, low toxicity, and favorable Bi 6s electronic features[6,7]. Current strategies for extending the light absorption edge mainly rely on ion doping and heterojunction construction, but effective methods for regulating near-infrared (NIR) light response remain limited.

Introducing oxygen vacancies (O_V) through low-valence metal doping or alkaline hydrothermal treatment further extends light absorption into the near-infrared region (NIR), allowing broader solar utilization. However, the narrowed bandgap accelerates rapid recombination of photoexcited electrons (e^-) and holes (h^+)[8], while lamellar stacking reduces active site exposure and exacerbates photocorrosion, thereby limiting photocatalytic activity[9]. Existing heterojunction designs mainly focus on the role of O_V in O_2 activation, whereas their ability to induce interfacial chemical bonding is rarely considered. In practical systems, additional challenges include reliance on artificial light sources, limited generation and use of reactive oxygen species , and poor selectivity toward target pollutants in complex water matrices[10]. Hence, integrated strategies such as O_V engineering, direct Z-scheme heterojunction construction, and adsorption-catalysis synergy are vital for overcoming these limitations[11–13]."

"The role of O_V in forming stable chemical bonds at heterojunction interfaces, and in generating an intrinsic built-in electric field (IEF) to direct charge separation, has been largely overlooked[17,18]. Conventional heterojunctions are mainly held together by weak physical interactions, such as van der Waals forces or electrostatic attraction, which can limit interfacial electron coupling because of lattice mismatch and poor orbital overlap[19,20]. Current studies on Z-direct heterojunctions mainly rely on XPS evidence of chemical bonds and analysis of photogenerated carriers' migration pathway [21,22]. However, the atomic-level connection between vacancy-induced interfacial bonding, charge redistribution, and IEF formation remains insufficiently understood. In contrast, O_V can induce the coordination of oxygen-containing functional groups, leading to the formation of robust interfacial chemical bonds with stronger electron interactions. Electron redistribution across these bonds creates depletion or accumulation regions and induces band bending at the interface. This atomic-scale interfacial channel establishes an IEF that drives directional charge migration, suppresses charge recombination, and enhances photocatalytic performance [23,24]. Therefore, O_V-induced interfacial bonding can improve both structural stability and charge separation, providing a rational strategy for constructing efficient photocatalytic systems."

Comment 2-2: To further verify the electron transport path of the Z heterojunction and demonstrate the excellent migration of photogenerated carriers, the authors were recommended to provide additional experimental evidence, such as in situ XPS measurements under dark and illuminated conditions.

Response: We sincerely thank the reviewer for this valuable suggestion. To further clarify the photogenerated charge transfer pathway and verify the direct Z-scheme heterojunction mechanism, we have added in situ XPS measurements under dark and illuminated conditions in the revised manuscript

Changes: *On pages 10 and 11, the Figure 2 and the description have been revised as follows:*

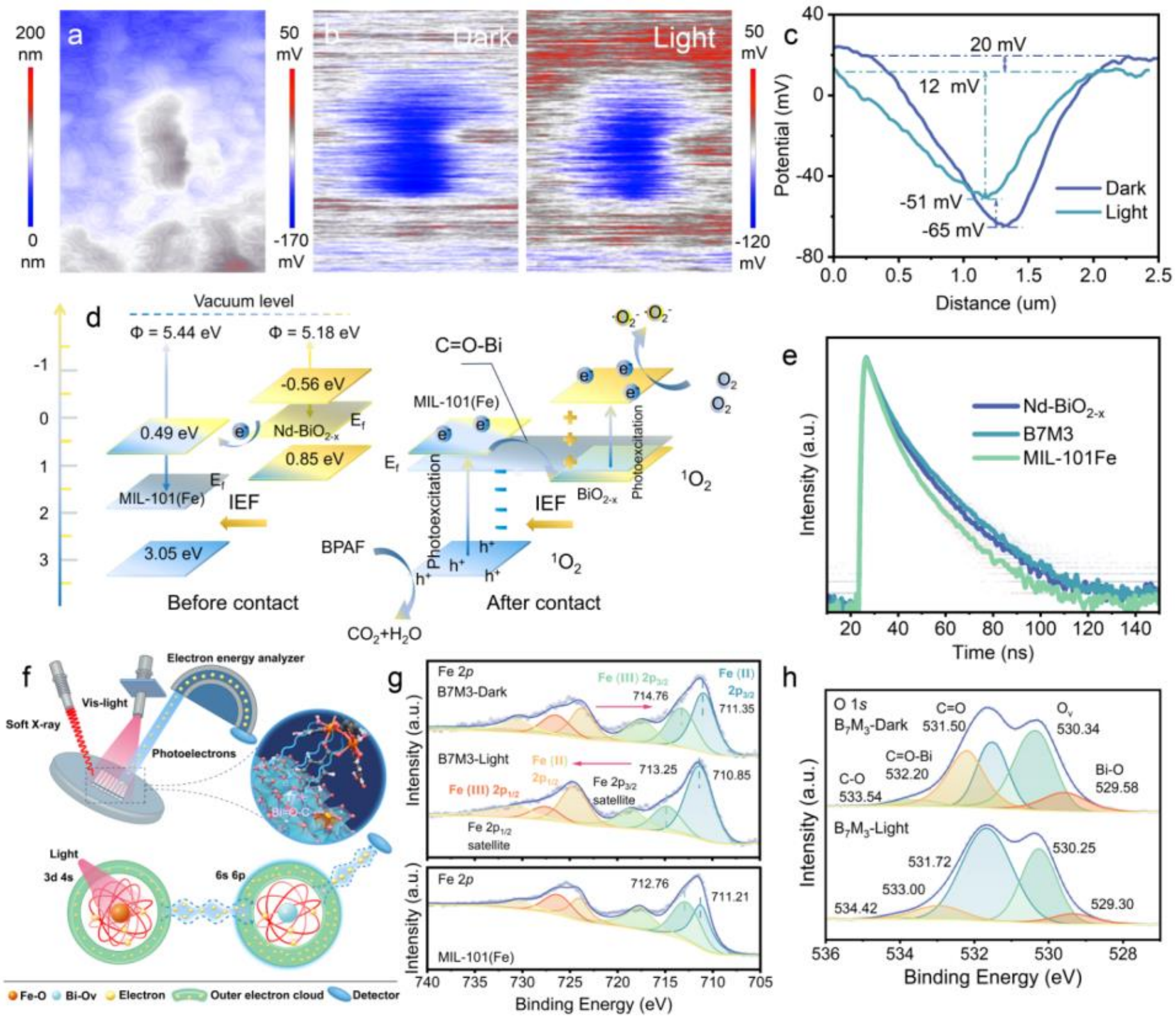

Figure 2 | Electron transfer pathway and photophysical and Electrochemical measurements. (a-c) KPFM images and corresponding surface potential distribution of B_xM_y heterojunctions in darkness and under light. **(d)** Schematic illustration of the Nd-BiO_{2-x}/MIL-101(Fe) direct Z-scheme heterojunction with an interfacial electric field. **(e)** Transient photocurrent response densities of as-prepared samples. **(f-h)** Schematic illustration of the in situ XPS setup and high−resolution Fe 2*p* and O 1*s* spectra before and after light.

On page 11:

"To gain deeper insights into the interfacial charge transfer mechanism within the B_xM_y heterojunction, comparative in situ XPS analyses were conducted under dark and illuminated conditions **(Figure 2f-h)**. The Fe 2*p* spectra show clear electronic changes around Fe sites after coupling with Nd-BiO_{2-x}, supporting interfacial electron redistribution between the two components. Under light irradiation, the binding energies of the Fe 2*p* peaks and the O 1*s* peaks assigned to C=O and C–O bonds shift positively, whereas the O 1*s* peak assigned to Bi–O shift negatively compared with those in the dark state. These opposite binding energy shifts reveal photoinduced charge redistribution across the heterojunction. Specifically, photogenerated electrons accumulate on Nd-BiO_{2-x}, while holes remain on MIL-101(Fe), consistent with the direct Z-scheme charge transfer pathway driven by the interfacial electric field."

Comment 2-3: The role of highlighting Nd and Ov on electron enrichment needs to be analyzed in DFT calculations and XPS. In particular, the mechanism by which O_v contributes to electron accumulation is crucial for understanding O_2 activation.

Response: We sincerely thank the reviewer for this valuable suggestion. To clarify the roles of Nd and O_v in electron enrichment and O_2 activation, we have added further analysis based on in situ illuminated XPS and DFT calculations. We deduced the migration of photogenerated charge carriers toward Bi-O_v within the Z-scheme heterojunction. Additionally, DFT calculations verified that the presence of Nd facilitates the adsorption and activation of O_2 around O_v sites.

Changes: *On pages 9 and 10, the description of photogenerated charge carriers toward Bi-Ov within the Z-scheme heterojunction has been revised as follows:*

"In n-type semiconductor photocatalysts, electrons are the dominant charge carriers and play a central role in catalytic reactions. The energy level alignment between Nd-BiO_{2-x} and MIL-101(Fe) is schematically shown in **Figure 2d**. Density functional theory calculations with dipole corrections were used to determine the work functions of the two components **(Figure S18)**. The calculated work function of MIL-101(Fe) is 5.44 eV, which is higher than that of Nd-BiO_{2-x} (5.18 eV), indicating that electrons migrate from Nd-BiO_{2-x} to MIL-101(Fe) upon contact to equilibrate their Fermi levels[39,40]. As a result, Nd-BiO_{2-x} becomes positively charged, while MIL-101(Fe) becomes negatively charged, establishing a built-in electric field at the interface. Under illumination, this built-in electric field facilitates directional charge migration and spatial separation of photogenerated carriers, driving the direct Z-scheme pathway in which electrons accumulate on Nd-BiO_{2-x} and holes remain on MIL-101(Fe)[41]. In addition, O_v-associated undercoordinated Bi/Nd sites introduce localized defect states and act as electron-accumulation centers on the Nd-BiO_{2-x} surface, facilitating subsequent redox reactions[11–13]."

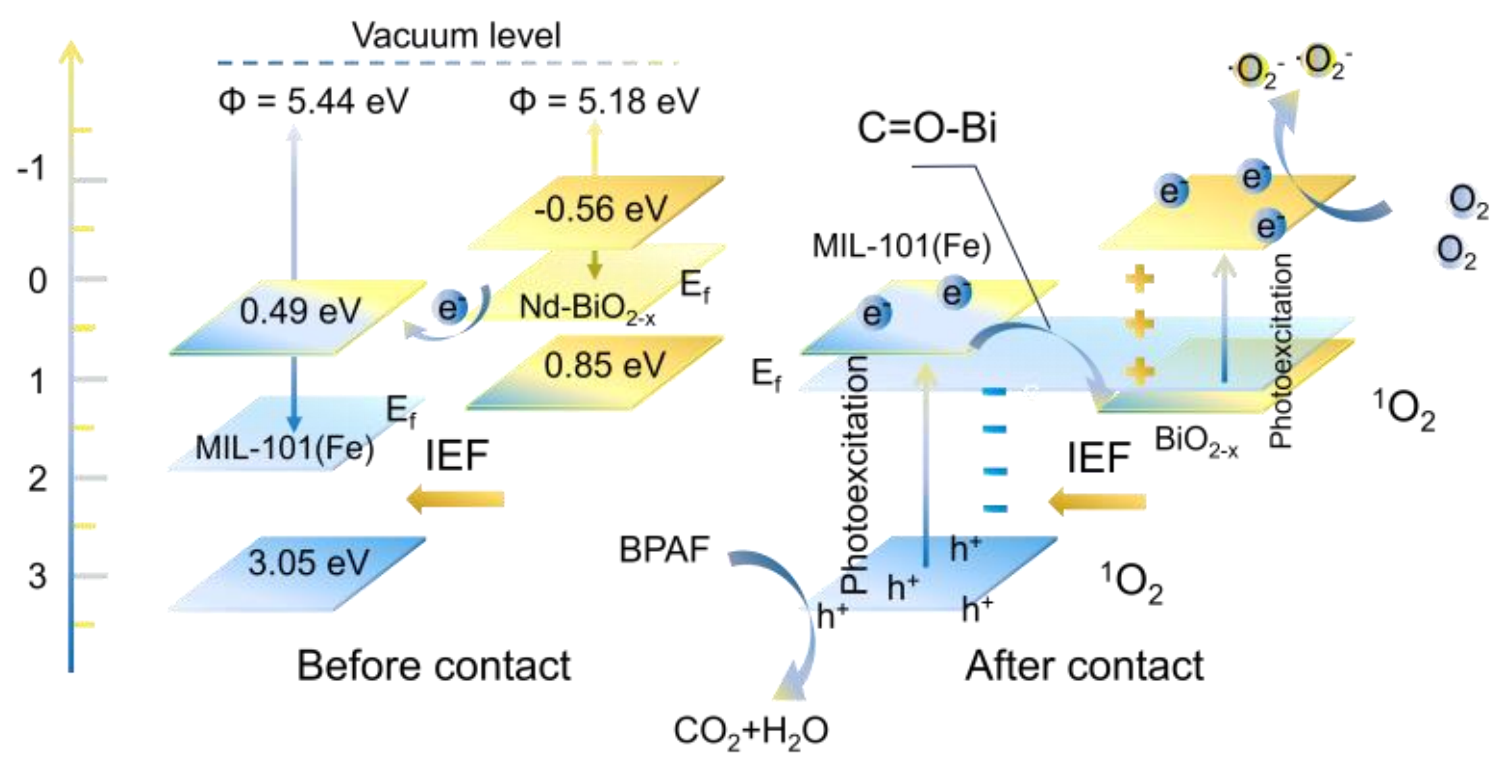

Figure 2 | (d) Schematic illustration of the Nd-BiO_{2-x}/MIL-101(Fe) direct Z-scheme heterojunction with an interfacial electric field.

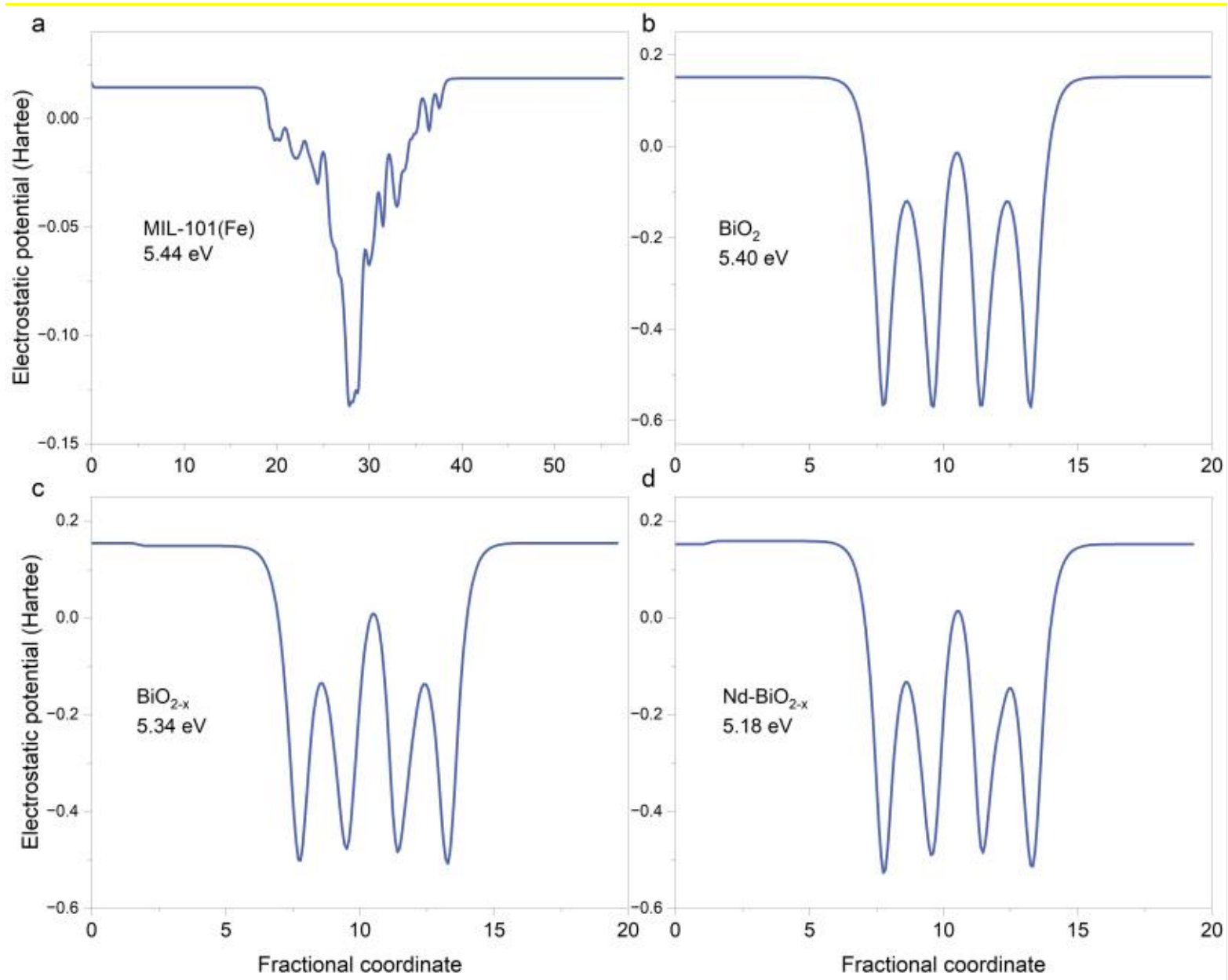

Supplementary Fig.18 | Electrostatic potentials of BiO_2, BiO_{2-x}, Nd-BiO_{2-x}, and MIL-101(Fe).

On page 11:

"To gain deeper insights into the interfacial charge transfer mechanism within the B_xM_y heterojunction, comparative in situ XPS analyses were conducted under dark and illuminated conditions **(Figure 2f-h)**. The Fe 2*p* spectra show clear electronic changes around Fe sites after coupling with Nd-BiO_{2-x}, supporting interfacial electron redistribution between the two components. Under light irradiation, the binding energies of the Fe 2*p* peaks and the O 1*s* peaks assigned to C=O and C–O bonds shift positively, whereas the O 1*s* peak assigned to Bi–O shift negatively compared with those in the dark state. These opposite binding energy shifts reveal photoinduced charge redistribution across the heterojunction. Specifically, photogenerated electrons accumulate on Nd-BiO_{2-x}, while holes remain on MIL-101(Fe), consistent with the direct Z-scheme charge transfer pathway driven by the interfacial electric field."

On page 17, the description of DFT has been revised as follows:

"The differential charge density distributions of Nd-BiO_{2-x}/MIL-101(Fe) and B_xM_y/O_2 are shown in **Figure 4g and Figure S30**, highlighting the effect of O_v. The presence of O_v enhances electron transfer from Nd-BiO_{2-x} to MIL-101(Fe), increasing the transferred charge from 2.12 to 2.59 e, which agrees with the work function and KPFM analyses (Figure 2). These calculations also support the O_v-induced formation of Bi–O=C interfacial bonds and the associated electron redistribution driven by the IEF. For Yeager-type O_2 adsorption, the differential charge analysis shows that O_v promote electron transfer from the Bi/Nd sites to adsorbed O_2, with the transferred charge increasing from 0.33 to 0.51 e. To further elucidate the electronic interaction of Nd and

Bi near O_v, the density of states and projected density of states were analyzed **(Figure 4h)**. Unlike transition metals that typically donate *d* electrons to the *π** antibonding orbitals of O_2, Bi possesses filled *d* orbitals, while its 6*s* and 6*p* states lie close to the Fermi level and dominate the valence band together with O 2*p* states. Upon excitation, electrons are promoted to Bi 6*p* and O 2*p* antibonding states, enabling electron transfer to adsorbed O_2. After O_2 adsorption, the PDOS center of the Bi 6*p* orbital shifts from 1.11 to 1.15 eV, and the Nd 4*f*-band center shifts from 0.47 to 1.39 eV **(Figure S31)**, indicating orbital hybridization between Bi/Nd states and O_2 orbitals. The interfacial binding between MIL-101(Fe) clusters and the $(0\bar{1}1)$ surface of Nd-BiO_{2-x} was further examined by DFT-based molecular dynamics **(Figure S32)**."

Supplementary Materials, on page 28, the description of Nd DFT is as follows:
"To further clarify the atomic-level interaction between Nd and adsorbed O_2, the PDOS before and after O_2 adsorption was calculated by DFT. As shown in **Figure S31a**, the electronic states of Nd are mainly dominated by Nd 4*f* orbitals. After O_2 adsorption, the most obvious change occurs in the Nd 4*f* orbitals (**Figure S31c**), where the 4*f*-band center shifts positively from 0.47 to 1.39 eV. This upward shift indicates orbital hybridization between Nd 4*f* and O_2-derived O 2*p* states, reflecting strong electronic interaction between Nd-associated sites and adsorbed O_2. Such electronic modulation supports the chemisorption and activation of O_2 near the Nd–O_v–Bi local structure, thereby facilitating the subsequent generation of reactive species. These results indicate that Nd enhances the catalytic process by regulating the electronic structure of O_v-rich Bi sites rather than acting as a separate nanoparticle active site."

Comment 2-4: Details regarding the assembly of the continuous flow device and the preparation of the catalyst coating in Figure 5 should be added to the Methods description.

Response: We appreciate the reviewer for this suggestion and we accept it. Accordling, we have added the relevant device fabrication details and catalyst loading procedure to the Methods section in the revised manuscript.
Changes: *On page 21, the description of the related characterization parameters and device fabrication procedures is as follows:*
Methods 4.4
In the continuous-flow sunlight setup, the reservoir was made of acrylic, and the angle-adjustable frame was constructed from stainless steel. The top surface was covered with a 3 mm-thick ultra-clear glass with a light transmittance exceeding 90%. 10 g of the catalyst was loaded onto a 1 cm-thick glass fibre mat via the dry impregnation method. The catalyst-loaded mat was then fixed onto the acrylic backplate with silicone sealant.

Comment 2-5: The detection methods for pollutants and the intermediate products should be described in detail with specific experimental procedures.

Response: We sincerely thank the reviewer for this suggestion. We have provided a detailed description of the detection procedures for various pollutants and the gradient elution program for mass spectrometry in the Supplementary Materials.

Changes: *Supplementary Materials, page 7, the description of the detection methods for pollutants and the intermediate products is as follows:*

Supplementary Materials and Methods 1.6

"BPAF was quantified using ultra-high performance liquid chromatography (UHPLC, LC 300, PerkinElmer, Inc. USA) with a C18 column (PE, Quasar C18 100×2.1 mm 3μm, UK). A methyl alcohol/water (70: 30, v: v) mixture was used as the mobile phase, and the detection wavelength was 225 nm. The elution process was conducted at a flow rate of 0.1 mL min^{-1}.

The concentration of Rhodamine B was determined using a UV-Vis spectrophotometer. The absorbance measurements were recorded at the maximum absorption wavelength of 554 nm using a standard 1 cm quartz cuvette.

Eleven trace emerging contaminants were analyzed using liquid chromatography coupled with triple quadrupole tandem mass spectrometry (LC-MS/MS). The mobile phase consisted of methanol and 0.2% formic acid aqueous solution (90:10, v/v), and the flow rate was 0.2 mL/min. The detailed detection parameters, including fragment ions and m/z ratios, are provided in **Supplementary Table 11**.

The photodegradation intermediates of BPAF were determined by a UHPLC system equipped with a time-of-flight (TOF) mass spectrometer (Agilent 6545). The electrospray interface (ESI) ion source was operated in negative ion mode, and the scan acquisition of MS measurements was m/z 50 − 450. The mobile phase comprised methanol (A) and 0.2% formic acid aqueous solution (B) at a flow rate of 0.2 mL/min. The gradient elution program was set as follows: 0 – 1 min, A/B at 5/95; 1 – 15 min, the A/B ratio transitioned linearly from 5/95 to 95/5 and was maintained at this ratio until 24 min; 24 – 26 min, the A/B ratio was rapidly switched back to 5/95 and held at this proportion until 30 min to equilibrate the column for the next injection."

Comment 2-6: The analysis of the final intermediate products and related toxicological changes is relatively brief in the text. Such analysis should be conducted in greater detail to highlight the practical advantages of the application.

Response: We thank the reviewer for this helpful suggestion. We have expanded the discussion of the BPAF degradation pathways and toxicity changes in the revised

manuscript. The added analysis provides a clearer description of the main reaction steps, including hydroxylation, oxidation, ring cleavage, and partial defluorination, as well as the reduced ecological toxicity of the degradation products.

Changes: *Page 20, the description of the final intermediate products and related toxicological changes is as follows:*

"Degradation intermediates and possible reaction pathways were identified using HPLC–MS combined with DFT calculations **(Figure 5h, Table S10-11, and Figures S38-39)**. Due to their strong oxidizing power, h^+ abstract electrons from the benzene ring via single-electron transfer, leading to hydroxylation reactions at the *ortho*- and *para*-positions. Additionally, $^1O_2$ undergoes addition to the phenol ring to form benzoquinone derivatives, which subsequently undergo ring cleavage. Meanwhile, e^- can contribute to the reductive cleavage of a small fraction of C–F bonds, promoting partial defluorination. Toxicity assessments of BPAF and its intermediates **(Figures S40 and S41)** show that toxicity toward algae, daphnia, and fish decreases as molecular polarity is reduced during degradation."

Comment 2-7: The comparison between simulated light intensity in laboratory conditions and actual solar irradiation should be further clarified to improve the relevance to realistic applications.

Response: We thank the reviewer for this valuable suggestion. We completely agree that clarifying the relationship between our simulated laboratory conditions and actual solar irradiation is crucial for demonstrating the real-world applicability of our work. The light intensity of the xenon lamp in the laboratory is 20×AM 1.5G, whereas the measured solar intensity during the continuous-flow outdoor experiments was approximately 2×AM 1.5G. We have added the corresponding light-intensity information in the Methods 4.4 section and in the captions of Figures 3 and 5. Furthermore, the spectral distribution of our simulated light in the laboratory highly matches the natural solar spectrum, containing comparable proportions of ultraviolet (~5%), visible (~43%), and near-infrared (~52%) photons. To directly address the relevance to realistic applications, our continuous-flow degradation experiments were conducted outdoors driven entirely by actual natural solar irradiation. These experiments were conducted outdoors on sunny days in July.

Comment 2-8: p.14, "the carbonyl and hydroxyl bands of the carboxyl groups in phthalic acid weaken and shift to lower wavenumbers, indicating outward electron transfer to BiO_{2-x} through Bi-O=C interfacial bonds." It should be clearly stated that the electrons of the carboxyl group are being transferred.

Response: We thank the reviewer for proposing this question. We have revised the claim to clarify that the electron transfer originates from the carboxyl groups.

Changes: *On page 16, the description of the in situ FTIR is as follows:*

"The carbonyl and hydroxyl bands of the carboxyl groups in phthalic acid weaken and shift to lower wavenumbers, indicating electron transfer from the carboxyl groups to BiO_{2-x} through Bi–O=C interfacial bonds."

Comment 2-9: Further miscellaneous comments:

* Provide the fitting model and corresponding equations used for the TRPL analysis. This would improve the clarity and reproducibility of the carrier lifetime interpretation.

Response: We thank the reviewer for this helpful suggestion. We have added the fitting model and corresponding equations used for TRPL analysis to the Supplementary Materials to improve the clarity and reproducibility of the carrier lifetime analysis.

Changes: *Supplementary Materials, pages 4 and 5, the following methods of the fitting model and corresponding equations used for the TRPL analysis were added:*

The time-resolved photoluminescence decay curves were fitted using a double-exponential decay model. Components 1 and 2 represent the short-lived and long-lived decay processes, respectively. The equations (S1-2) for the exponential fitting and the calculation of the average lifetime are given below:

$$Y = A + B_1 e^{-\frac{i}{T_1}} + B_2 e^{-\frac{i}{T_2}} \quad (S1)$$

$$T = \frac{B_1 T_1}{B_1 + B_2} + \frac{B_2 T_2}{B_1 + B_2} \quad (S2)$$

where B is the weighting coefficient, T is the lifetime, and Y represents the experimental data.

* In Figure 1k, the FT-EXAFS should indicate the positions of the characteristic peaks of Fe-O and Fe-Fe.

Response: We thank the reviewer for this helpful suggestion. We have revised Figure 1k by labeling the characteristic Fe–O and Fe–Fe coordination peaks in the k^2-weighted FT-EXAFS spectra. In addition, the FTIR spectra in Figure 1d have also been annotated more clearly, with paired dashed lines of the same color indicating the redshift or blueshift of the corresponding peaks.

Changes: *On page 6, the Figures 1d and 1k are as follows:*

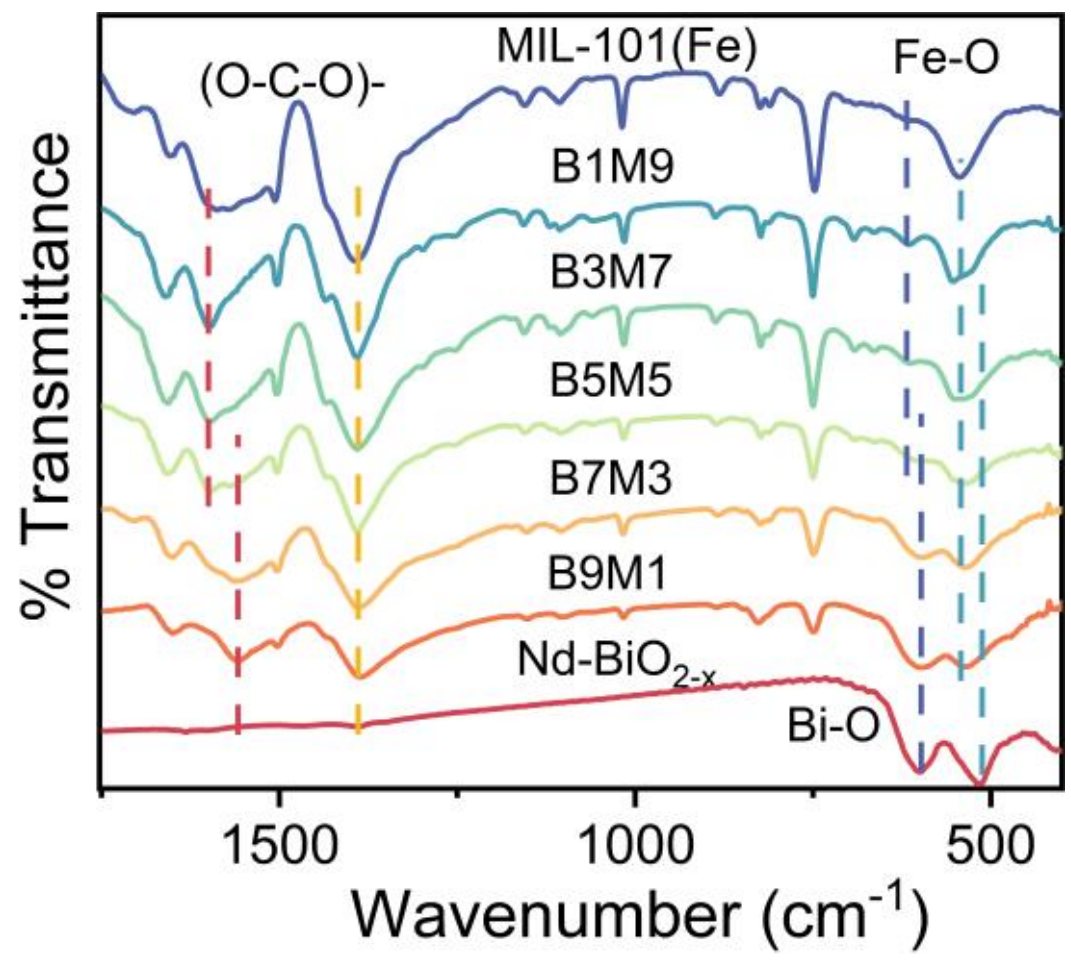

Figure 1 | (d) FTIR spectra of Nd-BiO_{2-x}, MIL-101(Fe), and B_xM_y heterojunctions, where two dashed lines indicate peak shifts.

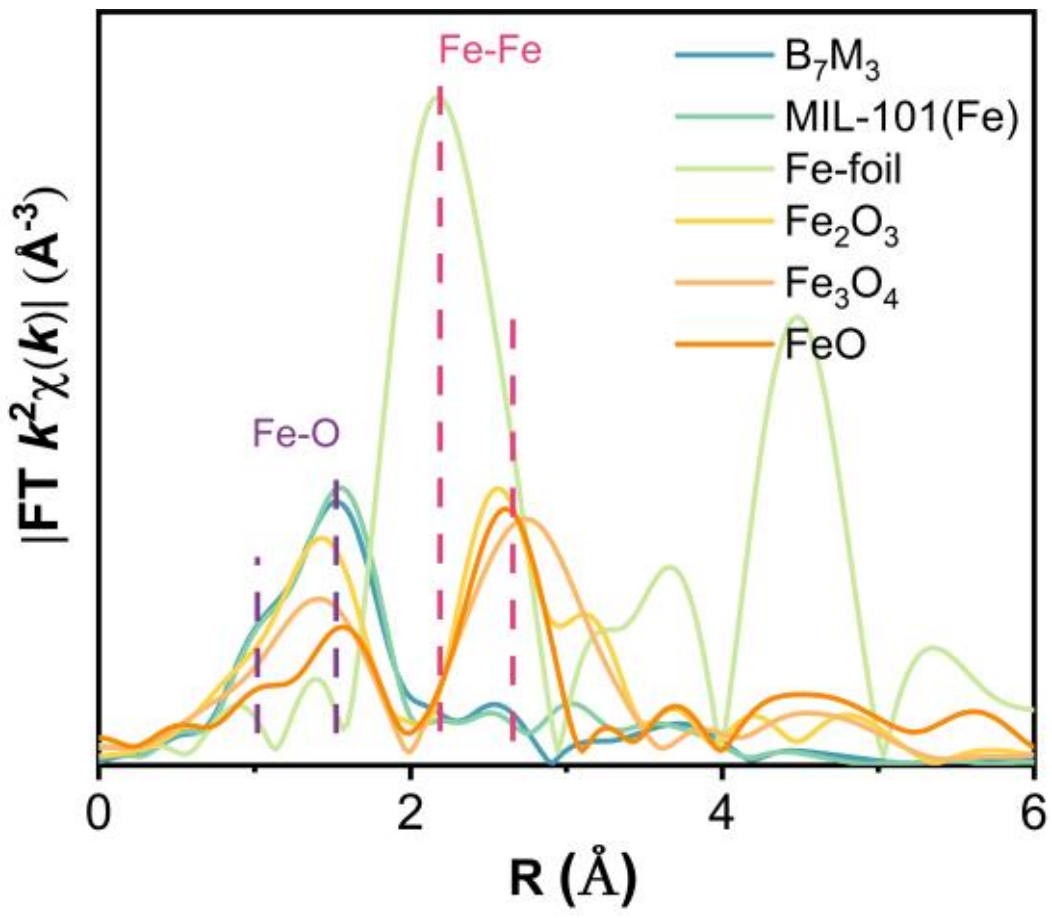

Figure 1 | (k) k^2-weighted Fe FT-EXAFS spectra of the as-prepared samples.

* Some sentences contain grammatical mistakes, such as on page 3, in the INTRODUCTION, "limited" should be "limit". On page 5, section 2.1, "confirm" should be "confirms". On page 15, section 2.5, "strong capability" should be "a strong capability", and "andmixed" should be "and mixed".

Response: We thank the reviewer for carefully checking the manuscript. We have corrected these grammatical errors and revised the corresponding sentences accordingly.

Changes:

"Conventional heterojunctions are mainly held together by weak physical interactions, such as van der Waals forces or electrostatic attraction, which often limit interfacial electron coupling because of lattice mismatch[19,20]."

"Element mapping **(Figure 1c and S3d)** confirms that Nd-BiO_{2-x} is uniformly anchored on the surface of MIL-101(Fe), with homogeneous distributions of Nd, Bi, Fe, and O."

"After BPAF addition, a rapid potential drop is observed, with B_7M_3 showing the largest decrease of 55 mV (from 515 to 460 mV), indicating a strong capability for direct oxidation of organic pollutants in water."

* For the "?" mark in Figure 2, no explanation was provided in the original text. It is suggested to add relevant descriptions to facilitate the subsequent discussion.

Response: We sincerely thank the reviewer for pointing out this issue. Initially, we intended to use a question mark to introduce the subsequent discussion of the hole oxidation mechanism. However, to make the figure clearer and avoid ambiguity, we have revised this part of Figure 2 to directly present the proposed charge transfer and oxidation mechanism.

Changes: *On page 10, the Figure 2 has been revised as follows:*

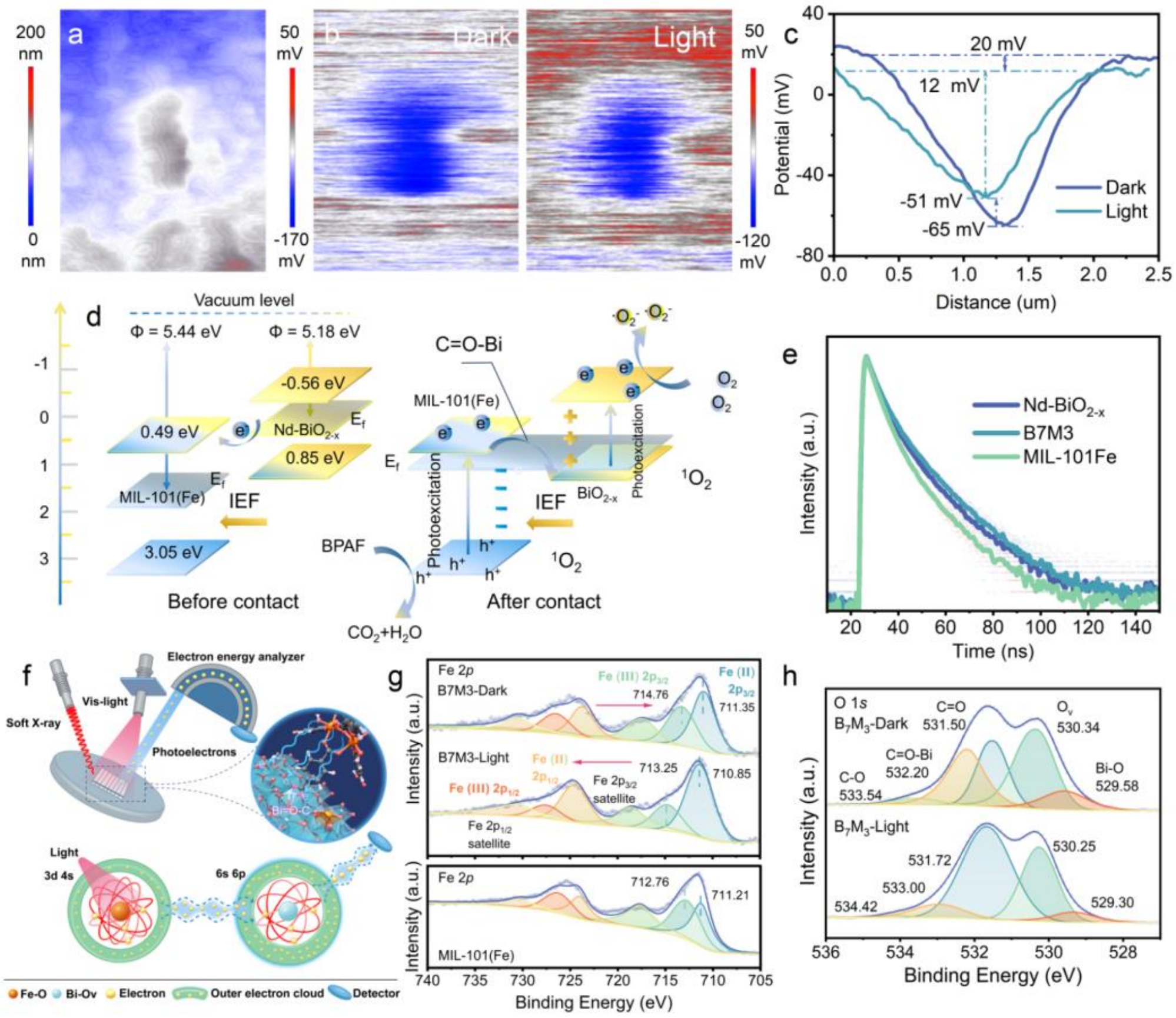

Figure 2 | Electron transfer pathway and photophysical and electrochemical measurements. (a-c) KPFM images and corresponding surface potential distribution of B_xM_y heterojunctions in darkness and under light. **(d)** Schematic illustration of the Nd-BiO_{2-x}/MIL-101(Fe) direct Z-scheme heterojunction with an interfacial electric field. **(e)** Transient photocurrent response densities of as-prepared samples. **(f-h)** Schematic illustration of the in situ XPS setup and high−resolution Fe 2*p* and O 1*s* spectra before and after light.

Response to Reviewer #3

Reviewer's general comments

The author prepared an Nd-BiO_{2-x}/MIL-101(Fe) photocatalyst and proposed an O_v-induced direct Z scheme heterojunction. The heterojunction material exhibited excellent degradation performance for both BPAF and RhB. Combined with DFT calculations and a series of advanced characterizations, the possible reaction mechanism for photocatalytic pollutant degradation was inferred. Furthermore, tests under natural sunlight demonstrated efficient removal of 11 pollutants from reservoir water, highlighting the system's potential.

Response:

We sincerely thank reviewer #3 for the constructive and encouraging assessment, which has been highly valuable and has substantially helped us improve the quality of the manuscript. We especially appreciate the professional comments regarding the electron transfer pathway, which have greatly helped us refine the mechanistic interpretation. All comments have been carefully addressed, and the manuscript has been thoroughly revised to improve its clarity and rigour. In particular, we have added extended performance tests and BET characterization to clarify the specific surface area of the material and the role of heterojunction in pollutant degradation. These revisions strengthen the mechanistic discussion and practical relevance of this work, and we hope they have addressed the reviewer's questions and suggestions

Comment 3-1: In the high-resolution TEM image of Figure 1c, the lattice fringes of MIL-101(Fe) cannot be observed. How can the construction of the heterojunction be proven?

Response: We thank the reviewer for the insightful question. MIL-101(Fe), as an Fe-containing metal-organic framework, is sensitive to high-energy electron beams irradiation and may undergo partial structural damage during high-resolution TEM observation. Therefore, the absence of clear MIL-101(Fe) lattice fringes in the HRTEM image does not exclude the formation of the heterojunction. In the revised manuscript, we have further clarified the construction of the heterojunction using multiple lines of evidence:

First, the HAADF-STEM and EDS mapping images **(Figure 1c)** show highly overlapping and uniform distribution of Nd, Bi, Fe, and O, confirming the close contact between Nd-BiO_{2-x} and MIL-101(Fe) at the nanoscale. SEM and HRTEM images also show that the octahedral boundaries morphology of MIL-101(Fe) is retained after hybridization **(Figure S2 and S3)**. In addition, the selected area electron diffraction pattern shows diffraction spots assignable to the {101} planes of MIL-101(Fe) **(Figure**

1b). The HRTEM and aberration-corrected TEM images also reveal local lattice distortion and close interfacial contact, which may contribute to the formation of defect-rich interfacial regions on the Nd-BiO_{2-x} surface and facilitate interfacial electronic coupling **(Figure 1c)**. As highlighted by the yellow dashed lines, the parallel lattice fringes are disrupted. In these identified regions, the atomic planes are seen to terminate abruptly, twist away from their optimal directions, or shift laterally by half a lattice spacing. These features are characteristic of classical edge dislocations and widespread lattice distortions. Instead of forming a perfect single crystal, the BiO_{2-x} component becomes highly fragmented into smaller, misoriented domains that are separated by these dislocation-rich boundaries.

Second, the high-resolution XPS spectra show a clear binding energy shift of Fe *2p* compared with pristine MIL-101(Fe), indicating strong interfacial electronic interaction and charge redistribution between MIL-101(Fe) and Nd-BiO_{2-x} **(Figure 2g)**. This supports the formation of a chemically coupled heterojunction rather than a simple physical mixture.

FTIR and XAS (**Figure 1d and 1j-k**) analyses reveal changes in the local coordination environment after coupling. In particular, the fitted Bi–O=C coordination in B_7M_3 confirms the formation of interfacial bonding between oxygen-containing groups from MIL-101(Fe) and Bi sites in Nd-BiO_{2-x}. These results indicate that the two components are chemically coupled rather than simply physically mixed.

Changes: *On page 5, the description of the HRTEM and Aberration-Corrected-TEM are as follows:*

"A combined hydrothermal treatment and ball-milling strategy was employed to fabricate a series of vacancy-rich Nd-BiO_{2-x}/MIL-101(Fe) (B_xM_y) **(Figure 1a and Figure S1)**. The sheet material of the BiO_{2-x} attached to the octahedron of the MIL-101(Fe) was observed in the scanning electron microscope (SEM) image **(Figure S2)**. Likewise, a heterostructure composed of MIL-101(Fe) and BiO_{2-x} layer can be observed in the high-angle annular dark-field scanning transmission electron microscopy (HAADF-STEM) and its corresponding electron diffraction image of B_xM_y (**Figure 1b and Figure S3a-c)**. The strained heterojunction shows local lattice distortion and close interfacial contact, which may create defect-rich regions on the Nd-BiO_{2-x} surface and facilitate interfacial electronic coupling. Although clear lattice fringes of MIL-101(Fe) are difficult to resolve in the high-resolution TEM image because MOF frameworks are sensitive to high-energy electron-beam irradiation, the retained octahedral morphology and the close interfacial contact between BiO_{2-x} and MIL-101(Fe) support the successful integration of the two components. In the selected area electron diffraction (SAED) pattern, diffraction spots corresponding to the {101} lattice planes of MIL-101(Fe) are also observed. Aberration-Corrected-TEM images **(Figure 1c)** show real

lattice spacings of 0.317 nm and 0.274 nm, corresponding to the (111) and (200) planes, respectively, indicating that BiO_{2-x} preferentially exposes the $(01\bar{1})$ facet[12]. Element mapping **(Figure 1c and S3d)** confirms that Nd-BiO_{2-x} is uniformly anchored on the surface of MIL-101(Fe), with homogeneous distributions of Nd, Bi, Fe, and O. These morphological, diffraction, and elemental mapping results collectively confirm the nanoscale contact between Nd-BiO_{2-x} and MIL-101(Fe), which provides structural evidence for heterojunction construction."

Page 7:

"With increasing MIL-101(Fe) content, the intensity of the Bi–O vibration on the exposed $(0\bar{1}\bar{1})$ facet decreases markedly, indicating interfacial bonding between C=O or Fe–O groups and Bi–O or Bi–O_v sites on BiO_{2-x}. These peak shifts indicate that the two components are not simply physically mixed, but are chemically coupled through vacancy-induced interfacial bonding."

Pages 7 and 8:

"The resolved O 1*s* peaks corresponding to O_v and chemisorbed O_2 in BiO_{2-x} and B_7M_3 further confirm the presence of O_v. The Bi 4*f* spectra indicate a shift consistent with continuous electron transfer from bismuth species to MIL-101(Fe). Combined with the FTIR results, these XPS changes provide direct chemical evidence for interfacial coupling between Nd-BiO_{2-x} and MIL-101(Fe)."

Comment 3-2: KPFM measurements before and after illumination should be further confirmed. Under dark conditions, the potential in regions associated with MIL-101(Fe) is lower than that of BiO_{2-x}, indicating that MIL-101(Fe) has a higher work function. This suggests that under dark conditions, BiO_{2-x} should serve as the electron donor, rather than MIL-101(Fe).

Response: We sincerely thank the reviewer for this insightful comment. We agree that the KPFM results under dark conditions should be interpreted more clearly. In the dark state, the lower surface potential of MIL-101(Fe) indicates its higher work function compared with Nd-BiO_{2-x}. Therefore, upon contact, electrons transfer from Nd-BiO_{2-x} to MIL-101(Fe) until Fermi-level equilibrium is reached, meaning that Nd-BiO_{2-x} acts as the electron donor and MIL-101(Fe) acts as the electron acceptor under dark conditions. This interfacial charge redistribution establishes an intrinsic electric field from Nd-BiO_{2-x} to MIL-101(Fe). Under illumination, the charge distribution changes because of photoexcitation. The KPFM results show an increase in the central potential and a decrease in the edge potential, indicating photoinduced charge redistribution across the heterojunction. Together with the in situ XPS results, these observations support the direct Z-scheme pathway, in which photogenerated electrons accumulate

on BiO_{2-x}, while holes remain on MIL-101(Fe). We have revised the related discussion to clearly distinguish the dark-state interfacial equilibration from the light-induced charge transfer process.

Changes: *On pages 7 and 8, the description of the XPS and KPFM are as follows:*

"B_7M_3 exhibits two additional O 1s components with binding energies located between those of C–O (533.71 eV) and C=O (531.80 eV), which is attributed to the formation of Bi–O=C bonds between Bi–O_v sites and the carboxyl groups of MIL-101(Fe)[10]. The resolved O 1*s* peaks corresponding to O_v and chemisorbed O_2 in BiO_{2-x} and B_7M_3 further confirm the presence of O_v. The Bi 4*f* spectra show clear binding energy shifts after heterojunction formation, indicating interfacial electron redistribution between Nd-BiO_{2-x} and MIL-101(Fe). Combined with the KPFM results, this charge redistribution is attributed to electron transfer from Nd-BiO_{2-x} to MIL-101(Fe) under dark equilibrium because MIL-101(Fe) has a higher work function. As a result, upon contact, Nd-BiO_{2-x} becomes relatively electron-deficient, while MIL-101(Fe) becomes electron-rich, establishing an intrinsic electric field directed from Nd-BiO_{2-x} to MIL-101(Fe). Under illumination, this built-in electric field facilitates directional charge migration and spatial separation of photogenerated carriers[30]."

On page 9:

"Therefore, the lower surface potential of MIL-101(Fe) indicates its higher work function compared with Nd-BiO_{2-x}. After contact, electrons transfer from Nd-BiO_{2-x} to MIL-101(Fe) until Fermi-level equilibrium is reached, with Nd-BiO_{2-x} serving as the electron donor and MIL-101(Fe) as the electron acceptor in the dark state[36]. Under irradiation, photoinduced charge redistribution occurs across the heterojunction, leading to an increase in the central potential from −65 to −51 mV and a decrease at the edges from 20 to 12 mV. These changes indicate that the light-induced charge transfer process differs from the initial dark-state equilibration. Photogenerated electrons preferentially accumulate on Nd-BiO_{2-x}, while holes remain on MIL-101(Fe), consistent with the direct Z-scheme charge transfer pathway[37].

In n-type semiconductor photocatalysts, electrons are the dominant charge carriers and play a central role in catalytic reactions. The energy level alignment between Nd-BiO_{2-x} and MIL-101(Fe) is schematically shown in **Figure 2d**. Density functional theory calculations with dipole corrections were used to determine the work functions of the two components **(Figure S18)**."

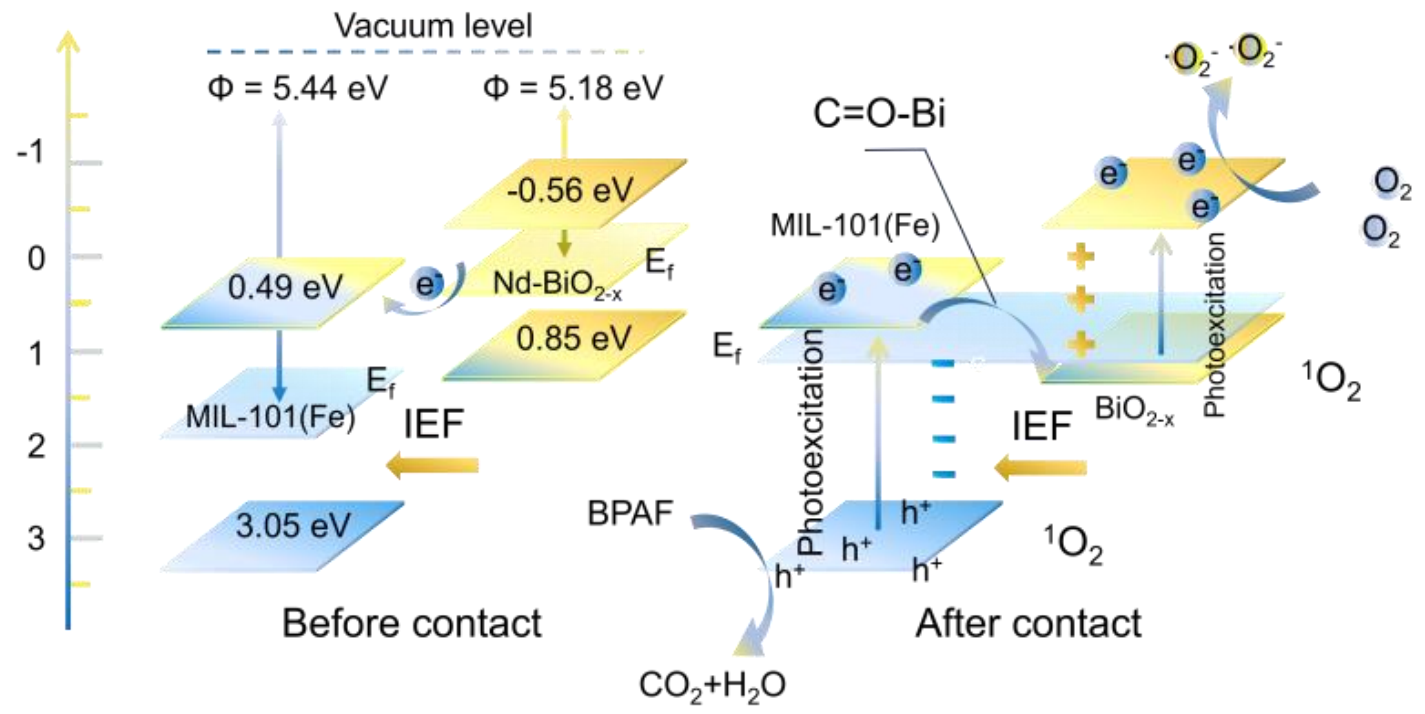

Figure 2 | (d) Schematic illustration of the Nd-BiO$_{2-x}$/MIL-101(Fe) direct Z-scheme heterojunction with an interfacial electric field.

Comment 3-3: In the charge transfer diagram of Figure 2d, the work function of MIL-101(Fe) should be checked and corrected.

Response: We sincerely thank the reviewer for this valuable suggestion. We have carefully checked and corrected the work function of MIL-101(Fe) in the revised Figure 2d. We have also revised the charge transfer diagram by clearly labeling the corresponding catalysts and distinguishing the interfacial charge redistribution under dark conditions from the photogenerated charge transfer process under illumination. In response to this comment and **Comment 3-20** later, we have recalculated the electrostatic potentials and work functions by including dipole corrections. The corrected results show that MIL-101(Fe) has a higher work function than Nd-BiO$_{2-x}$. Therefore, under dark conditions, electrons transfer from Nd-BiO$_{2-x}$ to MIL-101(Fe) until Fermi-level equilibrium is reached, establishing an interfacial electric field from Nd-BiO$_{2-x}$ to MIL-101(Fe). Under illumination, the built-in electric field promotes the direct Z-scheme charge transfer pathway, in which photogenerated electrons accumulate on Nd-BiO$_{2-x}$ and holes remain on MIL-101(Fe). These corrections have been included in the revised text and Figure 2d.

Changes: *Supplementary Materials, page 20, electrostatic potentials of different photocatalysis were added:*

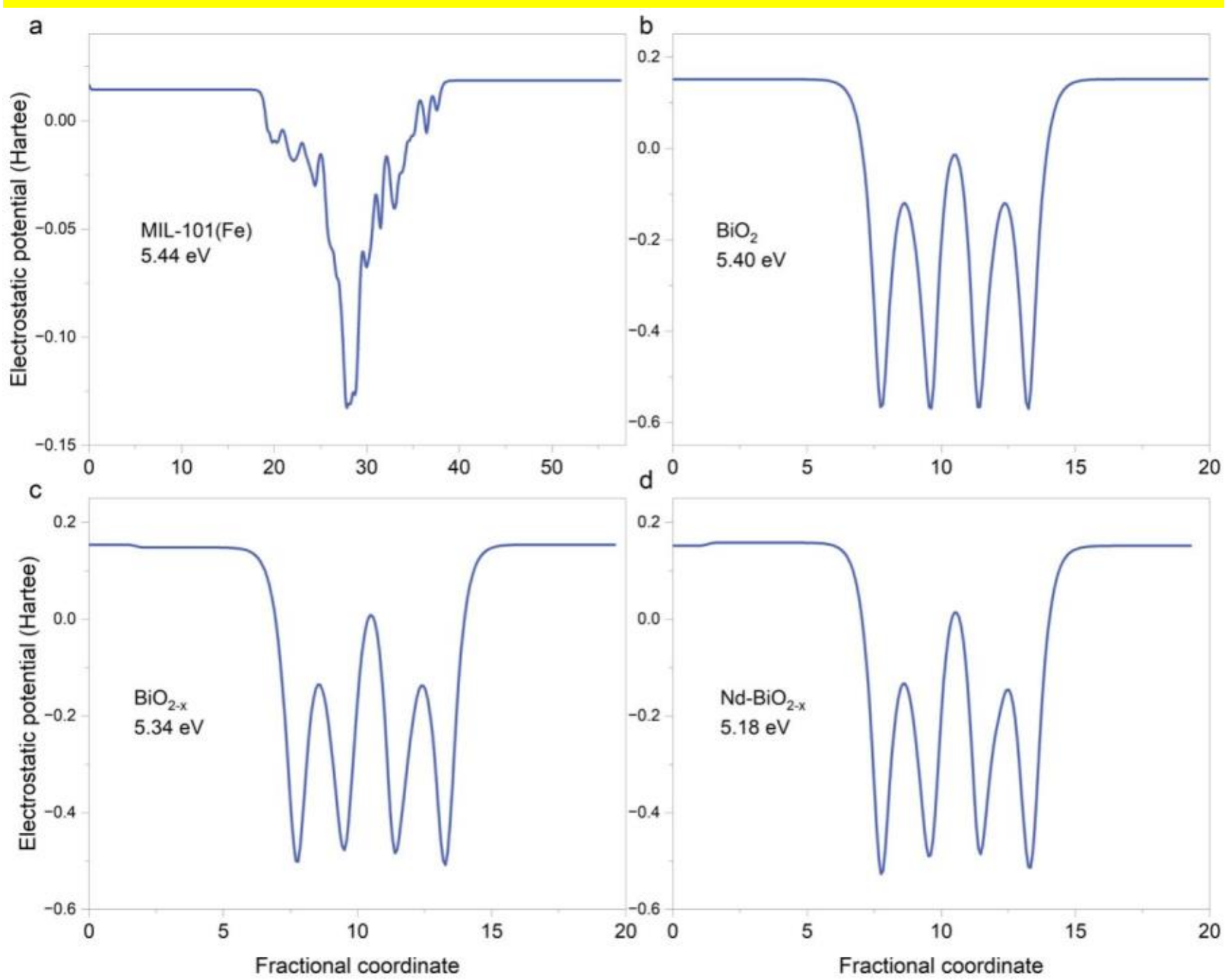

Supplementary Fig.18 | Electrostatic potentials of BiO_2, BiO_{2-x}, Nd-BiO_{2-x}, and MIL-101(Fe).

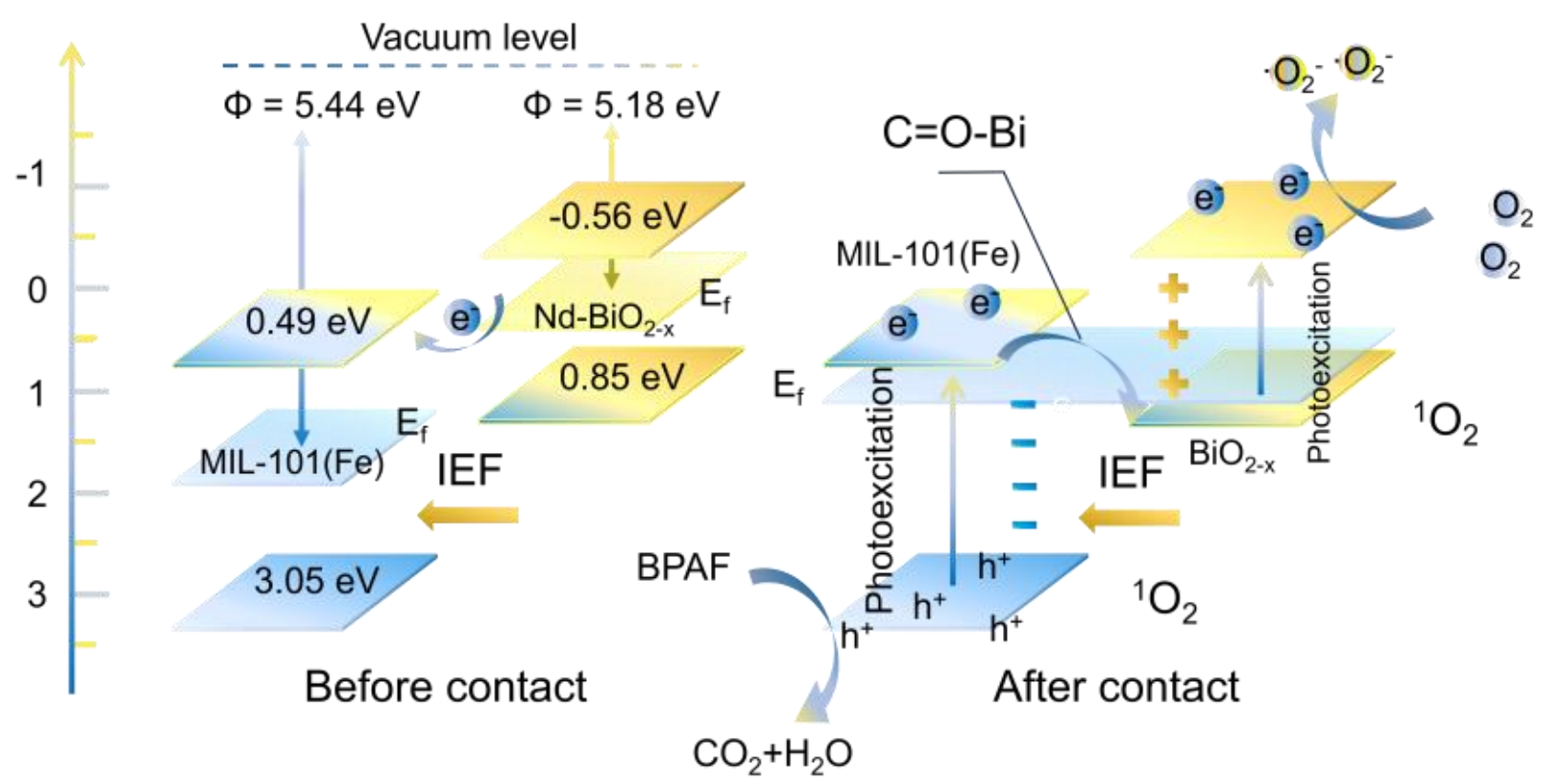

Figure 2 | (d) Schematic illustration of the Nd-BiO_{2-x}/MIL-101(Fe) direct Z-scheme heterojunction with an interfacial electric field.

Comment 3-4: The authors used XPS spectra of Bi 4f of the as-prepared samples to show an increase in the binding energy of Bi, inferring that BiO_{2-x} can provide electrons to MIL-101(Fe). It is recommended to supplement the XPS data of Fe for MIL-101(Fe) and the heterojunction material to further directly demonstrate the charge transfer direction.

Response: We deeply appreciate your valuable comments. In the revised manuscript, we have added Fe 2p XPS data for MIL-101(Fe) and the B_7M_3 heterojunction, together

with in situ XPS measurements under dark and illuminated conditions. These results allow the electronic changes of both Bi and Fe sites to be compared directly.

Changes: *On pages 10 and 11, the Figure 2 and the description have been revised as follows:*

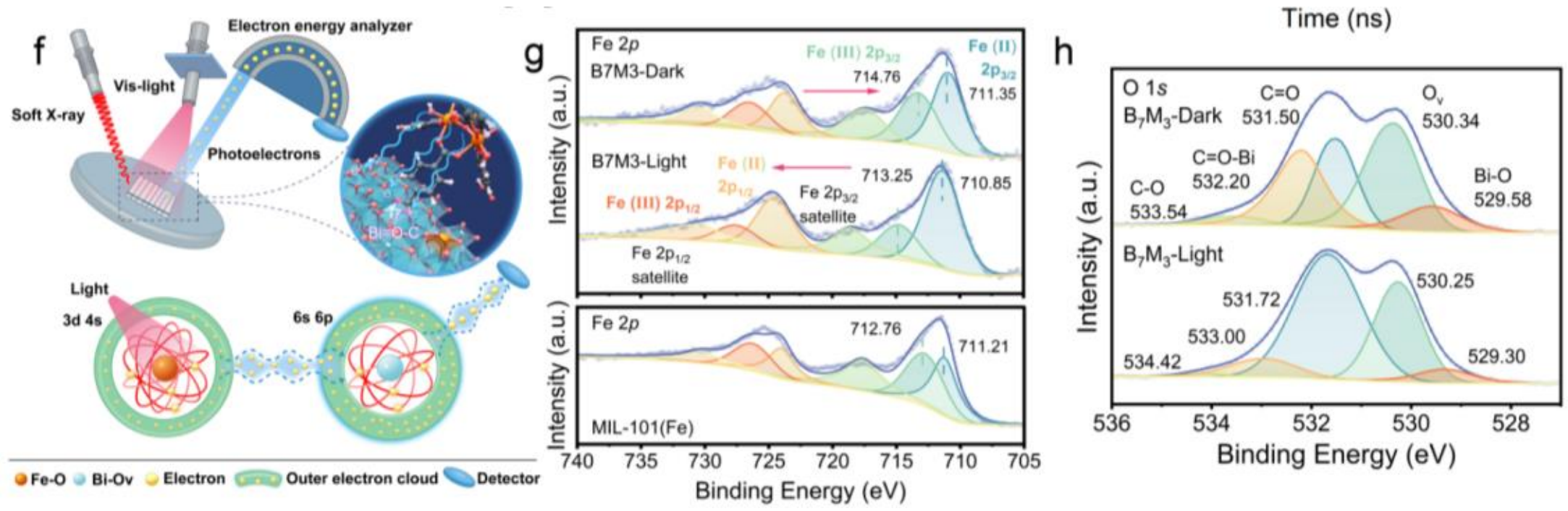

Figure 2 | Electron transfer pathway and photophysical and electrochemical measurements. **(f-h)** Schematic illustration of the in situ XPS setup and high−resolution Fe 2*p* and O 1*s* spectra before and after light.

"To gain deeper insights into the interfacial charge transfer mechanism within the B_xM_y heterojunction, comparative XPS analyses were conducted under both dark and illuminated conditions **(Figure 2f-h)**. The Fe 2*p* spectra show clear electronic changes around Fe sites after coupling with Nd-BiO_{2-x}, supporting interfacial electron redistribution between the two components. Under light irradiation, the binding energies of the Fe 2*p* peaks and the O 1*s* peaks assigned to C=O and C–O bonds shift positively, whereas the Bi 4*f* peaks and the O 1*s* peak assigned to Bi–O shift negatively compared with those in the dark state. These opposite binding energy shifts reveal photoinduced charge redistribution across the heterojunction. Specifically, photogenerated electrons accumulate on Nd-BiO_{2-x}, while holes remain on MIL-101(Fe), consistent with the direct Z-scheme charge transfer pathway driven by the interfacial electric field."

Comment 3-5: How can the statement "MIL-101(Fe) is highly porous with a large void structure" be justified? It is recommended to supplement the pore size distribution of MIL-101(Fe) to illustrate its microporous and mesoporous distribution.

Response: We sincerely thank the reviewer for this helpful suggestion. In the revised Supplementary Materials, we have added N_2 adsorption–desorption isotherms, pore-size distribution analysis, and the corresponding BET parameters of MIL-101(Fe) and the related samples. The BET results show that MIL-101(Fe) has a high specific surface area of 511 m^2 g^{-1}, a total pore volume of 0.44 cm^3 g^{-1}, and an average pore size of 3.46 nm. These results confirm the highly porous nature of MIL-101(Fe) and indicate the

presence of accessible mesoporous structures. We have revised the related statement to avoid overgeneralization and to better reflect the BET and pore-size distribution results.

Changes: In the Supplementary Materials, Supplementary Fig. 12 and Supplementary Table 2 have been added to show the N_2 adsorption–desorption isotherms and textural properties of the as-prepared samples.

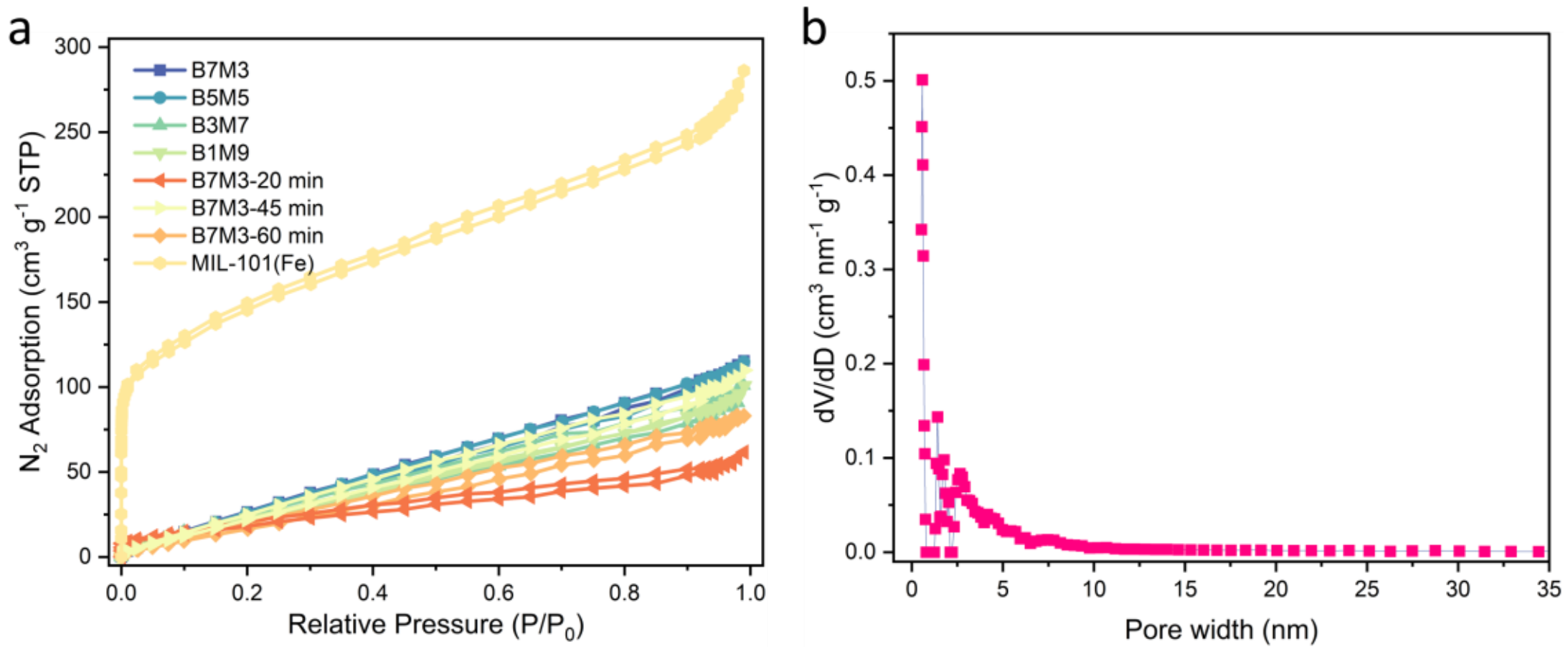

Supplementary Fig.12 | (a) N_2 adsorption-desorption isotherms of Nd-BiO$_{2-x}$, MIL-101(Fe), and BxMy heterojunctions and (b) the pore-size distribution of MIL-101(Fe) using a density functional theory-based pore mode.

Supplementary Table 2 | BET surface areas and pore parameters of the as-prepared samples.

Sample	BET surface area (m^2 g^{-1})	Total pore volume (mL g^{-1})	Average pore size (nm)
Nd-BiO$_{2-x}$	150	0.23	6.12
MIL-101(Fe)	511	0.44	3.46
B_1M_9	122	0.16	5.13
B_3M_7	118	0.16	5.28
B_5M_5	130	0.18	5.46
B_7M_3-20	76	0.10	5.00
B_7M_3-30	137	0.18	5.23
B_7M_3-45	136	0.17	5.01
B_7M_3-60	101	0.13	5.07

Comment 3-6: In the BET test (Figure S11 and Table S2), it is recommended to supplement the data for MIL-101(Fe).

Response: We thank the reviewer for this valuable suggestion. We have added the N_2 adsorption–desorption data and corresponding BET parameters of MIL-101(Fe) to Supplementary Fig. 12 and Supplementary Table 2

Changes: *In Supplementary Materials, on pages 16 and 34, the N_2 adsorption-desorption isotherms and the BET parameters of MIL-101(Fe) have been added as follows:*

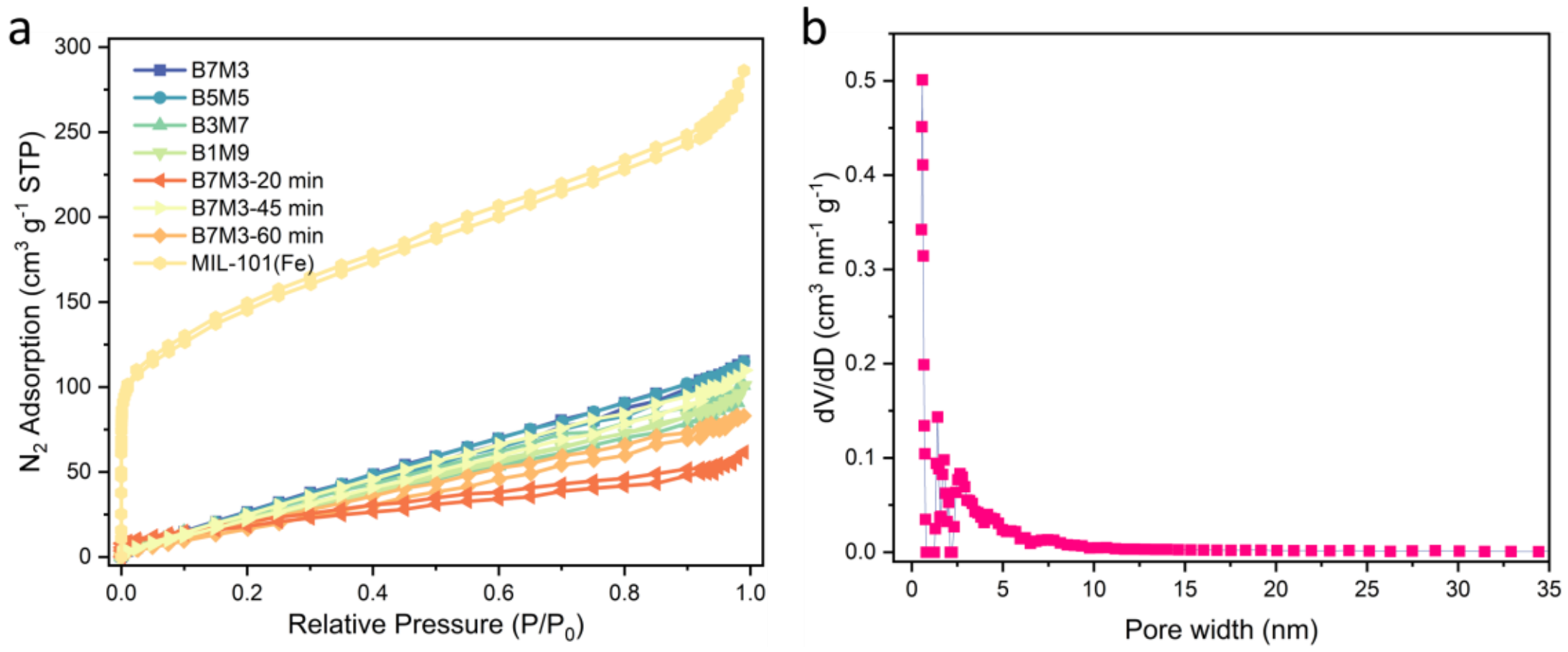

Supplementary Fig.12 | (a) N_2 adsorption-desorption isotherms of Nd-BiO_{2-x}, MIL-101(Fe), and BxMy heterojunctions and (b) the pore-size distribution of MIL-101(Fe) using a density functional theory-based pore mode.

Supplementary Table 2 | BET surface areas and pore parameters of the as-prepared samples.

Sample	BET surface area (m^2 g^{-1})	Total pore volume (mL g^{-1})	Average pore size (nm)
Nd-BiO_{2-x}	150	0.23	6.12
MIL-101(Fe)	511	0.44	3.46
B_1M_9	122	0.16	5.13
B_3M_7	118	0.16	5.28
B_5M_5	130	0.18	5.46
B_7M_3-20	76	0.10	5.00
B_7M_3-30	137	0.18	5.23
B_7M_3-45	136	0.17	5.01
B_7M_3-60	101	0.13	5.07

Comment 3-7: The adsorption and photocatalytic effects of MIL-101(Fe) on BPAF and RhB should be supplemented in the activity tests.

Response: We agree with the reviewer that this issue requires further clarification. We have added the adsorption and photocatalytic control experiments of MIL-101(Fe) toward BPAF and RhB in the revised manuscript and Supplementary Information. The added results show that MIL-101(Fe) exhibits clear adsorption capacity toward BPAF and RhB, but shows negligible photocatalytic activity after adsorption equilibrium under light irradiation.

Changes: *On pages 11 and 12, the description of the photocatalytic and adsorption performance is as follows:*

"MIL-101(Fe) contains abundant surface carboxyl groups and an accessible porous structure, which favors micropollutant adsorption. BPAF and RhB were selected as representative target pollutants to evaluate the adsorption and photocatalytic performance, with BPAF serving as a toxic bisphenol substitute and RhB as a common photocatalytic model compound. As shown in **Figure 3a**, BPAF adsorption increases with MIL-101(Fe) content, reaching 46% for MIL-101(Fe), while RhB shows a similar adsorption removal of 51%. However, after adsorption equilibrium, MIL-101(Fe) shows negligible additional removal under light irradiation, indicating its role mainly as an adsorbent rather than an active photocatalyst. Nd-BiO_{2-x} and B_9M_1 show limited adsorption, whereas B_1M_9 exhibits strong adsorption but poor photocatalytic activity. Under visible light, B_7M_3 achieves the highest BPAF removal of 64% after 90 min through coupled adsorption and photocatalysis, while B_5M_5 shows lower efficiency, likely due to partial coverage of exposed Bi–O and Bi–O_v sites on the $(0\bar{1}\bar{1})$ surface of Nd-BiO_{2-x} by excess MIL-101(Fe). Under NIR irradiation, Nd-BiO_{2-x} shows limited activity, whereas B_7M_3 reaches 51% BPAF removal, confirming its NIR-driven catalytic performance **(Figure S20)**. The RhB removal results show a similar trend under visible and NIR irradiation **(Figure S21)**, further supporting the adsorption–photocatalysis synergy of the Nd-BiO_{2-x}/MIL-101(Fe) heterojunction. Because RhB has better light absorption, its dye polarization effect leads to a faster degradation rate **(Figure S22)**."

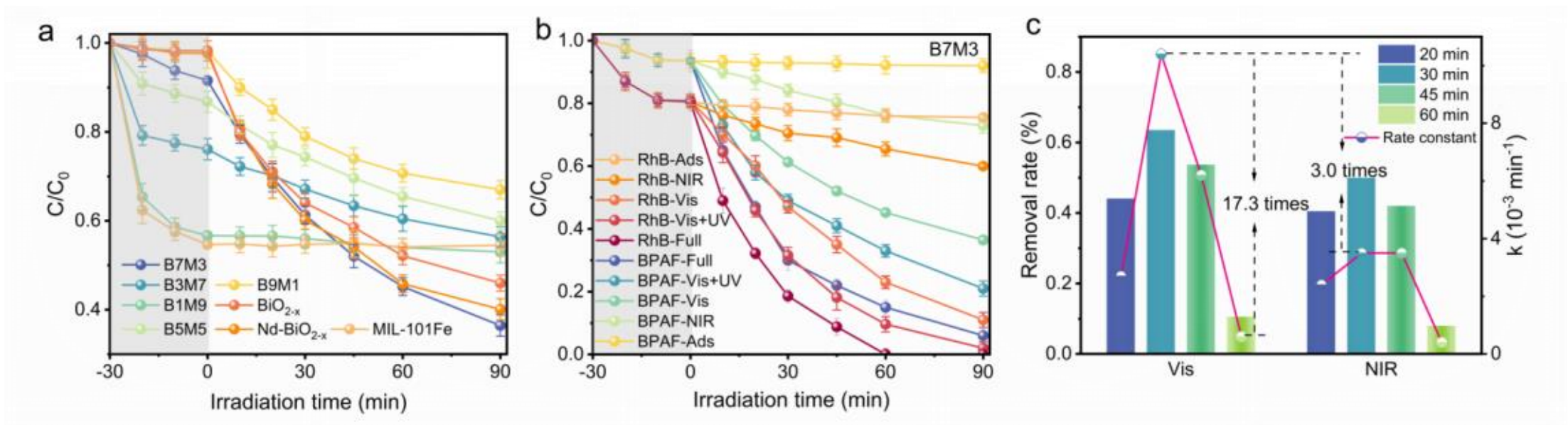

Figure 3 | Photocatalytic performance of B_xM_y. (a) Degradation curves of BPAF with a series B_xM_y under Vis light. **(b)** Photocatalytic efficiency of BPAF and RhB with B7M3 under different light conditions. The error bars represent the standard deviations, obtained by repeating the experiment twice. **(c)** Removal rate and apparent reaction rate constant of B7M3 with various ball-milling times. Reaction conditions: [BPAF] = [RhB] = 0.06 mM, [Cat.] = 0.2 g L^{-1}, $[TEMP]_0$ = 100 mM, $[TEMPO]_0$ = 100 mM, 20×AM 1.5G illumination, [Scavenger] = 10 mM, $[SOD]_0$ = 100 mg L^{-1}, pH_0 = 7.8, and T = 25 ± 3 ℃.

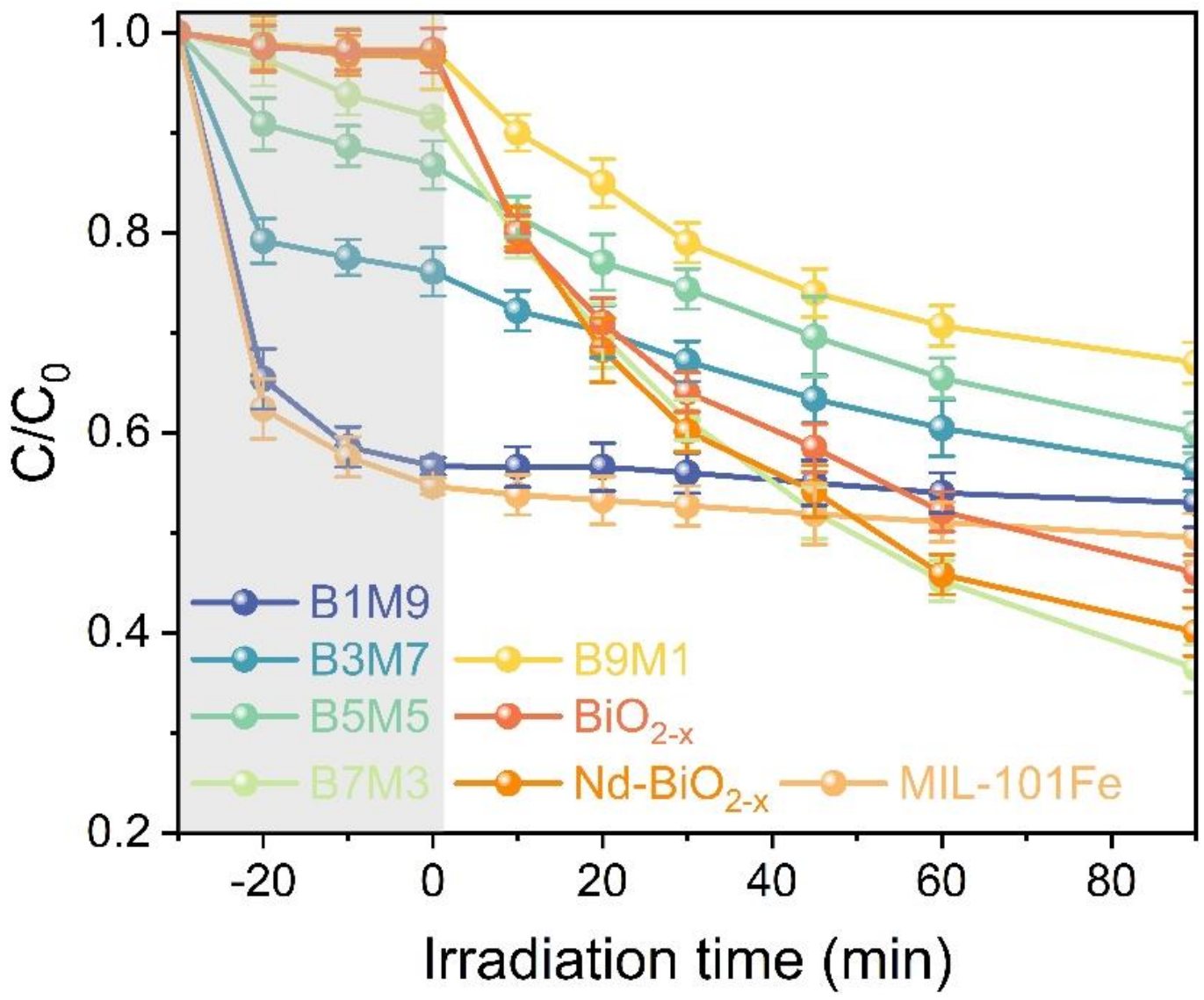

Supplementary Fig.20 | Degradation curves of BPAF with a series B_xM_y under NIR light. Reaction conditions: [BPAF] = 0.06 mM, [Cat.] = 0.2 g L^{-1}, pH_0 = 7.8, and T = 25 ± 3 °C.

Comment 3-8: The photocatalytic performance of the B_xM_y series materials on RhB under visible light and near infrared light should be supplemented.

Response: We are grateful for the reviewer's insightful comment. We have added the RhB removal results over the B_xM_y series under visible and near-infrared irradiation to the revised Supplementary Information (**Figure 3a, 3b and S21**).

Changes: *Supplementary Materials, page 23, the following degradation curves of RhB with a series BxMy under NIR light were added:*

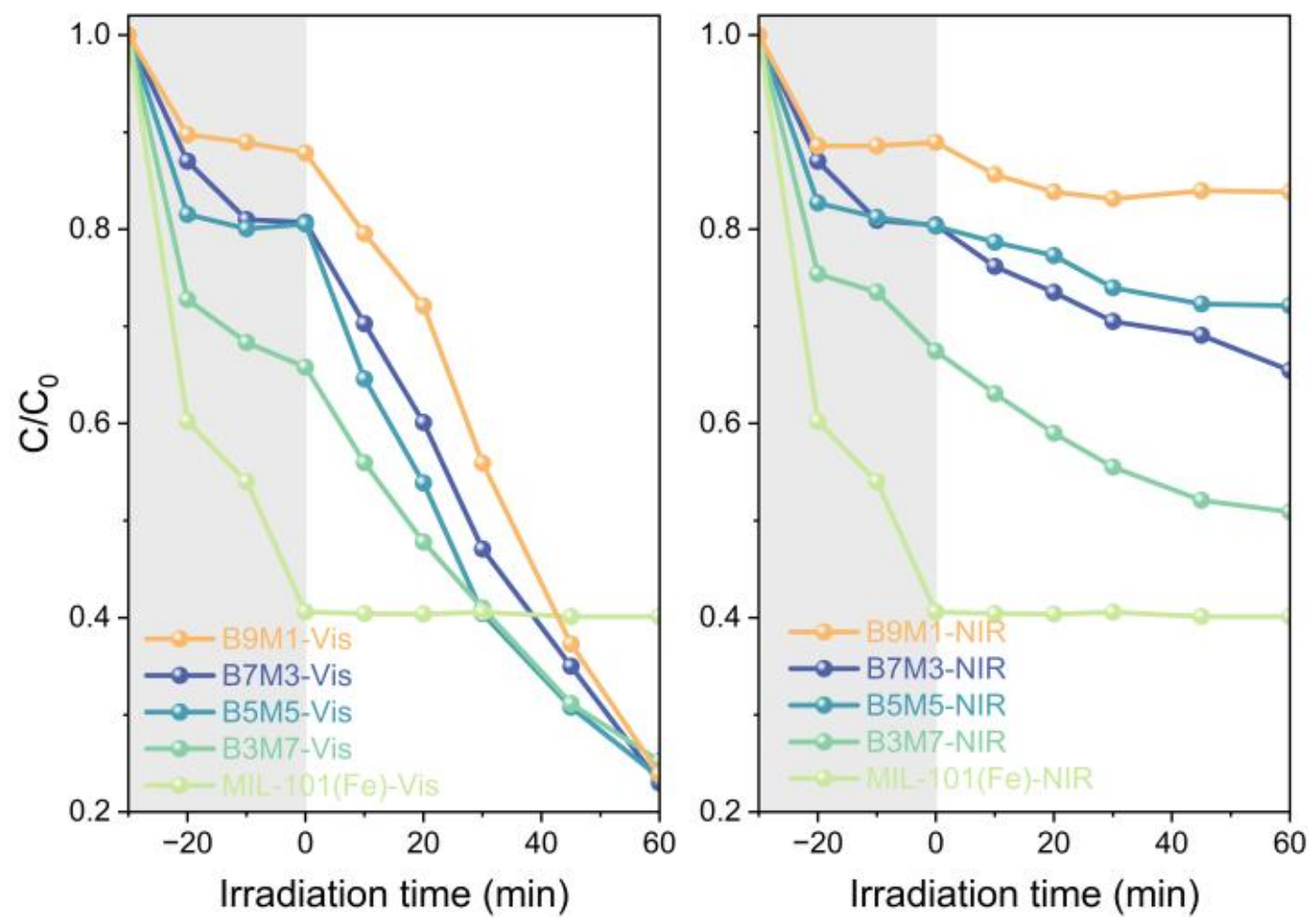

Supplementary Fig.21 | Degradation curves of RhB with a series B_xM_y under Vis and NIR light. Reaction conditions: [RhB] = 0.06 mM, [Cat.] = 0.2 g L^{-1}, pH_0 = 7.8, and T = 25 ± 3 °C.

On page 11:

"As shown in **Figure 3a**, BPAF adsorption increases with MIL-101(Fe) content, reaching 46% for MIL-101(Fe), while RhB shows a similar adsorption removal of 51%. However, after adsorption equilibrium, MIL-101(Fe) shows negligible additional removal under light irradiation, indicating its role mainly as an adsorbent rather than an active photocatalyst."

"The RhB removal results show a similar trend under visible and NIR irradiation **(Figure S21)**, further supporting the adsorption–photocatalysis synergy of the Nd-BiO_{2-x}/MIL-101(Fe) heterojunction. Because RhB has better light absorption, its dye polarization effect leads to a faster degradation rate **(Figure S22)**. Simulated full-spectrum irradiation **(Figure 3b)** further enhances the performance of B_7M_3, with added UV light (<420 nm) markedly increasing degradation efficiency. After 90 min under UV–Vis irradiation, BPAF and RhB removal reach 80% and 99.9%, respectively."

Comment 3-9: What are the cycling stability and reusability of the heterojunction material for the removal of BPAF and RhB?

Response: We deeply appreciate your helpful comments. To evaluate the cycling stability and reusability of the B_7M_3 heterojunction, we have added five consecutive cycling experiments for BPAF and RhB removal under identical experimental conditions. In addition, SEM images and XRD patterns of the catalyst before and after cycling were added to assess its structural stability.

Changes: *On page 13, the description of the stability of the catalyst is as follows:*

"The reusability and structural stability of the photocatalyst are important for its potential application in environmental remediation. To evaluate the cycling stability of B_7M_3, five consecutive degradation cycles were conducted for BPAF and RhB under identical experimental conditions. As shown in **Figure S24**, B_7M_3 maintains good catalytic durability during repeated use. In the first cycle, the removal efficiencies of BPAF and RhB reach 93.2% and 98.5%, respectively. After five successive cycles, the removal efficiencies remain at 78.4% for BPAF and 85.1% for RhB. The slight decrease in performance is mainly attributed to minor catalyst loss during recovery and partial occupation of surface active sites by adsorbed degradation intermediates. To further examine the structural stability of B_7M_3, XRD and SEM characterizations were performed before and after the five-cycle tests. As shown in **Figure S25**, the spent catalyst largely retains its original morphology after cycling, and its XRD pattern remains consistent with that of the pristine catalyst, confirming the good structural stability of B_7M_3."

Supplementary Materials, pages 24 and 25, five consecutive cycling experiments, along with SEM images and XRD patterns were added:

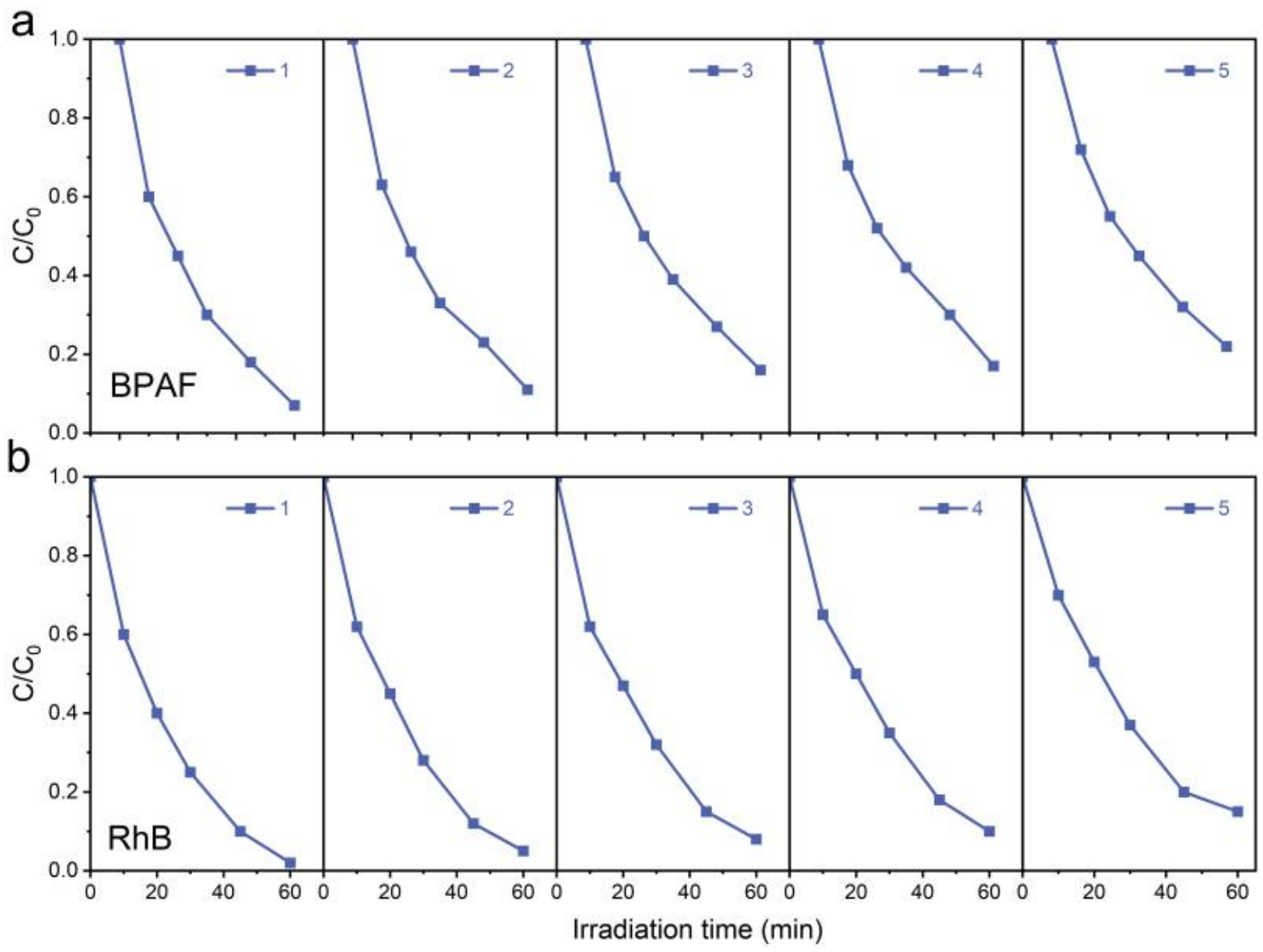

Supplementary Fig.24 | Five consecutive cycling experiments for the degradation of (a) BPAF and (b) RhB under identical experimental conditions. Xenon lamp, [BPAF] = [RhB] = 0.06 mM, [Cat.] = 0.2 g L^{-1}, pH_0 = 7.8, and T = 25 ± 3 °C.

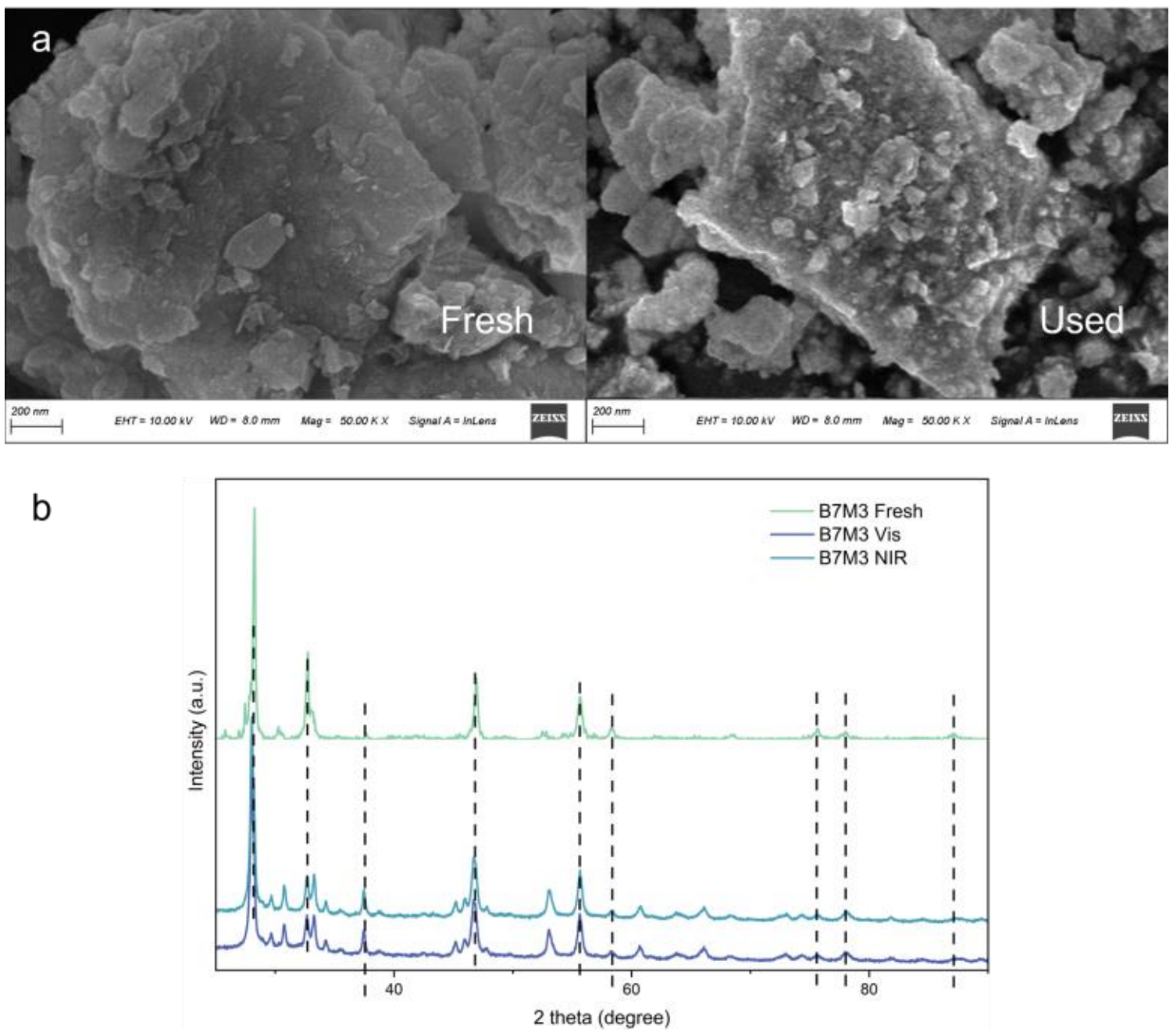

Supplementary Fig.25 | (a) SEM images and (b) XRD spectra of B7M3 before and after five consecutive degradation cycles.

Comment 3-10: In the activity tests, it was found that changing the ball milling time during the preparation of B_7M_3 affects its photocatalytic activity. The authors stated

that this "highlights the necessity of controlling the milling time to protect the pore structure". It is recommended to supplement the specific surface area and pore structure test data of B_7M_3 under different milling times (20-60 min).

Response: We thank the reviewer for raising this important point. To clarify the relationship between ball-milling time, pore structure, and photocatalytic performance, we have added the N_2 adsorption–desorption isotherms and BET parameters of B_7M_3 prepared with different ball-milling times in the revised Supplementary Materials.

Changes: *Supplementary Materials, pages 17, 35, and 41, BET of B7M3 under different milling times were added:*

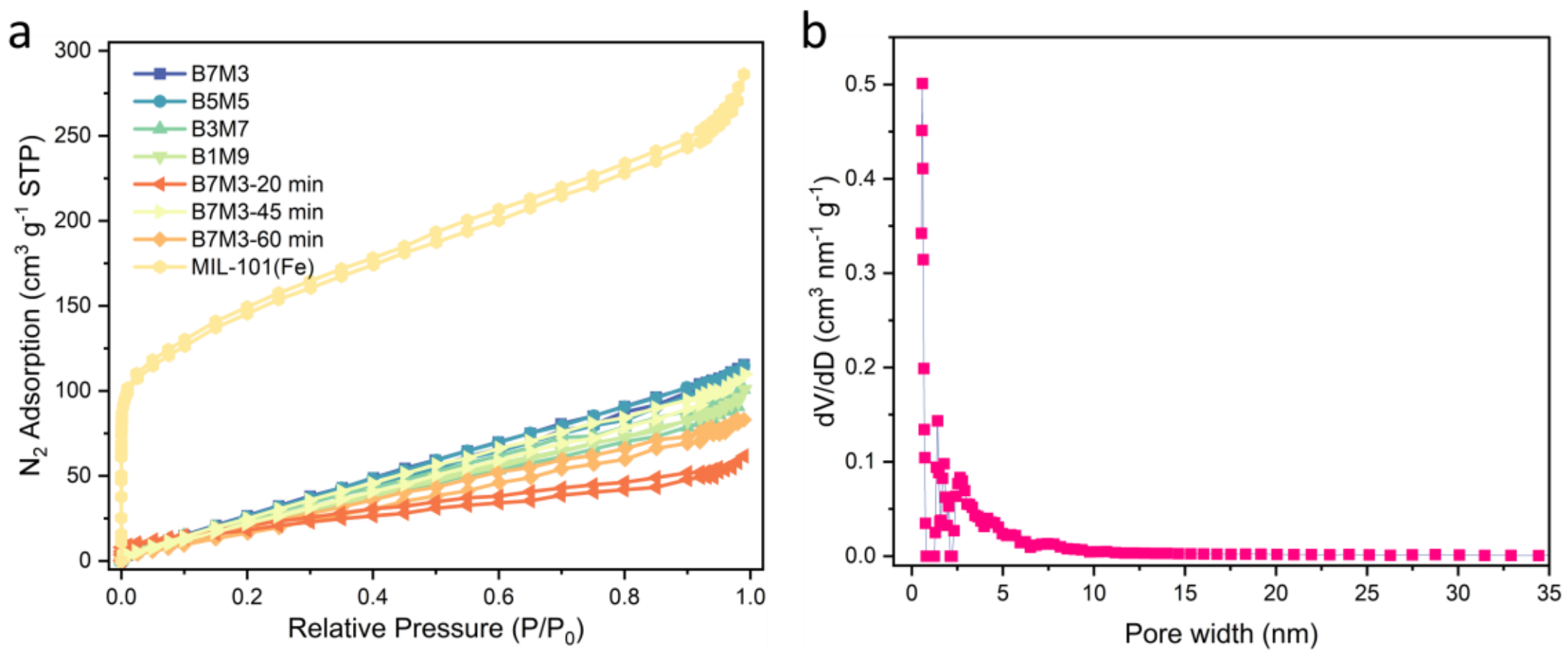

Supplementary Fig.12 | (a) N_2 adsorption-desorption isotherms of Nd-BiO_{2-x}, MIL-101(Fe), and BxMy heterojunctions and (b) the pore-size distribution of MIL-101(Fe) using a density functional theory-based pore mode.

Supplementary Note 1 | Identification of the Ov and the formation of the Bi−O=C bond.

The pore characteristics and specific surface area of the samples are shown in **Supplementary Fig. 12** and **Supplementary Table 2**. As the BiO_{2-x} content increases in the B_xM_y composites, the BET surface area increases from 122 to 137 m^2 g^{-1}, and the total pore volume increases from 0.16 to 0.18 cm^3 g^{-1}. Compared with MIL-101(Fe), the composites show lower surface areas and pore volumes, which can be attributed to interfacial bonding and partial coverage of the MIL-101(Fe) surface by sheet-like BiO_{2-x}.[5] The specific surface area is 76 m^2 g^{-1} with 20 minutes of ball milling, increases to 137 m^2 g^{-1} after 30 minutes. For B_7M_3 prepared with different ball-milling times, prolonging the milling time from 30 to 60 min decreases the BET surface area from 137 to 101 m^2 g^{-1} and the total pore volume from 0.18 to 0.13 cm^3 g^{-1}. This decrease indicates that excessive ball milling can damage the accessible pore structure and reduce the exposure of active sites, explaining the lower photocatalytic activity of

B_7M_3-60 min. Therefore, controlling the ball-milling time is necessary to balance interfacial contact, pore accessibility, and catalytic performance.

Supplementary Table 2 | BET surface areas and pore parameters of the as-prepared samples.

Sample	BET surface area ($m^2\ g^{-1}$)	Total pore volume ($mL\ g^{-1}$)	Average pore size (nm)
Nd-BiO_{2-x}	150	0.23	6.12
MIL-101(Fe)	511	0.44	3.46
B_1M_9	122	0.16	5.13
B_3M_7	118	0.16	5.28
B_5M_5	130	0.18	5.46
B_7M_3-20	76	0.10	5.00
B_7M_3-30	137	0.18	5.23
B_7M_3-45	136	0.17	5.01
B_7M_3-60	101	0.13	5.07

Comment 3-11: It is recommended to supplement the effects of quenching agents on the removal of RhB by the photocatalyst under visible and near infrared light.

Response: We sincerely thank the reviewer for this valuable suggestion. We have added quenching experiments for RhB removal under visible and NIR irradiation in the revised Supplementary Information. The added results show that quenching •OH, $•O_2^-$, and H_2O_2 only slightly affects RhB removal, whereas the scavenging of $^1O_2$ and h^+ leads to a clear decrease in degradation efficiency. These results indicate that $^1O_2$ and photogenerated h^+ are the dominant reactive species for RhB removal, consistent with the BPAF degradation mechanism.

Changes: *Supplementary Materials, page 26, effects of quenching agents on RhB removal were added:*

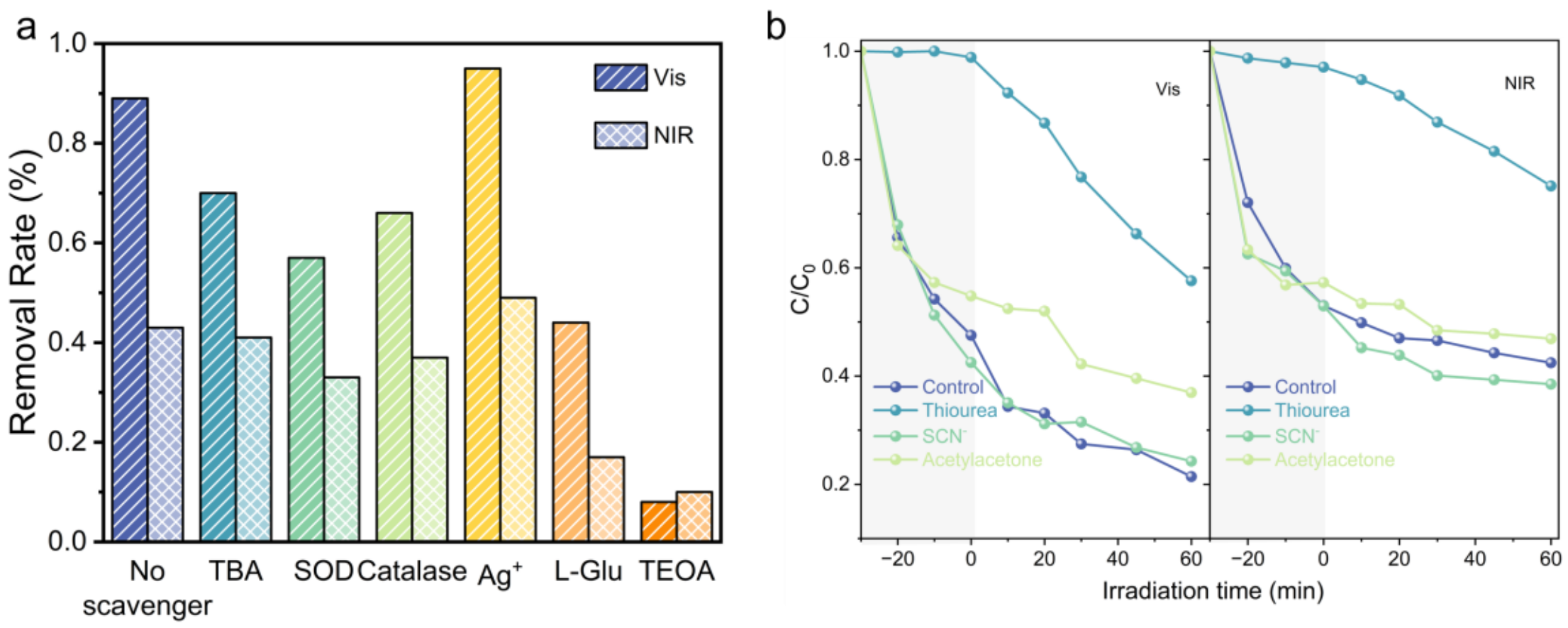

Supplementary Fig.28 | (a) Effects of quenching agents on RhB removal and **(b)** evaluation of active sites through metal masking under Vis and NIR light.

On pages 13 and 14:

"The effects of different scavengers on BPAF degradation are summarized in Table S7. Under both visible and NIR irradiation, tert-butanol (TBA, $k_{\cdot OH} = 5.2 \times 10^{10}$ M^{-1} S^{-1}) has little influence on BPAF removal **(Figure 3f and S27)**, indicating that •OH is not the dominant reactive species, consistent with the absence of DMPO–•OH signals in the EPR spectra[28]. In contrast, the addition of L-tyrosine (L-Tyr, $k{}^{1}O_2 = {\sim}10^8$ M^{-1} S^{-1})[49] significantly suppresses BPAF degradation, leading to a 42% decrease in removal efficiency, whereas superoxide dismutase shows no measurable effect. Triethanolamine (TEOA), a typical h^+ scavenger, almost completely inhibits BPAF removal[14,50]. This strong inhibition arises from the chelation of TEOA with both iron and bismuth species, which blocks hole-driven reactions and suppresses superoxide generation at O_v sites. These results indicate that ${}^1O_2$ and photogenerated h^+ are the main reactive species in this system. For RhB, scavenging •OH, $•O_2^-$, and H_2O_2 only slightly affected the degradation performance, while the quenching of ${}^1O_2$ and h^+ causes clear inhibition under both visible and NIR irradiation **(Figure S28a)**."

Comment 3-12: It is recommended to supplement the contribution ratios of different reactive oxygen species to the removal of BPAF and RhB by the photocatalyst, in order to visually indicate the main active species responsible for pollutant removal.

Response: We appreciate the reviewer's valuable suggestion. We agree that quantitative comparison of the active species can more clearly identify the dominant contributors to pollutant removal. In the revised manuscript, we have added the contribution ratios of the main reactive species based on competitive kinetic analysis, together with the results from EPR, metal masking, and scavenging experiments. These results further confirm that ${}^1O_2$ and photogenerated h^+ are the dominant active species for BPAF and RhB removal.

Changes: *On page 14, the description of the specific contribution rates of ROSs is as follows:*

"For B_7M_3, the contribution ratios of ${}^1O_2$ to BPAF degradation under visible and NIR irradiation are estimated to be 36% and 31%, respectively, while the corresponding h^+ contributions are 64% and 69%. These results demonstrate that BPAF degradation is mainly driven by the synergistic action of ${}^1O_2$ and photogenerated h^+, with h^+ playing a larger role under both light conditions. For RhB degradation, the contributions of ${}^1O_2$ and h^+ are estimated to be 56% and 44%, respectively, further confirming that these two active species dominate the pollutant removal process."

Comment 3-13: The introduction of Nd, the presence of oxygen vacancies, and the construction of the heterojunction all affect the generation of ROS. It is recommended to monitor the concentrations of generated ROSs (·OH, $^1O_2$, $\cdot O_2^-$, H_2O_2), which would help quantitatively understand the enhancement of photocatalytic activity of B_7M_3.

Response: We are grateful for the reviewer's insightful comment. To provide further quantitative evidence, we have added measurements of the main reactive species using ABDA for $^1O_2$, NBT for $\bullet O_2^-$, and KI oxidation for photogenerated h^+. The results of the quenching experiments indicated that neither H_2O_2 nor •OH were the main active species. Moreover, the corresponding signal of •OH was not detected in ESR via DMPO. Additionally, the absence of absorbance values in the titanium sulfate colorimetric experiment suggested that there was almost no hydrogen peroxide in the system. The corresponding experimental procedures have also been added to the Methods section in the Supplementary Information.

Changes: *On page 14, the description of the concentrations of generated ROSs is as follows:*

"Based on the results of the EPR and radical scavenging experiments, $^1O_2$, and h^+ are the primary reactive species in the system. To provide further quantitative evidence, we have added measurements of the main reactive species using ABDA for $^1O_2$, NBT for $\bullet O_2^-$, and KI oxidation for photogenerated h^+ (Table S8). Under visible and NIR light irradiation, the exposed h^+ equivalents are 59.64 and 19.46 μM, respectively, while the $^1O_2$ are 7.2 and 2.9 μM, with superoxide radicals being almost negligible."

Supplementary Materials, pages 8 and 9, the methods of the concentrations of generated ROSs were added:

"The exposure of photogenerated holes was determined using the potassium iodide (KI) oxidation method. A standard curve was first established by measuring the absorbance of triiodide solutions with concentrations ranging from 5 to 100 μM. For the photocatalytic measurement, 30 mg of the catalyst was dispersed in 100 mL of a 10 mM KI aqueous solution. The suspension was irradiated under visible or NIR light. During the 30 min reaction, 3 mL aliquots were collected at selected time intervals and filtered to remove the catalyst

Singlet oxygen was quantified using 9,10-anthracenediyl-bis(methylene)dimalonic acid (ABDA) as the scavenger. A standard curve was prepared using varying concentrations of ABDA diluted in a 0.05 M phosphate-buffered saline (PBS, pH = 7.5). In the typical experimental procedure, 30 mg of the catalyst was added to a mixed solution comprising 10 mL of 0.5 mM ABDA and 90 mL of deionized water. The

suspension was stirred in the dark for 10 min to adsorption equilibrium and then irradiated under visible or NIR light for 30 minutes.

Superoxide radicals were quantified utilizing the nitroblue tetrazolium (NBT) reduction assay. To prepare the standard curve, varying volumes of xanthine oxidase were added to a solution containing NBT, xanthine, and PBS. After reacting at 25 °C for 10 minutes, the reaction was quenched with superoxide dismutase (SOD), followed by extraction with dimethyl sulfoxide (DMSO). For the photocatalytic measurement, 30 mg of the catalyst was added to a solution containing 20 mL of NBT and 80 mL of deionized water. The mixture was stirred in the dark for 5 min and then irradiated under visible or NIR light. After irradiation, the light source was turned off, and 0.5 mL of SOD was immediately added to terminate the reaction. DMSO was then added for extraction, and the absorbance of the final solution was recorded using a UV–Vis spectrophotometer in the wavelength range of 500 – 700 nm."

Comment 3-14: What is the role of Nd? Does Nd exist in the material as ions or as nanoparticles? If it exists as nanoparticles, is there evidence from XRD, HR-TEM, and XPS?

Response: We sincerely thank the reviewer for this important question. Nd exists in the material mainly as ionic Nd^{3+} doped into Nd-BiO_{2-x}, rather than as Nd or Nd oxide nanoparticles. The XRD patterns show no additional diffraction peaks assignable to metallic Nd or Nd oxide phases, indicating the absence of crystalline Nd-based nanoparticles. HRTEM and aberration-corrected STEM images also show no isolated Nd-containing nanoparticles. Instead, elemental mapping shows a homogeneous distribution of Nd together with Bi and O, supporting the uniform incorporation of Nd into the BiO_{2-x} structure. To further clarify the chemical state and local coordination environment of Nd, we added XAFS analysis in the revised manuscript. The results confirm that Nd is present in an ionic form and is preferentially located near oxygen vacancies, forming an asymmetric Nd–O_v–Bi local coordination environment. This local structure helps regulate the electron density around O_v sites and promotes O_2 adsorption and activation

Changes: *On page 5, the description of the exist Nd^{3+} is as follows:*

"Element mapping (Figure 1c and S3d) confirms that Nd-BiO_{2-x} is uniformly anchored on the surface of MIL-101(Fe), with homogeneous distributions of Nd, Bi, Fe, and O. To further elucidate the chemical state of Nd, XAFS analysis was performed (Figure S4), confirming that Nd exists mainly in an ionic form. Neodymium (Nd^{3+}) is preferentially located near O_v in BiO_{2-x}, forming an asymmetric Nd–O_v–Bi local coordination environment[13,16]."

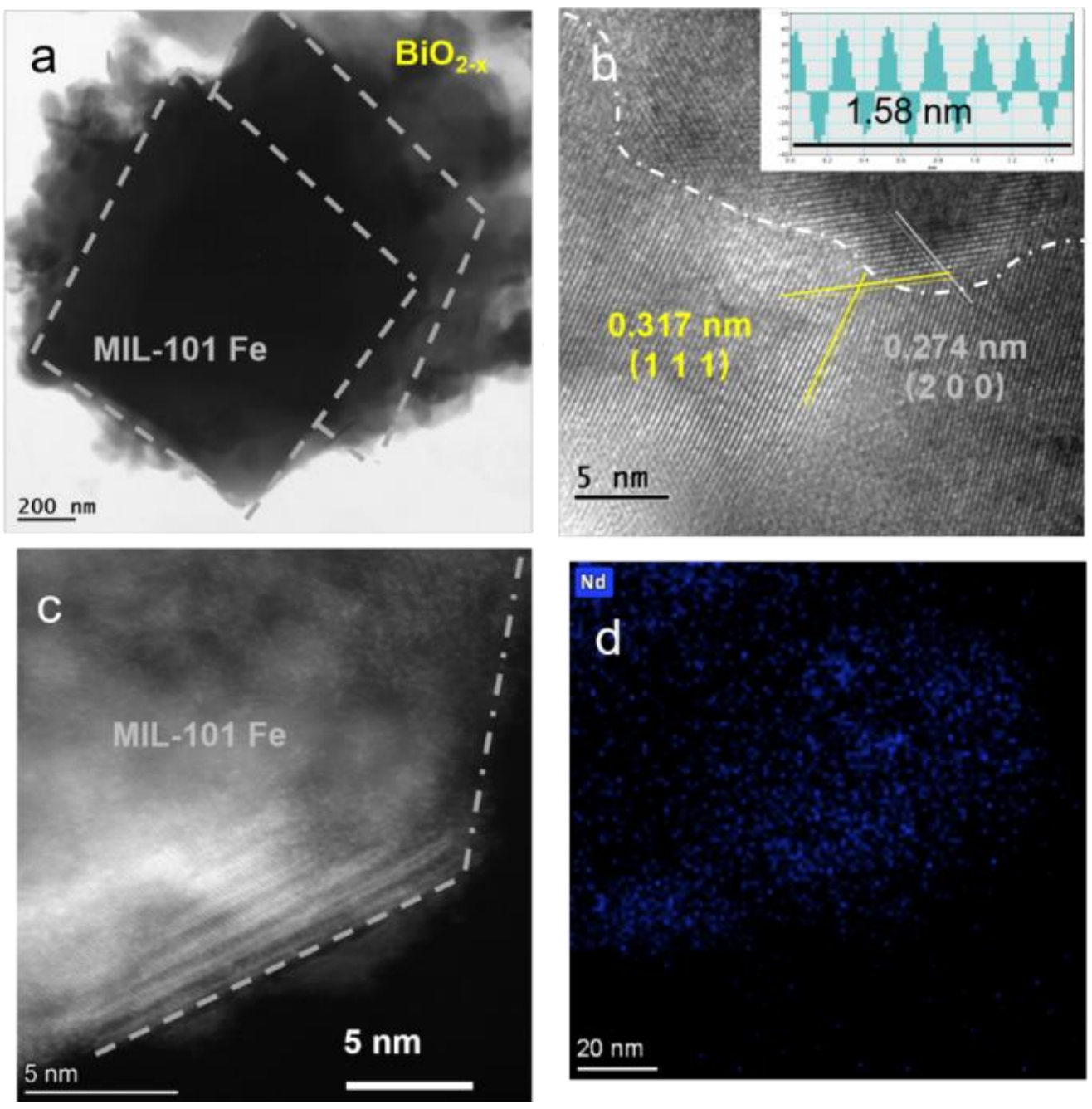

Supplementary Fig.3 | HRTEM and STEM images of the B_xM_y. (a) TEM, (b) HRTEM, (c) aberration-Corrected-TEM images, and (d) STEM mapping of Nd.

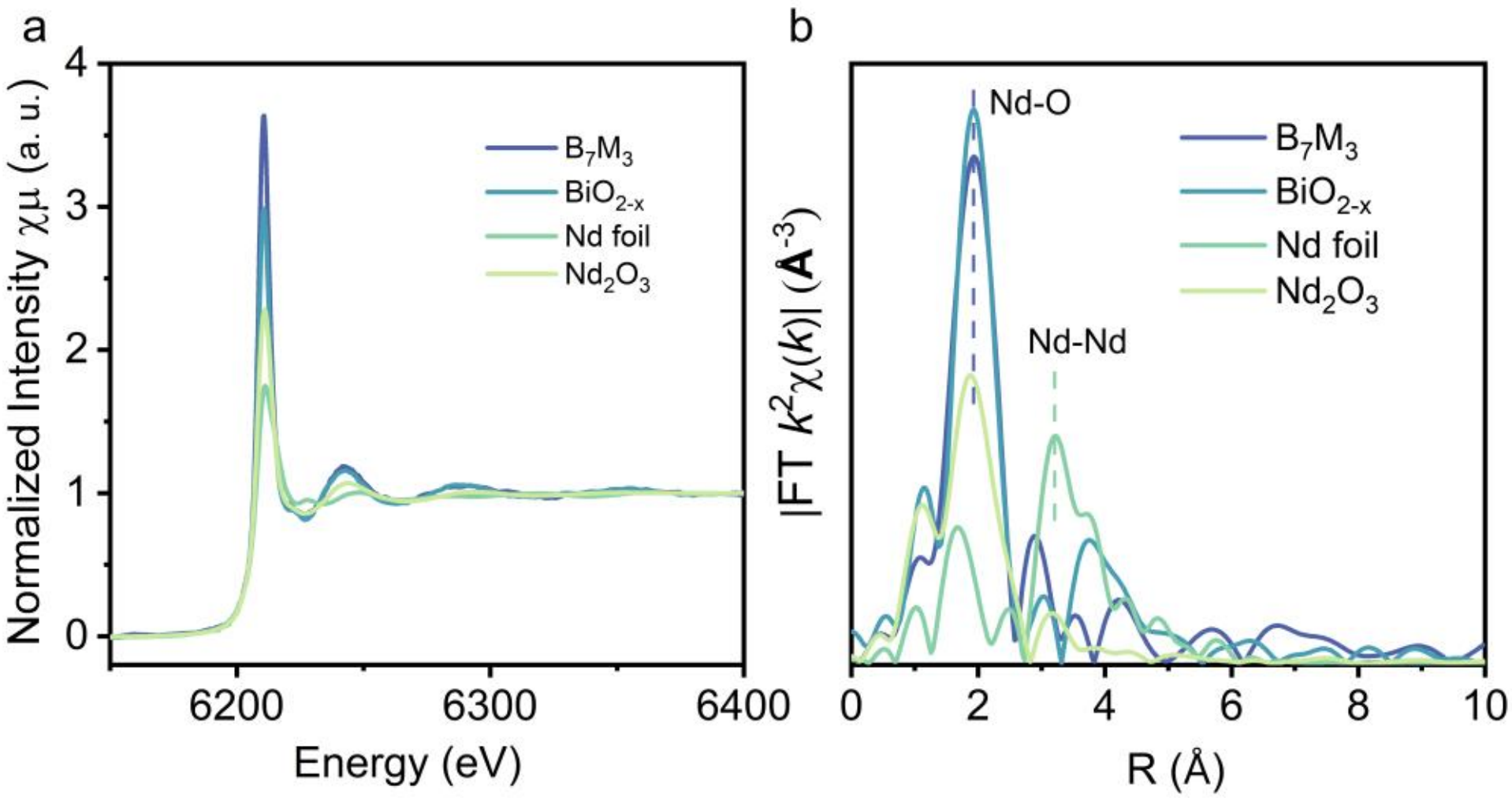

Supplementary Fig.4 | XAS characterization of the Nd element. **(a)** Nd L_3-edge normalized intensity in XANES spectra, and **(b)** k^2-weighted FT-EXAFS spectra of the as-prepared samples.

Comment 3-15: The authors introduced a series of metal chelators to evaluate the roles of metal active sites (Fe ions and Bi ions). In what form does Nd exist in the heterojunction material? Can Nd also serve as a metal active site?

Response: We much appreciate the reviewer for this professional and helpful suggestion. As clarified in the response to **Comment 3-14**, Nd exists mainly as ionic

Nd^{3+} doped into the Nd-BiO_{2-x} lattice rather than as Nd-containing nanoparticles. Therefore, Nd is not considered an independently exposed nanoparticle-type active site that can be selectively evaluated by conventional metal masking agents. In the present system, Nd mainly acts as an electronic and coordination modulator near oxygen vacancies. The Nd-associated Nd–O_V–Bi local structure redistributes the electron density around Ov sites, strengthens the interaction with adsorbed O_2, and promotes O_2 activation. This role is supported by DFT calculations, O_2 adsorption energy analysis, and PDOS results. Because the amount of doped Nd is low and no selective masking agent is available to distinguish Nd sites from neighboring Bi/O_V sites, we did not use metal masking to quantify the independent contribution of Nd. Instead, its role is discussed based on XAFS and DFT analyses.

Changes: *On page 15, the description of oxygen enrichment induced by the imprinting effect and Nd^{3+} is as follows:*

"To evaluate oxygen enrichment induced by the imprinting effect, O_2 adsorption energies were calculated for catalysts with oxygen vacancies and Nd doping **(Figure 3g)**. Compared with stoichiometric BiO_2, BiO_{2-x} shows a lower adsorption energy, decreasing from −1.48 to −1.71 eV, with O_2 adopting a Pauling-type configuration. Introducing Nd near oxygen vacancies forms an asymmetric Nd–O_V–Bi structure and further reduces the adsorption energy to −2.09 eV. After heterojunction formation, electron transfer from BiO_{2-x} to MIL-101(Fe) drives a transition of O_2 adsorption from Pauling-type to Yeager-type, resulting in a much stronger adsorption energy (−6.45 eV). These results suggest that ionic Nd^{3+} modulates the electronic and coordination environment of O_V sites, making them electron-rich centers for O_2 adsorption and activation under irradiation."

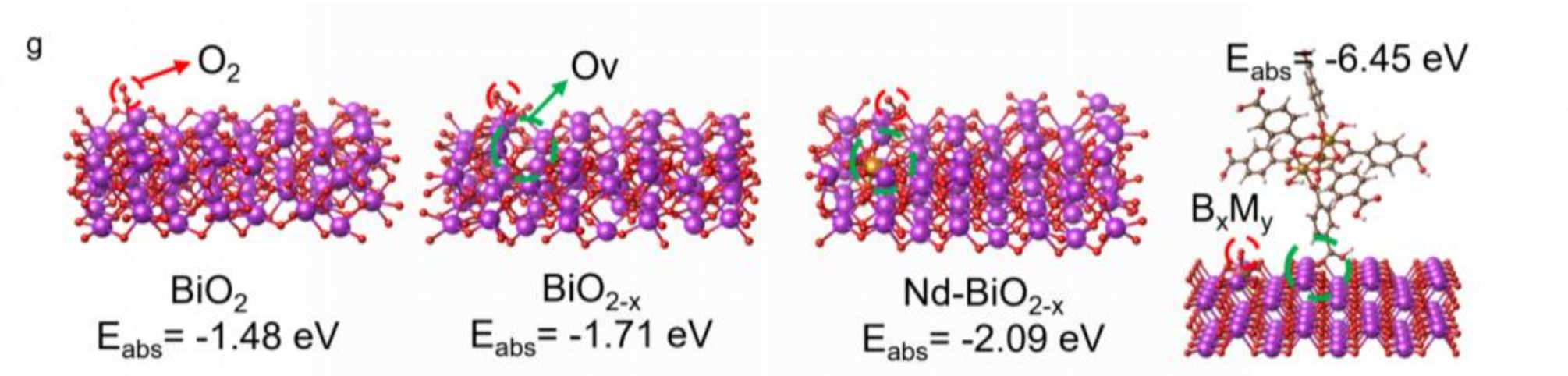

Figure 3 | Photocatalytic performance of B_xM_y. (g) The O_2 adsorption energies of as-prepared samples.

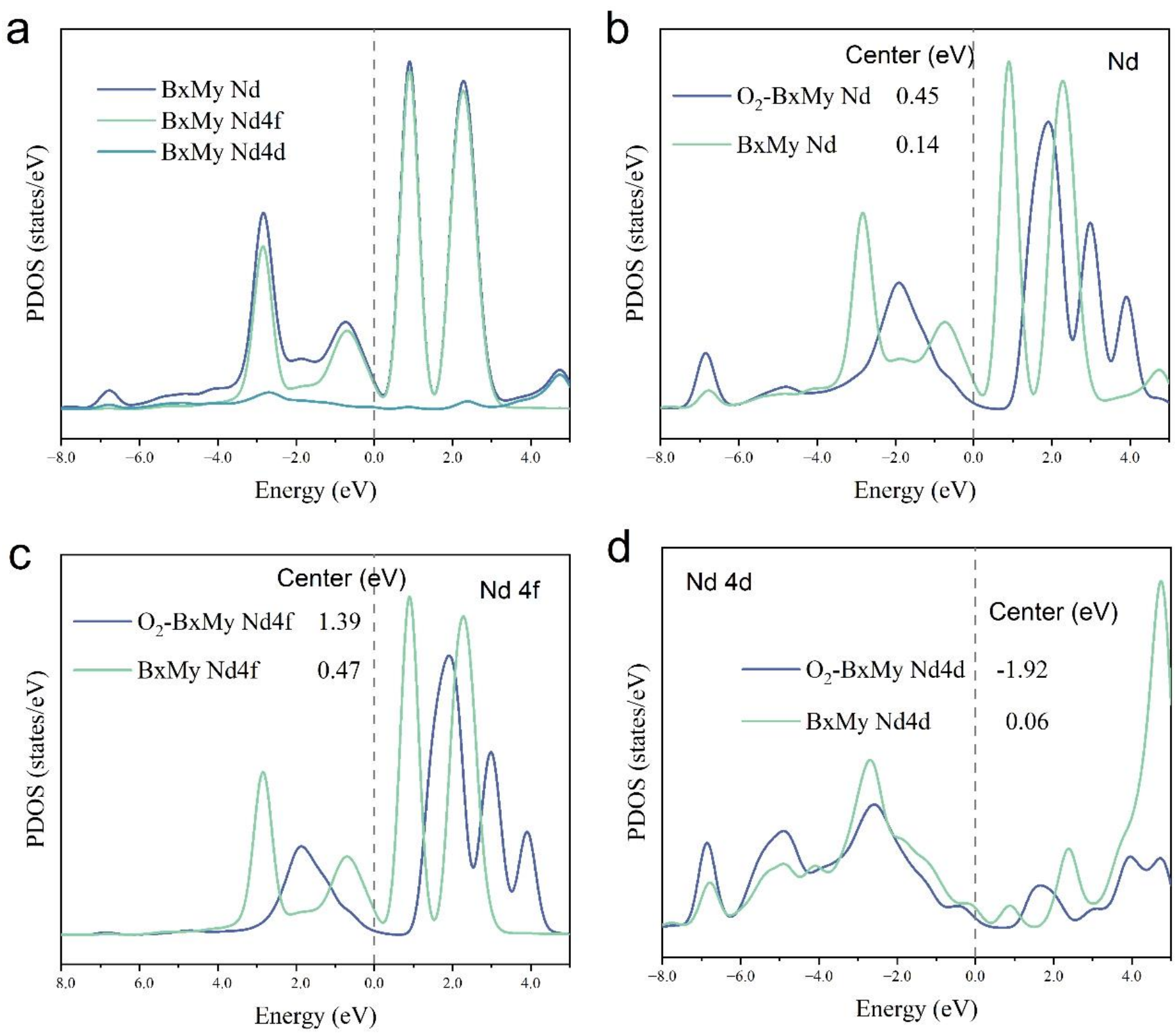

Supplementary Fig.31 | The Nd PDOS of the optimized configurations of BxMy. **(a)** The different Nd PDOS of the optimized configurations of BxMy. **(b-d)** The PDOS of Nd, Nd 4f, and Nd 4d before and after O_2 adsorption.

Comment 3-16: When investigating changes in metal active sites, h^+, and oxidation potential, only the photocatalytic reaction of the material with BPAF was considered. What about the reaction of the material with RhB?

Response: We sincerely thank the reviewer for pointing out this important issue. In the revised Supplementary Information, we have added metal masking experiments for RhB removal under visible and NIR irradiation to further evaluate the role of metal active sites. The added results show that thiourea causes the strongest inhibition among the tested masking agents, suggesting that Bi-related sites play a dominant role in RhB oxidation. Combined with the RhB quenching results in **Comment 3-11**, these data indicate that RhB degradation is mainly driven by Bi-site-assisted activation and the reactive species $^1O_2$ and h^+. These additional experiments provide a more complete comparison between BPAF and RhB degradation pathways

Changes: *Supplementary Materials, page 26, evaluation of active sites through metal masking under Vis and NIR light were added:*

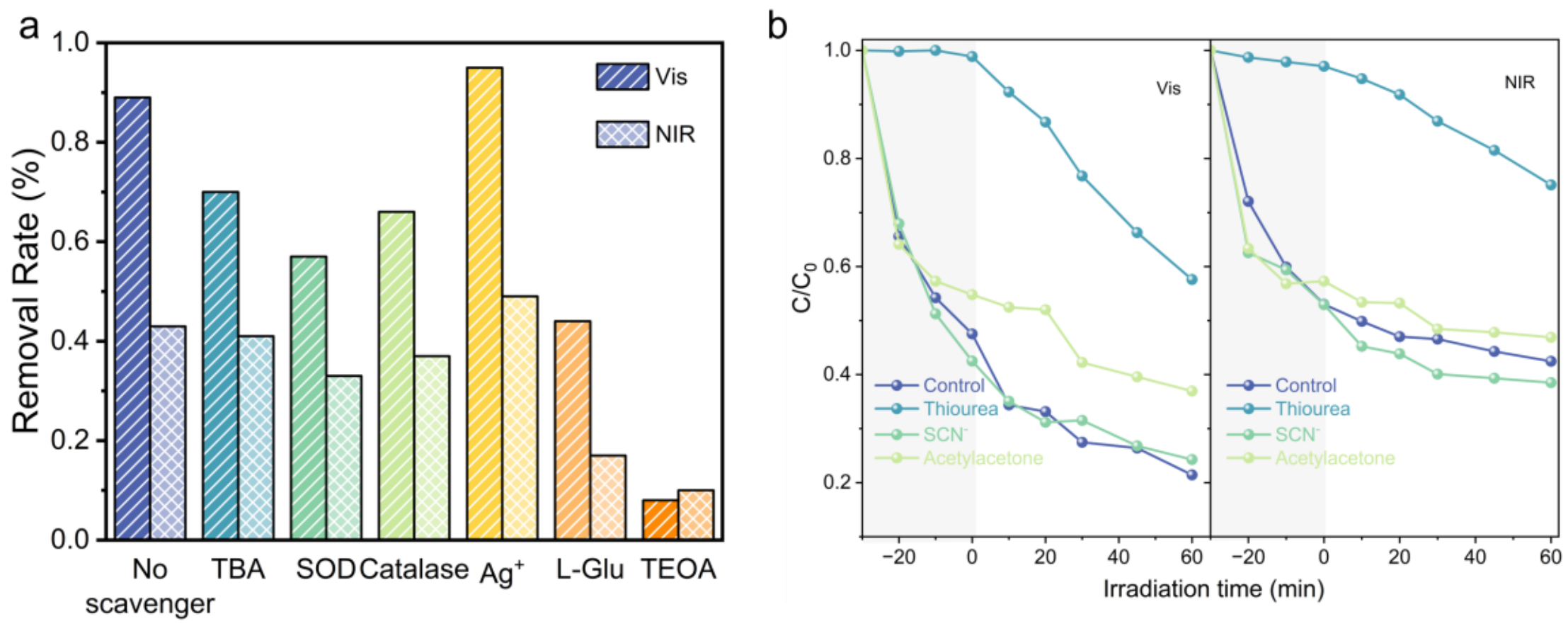

Supplementary Fig.28 | (a) Effects of quenching agents on RhB removal and **(b)** evaluation of active sites through metal masking under Vis and NIR light.

On page 15, the investigating changes in metal active sites, h^+, and oxidation potential for BPAF and RhB are as follows:

"Directional migration of photogenerated electrons enhances the oxidative ability of photogenerated holes in the valence band[53,54]. To assess the roles of h^+ and metal active sites, a series of metal chelating agents was introduced **(Figure 4a,b)**, and the stability constants of the corresponding complexes are listed in **Table S9**. The addition of SCN^- decreases BPAF removal by 11 and 28 percentage points under visible and near-infrared light, respectively, indicating partial suppression of metal-centered reactions. In dark conditions, acetylacetone increases BPAF adsorption to about 30% and, under illumination, provides a continuous electron source to Fe^{3+} in B_7M_3, leading to a photocatalytic removal of approximately 12%. Acetylacetone coordinates with iron ions in a bidentate mode, replacing surface water and hydroxyl groups and increasing surface hydrophobicity. Electron transfer from acetylacetone through Bi–O=C bonds to oxygen vacancies promotes oxygen radical generation and accelerates early-stage BPAF degradation. In contrast, thiourea addition results in pronounced decreases in removal efficiency, by 28% under visible light and 32% under near-infrared light. Thiourea preferentially binds to bismuth species and can also quench superoxide radicals in solution. For RhB removal, thiourea also shows the strongest inhibitory effect among the tested masking agents **(Figure S28b)**, suggesting that Bi-related sites play a major role in dye oxidation. These results indicate that Bi-site-assisted activation, $^1O_2$, and photogenerated h^+ dominate RhB degradation, while Fe-related hole-mediated reactions provide a secondary contribution."

Comment 3-17: Why did the authors choose BPAF and RhB as two typical water pollutants?

Response: We sincerely thank the reviewer for this helpful question. BPAF and RhB were selected as representative target pollutants for different purposes. BPAF is a widely used bisphenol substitute in plastic-related products and has raised environmental concern because of its toxicity and endocrine-disrupting potential. Compared with conventional dye pollutants, BPAF is also more difficult to degrade, making it a suitable target to evaluate the practical photocatalytic ability of B_7M_3. RhB was selected as a common dye pollutant and model compound in photocatalytic studies, allowing us to further verify whether the adsorption–photocatalysis synergy of the heterojunction is also effective for dye degradation. We have clarified this point in the revised manuscript.

Changes: *On page 11, the reason of the BPAF and RhB as two typical water pollutants is as follows:*

"BPAF and RhB were selected as representative target pollutants to evaluate the adsorption and photocatalytic performance, with BPAF serving as a toxic bisphenol substitute and RhB as a common photocatalytic model compound."

Comment 3-18: The authors used DFT calculations to explore the charge transfer process of the heterojunction, highlighting the role of O_v. What exactly is the role of Nd? How should it be explained?

Response: We are grateful for the reviewer's insightful comment. As clarified in the responses to Comments 3-14 and 3-15, Nd exists mainly as ionic Nd^{3+} doped into the Nd-BiO_{2-x} lattice rather than as Nd-containing nanoparticles. Its role is not to act as an isolated metal nanoparticle active site, but to regulate the local electronic and coordination environment around oxygen vacancies. The DFT results show that introducing Nd near O_v forms an asymmetric Nd–O_v–Bi local structure, which strengthens O_2 adsorption and promotes O_2 activation. To further clarify this role, we added PDOS analysis before and after O_2 adsorption. The results show clear changes in the Nd 4f states after O_2 adsorption, indicating orbital interaction between Nd-associated sites and adsorbed O_2. This electronic modulation supports the role of Nd in enhancing O_2 activation at vacancy-rich sites and thereby facilitating reactive species generation.

Changes: This point has been clarified in the revised DFT discussion in the Supplementary Materials, which was also updated in response to Comments 2-3 and 3-15. To maintain consistency, the relevant description is based on the same revised paragraph and is shown below:

Supplementary Materials, on page 28, the description of Nd DFT is as follows:

"To further clarify the atomic-level interaction between Nd and adsorbed O_2, the PDOS before and after O_2 adsorption was calculated by DFT. As shown in **Figure S31a**, the electronic states of Nd are mainly dominated by Nd 4*f* orbitals. After O_2 adsorption, the most obvious change occurs in the Nd 4*f* orbitals (**Figure S31c**), where the 4*f*-band center shifts positively from 0.47 to 1.39 eV. This upward shift indicates orbital hybridization between Nd 4*f* and O_2-derived O 2*p* states, reflecting strong electronic interaction between Nd-associated sites and adsorbed O_2. Such electronic modulation supports the chemisorption and activation of O_2 near the Nd–O_v–Bi local structure, thereby facilitating the subsequent generation of reactive species. These results indicate that Nd enhances the catalytic process by regulating the electronic structure of O_v-rich Bi sites rather than acting as a separate nanoparticle active site."

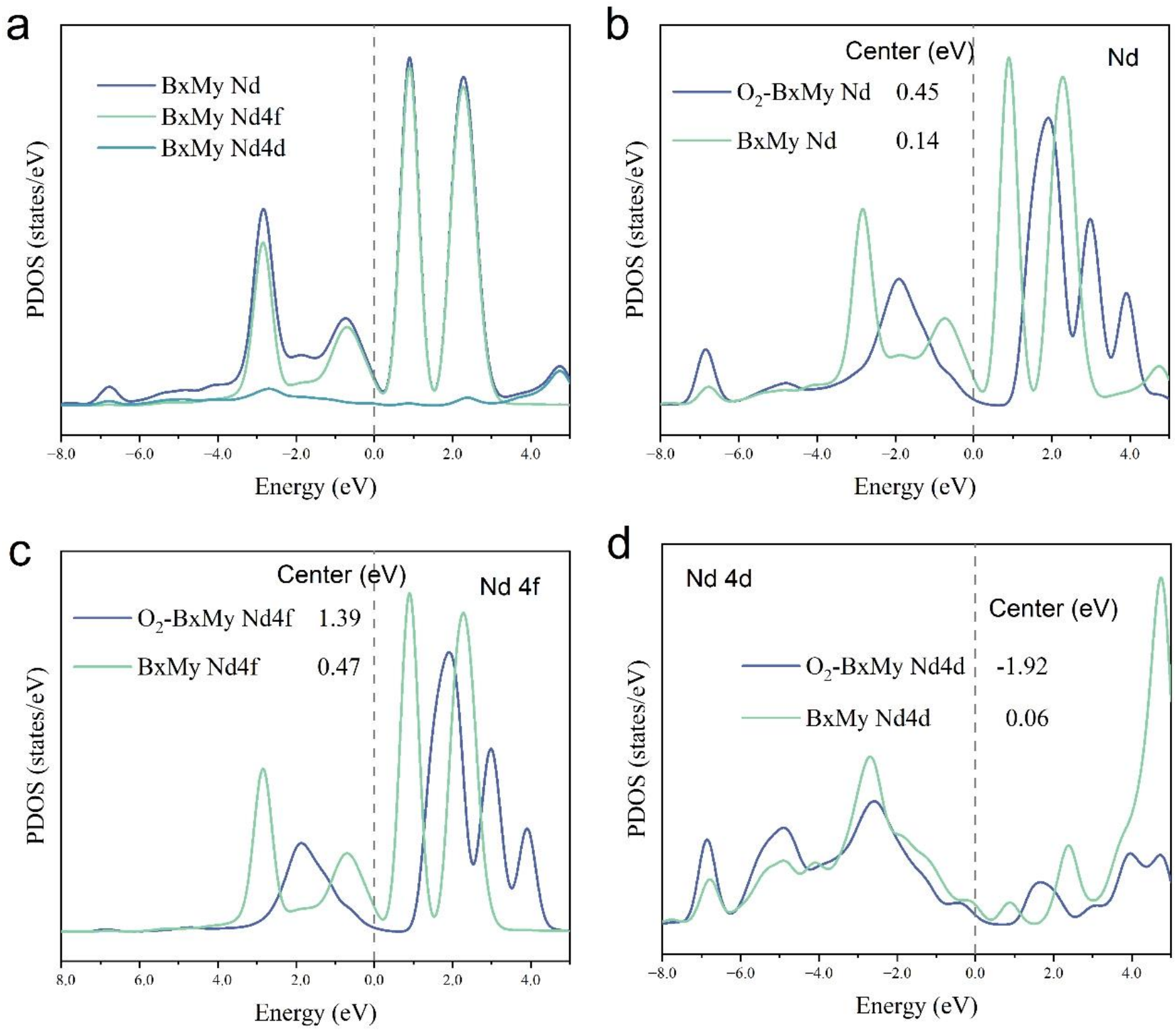

Supplementary Fig.31 | The Nd PDOS of the optimized configurations of BxMy. **(a)** The different Nd PDOS of the optimized configurations of BxMy. **(b-d)** The PDOS of Nd, Nd 4f, and Nd 4d before and after O_2 adsorption.

Comment 3-19: The authors demonstrated the formation of Bi-O=C interfacial bonds through the charge density difference diagram. It is recommended to supplement the electron localization functon (ELF) to visually determine whether electrons are transferred from one atom to another.

Response: We appreciate the reviewer's helpful suggestion, and we agree with it. In addition to the charge density difference analysis, binding energy and IGMH calculations were previously used to evaluate the strength of the interfacial interaction. To further visualize the bonding character at the interface, we have now added ELF calculations. The ELF results provide additional evidence that the interaction between the carboxyl O atoms and Bi sites near oxygen vacancies is significantly strengthened in Nd-BiO_{2-x}/MIL-101(Fe), supporting the formation of Bi–O=C interfacial bonds. We have revised the corresponding DFT discussion accordingly.

Changes: *Supplementary Materials, on page 29, the ELF of BiO_2 and BiO_{2-x} is as follows:*

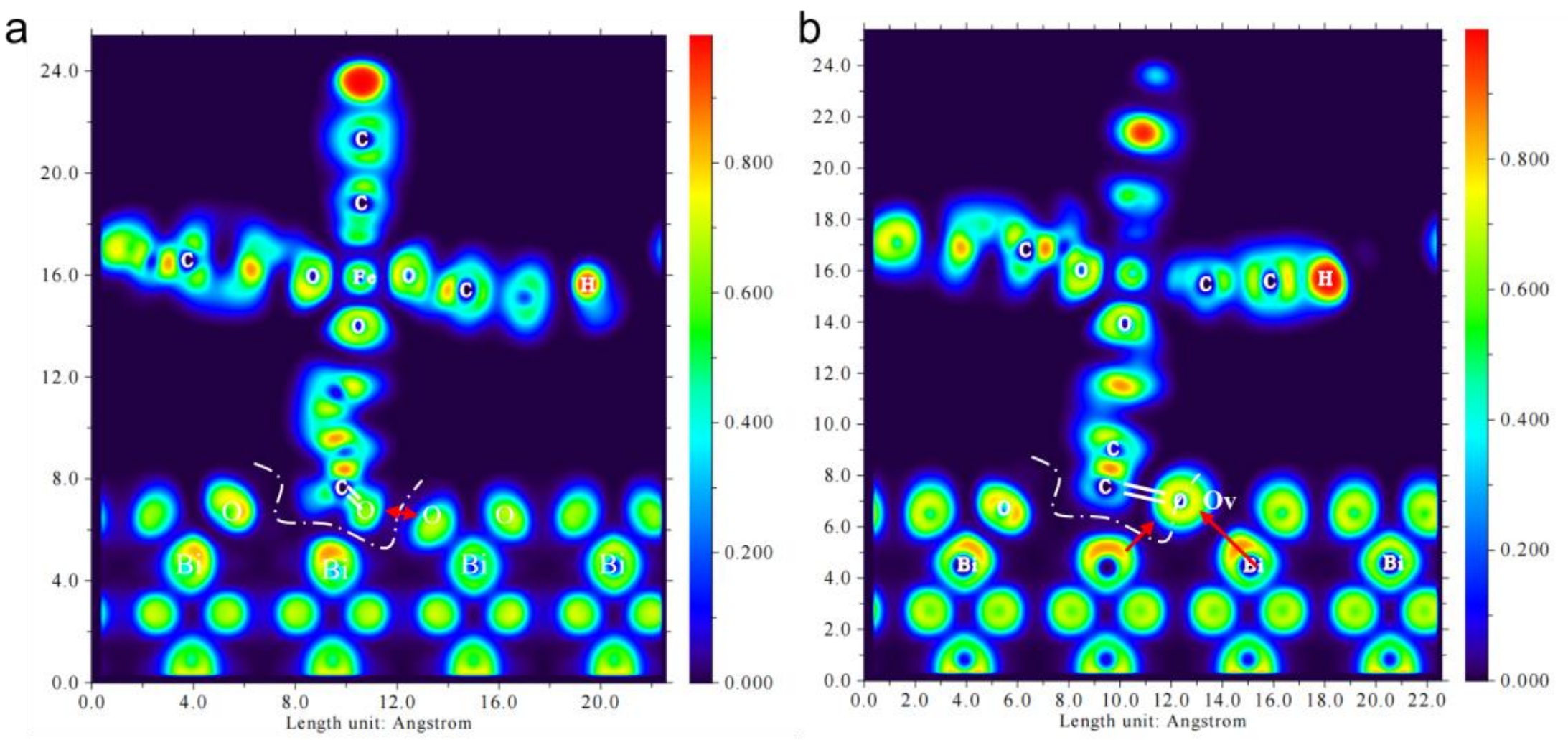

Supplementary Fig.34 | The ELF of MIL-101(Fe) clusters on the $(01\bar{1})$ surface of **(a)** BiO_2 and **(b)** BiO_{2-x}.

On pages 17 and 18, the descriptions of binding energy, IGMH, and ELF are as follows:

"Compared with BiO_2 without vacancies, the binding energy increases from 10.28 to 17.87 eV for BiO_{2-x}, indicating stronger interfacial interactions. Independent gradient model based on Hirshfeld partitioning (IGMH) shows a δg^{inter} value of 0.01 and 0.04 for the BiO_2/MIL-101(Fe) and BiO_{2-x}/MIL-101(Fe) interface **(Figure 4i and S33)**, further confirming the enhanced interfacial interaction induced by oxygen vacancies. To further examine the bonding character, electron localization function (ELF) calculations were performed **(Figure S34)**. The ELF map shows enhanced electron localization in the interfacial region of BiO_{2-x}/MIL-101(Fe) compared with BiO_2/MIL-101(Fe), indicating stronger interfacial electronic coupling induced by oxygen vacancies, supporting the formation of Bi–O=C interfacial bonds."

Comment 3-20: Was dipole correction considered when calculating the work function?

Response: We appreciate the reviewer's careful comment. The work functions were recalculated with dipole corrections included, and the corrected values have been added to **Supplementary Fig. 18**. Although the values show a slight increase compared with the previous results, the relative trend remains unchanged. MIL-101(Fe) has a higher work function (5.44 eV) than Nd-BiO_{2-x} (5.18 eV), confirming electron transfer from Nd-BiO_{2-x} to MIL-101(Fe) under dark equilibrium. The related discussion has been revised consistently with the responses to **Comments 3-2** and **3-3**.

Changes: *Supplementary Materials, on page 21, the work function is as follows:*

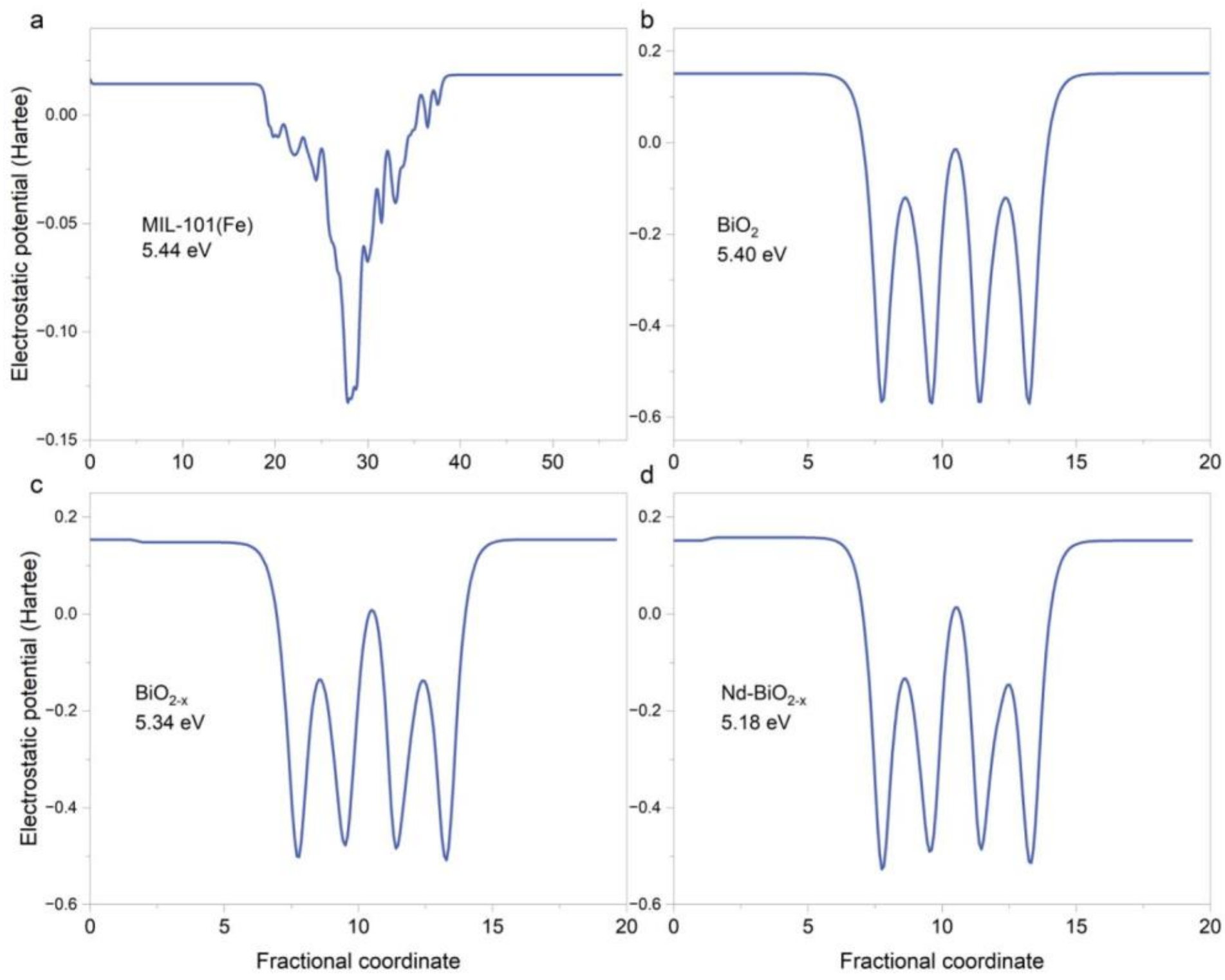

Supplementary Fig.18 | Electrostatic potentials of BiO_2, BiO_{2-x}, Nd-BiO_{2-x}, and MIL-101(Fe).

On pages 9 and 10, the work function and charge transfer discussion has been revised consistently with the changes made in response to Comments 3-2 and 3-3, as follows:

"In n-type semiconductor photocatalysts, electrons are the dominant charge carriers and play a central role in catalytic reactions. The energy level alignment between Nd-BiO_{2-x} and MIL-101(Fe) is schematically shown in **Figure 2d**. Density functional theory calculations with dipole corrections were used to determine the work functions of the two components **(Figure S18)**. The calculated work function of MIL-101(Fe) is 5.44 eV, which is higher than that of Nd-BiO_{2-x} (5.18 eV), indicating that electrons migrate from Nd-BiO_{2-x} to MIL-101(Fe) upon contact to equilibrate their Fermi levels[39,40]. As a result, Nd-BiO_{2-x} becomes positively charged, while MIL-101(Fe) becomes negatively charged, establishing a built-in electric field at the interface. Under

illumination, this built-in electric field facilitates directional charge migration and spatial separation of photogenerated carriers, driving the direct Z-scheme pathway in which electrons accumulate on Nd-BiO_{2-x} and holes remain on MIL-101(Fe)[41]. In addition, O_v-associated undercoordinated Bi/Nd sites introduce localized defect states and act as electron-accumulation centers on the Nd-BiO_{2-x} surface, facilitating subsequent redox reactions[11–13]."

Comment 3-21: Should the reaction mechanism for the photocatalytic removal of RhB by the heterojunction material also be explained?

Response: We agree with the reviewer that this issue requires further clarification. In the revised manuscript, we have added a discussion of the photocatalytic removal mechanism of RhB, based on the EPR results, quenching experiments, reactive species quantification, and contribution ratio analysis. These results show that RhB degradation follows a mechanism similar to BPAF degradation, with $^1O_2$ and photogenerated h^+ as the dominant active species, while $•O_2^-$ shows only a minor contribution. The difference lies mainly in the relative contribution of these active species and the molecular structure of the pollutant. Compared with BPAF, RhB contains an electron-rich conjugated dye structure, which is more susceptible to oxidation by $^1O_2$ and h^+. We have clarified this point in the revised manuscript.

Changes: *On pages 14 and 15, the discussion of the photocatalytic removal mechanism of RhB has been revised consistently with the reactive species analysis in response to Comments 3-11 and 3-12, as follows:*

"For B_7M_3, the contribution ratios of $^1O_2$ to BPAF degradation under visible and NIR irradiation are estimated to be 36% and 31%, respectively, while the corresponding h^+ contributions are 64% and 69%. These results demonstrate that BPAF degradation is mainly driven by the synergistic action of $^1O_2$ and photogenerated h^+, with h^+ playing a larger role under both light conditions. For RhB degradation, the contributions of $^1O_2$ and h^+ are estimated to be 56% and 44%, respectively, further confirming that these two active species dominate the pollutant removal process. The different contribution ratios are related to the molecular structures of the two pollutants. BPAF contains fluorinated aromatic rings, where the electron-withdrawing fluorine groups reduce the electron density of the aromatic structure and make electrophilic oxidation more difficult. In contrast, RhB has an electron-rich conjugated chromophore, which is more readily attacked by $^1O_2$ and directly oxidized by photogenerated h^+. Therefore, RhB removal mainly proceeds through oxidative destruction of the chromophoric conjugated structure, while BPAF degradation requires stronger hole-driven oxidation together with $^1O_2$-mediated transformation."

Comment 3-22: Format issues in the manuscript should be further checked and corrected (e.g., the font and format of figures and tables should be unified; subscripts and superscripts should be carefully reviewed).

Response: We appreciate the reviewer's careful reading. We have thoroughly checked the manuscript, figures, tables, and Supplementary Information, and corrected the related formatting issues. The fonts and formats in the figures and tables have been unified, and the subscripts and superscripts have been carefully reviewed and corrected.
